# Immunogenomic diversity of triple-negative breast cancers in obese and non-obese black and white women

**DOI:** 10.1038/s41523-025-00836-6

**Published:** 2025-11-12

**Authors:** Fokhrul Hossain, Denise Danos, Jovanny Zabaleta, Xiao-Cheng Wu, Luis Del Valle, Chindo Hicks, Jiande Wu, Jerneja Tomsic, Susan Neuhausen, Yuan Chun Ding, Kavitha Mukund, Zahra Mesrizadeh, Shankar Subramaniam, Augusto Ochoa, Victoria Seewaldt, Lucio Miele

**Affiliations:** 1 Department of Genetics, Louisiana State University Health Center, New Orleans, LA USA; 2 School of Public Health, Louisiana State University Health Center, New Orleans, LA USA; 3 Department of Interdisciplinary Oncology, Louisiana State University Health Center, New Orleans, LA USA; 4 Louisiana SEER Tumor Registry, New Orleans, USA; 5 Department of Population Sciences, City of Hope Comprehensive Cancer Center, Duarte, CA USA; 6 Department of Bioengineering, University of California, San Diego, USA; 7 Louisiana State University-Louisiana Children’s Medical Center Health (LSU-LCMC Health) Cancer Center, New Orleans, LA USA; 8 Present Address: Department of Public Health Sciences, Clemson University, Clemson, SC USA

**Keywords:** Cancer, Breast cancer, Cancer genomics, Cancer microenvironment

## Abstract

Racial disparities in incidence and outcomes of triple-negative breast cancer (TNBC) have been attributed to ancestry, socioeconomic factors and/or obesity. We studied 253 TNBCs from 128 Black and 125 White women. Arms were balanced for age, AJCC stage, histological grade and molecular subtypes, and differed significantly in BMI distribution, obesity, and Area Deprivation Index. We examined survival rates, whole-transcriptome RNASeq and genetic ancestry. In our sample, Black race or obesity were not intrinsically predictive of poor outcomes. TNBC molecular portraits varied with stage and biological aggressiveness, irrespective of race. We identified a novel group of TNBCs with a distinctive luminal-like and stem-cell-like transcriptional profile that were equally distributed among Blacks and Whites. Genetic ancestry was highly admixed. Immune deconvolution showed a higher abundance of several immune cell populations in tumors from Blacks, which may have precision therapeutic significance, warranting further investigation of potential drivers of tumor immunity.

## Introduction

Triple-negative breast cancer (TNBC) is a heterogeneous group of clinically aggressive tumors. TNBCs have a high risk of recurrence and metastasis, with limited options for targeted treatment^1–3^. Several studies suggested higher TNBC incidence and/or mortality rates among self-identified Black women compared to other racial/ethnic groups. Recent publications suggested that risk for TNBC^4–6^ and hereditary susceptibility for TNBC^7^ may be linked to West African/African ancestry. A recent study identified a 59-gene “race-specific” signature that discriminated between breast cancers from Black and White women^8^. None of these genes were associated with differential survival after controlling for multiple comparisons. However, that study included tumors of all molecular subtypes and did not focus on TNBC.

Racial/ethnic minorities are more likely to be exposed to unfavorable physical and socioeconomic environments that increase health risks^9–11^. Numerous studies suggest that chronic stress associated with an adverse neighborhood environment affects health through multiple mechanisms^12–16^. Using Louisiana Tumor Registry (LTR) data, we previously reported an association between neighborhood concentrated disadvantage index (CDI) and racial disparities in TNBC outcomes^17^.

The immune microenvironment contributes to tumor biology, transcriptional profiles and clinical outcomes. Using TCGA and METABRIC data, Liu et al. found that TNBCs had higher immune cell infiltration, including immunosuppressive cells, as well as higher expression levels of inflammation-promoting and metastasis-promoting genes, compared to non-TNBC breast cancers^18^. A recent immunogenomic study identified a tumor immune risk score (TIRS) panel containing eight potential biomarkers that stratifies high- and low-risk TNBC^19^. This study also identified four tumor immune microenvironment types (TIMTs) predictive of clinical outcomes^19^. In White women, the expression of immunologically relevant transcripts predicts good response to neoadjuvant chemotherapy (NACT)^20^. However, it is unclear whether the same transcripts predict a similar response in Black women. Martini et al. recently described ancestry-associated gene expression profiles in TNBC associated with population-based immune stroma landscapes^21^.

Obesity has been linked to increased risk of TNBC^22^ and poor response to NACT^23^. In a case-control study, including 2295 breast cancer cases (364 TNBC) and 8779 matched controls, we^24^ showed that in our sample, obesity was not associated with increased TNBC risk. Conversely, type 2 diabetes was strongly associated with TNBC risk irrespective of race or age. However, that study did not address TNBC outcomes or tumor biology. We aimed to understand the relationships between race, obesity, tumor molecular portraits, immunogenomic signatures, and clinical outcomes in Louisiana women with TNBC. We profiled the transcriptome of 253 TNBCs from 128 Black and 125 White women. Arms were balanced for age, AJCC stage, histological grade and molecular subtypes, and differed significantly in BMI distribution, obesity, and Area Deprivation Index. Genetic ancestry was highly admixed, with only 50% of Black women having ≥80% West African ancestry and 64% of White women having ≥80% Western European ancestry. We asked whether race-associated or obesity-associated differences existed in TNBC subtype distribution or gene expression profiles. Additionally, we asked whether gene expression profile differences existed based on AJCC tumor stage and clinical aggressiveness as determined by 2-year survival. Finally, we used two distinct bioinformatic deconvolution algorithms to estimate the distribution of immune microenvironment cell populations based on race, obesity, genetic ancestry, age, AJCC stage and clinical aggressiveness.

## Results

Table 1 shows the basic demographic, clinico-pathological and molecular characteristics of the tumors we studied. The study sample from which our tumors were selected is shown in Supplementary Table 1.

**Table 1:** Demographics of our sample, clinico-pathological and molecular characteristics of TNBC tumors

	All		Black		White		
	%	*n*	%	*n*	%	*n*	*p* value
All	100.0	253	100.0	128	100.0	125	
Age, *y*							0.1390
Mean, std	62.2	12.8	61.0	11.9	63.4	13.5	
Age							0.6204
Less than 50	17.4	44	18.8	24	16.0	20	
50 or older	82.6	209	81.3	104	84.0	105	
BMI							0.0045
Lean	30.4	77	21.1	27	40.0	50	
Obese I	31.6	80	32.0	41	31.2	39	
Obese II	20.2	51	24.2	31	16.0	20	
Obese III	17.8	45	22.7	29	12.8	16	
Obese							0.0016
No	30.4	77	21.1	27	40.0	50	
Yes	69.6	176	78.9	101	60.0	75	
AJCC stage							0.0960
I	41.1	104	35.2	45	47.2	59	
II	47.0	119	50.0	64	44.0	55	
III	9.1	23	12.5	16	5.6	7	
IV	2.8	7	2.3	3	3.2	4	
Late stage							0.0562
No	41.1	104	35.2	45	47.2	59	
Yes	58.9	149	64.8	83	52.8	66	
Grade							0.5891
1	2.4	6	2.3	3	2.4	3	
2	11.5	29	12.5	16	10.4	13	
3	82.2	208	82.8	106	81.6	102	
Unknown	4.0	10	2.3	3	5.6	7	
2-year survival							0.5812
No	13.4	34	14.8	19	12.0	15	
Yes	84.2	213	82.8	106	85.6	107	
Censored	2.4	6	2.3	3	2.4	3	
TNBC type							0.1830
Basal-like 1	14.6	37	13.3	17	16.0	20	
Basal-like 2	7.1	18	7.0	9	7.2	9	
Immuno-modulatory	19.4	49	20.3	26	18.4	23	
Luminal androgen receptor	5.5	14	7.0	9	4.0	5	
Mesenchymal	13.8	35	15.6	20	12.0	15	
Mesenchymal stem-like	16.6	42	10.9	14	22.4	28	
Unclassified	7.9	20	10.9	14	4.8	6	
Excluded	15.0	38	14.8	19	15.2	19	
Area deprivation index national rank							<0.0001
Median (IQR), *n*	76 (48–89)	251^a^	84 (67–92)	126^a^	56 (37–80)	125	

*BMI* body mass index, *AJCC* American Joint Commission on Cancer, *TNBC* Triple negative breast cancer, *IQR* Inter-quartile range.

^a^Area deprivation index could not be computed for 2 patients.

The two arms were not significantly different in age, AJCC stage, histological grade, 2-year survival, or molecular subtype as defined by TNBCtype^25,26^. We used the original 7-subtype Lehmann classification because the tumor microenvironment was a focus of our study. Genes expressed in the microenvironment contributed to the definition of 2 of the original subtypes^27^, and differences in NACT response among the original 7 subtypes were described^28^. The two arms differed significantly in BMI distribution and obesity rates, with Black women showing a higher prevalence of obesity and generally higher BMIs. The most significant difference between Black and White women was area-based socioeconomic disadvantage, as represented by ADI (Table 1, *p* < 0.0001). The prevalence of later-stage tumors (AJCC 2–4) as opposed to Stage 1 approached statistical significance (*p* = 0.0562).

### Transcriptomic analyses

**Fig. 1:**
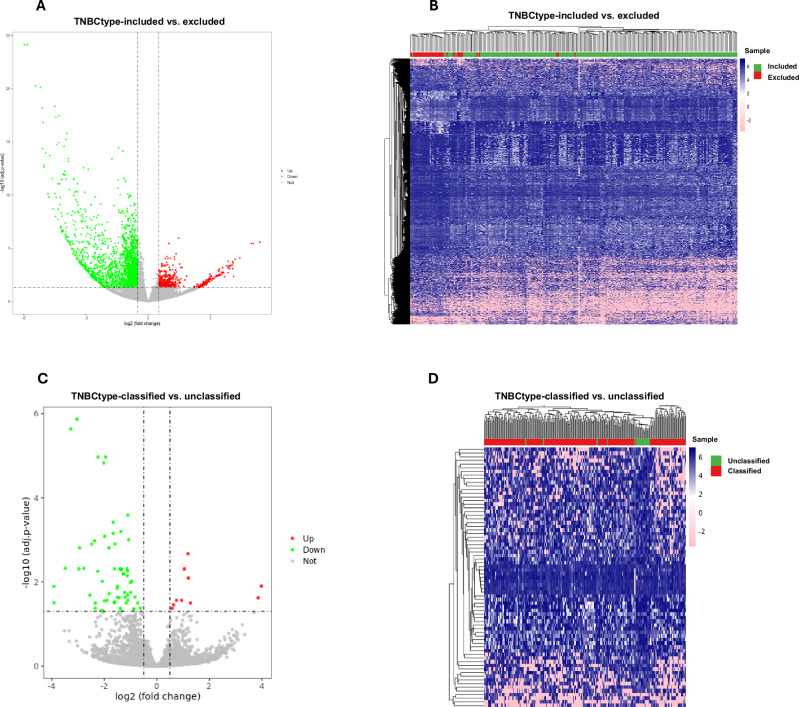
TNBC-excluded tumors have distinctive lumino-basal transcriptomic profiles. **A** Volcano plot and **B** Heatmap showing the subset of tumors excluded from TNBC-type analysis and characterized by a highly distinctive gene expression profile. **C** Volcano plot and **D** Heatmap showing transcripts significantly different between TNBC-type-classified and unclassified tumors in our sample.

We classified tumors using the TNBCtype algorithm^26^. However, the algorithm excluded 38 tumors from analysis. These tumors were confirmed to be TNBC by IHC. Their GEPs were clearly distinct from the tumors included in the analysis by the algorithm (Fig. 1A, B and Supplementary Table 2). The 3406 differentially expressed transcripts included a Notch signaling signature (high DLL1, HES1, and DTX3, low DLL3), as well as transcripts linked to estrogen signaling (TFF1, AHR), luminal B breast cancers (POTED), VEGFA, EGFR, BRAF, TGFB1, and several cytokines (e.g., IL17, IL12B, IL21, IL18). IPA (Supplementary Table 3) identified numerous pathways distinguishing TNBC-type-excluded from TNBC-type-included tumors. These included cyclin-cell cycle pathways, estrogen signaling, Notch, WNT, Sonic Hedgehog, TGF-β, HIPPO, and other cell fate regulatory pathways.

**Fig. 2:**
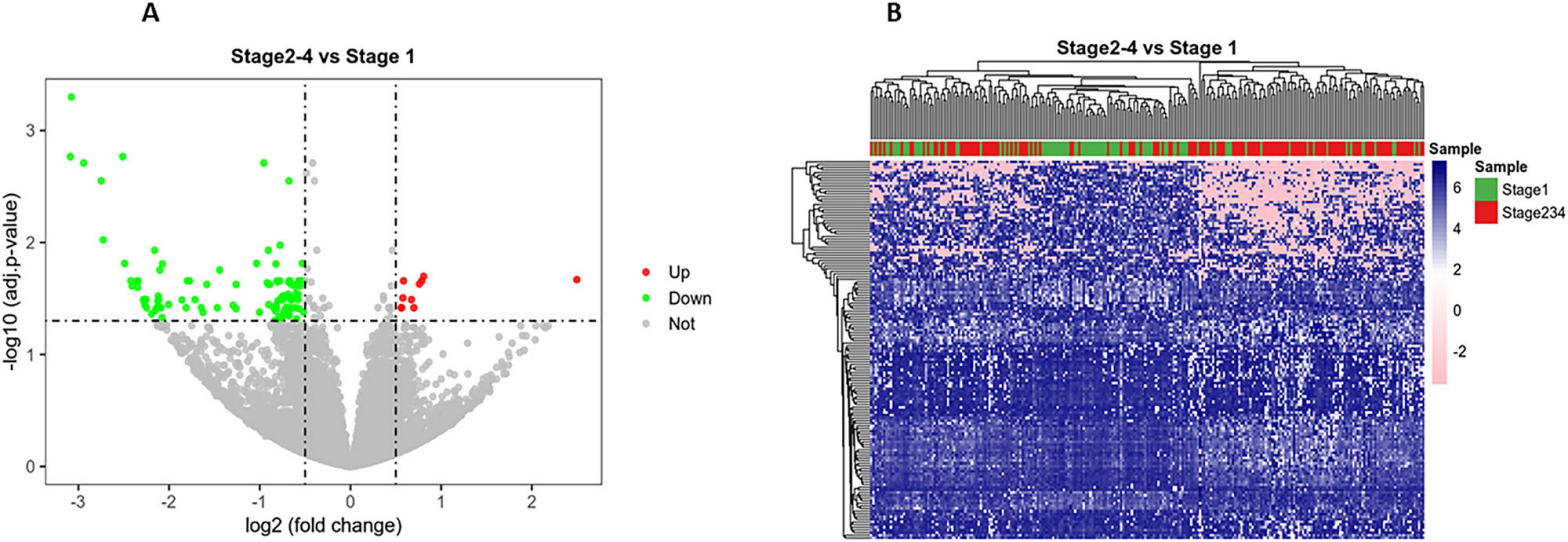
Transcriptomic differences between early- and late-stage tumors. **A** Volcano plot and **B** Heatmap showing transcripts significantly different between AJCC Stage 1 tumors and AJCC Stages 2–4 tumors.

Gene network analysis (Supplementary Table 4) identified endocrine system disorders, organismal injury and abnormalities, reproductive system disease, and developmental disorder, and others as the most significantly different networks. These tumors may represent a distinct “lumino-basal” phenotype and deserve further in-depth investigation. They were equally distributed between Black and White women. Among the 215 tumors included into the TNBCtype analysis, subtype distribution did not significantly differ between Black and White women (Table 1). However, 20/215 tumors were “unclassified” into the canonical subtypes. Of these, 14/20 occurred in Black women and 18/20 occurred in obese women. Seventy transcripts were differentially expressed in the unclassified versus classified tumors (Fig. 1C, D and Supplementary Table 5), including, among others, MYCN and immune-related transcripts such as CD3E and MICB (an NKGD2 ligand involved in NK and CD8 activation). IPA (Supplementary Table 6) identified chondroitin sulfate and dermatan sulfate degradation, telomere extension, and several immunologically relevant pathways that distinguished these tumors from classified TNBCs. Gene network analysis differences between unclassified and classified TNBCs are shown in Supplementary Table 7. As AJCC stage at diagnosis was strongly associated with survival (Supplementary Fig. 1) and Black women tended to be diagnosed at later stages, we compared GEPs of stage 1 tumors (confined to the breast and smaller than 2 cm in major diameter) to stages 2-4 tumors. One hundred forty-two transcripts were significantly different in stage 1 versus later stage tumors (Fig. 2A, B and Supplementary Table 8). Of 20 transcripts upregulated in later stage tumors, the most upregulated was LOC101928525, a lncRNA from a locus in chromosome 9q34.3 in the (-) strand of the MSRP2 gene, involved in mitochondrial metabolism^29^. Among the most significantly downregulated transcripts in later stage tumors are DEFB132 (a defensin involved in innate immunity), C14orf180 (an integral plasma membrane protein), TMEM252 (an integral plasma membrane protein), and LINC01537, an LncRNA associated with squamous carcinomas. None of the transcripts significantly up- or down-regulated in stage 1 versus later stage tumors was significantly different between Black and White women.

When we compared tumors that were fatal within 2 years of diagnosis to tumors with survival > 2 years, 20 transcripts were significantly different (Supplementary Fig. 2A, B and Supplementary Table 9). Among these, tumors with survival < 2 years showed decreased expression of TMEM64, a WNT negative regulator, and increased expression of EPN1 (Epsin-1), which promotes IKKgamma ubiquitination to drive NF-kB signaling in TNBC progression^30^.

Unsupervised cluster analysis identified 26 transcripts significantly different between self-reported Black and White women (Supplementary Fig. 3 and Supplementary Table 10). Pathway analysis (Supplementary Table 11) showed that xenobiotic metabolism and glutathione redox were the most significantly different pathways, alongside other breast cancer-related pathways such as WNT, embryonic stem cell pluripotency, mitochondrial dysfunction, and breast cancer regulation by stathmin. Gene network analysis (Supplementary Table 12) showed 5 significantly different networks, including cell death and survival, lipid metabolism/small molecule biochemistry, and cell-mediated immune response, consistent with *x*Cell results (see below). Three de-ubiquitinase genes in chromosome 8p23.1, USP17L1, USP17L4, and USP17L8, were differentially expressed between Black and White women. USP17L1 and USP17L4, which are contiguous to one another, were relatively over-expressed in Black women, while USP17L8, which is centromeric to USP17L1 and USP17L4, was relatively over-expressed in White women. Loss of heterozygosity in chromosome 8p, a region that includes genes controlling lipid metabolism^31^ is a marker of breast cancer progression^32^. Additionally, 8p23.1 chromosomal inversion is associated with poor prognosis in breast cancer^33^. FSHR, which was significantly over-expressed in White women, encodes the FSH receptor, a marker for vascular invasion and remodeling in breast cancer^34,35^. Twenty/26 transcripts, including USP17L1, USP17L4, USP17L8, and FSHR, remained significantly different when we limited our analysis to women older than 50 (104 Black and 105 White) (not shown). We observed no significant racial differences in GEPs in women younger than 50 (24 Black and 20 White), possibly due to the small sample size. Obesity did not account for these GEP differences. When we compared tumors from obese versus lean women, only 2 transcripts were significantly different (AP4E1 and KRT20, both significantly downregulated in tumors from obese women compared to lean ones). When we limited our analysis to obese women (101 Black and 75 White), 13 of the original 26 genes, including USP17L4, USP17L8, and FSHR, remained significantly different (not shown). Given the heavily admixed geographic ancestry of our cohort (see below) and the highly significant difference in ADI between the two arms, the hypothesis that these transcriptomic differences may be associated with life-long socioeconomic disadvantage deserves further investigation.

### Genetic ancestry analysis

Our cohort was genetically heavily admixed, including women with significant Mediterranean, North African, Native American, Pacific Islander and Asian components, consistent with the complex history of Louisiana (Supplementary Table 13 and Supplementary Fig. 4). Using an 80% threshold for predominant ancestry, only 59/118 (50%) Black women with evaluable DNA had >80% Central/West African ancestry, 71/111 (64%) of White women with evaluable DNA had >80% Northern/Western European ancestry, and 99/229 (43%) of the evaluable women in the sample set were multi-ancestral. Using a less stringent 60% threshold, 105/118 Black women (89%) had >60% Central/West African ancestry, 2/118 (1.7%) had >60% Western/Central European ancestry, and 12/118 (11%) were multi-ancestral. Among White women with evaluable DNA, 95/111 (86%) had >60% Northern/Western European ancestry, 2 (1.8%) had >60% Central/Western African ancestry, and 10 (9%) were multi-ancestral.

### Immune deconvolution

Next, we focused on tumor immune microenvironment-associated transcripts. CIBERSORTx^36^ results by race, ancestry, obesity, stage, and survival (Table 2) showed that memory B-cells were more common in Black, lean, and early-stage women, while activated mast cells were more common in Black women and women with at least 80% West African ancestry when compared to those with at least 80% European ancestry. Additionally, regulatory T cells were elevated among women with West African ancestry.

**Table 2:** CIBERSORTx results by race, ancestry, obesity status, age, stage, and 2-year survival

	Race		Ancestry^a^		Obesity		Age		Stage		2 y survival	
	*p* value	Elevated	*p* value	Elevated	*p* value	Elevated	*p* value	Elevated	*p* value	Elevated	*p* value	Elevated
B cells memory	**0.0031**	Black	0.1509		**0.0322**	Lean	0.9244		**0.0413**	Early	0.5060	
B cells naive	0.0681		0.1635		0.2488		**0.0320**	<50	0.0576		0.4228	
Dendritic cells activated	0.9231		0.2152		0.4525		0.6299		0.1054		0.3970	
Dendritic cells resting	0.0706		0.2995		0.2476		0.7184		0.4082		0.5434	
Eosinophils	0.3380		0.1729		0.0858		0.2766		0.1970		0.8076	
Macrophages M0	0.8085		0.9739		0.5085		0.4950		0.2555		0.4253	
Macrophages M1	0.5075		0.9944		0.0581		**0.0075**	<50	**0.0018**	Late	0.4577	
Macrophages M2	0.3017		0.4199		0.4786		**0.0264**	≥50	0.1121		0.9583	
Mast cells activated	**0.0073**	Black	**0.0059**	AA	0.1677		0.5380		0.1367		0.4456	
Mast cells resting	0.2458		**0.0230**	EA	0.7657		0.9259		**0.0006**	Early	0.8779	
Monocytes	0.2571		0.1656		0.6480		**0.0367**	≥50	**0.0343**	Early	0.5435	
Neutrophils	0.7167		0.4448		0.9084		0.2193		0.5069		0.6775	
NK cells activated	0.0684		0.1373		0.5227		0.2722		0.5473		0.2284	
NK cells resting	0.3471		0.7718		0.7192		0.8650		0.2832		0.5412	
Plasma cells	0.8600		0.9959		0.2419		0.2759		0.1169		0.6123	
T cells CD4 memory activated	0.8382		0.4780		0.6599		**0.0399**	<50	**0.0191**	Late	0.1802	
T cells CD4 memory resting	0.7807		0.4729		0.5843		0.8775		0.0577		0.1601	
T cells CD4 naive	0.5332		0.1529		0.1091		0.3989		0.8393		0.5176	
T cells CD8	0.2053		0.7610		0.5641		0.2489		0.2134		0.0788	
T cells follicular helper	0.9189		0.2367		0.1043		0.0877		**0.0166**	Late	0.5160	
T cells gamma delta	0.5699		0.1194		0.9128		0.4720		0.1463		0.7703	
T cells regulatory Tregs	0.2732		**0.0453**	AA	0.0774		0.7194		0.0742		0.6151	

*AA* African American, *EA* European American.

^a^Comparisons by ancestry included only patients found to have at least 80% West African ancestry (AA; *n* = 59) or at least 80% European ancestry (EA; *n* = 71) from genetic ancestry analysis.

Bold values indicate statistically significant differences.

Tumors from women <50 years of age contained more M1 macrophages and activated memory CD4 cells compared to older women, while tumors from older women contained more M2 macrophages and monocytes. Stage 1 tumors contained more memory B-cells, monocytes, and resting mast cells, while later stage tumors contained more M1 macrophages, activated memory CD4 cells, and follicular helper T-cells. CIBERSORTx analysis confirmed that the TNBC-type-excluded tumors clustered separately from the canonical subtypes, notably different for higher content in mast cells and lower content in M0 macrophages, while the TNBC-type-unclassified tumors were most similar to the IM subtype (Supplementary Fig. 5A and Supplementary Table 14). Several of the cell populations identified by CIBERSORTx were validated by IHC (Supplementary Fig. 5B). *x*Cell analysis^37^, which uses a different logic compared to CIBERSORTx and estimates 64 stromal populations, confirmed that B-cells were enriched in tumors from Black women and women with at least 80% West African ancestry, along with plasma cells, interdigitating dendritic cells (iDC), CD4 T-cells, CD4 central memory, CD4 memory and CD8 effector memory cells, while tumors from White women were relatively enriched in M2 macrophages, resting mast cells, monocytes, neutrophils, activated dendritic cells (aDC) and conventional dendritic cells (cDC) (Table 3). B-cells, CD4 memory T-cells, CD4 naïve T-cells, CD4 effector memory T-cells, CD8 central memory and naïve CD8 T-cells were also enriched in tumors with survival >2 years, along with Th1 and Treg T-cells and plasmacytoid dendritic cells (pDC). *x*Cell analysis also showed numerous significant differences among TNBC-type-excluded, TNBC-type-unclassified, and the canonical subtypes (Supplementary Table 15).

**Table 3:** xCELL results by race, ancestry, obesity, age, stage and 2-year survival

	Race		Ancestry^a^		Obesity		Age		Stage		2 y survival	
	*p* value	Elevated	*p* value	Elevated	*p* value	Elevated	*p* value	Elevated	*p* value	Elevated	*p* value	Elevated
Adipocytes	0.2944		0.1413		0.4932		0.2295		**<0.0001**	Early	0.4604	
Astrocytes	**0.0199**	White	0.4411		0.1901		0.4837		0.3548		0.4607	
B-cells	**0.0470**	Black	**0.0141**	AA	0.4631		0.0698		0.2114		**0.0113**	Yes
Basophils	0.2848		0.1967		0.5245		0.5450		0.0716		0.9762	
CD4+ T-cells	**0.0040**	Black	**0.0019**	AA	0.3517		0.4815		0.4123		0.0612	
CD4+ Tcm	**<0.0001**	Black	**0.0185**	AA	0.2913		0.9034		**0.0233**	Early	0.9322	
CD4+ Tem	0.9644		**0.0412**	AA	0.6215		0.1066		0.7809		**0.0344**	Yes
CD4+ memory T-cells	**0.0017**	Black	**0.0002**	AA	**0.0376**	Obese	**0.0092**	<50	**0.0008**	Late	**0.0057**	Yes
CD4+ naive T-cells	0.8369		0.2266		0.8972		0.5781		**0.0107**	Early	**0.0285**	Yes
CD8+ T-cells	0.7885		0.1954		0.6876		0.4301		0.7859		0.1527	
CD8+ Tcm	0.7135		0.2095		0.8898		**0.0303**	<50	0.2071		**0.0449**	Yes
CD8+ Tem	**0.0083**	Black	**0.0003**	AA	0.3565		0.5187		0.4441		0.0978	
CD8+ naive T-cells	0.0541		**0.0018**	AA	0.2407		**0.0317**	<50	**0.0275**	Late	**0.0331**	Yes
CLP	**<0.0001**	Black	**<0.0001**	AA	0.1199		**0.0440**	<50	**<0.0001**	Late	0.8666	
CMP	**<0.0001**	White	**0.0025**	EA	0.8484		0.8438		**0.0041**	Early	0.8647	
Chondrocytes	0.8127		0.7944		0.0918		**0.0287**	≥50	**0.0010**	Early	0.4749	
Class-switched memory B-cells	0.9881		0.3887		0.6055		0.2977		0.5554		0.0993	
DC	**<0.0001**	White	**0.0026**	EA	0.3368		0.3245		0.0947		0.7862	
Endothelial cells	**0.0008**	Black	0.0605		0.4200		0.9151		**0.0004**	Early	0.7182	
Eosinophils	0.6968		0.4271		0.9316		0.3393		**0.0150**	Early	0.2153	
Epithelial cells	0.6426		0.9683		0.9219		0.8677		**0.0039**	Late	**0.0281**	No
Erythrocytes	0.1516		0.3620		0.3486		0.5156		0.0899		0.5713	
Fibroblasts	**0.0222**	White	0.0593		0.8383		0.2714		**<0.0001**	Early	0.7460	
GMP	0.0785		0.3620		0.2501		0.4645		0.7907		0.3300	
HSC	0.2005		0.6704		0.4167		0.8900		**0.0002**	Early	0.1477	
Hepatocytes	0.8601		0.3031		0.2194		**0.0340**	≥50	**0.0004**	Early	0.0664	
Keratinocytes	0.2398		0.8534		0.4902		0.9071		**0.0464**	Late	**0.0402**	No
MEP	**<0.0001**	White	**<0.0001**	EA	0.0959		0.1911		0.0722		0.4020	
MPP	0.3116		1.0000		0.5083		0.6464		0.2313		0.6895	
MSC	**<0.0001**	White	**0.0001**	EA	**0.0296**	Lean	0.5876		0.3241		0.3087	
Macrophages	0.8365		0.1894		**0.0326**	Obese	0.1794		0.9353		0.1432	
Macrophages M1	0.3374		0.0786		**0.0279**	Obese	0.1127		0.1440		0.3737	
Macrophages M2	**0.0131**	White	0.1425		0.4077		0.0692		**0.0007**	Early	0.6102	
Mast cells	**<0.0001**	White	**0.0022**	EA	0.3807		0.1226		0.1327		0.9563	
Megakaryocytes	**0.0083**	White	**0.0019**	EA	0.2048		0.3152		**<0.0001**	Early	0.5062	
Melanocytes	0.2299		0.7729		0.2375		0.3820		0.8571		0.1712	
Memory B-cells	0.0567		**0.0120**	AA	0.5636		**0.0072**	<50	0.1995		0.0537	
Mesangial cells	**0.0294**	Black	0.1574		0.5270		0.3880		**0.0011**	Early	0.2529	
Monocytes	**0.0099**	White	0.1375		0.5815		0.3504		0.0780		0.2241	
Myocytes	**<0.0001**	White	**0.0007**	EA	0.9194		0.0655		0.1277		0.7261	
NK cells	0.0600		0.2257		0.3440		0.4158		0.1461		0.2846	
NKT	0.6836		0.2756		0.0785		0.8184		0.5267		0.3579	
Neurons	0.4778		0.9273		0.9925		0.0977		**0.0001**	Early	0.9166	
Neutrophils	**0.0166**	White	**0.0384**	EA	0.8660		0.1619		0.3884		0.0778	
Osteoblast	**0.0137**	Black	**0.0313**	AA	0.0657		0.2127		**0.0001**	Late	0.1221	
Pericytes	**0.0245**	White	**0.0106**	EA	0.0876		0.1335		**0.0205**	Early	0.3233	
Plasma cells	**0.0243**	Black	**0.0138**	AA	0.9289		**0.0389**	<50	**0.0002**	Late	0.1751	
Platelets	0.1791		0.0974		0.7472		0.3184		**0.0045**	Early	0.9650	
Preadipocytes	**0.0119**	White	**0.0040**	EA	0.4857		0.2695		**<0.0001**	Early	0.3783	
Sebocytes	**0.0016**	White	**0.0052**	EA	0.7384		0.2248		**0.0040**	Late	**0.0407**	No
Skeletal muscle	**<0.0001**	White	**0.0019**	EA	0.1965		0.5009		**0.0005**	Early	0.6522	
Smooth muscle	**<0.0001**	Black	**<0.0001**	AA	0.1461		0.0659		**0.0082**	Late	0.8181	
Tgd cells	0.2534		**0.0224**	AA	0.1657		0.0663		**<0.0001**	Late	**0.0212**	Yes
Th1 cells	0.3280		0.2352		0.2454		0.7777		**0.0006**	Late	0.1749	
Th2 cells	0.4527		0.8920		0.7792		**0.0228**	<50	**<0.0001**	Late	0.7094	
Tregs	0.5539		0.3807		0.5297		0.9401		0.4098		**0.0197**	Yes
aDC	**<0.0001**	White	**<0.0001**	EA	0.6348		0.4587		0.7703		0.1618	
cDC	**0.0149**	White	0.1393		0.5741		0.3728		**0.0093**	Early	0.6054	
iDC	**0.0132**	Black	**0.0421**	AA	0.1314		0.5650		0.0558		0.7121	
Lymphatic endothelial cells	**<0.0001**	Black	**0.0005**	AA	0.1130		0.8271		**0.0019**	Early	0.9669	
Microvascular endothelial cells	0.2791		0.7410		0.1789		0.7660		**0.0001**	Early	0.6743	
Naive B-cells	**<0.0001**	Black	**<0.0001**	AA	0.6532		**0.0167**	<50	0.3250		0.1743	
pDC	0.9174		**0.0276**	AA	0.2540		0.1303		0.0618		**0.0085**	Yes
pro B-cells	0.9686		0.7591		0.1663		0.6134		**0.0022**	Late	0.2921	
ImmuneScore	0.6750		0.1046		0.1025		0.1240		0.8415		0.0609	
StromaScore	0.8038		0.9851		0.8827		0.4835		**<0.0001**	Early	0.5676	
MicroenvironmentScore	0.8663		0.6037		0.2927		0.6188		**0.0008**	Early	0.1116	

*AA* African American, *EA* European American.

^a^Comparisons by ancestry included only patients found to have at least 80% West African ancestry (AA; *n* = 59) or at least 80% European ancestry (EA; *n* = 71) from genetic ancestry analysis.

Bold values indicate statistically significant differences.

The most consistent immune microenvironmental difference identified by both algorithms was higher B-cell lineage infiltration in Black women. Tumor-infiltrating B-cells (B-TIL) are a favorable prognostic indicator in breast cancer^38^. Tumor-infiltrating B- and T-cells are a positive prognostic indicator in TNBC^39^.

### Survival analysis

Supplementary Fig. 1 shows a highly significant association between stage at diagnosis and survival in our sample, consistent with our previous observations^17^. Due to the magnitude of the effect of stage on survival, additional associations with survival were performed via Cox proportional hazard models, controlling for stage as a fixed covariate. Results are reported as Supplementary Table 16. Race was not significantly associated with survival (*p* = 0.094), but obese I and II cases had significantly greater survival compared to lean cases (*p* < 0.05). Additionally, there were significant differences in survival by TNBCtype, with Immuno-modulatory having the greatest rates of survival (*p* < 0.05), consistent with the literature^40,41^. The naïve B cell population in the tumor microenvironment (CIBERSORT) was associated with greater survival (*p* < 0.05) but not the memory B cell population (*p* > 0.05).

## Discussion

We compared GEPs in TNBC from Black and White women with TNBC from Louisiana. The study arms differed significantly in BMI, obesity, and area deprivation index (ADI). The limited GEP differences we observed between TNBCs from Black and White women were not linked to stage, survival, or obesity. The relative roles of socioeconomic exposures and ancestry admixture in the GEP differences we observed remain to be determined. In our sample set, race was not associated with differences in survival, while obesity (as determined by BMI) was associated with longer survival. AJCC stage was a strong predictor of survival. The relationship between BMI and TNBC survival is poorly understood, with some studies showing no association^42,43^ and others an association with poor prognosis^44,45^. However, an “obesity paradox” has been proposed for TNBC, whereby obesity may promote response to immunotherapy by modulating the immune microenvironment^46^. In our sample, CD4 memory T-cells (Tcm) were associated with Black race, African ancestry, obesity, late stage and survival over 2 years. The relationship between the immune microenvironment, BMI, race and ancestry deserves further investigation.

Our analysis revealed a subset of 38 immunohistochemically confirmed TNBCs (<1% ER and PR expression) that were excluded from TNBC-type analysis and had vastly different transcriptomic features from TNBCs-included tumors. These tumors appear to have a “lumino-basal” transcriptomic signature, with estrogen signaling and Notch activity signatures. Haughian et al.^47^ showed that over 50% of primary ER(+)PR(+) breast cancers contain an ER(-)PR(-)CK5(+) “lumino-basal” subpopulation, which is driven by Notch signaling and shows phenotype and GEP matching those of TNBC. Whether this subset of tumors originate from initially ER(+) pre-neoplastic lesions through phenotypic plasticity and/or loss of ER expression in luminal progenitor cells, and whether these tumors are sensitive to Notch inhibition, remains to be established.

The molecular profiles of tumors varied significantly with AJCC stage irrespective of race, indicating that tumor progression is associated with remarkable transcriptomic and immunological changes. Rapidly fatal (<2 year) tumors also had distinctive molecular profiles irrespective of race.

We observed immunogenomic diversity based on race, ancestry, age, stage, obesity, and survival. Our *x*Cell results (Table 3) are generally consistent with those of Martini et al.^21^, who showed that several immune populations, including B-cells were more abundant in TNBC from women of West African ancestry. Our results confirm these findings and indicate an association between B-cell infiltration and improved survival. Intratumoral B-cells promote optimal anti-tumor T-cell responses and are thought to contribute to the efficacy of immunotherapy^48^. The enrichment in cDC and aDC in tumors from White women suggests that immunotherapies harnessing DCs^49^ may be of interest in this group of patients. In our heavily admixed cohort, the possible roles of different ancestries and their interactions with the exposome in modulating gene expression will require additional studies. Our cohort also differs from that studied by Martini et al.^21^ in that our women were from a single US state (Louisiana), and the two arms of the study were demographically and clinico-pathologically matched except for BMI and ADI.

Limitations of our study include the exclusion of overweight patients to maximize statistical power to detect differences between lean and obese patients, the potential for selection bias, and the use of retrospective registry data. Our sample size did not permit an analysis of different degrees of obesity (I, II, or III). Supplementary Table 17 shows the demographic distribution of our cases across the obesity spectrum. Importantly, BMI does not necessarily provide information on the metabolic status of patients^50^. Future studies should evaluate the relationship between metabolic health and TNBC prognosis.

Taken together, our findings suggest that while biological/immunological differences with potential precision oncology implications may exist between TNBCs occurring in women who self-identify as either Black or White, these self-identified classifiers do not necessarily predict poorer survival outcomes in Black women—which are more likely to be due to access to adequate care and/or comorbidities.

Our findings highlight the need for a refined classification of TNBCs that considers socioeconomic factors, genetic admixture, stage, the phenotypic plasticity of tumor cells, and the heterogeneity of the tumor microenvironment, to better predict clinical outcomes.

## Methods

### Study design and data collection

We collaborated with the Louisiana Tumor Registry (LTR) and the SEER-Linked virtual tumor repository program to obtain de-identified formalin-fixed, paraffin-embedded (FFPE) tissue and clinical data for TNBC cases in Louisiana. LTR acquired and de-identified FFPE tumor blocks under a protocol deemed “exempt” by the LSUHSC IRB. The target population was TNBC cases diagnosed from 2010 to 2016 (Supplemental Table 1). Clinico-pathological parameters collected included: Age at diagnosis, race (self-reported), family history of breast cancer (if known), height, weight, diagnosis of diabetes (Y/N, if known), histological type, histological grade, of ER, PR, Her2/Neu, Ki67 (if available) and any other immunohistochemistry markers studied (e.g., CK5/6, c-Kit), AJCC stage at diagnosis, and recurrence (Y/N). Body mass index (BMI) (kg/m^2^) around the time of diagnosis was calculated from height and weight. BMI was classified as lean (18.5 ≤ BMI < 25), obese class I (30 ≤ BMI < 35), obese class II (35 ≤ BMI < 40), and obese class III (BMI ≥ 40).

Inclusion criteria were: Female breast cancer; ER, PR negative (<1% by immunohistochemistry), absence of HER2-amplification by immunohistochemistry (IHC) and/or fluorescence in situ hybridization (FISH); Age at diagnosis ≥ 18; Race: Black or White; Any AJCC stage (0, I II, III, IV); BMI lean (BMI < 25) or obese (BMI > 30); Documented surgery (lumpectomy or any partial or total mastectomy); documented NACT (Y/N). We excluded underweight (BMI < 18.5) and overweight (25 ≤ BMI < 30) women to maximize statistical power to detect differences between lean and obese women. These criteria identified 1123 cases (Supplemental Table 2).

The study design included two races (Black, White) and 4 obesity categories (lean, obese I, obese II, obese III). From the sampling frame, tissues were requested from 3 statewide pathology laboratories to establish a stratified random sampling of each race and obesity category, targeting up to 50 cases per combination. From these tissue requests, we obtained tissue from 256 cases. Three cases were censored. Two were women with bilateral tumors, and one was found to be incorrectly identified as TNBC. The final sample included 253 TNBC cases. Area Deprivation Index (ADI)^51^ was calculated from census tract data available for 251 cases.

### Immunohistochemistry

Sections of FFPE tissue containing ≥ 50% tumor, as judged by the study pathologist, were cut and processed for immunohistochemistry and RNA extraction. Formalin-fixed, paraffin-embedded tissues were microtome-sectioned to a thickness of 4 μm, placed on electromagnetically charged slides (Fisher Scientific; Waltham, MA), and stained with Hematoxylin & Eosin (H&E) for routine histologic analysis. Immunohistochemistry was performed using Avidin-Biotin-Peroxidase, according to the manufacturer’s instructions (Vectastain Elite ABC Peroxidase Kit; Vector Laboratories, Burlingame, CA). Our modified protocol includes melting the paraffin at 56 °C for 15 min, clearing in xylenes 3 times for 15 min each, rehydration through descending grades of alcohol up to water, non-enzymatic antigen retrieval with 0.01 M sodium citrate buffer pH 6.0 at 95 °C for 25 minutes in a vacuum oven, and endogenous peroxidase quenching with 3% H_2_O_2_ in methanol, Following these steps, slides were washed with PBS and blocked in PBS/0.1% BSA containing 5% normal horse serum (for mouse monoclonal antibodies), or normal goat serum (for rabbit generated antibodies) for 2 h at room temperature, then incubated overnight with primary antibodies. Primary antibodies included rabbit recombinant anti-CD-3 (clone SP162, 1:150 dilution, Abcam, Fremont, CA), anti-CD-8 α (clone EPR20305, 1:200 dilution, Abcam), and an anti-FoxP3 (clone EPR15038-69, 1:50 dilution, Abcam), and mouse monoclonal anti-IL-17A (clone 4K5F6, 1:20 dilution, Abcam) and anti-CD20cy (clone L26, 1:100 dilution, DAKO, Santa Clara, CA). Breast Cancer specific biomarkers were tested with rabbit recombinant anti-estrogen Receptor alpha (clone EPR4097, 1:250 dilution, Abcam), anti-Progesterone Receptor (clone YR85, 1:250 dilution, Abcam), and anti-ErbB2/HER2 (clone SP3, 1:100 dilution, Abcam). The following day, slides were incubated with biotinylated secondary anti-rabbit or anti-mouse antibodies for 1 h at room temperature, developed using a diaminobenzidine substrate, counterstained with hematoxylin, and mounted with Permount. Images were collected at 200x and 600x magnification using a BX61 Olympus microscope equipped with a high-resolution Olympus DP74 digital camera and CellSense image capture software.

### RNA extraction and library preparation

Total RNA was extracted from FFPE tissue samples using the truXTRAC FFPE total NA (column) kit (Covaris). Directions from the manufacturer were followed with a few modifications. Tissue sections of 20 μm were first deparaffinized using Sigma’s xylene substitute, followed by two ethanol washes and air dried for 20 min at 37 °C. After deparaffinization, tissues were lysed in lysis buffer containing proteinase K using the Covaris M220 focused ultrasonicator and following the recommended settings by the manufacturer, followed by an incubation at 56 °C for 30 min. RNA-containing supernatant was then de-crosslinked at 80 °C for 20 min and purified using the RNA purification columns as indicated by the manufacturer’s instructions. A treatment with DNase I (Qiagen) was included. RNA was eluted in 30 μl of H_2_O. RNA quantification was performed using the Qubit RNA HS Assay kit (Invitrogen) and RNA quality was assessed with the Agilent 2100 bioanalyzer (Agilent Technologies).

Libraries were generated using Illumina’s TruSeq RNA exome library preparation kit and according to manufacturer’s instructions, with the following modifications^52^: 300 ng of input RNA was used, PCR cycles were reduced to 9 cycles, and hybridization time was extended to 16 h. After library preparation, free adapters were blocked using Illumina’s free adapter blocking reagent. Libraries were validated on Agilent’s 2100 bioanalyzer (Agilent Technologies) using a DNA 1000 or HS DNA Kit and quantified using the Qubit dsDNA HS Assay kit (Invitrogen). For sequencing, libraries were pooled in equimolar ratios and run on Illumina’s NextSeq 500 instrument using a NextSeq 500 high-output v2 kit with pair-end 75 bp reads.

### RNA sequencing

The FASTQ files were obtained from Illumina’s BaseSpace and were uploaded to Partek Flow. Contaminants were removed with Bowtie 2.2.5, aligned to STAR 2.6.1 d using hg38 as reference, and quantified with RefSeq 96 (release 2020-11-02). Raw counts were downloaded for differential expression analysis. For cell deconvolution and enrichment, the CIBERSORTx^36^ and *x*Cell^37^ algorithms were used.

### Bioinformatics analysis

Data consisting of raw sequence reads generated using RNA-Sequencing of 258 samples was transferred from the Translational Genomics Core to the Center for Bioinformatics Services. Gene expression data were quantified using Transcripts Per Kilobase Million (TPM) and were log2 (TPM+1) transformed. Patients with bilateral tumors were removed to avoid potential confounding of the results by hereditary cancer syndromes. After data preprocessing, we generated a dataset consisting of 253 samples and 28,278 genes. Gene expression data was processed and normalized using loess normalization method implemented using Bioconductor R-package LIMMA^53^. The gene expression data matrix was filtered to remove transcripts with missing data and very low expression values, as the values were noise and not informative. After data preprocessing, we generated a dataset containing 19,361 genes consisting of 128 samples from self-identified Black/African American (AA) women and 125 samples from self-identified White/European American (EA) women, which were used in the analysis. The probe IDs, gene symbols, and names were matched for interpretation using the Ensemble database, a database used for gene annotation of RNA-sequencing experiments. Using normalized data, we performed whole transcriptome analysis comparing gene expression levels between Black and White women using a paired t-test implemented in the R-package LIMMA^53^, to discover a signature of significantly differentially expressed genes distinguishing Black from White women. In addition, we performed differential expression by various variables and self-reported race. We compared gene expression at age ≥ 50, Black vs White, obese versus non-obese overall and Black vs White, stage 2-4 vs stage1, stage 2-4 Black vs White, and mortality >2 year vs ≤2 year. In addition, we performed differential expression analysis comparing 215 TNBC-type “included” versus 38 “TNBC-type “excluded” tumors, and 195 TNBC-type- included “classified” samples vs 20 TNBC-type-included “unclassified” samples. Throughout these analyses, we used the false discovery rate (FDR) procedure^54^ to adjust p-values for multiple hypothesis testing. Additionally, we computed the log2 Fold Change (Log2FC) defined as the median of tumors minus median of normal for each gene. Genes were ranked based on adjusted *p*-values, FDR, and LogFC. We performed unsupervised analysis using hierarchical clustering using the Pearson correlation coefficient as the distance measure between pairs of genes to characterize the patterns of expression profiles of identified sets of genes.

We performed network and pathway analysis using the Ingenuity Pathway Analysis (IPA) software package^55^. Using IPA, we mapped the set of significantly differentially expressed genes distinguishing comparison groups. We computed probability Z-scores and log *p* values to assess the likelihood and reliability of correctly assigning the genes to the correct molecular networks and signaling pathways, respectively. FDR was used to correct for multiple hypothesis testing in pathway analysis. The predicted molecular networks and biological pathways were ranked based on z-scores and log *P* values, respectively. Gene ontology (GO)^56^ analysis, as implemented in IPA, was used to classify the genes according to the molecular functions, biological process, and cellular components in which they are involved.

### Genetic ancestry analysis

DNA samples were first checked for quality using the Illumina Infinium HD FFPE QC Kit, following vendor recommendations. All evaluable samples with sufficient DNA passed the QC test, and 200 ng of DNA were used to interrogate 1.8 million SNPs using the Illumina Global Diversity Array (GDA). Briefly, samples were amplified at 37 °C for 24 h, fragmented at 37 °C for 1 h, precipitated, and resuspended in buffer. Samples were hybridized to beadchips for 18 h at 48 °C. The beadchips were washed to remove unhybridized and nonspecifically hybridized DNA, and samples were subjected to a single-base extension at 44 °C and staining at 32 °C. The beadchips were washed and scanned in the Illumina iScan system. The IDAT files (green and red fluorescence) were downloaded and used for allele calling.

Global ancestry was inferred using a total of 163 ancestry-informative single nucleotide polymorphisms (SNPs) (AISNP) published by Pakstis et al.^57^. From the Illumina Genome Diversity Array, we extracted the plus-string genotypes of the 163 AISNPs for the 233 samples. Two SNPs with over 90% missing genotype were excluded. Genetic ancestry proportion was calculated using the STRUCTURE software 2.3.4^58^. under the admixture model assuming correlated allele frequencies. Based on the genotypes of 161 AISNPs, we first determined the optimal number of clusters that best fits the 1296 reference samples from 22 diverse human populations^57^ by running the program 20 times at each K level from *K* = 7 to *K* = 14 with 10,000 burn-in and 10,000 Markov Chain Monte Carlo (MCMC) iterations. Based on the optimum *k*-value of 11, we ran unsupervised structure analysis on the combined data from 1296 reference and 321 test samples. Because one of the 11 clusters was specific to the TNBC test samples, we also conducted supervised structure analysis as follows: 672 reference samples were selected from 8 predefined ethic groups where the majority of samples in each predefined group had high ancestry proportion (>0.9) in one of 11 clusters in the initial unsupervised analysis. The STRUCTURE program then grouped the test samples based on the pre-defined population-of-origin. In order to examine reliability of ancestry inference in the current supervised analysis, we included an additional 88 test samples with results generated using unsupervised Structure analysis in order to assess variation between the supervised and unsupervised analyses.

The overall correlation between results from the supervised and unsupervised was very high (0.68 to 0.97 across 8 different clusters), and concordant with self-reported race and ethnicity in the additional 88 test samples. Therefore, we are confident that the ancestry proportions for these 233 test samples are appropriate. In addition, we used principal components analysis, calculated using PLINK 1.946^59^, as a complementary method to assess ancestry.

### Statistical analysis

Group comparisons of age at diagnosis were assessed using ANOVA. Categorical variables were compared using Fisher’s exact tests. Overall survival was visualized via Kaplan Meier plots and compared using log-rank tests and Cox proportional hazards models. Cell population data (CIBERSORTx^36^, *x*Cell^37^) were visually summarized using heatmaps of the median values, and compared using non-parametric Kruskal–Wallis tests.

## Supplementary information

Supplementary Information

## Data Availability

RNA Sequence data that supports the findings from this study of 253 TNBC cases analyzed by RNAseq has been deposited to the NCBI Gene Expression Omnibus (GEO) https://www.ncbi.nlm.nih.gov/geo/ with the accession number GSE268851. The data will be released to the public upon publication.

## Code availability

All code used for our analyses is publicly available as described in the Methods section. No new code was developed for this manuscript.
