# Supplementary material for: Immunogenomic diversity of triple-negative breast cancers in obese and non-obese black and white women
Source: NPJ Breast Cancer. 2025 Nov 12;11:126. doi: 10.1038/s41523-025-00836-6 (PMC12612227; doi:10.1038/s41523-025-00836-6)
Supplement: Supplementary file 1 — Supplementary Information [file 41523_2025_836_MOESM1_ESM.pdf]

Supplementary Table 1: Study population

Number of Triple Negative Breast Cancer Cases by Louisiana Region, Diagnosis Year and Race					
		White	Black	Other	All
	Diagnosis Year				
North	2010	61	52	.	113
	2011	48	47	.	95
	2012	51	48	.	99
	2013	52	57	1	110
	2014	45	72	.	117
	2015	64	64	.	128
	2016	64	64	.	128
	All	385	404	1	790
South	Diagnosis Year	168	167	5	340
	2010				
	2011	191	153	3	347
	2012	155	147	.	302
	2013	171	164	3	338
	2014	177	172	4	353
	2015	191	172	5	368
	2016	164	183	3	350
	All	1,217	1,158	23	2,398
Total		**1,602**	**1,562**	**24**	**3,188**

Supplementary Table 2: Differentially expressed transcripts in tumors included versus excluded in TNBCtype analysis

Symbol	Chromosome	logFC	adj.P.Val	TNBCtype-included regulated
IBSP	4q22.1	5.395371441	2.23E-08	UP
CCL7	17q12	5.100703427	2.23E-08	UP
NT5DC4	2q14.1	5.039727765	2.30E-08	UP
LHX2	9q33.3	4.486542638	1.40E-06	UP
HMMR-AS1	5q34	4.239555598	7.38E-06	UP
WT1	11p13	4.16973237	0.002829162	UP
CLEC6A	12p13.31	4.122007941	9.01E-06	UP
DMP1	4q22.1, 7q21.12	4.117174247	0.000265203	UP
CDKN2A-DT	9p21.3	4.061617363	7.34E-06	UP
ACTL8	1p36.13	4.055699268	1.45E-05	UP
LINC00634	22q13.2	3.992135988	1.83E-05	UP
OR2B6	6p22.1	3.968074202	0.000299686	UP
LINC00518	6p24.3	3.936107749	2.06E-05	UP
PTPRN	2q35	3.922007137	0.000534173	UP
RAET1K	6q25.1	3.887013631	2.40E-05	UP
PRSS41	16p13.3	3.885678581	1.08E-05	UP
NMU	4q12	3.84821347	0.000120763	UP
ZNF670-ZNF695	1q44	3.840161646	3.07E-05	UP
WT1-AS	11p13	3.705536801	0.00339723	UP
SNORA22B	7p11.2	3.686982206	0.000298799	UP
NKX3-2	4p15.33	3.6723166	0.000200131	UP
TCL1A	14q32.13	3.634645986	0.000355361	UP
IGF2BP1	17q21.32	3.628952443	0.000217568	UP
MCEMP1	19p13.2	3.609243152	0.000200202	UP

**POU4F1**	13q31.1	3.606288356	0.000266631	UP
**FCRL4**	1q23.1	3.596436728	6.15E-05	UP
**INA**	10q24.33	3.484866979	0.000287478	UP
**TMEM52B**	12p13.2	3.463669337	0.000798141	UP
**SERPINB7**	18q21.33	3.460031581	0.001502834	UP
**GPR31**	6q27	3.456695559	0.000131356	UP
**DAZL**	3p24.3	3.437014108	0.000711757	UP
**LOC101929427**		3.42127784	0.000487722	UP
**LINC01096**	4p15.33	3.406354156	0.000215741	UP
**TLX3**	5q35.1	3.392619214	0.000172882	UP
**IGFBPL1**	9p13.1	3.381216098	0.00063451	UP
**LHX8**	1p31.1	3.379615953	0.000492294	UP
**HOXB9**	17q21.32	3.371186326	0.000614008	UP
**TAFA4**	3p14.1	3.322875946	0.000193245	UP
**PRDM13**	6q16.2	3.303331997	0.000200386	UP
**FGF5**	4q21.21	3.279381907	0.000697621	UP
**MAGEB4**	Xp21.2	3.207266031	0.000417537	UP
**OTOG**	11p15.1	3.190434902	0.001168757	UP
**OR2B2**	6p22.1	3.187178704	0.006621378	UP
**ULBP1**	6q25.1	3.144189503	0.002048486	UP
**DNAH17-AS1**	17q25.3	3.12368905	0.00197172	UP
**TLX1**	10q24.31	3.116048589	0.001539663	UP
**CXorf49B**	Xq13.1	3.103831471	0.001710252	UP
**METTL11B**	1q24.2	3.096761148	0.000645782	UP
**HOXB13**	17q21.32	3.096143907	0.002175095	UP
**MEIG1**	10p13	3.078750129	0.001937572	UP
**TRIM15**	6p22.1	3.071957886	0.000644179	UP
**IL21**	4q27	3.070534211	0.001024929	UP

**HLA-DPB2**	6p21.32	3.055828992	0.002854089	UP
**C11orf86**	11q13.2	3.053921122	0.001231919	UP
**CARD17**	11q22.3	3.024295509	0.003152605	UP
**HTR1D**	1p36.12	3.017322108	0.002975145	UP
**CENPA**	2p23.3	3.005750086	0.002534368	UP
**MYLK2**	20q11.21	2.998155658	0.005625969	UP
**NOTUM**	17q25.3	2.991704736	0.003758163	UP
**KISS1**	1q32.1	2.988776745	0.002170617	UP
**PATE1**	11q24.2	2.984673509	0.001533749	UP
**DMRT1**	9p24.3	2.968472329	0.002398016	UP
**PIMREG**	17p13.2	2.966989972	0.01553097	UP
**CST1**	20p11.21	2.960141914	0.003871572	UP
**LUZP4**	Xq23	2.927402236	0.002488563	UP
**MAGEB1**	Xp21.2	2.887803218	0.00080308	UP
**ADAMTS20**	12q12	2.88078499	0.004276511	UP
**HOXC12**	12q13.13	2.871537066	0.004764157	UP
**SNORA30**	16p11.2	2.869758252	0.012225134	UP
**CLPS**	6p21.31	2.858844359	0.00541584	UP
**F13B**	1q31.3	2.858292584	0.002207683	UP
**ZNF280A**	22q11.22	2.856691726	0.00433331	UP
**STRA8**	7q33	2.84645004	0.005874636	UP
**MMP1**	11q22.2	2.843439892	0.010418863	UP
**KLF1**	19p13.13	2.839523854	0.005455162	UP
**SPIC**	12q23.2	2.833224926	0.007164983	UP
**C4orf54**	4q23	2.832787297	0.005424074	UP
**NPHS1**	19q13.12	2.817028223	0.004870236	UP
**FAM216B**	13q14.11	2.802974413	0.02614963	UP
**NKX1-2**	10q26.13	2.783331764	0.008196566	UP

**PCAT7**	9q22.32	2.780811773	0.004193795	UP
**CSAG3**	Xq28	2.777514187	0.007682651	UP
**TMEM89**	3p21.31	2.771236399	0.007419574	UP
**IFNL3**	19q13.2	2.766903133	0.001096771	UP
**SLC5A5**	19p13.11	2.766036768	0.008762444	UP
**SMIM43**	4q27	2.753642217	0.005467163	UP
**H1-6**	6p22.2	2.747076325	0.008912724	UP
**MMP8**	11q22.2	2.737626867	0.008912724	UP
**SHD**	19p13.3	2.731712925	0.00894212	UP
**KCNV1**	8q23.2	2.713802031	0.002465082	UP
**LCT-AS1**	2q21.3	2.684445093	0.009974545	UP
**LIX1**	5q15	2.669435971	0.013251266	UP
**ACP7**	19q13.2	2.667876155	0.010588062	UP
**LINC00461**	5q14.3	2.665402502	0.007423346	UP
**DLX4**	17q21.33	2.652440771	0.013309346	UP
**IL31**	12q24.31	2.65221655	0.003577995	UP
**GJB6**	13q12.11	2.640859724	0.013118368	UP
**CAMKV**	3p21.31	2.639079299	0.011122251	UP
**MAFA**	12p13.31, 8q24.3	2.638245893	0.004620223	UP
**LY6D**	8q24.3	2.632980993	0.013309346	UP
**TLX2**	2p13.1	2.624133743	0.011279168	UP
**CSAG1**	Xq28	2.613259566	0.005060021	UP
**ZP4**	1q43	2.612681467	0.001463898	UP
**MAGEA2**	Xq28	2.611138185	0.00618094	UP
**APOC4-APOC2**	19q13.32	2.609463317	0.017798591	UP
**GTSF1L**	20q13.12	2.607457906	0.003059312	UP
**SH2D7**	15q25.1	2.60627902	0.012842873	UP
**LOC149684**		2.604907401	0.011579243	UP

**CCDC166**	8q24.3	2.593662157	0.013309346	UP
**IL12B**	5q33.3	2.592306942	0.015338142	UP
**RDM1**	17q12	2.584098319	0.034456811	UP
**LIN28B**	6q16.3-q21	2.557650715	0.008178444	UP
**AMTN**	4q13.3	2.557439324	0.012484264	UP
**FOXD3**	1p31.3	2.557019501	0.016145238	UP
**MIR3142HG**	5q33.3	2.554199421	0.012842873	UP
**HOTAIR**	12q13.13	2.542529629	0.004479859	UP
**HMSD**	18q22.1	2.541197972	0.018969046	UP
**SNORD5**	11q21	2.531956631	0.007406684	UP
**C1orf94**	1p35.1	2.531554654	0.011224551	UP
**FOXG1**	14q12	2.521479667	0.01662376	UP
**TAFA3**	1p13.2	2.506170745	0.020135852	UP
**GABRQ**	Xq28	2.496526383	0.018769801	UP
**TEX11**	Xp11	2.484618712	0.017538062	UP
**CLLU1-AS1**	12q22	2.477389119	0.015057817	UP
**AVPR1B**	1q32.1	2.476348017	0.011453649	UP
**TCF24**	8q13.1	2.454805154	0.020090199	UP
**PDCL2**	4q12	2.451288611	0.002357163	UP
**LINC00673**	17q24.3	2.450249117	0.008881594	UP
**LINC01276**	6p21.1	2.447723125	0.018272382	UP
**KRT75**	12q13.13	2.439218654	0.027359596	UP
**RAET1L**	6q25.1	2.421923682	0.027091707	UP
**AKR1C4**	10p15.1	2.416144332	0.022686392	UP
**KRT2**	12q13.13	2.412846594	0.023592189	UP
**PRR27**	4q13.3	2.409623514	0.011451635	UP
**DLL3**	19q13.2	2.404557567	0.028481788	UP
**LRRC37A8P**	17q12	2.399700471	0.031180895	UP

**CCL20**	2q36.3	2.394337058	0.021664426	UP
**PDX1**	11p13, 13q12.2	2.381541029	0.00294708	UP
**RBM14-RBM4**	11q13.2	2.380872178	0.030249735	UP
**GRM4**	6p21.31	2.378010854	0.027498791	UP
**TRIML2**	4q35.2	2.377207235	0.026531864	UP
**TSACC**	1q22	2.373374019	0.031378033	UP
**TMEFF1**	9q31.1	2.367115047	0.042437541	UP
**FABP2**	4q26	2.359489317	0.02408582	UP
**LY6H**	8q24.3	2.355380454	0.031940926	UP
**SNORA10B**	2p23.1	2.351617969	0.037519772	UP
**C17orf99**	17q25.3	2.348763991	0.032084365	UP
**SNORD36B**	9q34.2	2.331854669	0.010774843	UP
**RNASE9**	14q11.2	2.322540494	0.003890118	UP
**GABRA5**	15q12	2.31714003	0.028532352	UP
**LMO1**	11p15.4	2.315255542	0.032889128	UP
**TONSL-AS1**	8q24.3	2.309621563	0.036110162	UP
**PPP1R2P2**	21q22.12	2.292403451	0.032727382	UP
**C2orf91**	2p21	2.286130367	0.0325566	UP
**SYCN**	19q13.2	2.276228053	0.030584912	UP
**SLC1A6**	19p13.12	2.275094501	0.031198876	UP
**IL36RN**	2q14.1	2.273101527	0.019047147	UP
**FABP6**	5q33.3	2.268539666	0.028273073	UP
**CHRNA6**	8p11.21	2.268488112	0.032239426	UP
**H2BS1**	21q22.3	2.265450758	0.048441926	UP
**EPYC**	12q21.33	2.246992724	0.034325608	UP
**CASP14**	19p13.12	2.243986431	0.040922073	UP
**TTBK1**	6p21.1	2.23682212	0.044992034	UP
**NPBWR2**	20q13.33	2.229545147	0.012200397	UP

**IVL**	1q21.3	2.220916708	0.041139825	UP
**FAM163A**	1q25.2	2.213237936	0.004813844	UP
**LOC100130331**		2.206607214	0.046908361	UP
**RXFP3**	5p13.2	2.205097501	0.02309692	UP
**DPYSL5**	2p23.3	2.200427035	0.039018575	UP
**OR1F1**	16p13.3	2.195373821	0.030122272	UP
**VIM-AS1**	10p13	2.194170343	0.019879065	UP
**RNASE10**	14q11.2	2.18372525	0.034710383	UP
**DPPA4**	3q13.13	2.183362732	0.042875632	UP
**KRT85**	12q13.13	2.175867018	0.035342773	UP
**ALPP**	2q37.1	2.161967469	0.011122251	UP
**TMEM270**	7q11.23	2.16173809	0.02377625	UP
**LINC02605**	8q21.13	2.159783868	0.010099752	UP
**MAGEA2B**	Xq28	2.150538247	0.028254844	UP
**LINC01614**	2q35	2.141697389	0.036141197	UP
**GJA3**	13q12.11	2.139899741	0.031006296	UP
**GHRHR**	7p14.3	2.133786908	0.022316019	UP
**KASH5**	19q13.33	2.128360512	0.033108537	UP
**LINC01561**	10q26.12	2.106300534	0.021740377	UP
**NPW**	16p13.3	2.09633556	0.030377609	UP
**CYTOR**	2p11.2	2.057777909	0.036470509	UP
**NKX2-2**	20p11.22	2.045470164	0.031163315	UP
**XAGE2**	Xp11.22	2.009408066	0.030785668	UP
**FOXCUT**	6p25.3	1.991577832	0.016747045	UP
**BCAR4**	16p13.13	1.989569629	0.037587827	UP
**EFNA2**	19p13.3	1.984960703	0.037908924	UP
**CHRNA1**	2q31.1	1.975591286	0.048613256	UP
**SLC18A1**	8p21.3	1.934082811	0.034028461	UP

**TMEM67**	8q22.1	1.897459833	0.003479443	UP
**NKX2-3**	10q24.2	1.834083337	0.038812093	UP
**TCAM1P**	17q23.3	1.820708394	0.019639175	UP
**NRDE2**	14q32.11	1.80130683	0.010648749	UP
**DMRTA2**	1p32.3	1.798738377	0.035402215	UP
**MIGA1**	1p31.1	1.771987599	0.034387267	UP
**CACNG5**	17q24.2	1.729108178	0.030798123	UP
**C7orf77**	7q31.33	1.709264557	0.027417354	UP
**TECPR2**	14q32.33	1.674266914	0.000329867	UP
**SH3GLB2**	9q34.11	1.665595545	0.000584639	UP
**TBC1D28**	17p11.2	1.615837929	0.031879814	UP
**C6orf201**	6p25.2	1.606847697	0.013965375	UP
**AAAS**	12q13.13	1.605592672	0.000165879	UP
**FANK1**	10q26.2	1.589498756	0.008598485	UP
**IFT88**	13q12.11	1.571701696	1.62E-07	UP
**EPB41L5**	2q14.2	1.569820667	0.011846657	UP
**MAGEB6**	Xp21.3	1.553982729	0.038944511	UP
**PLA2G6**	22q13.1	1.526398873	0.011101869	UP
**ZHX3**	20q12	1.526252111	0.02054402	UP
**WDFY3**	4q21.23	1.513833798	0.032617117	UP
**HNRNPCL4**	1p36.21	1.51218068	0.043485886	UP
**HECTD2**	10q23.32	1.507417977	0.008997989	UP
**IDNK**	9q21.32	1.504588513	0.038222962	UP
**R3HDML**	20q13.12	1.495786364	0.04123148	UP
**ENTPD5**	14q24.3	1.479308984	0.000750684	UP
**DGKD**	2q37.1	1.445936108	0.022126693	UP
**BTRC**	10q24.32	1.436545921	9.01E-06	UP
**SNHG3**	1p35.3	1.435176513	0.004937954	UP

LOC100505915		1.4338096	0.015223581	UP
EXOC6	10q23.33	1.422477645	0.030451957	UP
EOGT	3p14.1	1.422326482	0.005116459	UP
ZBTB37	1q25.1	1.417990073	0.003784264	UP
PCNX1	14q24.2	1.410913145	0.000668654	UP
ZNF582	19q13.43	1.398077881	0.020218662	UP
GK3P	4q32.3	1.38227024	0.000811216	UP
TTC8	14q31.3	1.377681974	7.34E-06	UP
ARHGAP23	17q12	1.36875668	0.010648749	UP
CDK9	9q34.11	1.35946919	0.023305418	UP
CMC2	16q23.2	1.358532881	0.000371682	UP
TOB2	22q13.2	1.355300124	4.22E-05	UP
MAPKBP1	15q15.1	1.338918465	0.015394337	UP
DDI2	1p36.21	1.337174891	0.032540013	UP
RGPD5	2q13	1.336454481	0.044609441	UP
ENKD1	16q22.1	1.331478242	0.031486131	UP
FAM83G	17p11.2	1.326354965	0.002146799	UP
ALDH18A1	10q24.1	1.320530173	0.00161822	UP
GRAMD1B	11q24.1	1.31234179	0.019425422	UP
EML3	11q12.3	1.309544779	0.035785635	UP
PLPP3	1p32.2	1.309021164	0.018563805	UP
RNLS	10q23.31	1.308138941	0.04420859	UP
BAZ2B	2q24.2	1.301491006	0.032617117	UP
ANTXR2	4q21.21	1.291080118	0.008056658	UP
E2F5	8q21.2	1.289823765	0.004167112	UP
RPS6KA5	14q32.11	1.288622352	0.003137278	UP
MRTFA	22q13.1-q13.2	1.280810876	0.011224551	UP
FOXO1	13q14.11	1.277635128	0.016433856	UP

**PRMT9**	4q31.23	1.275840651	0.019559315	UP
**IFT81**	12q24.11	1.273666165	0.001510834	UP
**PPP3CC**	8p21.3	1.272875484	0.010003113	UP
**AMT**	3p21.31	1.26488444	0.003871572	UP
**DAAM2**	6p21.2	1.264467242	0.029923712	UP
**PPM1D**	17q23.3	1.260086964	0.000167317	UP
**HECTD4**	12q24.13	1.240674198	0.039709511	UP
**MTERF4**	2q37.3	1.226003292	0.003131493	UP
**PHF1**	6p21.32	1.225613059	0.000615941	UP
**SLC5A4**	22q12.3	1.21160606	0.044609441	UP
**RECK**	9p13.3	1.209595469	0.015737524	UP
**STXBP1**	9q34.11	1.206293432	0.044602965	UP
**CCT6A**	7p11.2	1.203662662	0.002756525	UP
**DISP1**	1q41	1.20250727	0.017192159	UP
**MUC1**	1q22	1.202402868	0.030116217	UP
**VWF**	12p13.31	1.19832195	0.023275211	UP
**SAMD15**	14q24.3	1.197106846	0.005462453	UP
**TNFSF4**	1q25.1	1.195097068	0.027324431	UP
**ZNF559**	19p13.2	1.193608716	0.001163045	UP
**SLC24A1**	15q22.31	1.188941443	0.04428363	UP
**MAD2L2**	1p36.22	1.188616086	0.043822192	UP
**EVC2**	4p16.2	1.184521692	0.027032612	UP
**ENTPD3**	3p22.1	1.183514832	0.031833114	UP
**GNPTG**	16p13.3	1.181870131	0.02614963	UP
**DUSP6**	12q21.33	1.179845043	0.008635132	UP
**ACOX3**	4p16.1	1.178071851	0.037414824	UP
**NSUN6**	10p12.31	1.176784837	0.032035613	UP
**IFT140**	16p13.3	1.169955527	0.021191926	UP

**PHLPP1**	18q21.33	1.169049081	0.049610495	UP
**ALDH1B1**	9p13.1	1.168502533	0.036691522	UP
**TTC12**	11q23.2	1.167153621	1.52E-05	UP
**C4orf46**	4q32.1	1.165403586	0.016816588	UP
**GHDC**	17q21.2	1.165084698	0.038982829	UP
**DNAJC27**	2p23.3	1.164256646	0.041074083	UP
**OGA**	10q24.32	1.156713849	0.042233743	UP
**MON2**	12q14.1	1.154580917	0.001156223	UP
**PRICKLE2**	3p14.1	1.144809907	0.032716815	UP
**YIF1B**	19q13.2	1.140505077	0.004744496	UP
**PRKDC**	8q11.21	1.136969547	0.011031552	UP
**SCNN1D**	1p36.33	1.132677321	0.016506225	UP
**ZNF214**	11p15.4	1.130578744	0.024452187	UP
**RARA**	17q21.2	1.129139653	0.028273073	UP
**RNASEH2A**	19p13.13	1.128719145	0.005574378	UP
**ERMARD**	6q27	1.123989716	0.020551978	UP
**ZNF561**	19p13.2	1.120517464	0.043401002	UP
**SUV39H2**	10p13	1.116640254	0.000549595	UP
**ARMCX4**	Xq22.1	1.112892511	0.015351985	UP
**LGI4**	19q13.12	1.111912862	0.02995378	UP
**AP4B1**	1p13.2	1.109856767	0.034042865	UP
**RSPH4A**	6q22.1	1.100775138	0.04750634	UP
**PACC1**	1q32.3	1.098008672	0.002901145	UP
**EEF1E1**	6p24.3	1.09637095	0.000344455	UP
**GAS7**	17p13.1	1.093988811	0.025707915	UP
**IQUB**	7q31.32	1.087544185	0.01965869	UP
**CKS1B**	1q21.3	1.084017888	0.006789051	UP
**DOCK1**	10q26.2	1.083137934	0.012200397	UP

**RBM38**	20q13.31	1.082037883	0.006353716	UP
**SGSH**	17q25.3	1.080994248	0.004950302	UP
**YIPF2**	19p13.2	1.078944208	0.04063949	UP
**NCAPD2**	12p13.31	1.077873633	0.041387133	UP
**ANKAR**	2q32.2	1.077372778	0.019590852	UP
**TRIM41**	5q35.3	1.074391203	0.011846712	UP
**RGS11**	16p13.3	1.07369184	0.04240801	UP
**ACVR2A**	2q22.3-q23.1	1.071691586	0.009260791	UP
**WDR91**	7q33	1.071542229	0.005252678	UP
**CCNF**	16p13.3	1.071332297	0.002595338	UP
**ZNF106**	15q15.1	1.07052922	4.56E-07	UP
**ABTB2**	11p13	1.065546974	0.005825857	UP
**MAPK3**	16p11.2	1.060814556	0.000311231	UP
**PEX11A**	15q26.1	1.057019285	0.007167886	UP
**ZNF724**	19p12	1.054371692	0.007193003	UP
**CCDC144B**	17p11.2	1.052836574	0.023963098	UP
**BOD1L1**	4p15.33	1.049243824	0.017911903	UP
**GNAQ**	9q21.2	1.04637355	0.008196566	UP
**KDM8**	16p12.1	1.046157053	0.022219168	UP
**ATE1**	10q26.13	1.045792208	0.001862575	UP
**RETSAT**	2p11.2	1.045671134	0.00624152	UP
**F8**	Xq28	1.045116139	0.008888485	UP
**DAP3**	1p34.3, 1q22	1.043794025	0.006343447	UP
**USP1**	1p31.3	1.038694973	0.027619493	UP
**SPATA6**	1p33	1.038354729	0.005169129	UP
**STIM2**	4p15.2	1.037554574	0.009234598	UP
**CCL8**	17q12	1.036691294	0.024186583	UP
**H4C8**	6p22.2	1.036323737	0.004118948	UP

**IFT74**	9p21.2	1.033112201	0.037661632	UP
**OLA1**	2q31.1	1.032473205	0.022731578	UP
**STRAP**	12p12.3, 5q23.1	1.031863904	0.011738	UP
**LAMA2**	6q22.33	1.029744867	0.015089527	UP
**H3C8**	6p22.2	1.028911999	0.043822192	UP
**SH3YL1**	2p25.3	1.026874821	0.049941819	UP
**GMPS**	3q25.31	1.026736685	0.016710646	UP
**DRC3**	17p11.2, 19q13.42	1.023035265	0.008635132	UP
**TOM1L2**	17p11.2	1.022938383	0.008881594	UP
**TXNIP**	1q21.1	1.020774308	0.004789862	UP
**VEGFA**	6p21.1	1.020755274	0.029422591	UP
**IDH2**	15q26.1	1.020018786	0.012778211	UP
**YES1**	18p11.32	1.019264441	0.001937555	UP
**TNFRSF21**	6p12.3	1.018692037	0.032719879	UP
**LOC100289511**		1.014461723	0.00102874	UP
**ANAPC2**	9q34.3	1.01432689	0.003022817	UP
**CKMT2**	5q14.1	1.013389613	0.042097285	UP
**STRBP**	9q33.1-q33.3	1.007298955	0.0266412	UP
**TFRC**	3q29	1.00342865	0.00624152	UP
**CARF**	2q33.2, 4q35.1	1.001215089	0.028996102	UP
**B4GALT1**	9p21.1	1.000298704	3.43E-05	UP
**PARPBP**	12q23.2	1.000094925	0.005424074	UP
**PABPC3**	13q12.13	0.998419827	0.001882417	UP
**MAML3**	4q31.1	0.996597063	0.045728674	UP
**TSPAN2**	1p13.2	0.996462581	0.034487621	UP
**N4BP2L2**	13q13.1	0.994778344	0.010662674	UP
**CEBPG**	19q13.11	0.994524138	0.017538062	UP
**STIP1**	11q13.1	0.991122509	0.021500717	UP

**PAPSS1**	4q25	0.987445916	0.046369659	UP
**ACADSB**	10q26.13	0.987218535	0.000122252	UP
**SCAF1**	19q13.3-q13.4, 2p21	0.984187231	0.044912958	UP
**BDH1**	3q29	0.981656676	0.029923712	UP
**HOMER3**	19p13.11	0.979156505	0.004830936	UP
**NUDT19**	19q13.11	0.978550724	0.008174556	UP
**PGAM5**	12q24.33	0.97836932	0.038973855	UP
**CCDC171**	9p22.3	0.968681224	0.014638059	UP
**ZNF491**	19p13.2	0.968262896	0.045157899	UP
**GORASP2**	2q31.1	0.959923316	0.015163773	UP
**SNTB2**	16q22.1	0.956160809	1.44E-05	UP
**UFSP2**	4q35.1	0.952443747	0.012184523	UP
**EFCAB13**	17q21.32	0.949879264	0.002592279	UP
**TPST1**	7q11.21	0.949795549	0.019558518	UP
**H2BC17**	6p22.1	0.947718165	0.044180956	UP
**SLC12A8**	3q21.2	0.945829473	0.024208906	UP
**SSR2**	1q22	0.945437376	0.010401322	UP
**RBM6**	3p21.31	0.937445148	0.018904714	UP
**PSMB4**	1q21.3	0.934694579	0.016160915	UP
**ARID5A**	2q11.2	0.934274856	0.00074737	UP
**PDIA6**	2p25.1	0.928631191	0.011297034	UP
**POLD1**	19q13.3	0.925449867	9.29E-05	UP
**INTU**	4q28.1	0.923014655	0.019337557	UP
**FZD6**	8q22.3	0.922143879	0.037088918	UP
**KLHL41**	2q31.1	0.920705511	0.027438842	UP
**HDGF**	1q23.1	0.918019069	0.015370944	UP
**TBCCD1**	3q27.3	0.917689237	0.026775735	UP
**SNORA54**	11p15.4	0.917266667	0.041868618	UP

**USP18**	22q11.2	0.914402219	0.038330714	UP
**ARHGAP24**	4q21.23-q21.3	0.913475139	0.010779206	UP
**PSPH**	7p11.2	0.912570452	0.024298319	UP
**TARS1**	5p13.3	0.912295781	0.001240595	UP
**ADSS2**	1q44	0.911641627	0.04279921	UP
**ZNF658B**	9p12	0.908341925	0.024051744	UP
**SLC52A2**	8q24.3	0.904527735	0.009328379	UP
**MROH8**	20q11.23	0.903961628	0.010630039	UP
**TMPO**	12q23.1	0.903010335	0.015737524	UP
**TGIF2-RAB5IF**	20q11.23	0.902749105	0.044551183	UP
**ZDHHC3**	3p21.31	0.900730324	0.035625248	UP
**ARHGEF25**	12q13.3	0.900532629	0.00054609	UP
**EIF4A1**	17p13.1	0.899390084	0.00055293	UP
**SLC35A2**	Xp11.23	0.898107242	0.010375296	UP
**DCPS**	11q24.2	0.89737791	0.011553435	UP
**SPTBN2**	11q13.2	0.89395362	0.011310224	UP
**RAI14**	5p13.2	0.892310531	0.008525896	UP
**DCK**	4q13.3	0.888903599	0.002650025	UP
**HELZ**	17q24.2	0.888195507	0.045911207	UP
**TSHZ1**	18q22.3	0.887645479	0.000156362	UP
**H3P6**	2q31.1	0.882881147	0.011864114	UP
**TUBA1C**	12q13.12	0.879360985	0.002724742	UP
**SKA2**	17q23.2	0.878780481	0.039324941	UP
**TRIM24**	7q33-q34	0.877911838	0.030287255	UP
**NSUN5**	7q11.23	0.875717027	0.029128609	UP
**NIPSNAP3B**	9q31.1	0.875466522	0.015163773	UP
**PDIA4**	7q36.1	0.875312446	0.017217484	UP
**BDP1**	2q21.1, 5q13.2	0.872154137	0.032679208	UP

**RCC1**	1p35.3	0.871471175	0.019622042	UP
**PEG3**	19q13.43	0.870142146	0.01751541	UP
**LIN9**	1q42.12	0.869963658	0.048247107	UP
**TFG**	3q12.2	0.868006235	0.031940926	UP
**C5orf34**	5p12	0.867189228	0.016816588	UP
**GART**	21q22.11	0.866790913	0.017902352	UP
**HARS2**	20p11.23, 5q31.3	0.866668919	0.038424772	UP
**PITPNM3**	17p13.2-p13.1	0.862832759	0.038578958	UP
**BCAR3**	1p22.1	0.860614829	0.016415086	UP
**SLC16A3**	17q25.3	0.86009352	0.004118948	UP
**SMC6**	2p24.2	0.859846927	1.15E-05	UP
**FANCB**	Xp22.2	0.859193634	0.037286618	UP
**PNO1**	2p14	0.859177402	0.001862575	UP
**LARP4B**	10p15.3	0.856378834	0.015295248	UP
**WDR77**	1p13.2	0.855331118	0.023212701	UP
**MAIP1**	2q33.1	0.851056634	0.031131944	UP
**NCSTN**	1q23.2	0.848786208	0.003276222	UP
**SSBP2**	5q14.1	0.848657262	0.019767876	UP
**ITGB3BP**	1p31.3	0.847584709	0.038812093	UP
**MRPL11**	11q13.2	0.846893038	0.009977667	UP
**TOMM40**	19q13.32	0.844406169	0.03672625	UP
**PLOD1**	1p36.22	0.84418503	0.014864814	UP
**STARD7**	2q11.2	0.844156735	0.013519647	UP
**SLC29A2**	11q13.2	0.842748296	0.029419343	UP
**NOD1**	7p14.3	0.842140932	0.040771598	UP
**ATXN3**	14q32.12	0.83921868	0.043382528	UP
**ABHD14A**	3p21.2	0.838859797	0.00805746	UP
**ATP7B**	13q14.3	0.837931216	0.019389862	UP

ABCD4	14q24.3	0.837194617	0.000453623	UP
PKD1P3-NPIPA1		0.834537504	0.027958456	UP
GCH1	14q22.2	0.833447639	0.013216805	UP
ARRB1	11q13.4	0.832411053	0.037903673	UP
KDELR2	7p22.1	0.832172168	0.000719272	UP
RECQL4	8q24.3	0.832112484	0.037286618	UP
LRR1	14q21.3	0.830856667	0.010634581	UP
NME1	17q21.33, 9p13.3	0.829865526	0.026596072	UP
WWOX	16q23.1-q23.2	0.82721462	8.95E-05	UP
PDXP	22q13.1	0.827018602	0.010050366	UP
PHYKPL	5q35.3	0.826996079	0.007638696	UP
NIP7	16q22.1	0.826035078	0.015500905	UP
MESD	15q25.1	0.825050965	0.000182031	UP
TOMM34	20q13.12	0.82155214	0.024109121	UP
RELL1	4p14	0.820034035	0.029453509	UP
TEDC2	16p13.3	0.819523829	0.049660568	UP
EMC8	16q24.1	0.817838296	0.009706511	UP
HYOU1	11q23.3	0.815971884	0.005441299	UP
DMXL1	5q23.1	0.815381663	0.012380396	UP
PCMTD2	20q13.33	0.815126107	0.017202738	UP
TBC1D17	19q13.33	0.81224152	0.006225014	UP
ARHGAP22	10q11.22-q11.23	0.811951355	0.040923682	UP
ABCF1	6p21.33	0.811024246	0.040525855	UP
ORAI1	12q24.31	0.810609565	0.023966745	UP
TTC7B	14q32.11	0.809634657	0.001969041	UP
CCT3	1q22	0.809213904	0.019245199	UP
JADE2	5q31.1	0.805437795	0.003881357	UP
TP53BP2	1q41	0.805056334	0.041610108	UP

**ZNF23**	16q22.2	0.804302907	0.003749717	UP
**TXNDC17**	17p13.1	0.803938639	0.023153566	UP
**AMIGO2**	12q13.11	0.803463133	0.024934058	UP
**ST14**	11q24.3	0.792647518	0.0309727	UP
**SNRPE**	1q32.1	0.791749491	0.038894104	UP
**POP4**	19q12, Xp11.21	0.789287287	0.049252809	UP
**GALNT1**	18q12.2	0.788685637	0.001232985	UP
**NHSL2**	Xq13.1	0.788531528	0.032239426	UP
**SSRP1**	11q12.1	0.786222077	0.044128079	UP
**XAB2**	19p13.2	0.786147752	0.033439552	UP
**NDUFB2**	7q34	0.783604271	0.013237198	UP
**KNTC1**	12q24.31	0.782237456	0.006119687	UP
**CCNQ**	Xq28	0.778739385	0.048015014	UP
**HDAC4**	2q37.3	0.777500145	0.04279921	UP
**MRGBP**	20q13.33	0.774055491	0.036855436	UP
**PSMB6**	17p13.2	0.770070409	0.002981316	UP
**PLK4**	4q28.1	0.768133596	0.039234692	UP
**MAGED1**	Xp11.22	0.76344442	0.023090791	UP
**ALG3**	3q27.1	0.759860809	0.013409067	UP
**NDUFS2**	1q23.3	0.758438814	0.021023793	UP
**LRRC45**	17q25.3	0.757045394	0.016004736	UP
**POLE2**	14q21.3	0.752707154	0.032719879	UP
**ANKHD1**	5q31.3	0.750024301	0.038222962	UP
**CLK3**	15q24.1	0.750015484	0.002388026	UP
**RPA3**	7p21.3	0.748005029	0.041117848	UP
**SF3A3**	1p34.3	0.747610843	0.048507485	UP
**XPO5**	6p21.1	0.746878718	0.019389862	UP
**ARL6IP6**	2q23.3	0.745548821	0.023305418	UP

**CAPZA1**	1p13.2	0.744650088	0.011695205	UP
**C1orf131**	1q42.2	0.743102456	0.025884407	UP
**SPINT2**	19q13.2	0.741544101	0.036691522	UP
**COA7**	1p32.3	0.740896684	0.047393152	UP
**ARHGAP11B**	15q13.2	0.739051392	0.013490197	UP
**FGFR2**	10q26.13	0.737968894	0.046137451	UP
**DIXDC1**	11q23.1	0.734891189	0.036180719	UP
**CLMP**	11q24.1	0.731630205	0.010650749	UP
**C3orf18**	3p21.31	0.727710832	0.014502405	UP
**CDC7**	1p22.1	0.726292464	0.011578548	UP
**DPH2**	1p34.1	0.724557574	0.04425988	UP
**ORMDL2**	12q13.2	0.724120302	0.026786206	UP
**PRPS2**	Xp22.2	0.722499763	0.036691522	UP
**LSM5**	7p14.3	0.722017368	0.031486131	UP
**MCM7**	7q22.1	0.721427114	0.031782403	UP
**GYPC**	2q14.3	0.720390256	0.010783163	UP
**VCPIP1**	8q13.1	0.719966161	0.035530609	UP
**DST**	6p12.1	0.719457419	0.023181718	UP
**HAVCR2**	5q33.3	0.718749577	0.011031552	UP
**MEIS2**	15q14	0.717258987	0.018023682	UP
**MRPS17**	7p11.2	0.717040924	0.040927472	UP
**VBP1**	Xq28	0.716592867	0.007005273	UP
**KCTD5**	16p13.3	0.714455637	8.12E-05	UP
**MRPS23**	17q22	0.711327874	0.036110162	UP
**BRCC3**	5q12.1, Xq28	0.708561419	0.001726288	UP
**SRSF3**	6p21.31-p21.2	0.708449619	0.042618097	UP
**CEP68**	2p14	0.707189926	0.008108627	UP
**SMIM30**	7q31.1	0.706591778	0.031782403	UP

**GOLPH3**	5p13.3	0.706396488	0.037495211	UP
**ZFC3H1**	12q21.1	0.704590824	0.015794695	UP
**UBQLN4**	1q22	0.702087743	0.037661632	UP
**AP1S1**	7q22.1	0.700810904	0.019294465	UP
**SH2B1**	16p11.2	0.699390045	0.005809342	UP
**ATP5MPL**	14q32.33	0.698589561	0.00502757	UP
**ZNF548**	19q13.43	0.697516726	0.042152376	UP
**RCC2**	1p36.13	0.69694979	0.030487115	UP
**RPN1**	3q21.3	0.696906742	0.012200397	UP
**TSPAN17**	5q35.2	0.696164543	0.03114018	UP
**ANP32B**	9q22.33	0.695802727	0.011461061	UP
**AKAP6**	14q12	0.692992315	0.01870179	UP
**PPP1R11**	6p22.1	0.691393174	0.009095243	UP
**ARPC2**	2q35	0.687367225	0.00014621	UP
**ZBTB7A**	19p13.3	0.68363444	0.022978666	UP
**H2AC12**	6p22.1	0.682761658	0.029923712	UP
**PABPC4**	1p34.3	0.681637099	0.003052691	UP
**IL18**	11q23.1	0.680131693	0.031811913	UP
**SESN1**	6q21	0.680064179	0.043406026	UP
**C6orf62**	6p22.3	0.679724477	4.54E-05	UP
**ALDH6A1**	14q24.3	0.679324708	0.046195614	UP
**AP2M1**	3q27.1	0.677231107	0.004166502	UP
**SEC61A1**	3q21.3	0.67456939	0.002398016	UP
**ARPC5L**	9q33.3	0.673256406	0.01010739	UP
**PSMD2**	3q27.1	0.672808809	0.024429558	UP
**CALU**	7q32.1	0.670204195	0.043822192	UP
**BAX**	19q13.33	0.669263887	0.034394117	UP
**GPX8**	5q11.2	0.668670644	0.04420859	UP

**RAP2A**	13q32.1	0.667745183	0.037587827	UP
**R3HDM1**	2q21.3	0.667540011	0.037171682	UP
**PDCL3**	2q11.2	0.667089852	0.000876661	UP
**CAD**	13q22.3, 1p36.32, 2p23.3	0.665387109	0.043006047	UP
**BID**	22q11.21	0.664920628	0.034041325	UP
**CENPO**	2p23.3	0.660752346	0.000306298	UP
**MCC**	5q22.2	0.659778697	0.038710587	UP
**TARS3**	15q26.3	0.657763197	0.007868274	UP
**WDR76**	15q15.3	0.657614094	0.049316806	UP
**HENMT1**	1p13.3	0.656032057	0.025768947	UP
**DDOST**	1p36.12	0.655419133	0.031440733	UP
**CAP1**	16p11.2, 1p34.2	0.652872237	0.00062346	UP
**TXNL4A**	18q23	0.652870254	0.007940452	UP
**PCDH12**	5q31.3	0.650964926	0.009790821	UP
**PDSS1**	10p12.1	0.650686628	0.011292837	UP
**SERBP1**	1p31.3	0.64835333	0.033133146	UP
**NOP58**	2q33.1	0.648296647	0.022343356	UP
**MYL12A**	18p11.31	0.646359303	0.038651577	UP
**FAM210A**	18p11.21	0.642834094	0.007794922	UP
**ATP6V1F**	7q32.1	0.642452323	0.006119687	UP
**ACTG1P20**	1p36.11	0.642413878	0.004540281	UP
**ENOPH1**	4q21.22	0.642225801	0.000485568	UP
**ATP6AP2**	Xp11.4	0.64191486	0.043601174	UP
**RFC5**	12q24.23	0.640268994	0.010302384	UP
**SETMAR**	3p26.1	0.637526468	0.030733656	UP
**EIF2AK2**	2p22.2	0.637190565	0.018002936	UP
**MAZ**	16p11.2	0.636187464	0.006926766	UP

**L3MBTL3**	6q23.1	0.636098692	0.017833426	UP
**SHLD2P3**	10q11.22	0.636002904	0.015448001	UP
**PFN1**	17p13.2	0.635422791	0.001156223	UP
**ITPK1**	14q32.12	0.633548307	0.001441639	UP
**YEATS2**	3q27.1	0.633027092	0.003155981	UP
**TMEM258**	11q12.2	0.631783773	0.015470305	UP
**CCNYL1**	2q33.3	0.631347656	0.017721263	UP
**PTP4A1**	6q12	0.630470185	0.049332117	UP
**TK2**	16q21	0.627630638	0.029453509	UP
**MAN2B1**	19p13.13	0.625923855	0.016201349	UP
**PLEKHB2**	2q21.1	0.621370047	0.003008864	UP
**ERI1**	8p23.1	0.619366681	0.004401658	UP
**RAB29**	1q32.1	0.618726771	0.002173767	UP
**EPHA2**	1p36.13	0.617760455	0.03186548	UP
**DUSP12**	1q23.3	0.613620174	0.000822137	UP
**HYLS1**	11q24.2	0.613392718	0.045397979	UP
**ATP5F1C**	10p14	0.611763417	0.037299766	UP
**APEX2**	Xp11.21	0.611227605	0.036302149	UP
**KIAA0825**	5q15	0.610282492	0.037712697	UP
**MARS1**	12q13.3	0.608839114	0.00303172	UP
**CFAP20**	16q21	0.607986615	0.003986588	UP
**CDC5L**	6p21.1	0.6073479	0.045372636	UP
**RDH11**	14q24.1	0.605222528	0.006441722	UP
**PRIM1**	12q13.3	0.603552878	0.003639373	UP
**ACTR2**	2p14	0.602037626	0.005313224	UP
**ANKRD53**	2p13.3	0.600242449	0.022316019	UP
**DNASE2**	19p13.13	0.598599325	0.049380531	UP
**INTS8**	8q22.1	0.597551007	0.007130485	UP

**SNRPA1**	15q26.3	0.596104257	0.040476344	UP
**CSTF2**	Xq22.1	0.593055446	0.024253235	UP
**GOLT1B**	12p12.1	0.592971129	0.008890856	UP
**CKLF**	13q22.1, 16q21	0.588636546	0.005994348	UP
**RANBP9**	6p23	0.583512503	0.029923712	UP
**SAMD1**	19p13.12	0.583042172	0.00412276	UP
**LSM14A**	19q13.11	0.582010151	0.038871052	UP
**SMG5**	1q22	0.581965054	0.030236659	UP
**SSR1**	6p24.3	0.581931799	0.017741326	UP
**COQ2**	4q21.23	0.579332237	0.007884052	UP
**KHSRP**	19p13.3	0.579044511	0.008038226	UP
**ZNF37BP**	10q11.21	0.578967586	0.015365278	UP
**CASTOR3**	7q22.1	0.578468052	0.04568659	UP
**BRMS1**	11q13.2, 14q13.2	0.577276174	0.011353784	UP
**ITPA**	20p13	0.574313673	0.011003823	UP
**SNX8**	7p22.3	0.569107181	0.009064252	UP
**RTL8A**	Xq26.3	0.567625891	0.021943969	UP
**IPO9**	1q32.1	0.562839637	0.0041372	UP
**ATL2**	2p22.2-p22.1	0.560432763	0.042134543	UP
**MITF**	3p13	0.559646075	0.018184287	UP
**TMEM104**	17q25.1	0.559563608	0.000965602	UP
**NARS1**	18q21.31	0.559326624	0.008741059	UP
**KPNA4**	3q25.33	0.558063453	0.019264013	UP
**TAX1BP3**	17p13.2	0.557373514	0.001464765	UP
**TOPBP1**	3q22.1	0.556728096	0.007406684	UP
**ICMT**	1p36.31	0.556645804	0.008913249	UP
**TM9SF2**	13q32.3	0.555128443	0.021916575	UP
**COMMD6**	13q22.2	0.550335011	0.020821909	UP

**TOMM22**	22q13.1	0.544440887	0.02030283	UP
**FAM91A1**	8q24.13	0.542622814	0.00438615	UP
**NME2**	17q21.33	0.541468788	0.04470483	UP
**UBE2D1**	10q21.1	0.539918087	0.000609026	UP
**NACC1**	19p13.13	0.539641637	0.021701236	UP
**UQCRB**	8q22.1	0.539527665	0.030891796	UP
**ACTG1**	17q25.3	0.539080085	0.034921993	UP
**TBL2**	7q11.23	0.537839374	0.032716815	UP
**ARMC6**	19p13.11	0.537804943	0.021923309	UP
**BTF3L4**	1p32.3	0.536530563	0.028291008	UP
**DYNC2H1**	11q22.3	0.536477658	0.047490526	UP
**CMTM6**	3p22.3	0.536054382	0.007757006	UP
**CSNK2A3**	11p15.4	0.533907978	0.04811239	UP
**GTF2H4**	6p21.33	0.530460323	0.023780152	UP
**YLPM1**	14q24.3	0.528827936	0.037990294	UP
**PSMD1**	2q37.1	0.527351276	0.000212453	UP
**SLC37A4**	11q23.3	0.526589159	0.033589104	UP
**RPS10**	6p21.31	0.52610879	0.019118259	UP
**PTPRJ**	11p11.2	0.525120537	0.033696708	UP
**XPOT**	12q14.2	0.522931117	0.046973637	UP
**SLC9A6**	Xq26.3	0.522832431	0.04102979	UP
**SLC50A1**	1q22	0.522644804	0.034166202	UP
**TXNDC9**	2q11.2	0.522398271	0.000490363	UP
**FTSJ1**	Xp11.23	0.521467145	0.008612297	UP
**ZDHHC18**	1p36.11	0.521165334	0.001127384	UP
**STMP1**	7q33	0.520018824	0.017887703	UP
**MTHFR**	1p36.22	0.518514667	0.032314922	UP
**COX8A**	11q13.1	0.517761109	0.02394104	UP

**GCA**	2q24.2	0.516643202	0.008997989	UP
**RPL28**	19q13.42	0.516264569	0.028164186	UP
**CCDC43**	17q21.31	0.512796194	0.016940642	UP
**SDHC**	1q23.3	0.507184117	0.038222962	UP
**ZNF483**	9q31.3	0.505419495	0.032716815	UP
**TBC1D1**	4p14	0.504154334	0.040167061	UP
**DESI1**	22q13.2	0.500510165	0.007710754	UP
**COPG1**	3q21.3	0.50008	0.002051836	UP
**URI1**	19q12	0.496387842	0.045722179	UP
**VDR**	12q13.11	0.494158855	0.038149163	UP
**MRPS33**	7q34	0.493408096	0.031150844	UP
**MRPS34**	16p13.3	0.490630887	0.039032609	UP
**MAPKAPK2**	1q32.1	0.488492111	0.036166218	UP
**TOR3A**	1q25.2	0.484942885	0.001710252	UP
**PANX1**	11q21	0.48357912	0.003125937	UP
**ARPC3**	12q24.11	0.477988482	0.006768701	UP
**CLPP**	19p13.3	0.475520901	0.04504887	UP
**ZBTB8OS**	1p35.1	0.475245787	0.001131523	UP
**EAF1**	3p25.1	0.472427342	0.021130671	UP
**GSPT1**	16p13.13	0.471624645	0.006063134	UP
**SIAH2**	3q25.1	0.466770294	0.027528222	UP
**NSDHL**	Xq28	0.462173785	0.041857832	UP
**PHF19**	9q33.2	0.461530096	0.016415086	UP
**RBBP4**	1p35.1	0.460944861	0.022343356	UP
**TBRG4**	7p13	0.460828139	0.03831673	UP
**SET**	9q34.11	0.457443576	0.045484742	UP
**MRPL44**	2q36.1	0.457327713	0.005040624	UP
**PNKD**	2q35	0.457073077	0.017999788	UP

**MFSD12**	19p13.3	0.450224429	0.049334251	UP
**FTH1**	11q12.3	0.449104875	0.049839648	UP
**SLC31A1**	9q32	0.448392943	0.021466118	UP
**SH3BP5L**	1q44	0.44800423	0.02596741	UP
**SFXN1**	5q35.2	0.445050331	0.028164186	UP
**MRPL21**	11q13.3	0.440312777	0.031209106	UP
**GNB1**	1p36.33	0.437444167	0.003294831	UP
**HSPH1**	13q12.3	0.436757135	0.035826341	UP
**IFNGR2**	21q22.11	0.435706244	0.044556637	UP
**TWF2**	3p21.2	0.435668779	0.023917901	UP
**RHOA**	3p21.31	0.42351603	0.041465061	UP
**HNRNPF**	10q11.21	0.417804861	0.005975843	UP
**MEMO1**	2p22.3	0.41765611	0.0004242	UP
**LAMTOR5**	1p13.3	0.414909949	0.043244571	UP
**DPP3**	11q13.2	0.412957894	0.03672625	UP
**TRMT44**	4p16.1	0.412083835	0.043956803	UP
**SEPHS2**	16p11.2	0.411320188	0.013314395	UP
**GET4**	7p22.3	0.409282202	0.041365777	UP
**SRM**	1p36.22, 20q13.33	0.4079417	0.035916179	UP
**UBE2W**	8q21.11	0.406177823	0.035811096	UP
**PUS3**	11q24.2	0.405916604	0.019554427	UP
**HCFC1**	Xq28	0.403208993	0.035175462	UP
**UGGT1**	2q14.3	0.400341209	0.000891923	UP
**SIRT7**	17q25.3	0.399887129	0.024124076	UP
**PTPMT1**	11p11.2	0.399761886	0.03749423	UP
**TNPO3**	7q32.1	0.396327725	0.04772122	UP
**RRP15**	1q41	0.393247706	0.042366649	UP
**RLIM**	Xq13.2	0.392680197	0.001355506	UP

**IFT20**	17q11.2	0.391254736	0.046580385	UP
**TSEN54**	17q25.1	0.390747136	0.022855213	UP
**JOSD1**	22q13.1	0.386718556	0.039234692	UP
**SARNP**	12q13.2	0.383102344	0.046986884	UP
**ZNHIT3**	17q12	0.380150142	0.027459008	UP
**NPC1**	18q11.2	0.376092124	0.032716815	UP
**UBL7**	15q24.1	0.373656945	0.018904714	UP
**FAM118B**	11q24.2	0.368097843	0.048575057	UP
**CCDC92**	12q24.31	0.364712236	0.037286618	UP
**UBE3C**	7q36.3	0.364169895	0.048420721	UP
**COP1**	11q22.3, 1q25.1-q25.2	0.362394228	0.019879065	UP
**PLEKHA8**	7p14.3	0.353836323	0.04063949	UP
**ELK1**	Xp11.23	0.347335102	0.013685361	UP
**ARCN1**	11q23.3	0.339590986	0.02840473	UP
**EIF4E**	4q23	0.336337534	0.030079422	UP
**XPO6**	16p12.1	0.331405779	0.022724482	UP
**ACLY**	17q21.2	0.324363668	0.031376206	UP
**MED8**	1p34.2	0.315905322	0.045612027	UP
**NEMP1**	12q13.3	0.304149263	0.031490653	UP
**NECAP2**	1p36.13	0.286765971	0.048529973	UP
**HNRNPL**	19q13.2	0.286409016	0.044190138	UP
**RAVER1**	19p13.2	0.280456758	0.010530586	UP
**COPB1**	11p15.2	0.278588039	0.000470761	UP
**VPS35**	16q11.2	0.277156828	0.04856385	UP
**GANAB**	11q12.3	0.266187184	0.015797782	UP
**UBE2G1**	17p13.2	0.258401639	0.021068363	UP
**GMPPA**	2q35	0.25504289	0.027324431	UP
**HNRNPUL1**	19q13.2	0.237444353	0.031490653	UP

**EML4**	2p21	0.233108055	0.009443574	UP
**SLC15A4**	12q24.33	0.230921758	0.017854772	UP
**WDR26**	1q42.11-q42.12	0.174722953	0.040894412	UP
**WIPF2**	17q21.2	-0.14095965	0.028713669	DOWN
**C7orf26**	7p22.1	-0.181319774	0.028587299	DOWN
**PRDM10**	11q24.3	-0.184100506	0.013068358	DOWN
**SF1**	11q13.1, 9q33.3	-0.187243863	0.00304618	DOWN
**CDK12**	17q12	-0.188776316	0.005243666	DOWN
**RANBP10**	16q22	-0.190706287	0.030221188	DOWN
**BAZ2A**	12q13.3	-0.191418139	0.000314343	DOWN
**RIC8B**	12q23.3	-0.194869036	0.041820394	DOWN
**GAPVD1**	9q33.3	-0.197926973	0.041387133	DOWN
**MOAP1**	14q32.12	-0.198470822	0.02621185	DOWN
**TUT1**	11q12.3	-0.198891828	0.01680818	DOWN
**WHAMM**	15q25.2	-0.199334967	0.038439185	DOWN
**TSTD2**	9q22.33	-0.199730002	0.009211179	DOWN
**EP400**	12q24.33	-0.20015703	0.034545338	DOWN
**CIPC**	14q24.3	-0.200448529	0.015163773	DOWN
**ARID2**	12q12	-0.201103109	0.00734572	DOWN
**SP2**	17q21.32	-0.203080106	0.000242859	DOWN
**LZTS2**	10q24.31	-0.204699855	0.026521885	DOWN
**NFE2L2**	2q31.2	-0.2083898	0.043668111	DOWN
**PCBP2-OT1**	12q13.13	-0.210524642	0.046189453	DOWN
**ATG14**	14q22.3	-0.210713696	0.039698119	DOWN
**RHBDD1**	2q36.3	-0.21286008	0.039018575	DOWN
**MNT**	17p13.3	-0.214088122	0.018442999	DOWN
**RBM26**	13q31.1	-0.216448669	0.047745273	DOWN
**MYO1C**	15q22.2, 17p13.3	-0.218278408	0.024511819	DOWN

**SMARCC2**	12q13.2	-0.219673794	0.010375296	DOWN
**HEATR5B**	2p22.2	-0.219878164	0.001918053	DOWN
**FKTN**	9q31.2	-0.22063079	0.042381233	DOWN
**DIDO1**	20q13.33	-0.221901139	0.025090654	DOWN
**EPG5**	18q12.3-q21.1	-0.222159617	0.048768655	DOWN
**ZNF207**	17q11.2	-0.222409715	0.019044508	DOWN
**FBXL17**	5q21.3	-0.223858728	0.003002481	DOWN
**UBR3**	2q31.1	-0.2239435	0.000624395	DOWN
**KDM3B**	5q31.2	-0.225264685	0.04465884	DOWN
**SAMD8**	10q22.2	-0.226023214	0.036260371	DOWN
**NCOR2**	12q24.31	-0.226614317	0.008458807	DOWN
**TSPAN31**	12q14.1	-0.226692345	0.01553097	DOWN
**KDM6A**	Xp11.3	-0.228753517	0.028889244	DOWN
**PHC3**	3q26.2	-0.230519738	0.005372485	DOWN
**GPATCH8**	17q21.31	-0.232074729	0.032347304	DOWN
**FAM120AOS**	9q22.31	-0.233845415	0.02309692	DOWN
**SZT2**	1p34.2	-0.235037394	0.005066643	DOWN
**TPCN2**	11q13.3	-0.235393315	0.007629579	DOWN
**CRELD1**	3p25.3	-0.23909944	0.041823395	DOWN
**RALGAPA1P1**	9q31.2	-0.2395109	0.041780709	DOWN
**ZZEF1**	17p13.2	-0.239520005	0.000189	DOWN
**BCL2L2**	14q11.2	-0.240458359	0.042767024	DOWN
**ATXN2**	12q24.12	-0.241714482	0.00743956	DOWN
**ELL**	19p13.11	-0.244768091	0.035409917	DOWN
**SUOX**	12q13.2	-0.248453933	0.045670523	DOWN
**XYLT2**	17q21.33	-0.249585816	0.003938048	DOWN
**ZFYVE1**	14q24.2	-0.249947581	0.04428363	DOWN
**MARF1**	16p13.11	-0.25267098	0.000736104	DOWN

**ZNF624**	17p11.2	-0.252738794	0.035765973	DOWN
**ST3GAL3**	1p34.1	-0.252793858	0.030417753	DOWN
**ZNF518A**	10q24.1	-0.25320981	0.011813924	DOWN
**FNDC3A**	13q14.2	-0.254178833	0.017356908	DOWN
**MAPK7**	17p11.2	-0.259352008	0.027651626	DOWN
**CCDC71**	3p21.31	-0.259739555	0.004446476	DOWN
**MPRIP**	17p11.2	-0.260536063	0.004717332	DOWN
**SYNRG**	17q12	-0.262096736	0.03132123	DOWN
**NAF1**	4q32.2, 5q33.1	-0.263212971	0.038222962	DOWN
**ZFYVE26**	14q24.1	-0.263330642	0.030245149	DOWN
**PISD**	22q12.2	-0.264797841	0.005920593	DOWN
**SHPK**	17p13.2	-0.265505321	0.015500905	DOWN
**RUNDC1**	17q21.31	-0.265641219	0.000815146	DOWN
**FPGS**	9q34.11	-0.270622912	0.018442999	DOWN
**CES2**	16q12.2, 16q22.1	-0.273426656	0.022656136	DOWN
**ZNF197**	3p21.31	-0.273611811	0.028041402	DOWN
**AKAP11**	13q14.11	-0.274213446	0.028264464	DOWN
**TNRC6B**	22q13.1	-0.27430629	0.002284317	DOWN
**ERMAP**	1p34.2	-0.274640836	0.015500905	DOWN
**POLG2**	17q23.3	-0.274731317	0.030224852	DOWN
**ZNF25**	10p11.21	-0.274758631	0.03635547	DOWN
**FNBP4**	11p11.2	-0.276171594	0.003910275	DOWN
**TAF1L**	9p21.1	-0.276300977	0.000156362	DOWN
**RBM33**	7q36.3	-0.279032669	0.013043612	DOWN
**ELP1**	9q31.3	-0.280034237	0.0385416	DOWN
**IDS**	Xq28	-0.281237716	0.029461106	DOWN
**TSEN2**	3p25.2	-0.281676875	0.040135235	DOWN
**SEC24B**	4q25	-0.281882312	0.011478963	DOWN

**SETD4**	21q22.12	-0.282037022	0.046530253	DOWN
**ASTE1**	3q22.1	-0.283284021	0.024199035	DOWN
**OGG1**	3p25.3	-0.284764135	0.04938187	DOWN
**CLASP2**	3p22.3	-0.285581874	0.000486758	DOWN
**RBM4B**	11q13.2	-0.285768069	0.039129045	DOWN
**ZNF641**	12q13.11	-0.287710046	0.01439854	DOWN
**TRPV1**	17p13.2	-0.290910985	0.020844622	DOWN
**ASMTL**	Xp22.3 and Yp11.3	-0.29154276	0.010120468	DOWN
**POLR2J4**	7p13	-0.29197762	0.011122251	DOWN
**CDAN1**	15q15.2	-0.292579255	5.60E-05	DOWN
**CBWD5**	9q21.11	-0.293204537	0.026622196	DOWN
**SMG1P1**	16p12.2	-0.294699825	0.046997991	DOWN
**SOS2**	14q21.3	-0.295609366	0.001158186	DOWN
**MKLN1**	7q32.3	-0.296224301	0.006023752	DOWN
**APBB3**	5q31.3	-0.296888401	0.023167575	DOWN
**CRTC1**	19p13.11	-0.298886018	0.019389862	DOWN
**TANC1**	2q24.2	-0.298895485	0.015295248	DOWN
**FAM185A**	7q22.1	-0.300947238	0.001809223	DOWN
**NBEAL1**	2q33.2	-0.303794878	0.001240595	DOWN
**BTD**	3p25.1	-0.304754408	0.000399562	DOWN
**TP53BP1**	15q15.3	-0.306413711	7.52E-06	DOWN
**INPP5E**	9q34.3	-0.306472931	0.017417116	DOWN
**STN1**	10q24.33	-0.307469816	0.002688232	DOWN
**WBP1L**	10q24.32	-0.307759075	0.005214597	DOWN
**CDC14B**	9q22.32-q22.33	-0.308400285	0.011981843	DOWN
**EXOC4**	7q33	-0.309014996	0.019884988	DOWN
**NLRX1**	11q23.3	-0.309431534	0.021800178	DOWN
**DLSTP1**	1p31.1	-0.310686575	0.005719042	DOWN

**PRDM2**	1p36.21	-0.310731615	0.000999916	DOWN
**ZSCAN26**	6p22.1	-0.311992964	0.002699649	DOWN
**NUMBL**	19q13.2	-0.312183563	0.005662475	DOWN
**HIPK2**	7q34	-0.31232177	0.049851545	DOWN
**MAT2A**	2p11.2	-0.313466514	0.035530609	DOWN
**LMBR1L**	12q13.12	-0.313923556	0.000742766	DOWN
**ZSCAN30**	18q12.2	-0.31427341	0.031378033	DOWN
**GATAD1**	7q21.2	-0.315620018	0.000200589	DOWN
**C2CD5**	12p12.1	-0.316270267	0.036302149	DOWN
**RASA1**	5q14.3	-0.317187394	0.004118948	DOWN
**LRRTM2**	5q31.2	-0.318215711	0.035652558	DOWN
**CCDC149**	4p15.2	-0.318424143	0.002688232	DOWN
**PKD1P6-NPIPP1**		-0.318578837	0.000236562	DOWN
**ZNF592**	15q25.3	-0.318728731	0.016415086	DOWN
**ZBTB38**	3q23	-0.321377402	0.001153614	DOWN
**SELENOO**	22q13.33	-0.321962203	0.007462509	DOWN
**LOC100288162**		-0.323117336	0.013119357	DOWN
**PACRGL**	4p15.31	-0.323959403	0.009515938	DOWN
**MARCHF8**	10q11.21-q11.22	-0.324367515	0.011621746	DOWN
**TAMM41**	3p25.2	-0.324423172	7.84E-05	DOWN
**ENGASE**	17q25.3	-0.325017733	0.02030283	DOWN
**ZNF181**	19q13.11	-0.325283943	0.0329089	DOWN
**GORASP1**	3p22.2	-0.325560015	0.003166135	DOWN
**UBOX5**	20p13	-0.325653233	0.004841128	DOWN
**CBR4**	4q32.3	-0.326130248	0.002742968	DOWN
**CASKIN2**	17q25.1	-0.326812222	0.000147979	DOWN
**THAP6**	4q21.1	-0.327856098	0.012030612	DOWN
**WDR73**	15q25.2	-0.328459868	8.29E-05	DOWN

**ZNF585A**	19q13.12	-0.329083806	0.02614963	DOWN
**ZNF783**	7q36.1	-0.329735259	0.048328886	DOWN
**CYP2R1**	11p15.2	-0.330367592	0.040476344	DOWN
**ZFAND4**	10q11.22	-0.331074214	0.010032621	DOWN
**FNIP1**	5q31.1	-0.331853795	0.002036579	DOWN
**ZNF383**	19q13.13	-0.331882811	0.022350705	DOWN
**NCKIPSD**	3p21.31	-0.331957672	0.009328379	DOWN
**USP33**	1p31.1	-0.33203025	0.012660964	DOWN
**ERC1**	12p13.33	-0.333607985	0.020773448	DOWN
**ZFHX3**	16q22.2-q22.3	-0.336095233	0.008196566	DOWN
**LATS1**	6q25.1	-0.337186515	0.000990676	DOWN
**CBLB**	3q13.11	-0.337741798	0.002660452	DOWN
**LOC729558**		-0.338126391	0.047704324	DOWN
**WDR13**	Xp11.23	-0.33827266	0.012043922	DOWN
**CLCN6**	1p36.22	-0.338944259	0.013549327	DOWN
**DMWD**	19q13.32	-0.340665862	0.045413416	DOWN
**ZFYVE16**	5q14.1	-0.342736643	0.00054609	DOWN
**TYW1B**	7q11.22-q11.23	-0.344562589	0.013612047	DOWN
**ZFYVE19**	15q15.1	-0.344719523	0.020844622	DOWN
**BLCAP**	20q11.23	-0.345470764	0.033693939	DOWN
**ASB8**	12q13.11	-0.345733514	0.007333233	DOWN
**ING5**	2q37.3	-0.34649927	0.027417354	DOWN
**TBC1D4**	13q22.2	-0.346754531	0.005188516	DOWN
**ZNF35**	3p21.31	-0.346915153	0.020533271	DOWN
**TSC2**	16p13.3	-0.34798836	2.66E-05	DOWN
**RMND5B**	5q35.3	-0.349867994	0.013041589	DOWN
**NAA80**	3p21.31	-0.350503616	0.02607791	DOWN
**DENND4A**	15q22.31	-0.350579843	0.002044692	DOWN

**RGL4**	22q11.23	-0.352608688	0.037412088	DOWN
**LPP**	3q27.3-q28	-0.353344769	0.009542104	DOWN
**CNNM3**	2q11.2	-0.354450234	0.042391362	DOWN
**ATRIP**	3p21.31	-0.354592953	0.000752727	DOWN
**CSF1**	1p13.3	-0.35592966	0.029453509	DOWN
**ZNF573**	19q13.12	-0.355941362	0.008733253	DOWN
**ZFP36L1**	14q24.1	-0.356896347	0.045091091	DOWN
**SRGAP2B**	1q21.1	-0.358139402	0.028009621	DOWN
**ZC2HC1A**	8q21.13	-0.358436961	0.04467042	DOWN
**USP20**	9q34.2	-0.359121065	0.041387133	DOWN
**PCDHGA12**	5q31.3	-0.359190544	0.039553195	DOWN
**TMOD2**	15q21.2	-0.360009561	0.044992034	DOWN
**CLTCL1**	22q11.21	-0.361618458	0.037990294	DOWN
**USP4**	3p21.31	-0.361851947	0.001236462	DOWN
**TRANK1**	3p22.2	-0.361966956	0.009706511	DOWN
**KIF13A**	6p22.3	-0.362788748	0.001063211	DOWN
**ZFP14**	19q13.12	-0.365557997	0.016650081	DOWN
**ACSF2**	17q21.33	-0.367492191	0.040924632	DOWN
**SP1**	12q13.13	-0.368673001	0.000750684	DOWN
**ANKRD26**	10p12.1	-0.36902078	0.000485568	DOWN
**ZNF304**	19q13.43	-0.36961577	0.04214928	DOWN
**CEP44**	4q34.1	-0.370234179	0.000451232	DOWN
**SECISBP2L**	15q21.1	-0.370626854	0.000166704	DOWN
**RBKS**	2p23.2	-0.371341091	0.028164186	DOWN
**ZC3H12A**	1p34.3	-0.371652434	0.026716558	DOWN
**ASPH**	8q12.3	-0.372135865	0.025024086	DOWN
**TENT2**	5q14.1	-0.372938244	0.046908361	DOWN
**ZC3H14**	14q31.3	-0.372976582	0.000281083	DOWN

**MAP3K1**	5q11.2	-0.373334881	0.008316483	DOWN
**ANKMY2**	7p21.1	-0.37350444	0.011811864	DOWN
**CREBL2**	12p13.1	-0.373963705	0.004104404	DOWN
**TBRG1**	11q24.2	-0.376007961	0.024452187	DOWN
**ELP2**	18q12.2	-0.37604116	0.012200397	DOWN
**STS**	Xp22.31	-0.376409958	0.033024801	DOWN
**CDKL1**	14q21.3	-0.37724848	0.025520174	DOWN
**ZNF136**	19p13.2	-0.377964045	0.000101826	DOWN
**PPIP5K2**	5q21.1	-0.378447798	0.002701761	DOWN
**SPDYE21**	7q11.21	-0.378613267	0.028132297	DOWN
**LOC389765**		-0.37921747	0.034382393	DOWN
**SLC4A7**	3p24.1	-0.379769128	0.009187347	DOWN
**CLHC1**	2p16.1	-0.380446596	0.033306693	DOWN
**ZSWIM8**	10q22.2	-0.380816446	0.008043556	DOWN
**ZSCAN25**	7q22.1	-0.382053836	0.030116217	DOWN
**TMEM192**	4q32.3	-0.382818762	0.027417487	DOWN
**CASC3**	17q21.1	-0.382908745	0.002986294	DOWN
**FLYWCH1**	16p13.3	-0.383298094	0.036338535	DOWN
**ZNF660-ZNF197**		-0.383507415	0.000556909	DOWN
**CABYR**	18q11.2	-0.383682592	0.040844347	DOWN
**GPC1**	2q37.3	-0.38388028	0.027498791	DOWN
**ZNF549**	19q13.43	-0.384124718	0.002212341	DOWN
**FHL2**	2q12.2	-0.384570391	0.030873136	DOWN
**MTMR9**	8p23.1	-0.384966468	0.011579243	DOWN
**NCOA1**	2p23.3	-0.386359655	0.002839923	DOWN
**CLK1**	2q33.1	-0.387240457	0.00713698	DOWN
**UBR1**	15q15.2	-0.387342209	1.53E-05	DOWN
**FBXO8**	4q34.1	-0.387419493	0.011022112	DOWN

**MICA**	6p21.33	-0.388444395	0.008544485	DOWN
**MLH3**	14q24.3	-0.389140841	8.26E-06	DOWN
**TMEM175**	4p16.3	-0.389155177	0.001058508	DOWN
**PKP4**	2q24.1	-0.389279974	0.007075229	DOWN
**OSMR**	5p13.1	-0.390390386	0.043244571	DOWN
**SLC9A3R2**	16p13.3	-0.390668631	0.045629304	DOWN
**INO80**	15q15.1	-0.391750796	0.001744089	DOWN
**ZNF70**	22q11.23	-0.391852352	0.001063211	DOWN
**RELCH**	18q21.33	-0.392132705	0.019878133	DOWN
**L3HYPDH**	14q23.1	-0.392408456	0.000400212	DOWN
**NCKAP5L**	12q13.12	-0.392686152	0.007745696	DOWN
**NSUN3**	3q11.2	-0.393299438	0.012200397	DOWN
**NEK3**	13q14.3	-0.395985405	0.009271071	DOWN
**GLCCI1**	7p21.3	-0.39625384	0.039158822	DOWN
**HEG1**	3q21.2	-0.396297913	0.015487141	DOWN
**ERO1B**	1q42.3	-0.397948484	0.000282672	DOWN
**NAGS**	17q21.31	-0.398392185	0.032818808	DOWN
**YPEL3**	16p11.2	-0.399357788	0.012043922	DOWN
**PREPL**	2p21	-0.399520948	0.008213029	DOWN
**KLF7**	2q33.3	-0.399536552	0.002044764	DOWN
**DMAP1**	1p34.1	-0.399950131	0.013118745	DOWN
**EML1**	14q32.2	-0.400430379	0.028164186	DOWN
**PCNX4**	14q23.1	-0.40157483	0.006290237	DOWN
**HES1**	21q22.3, 3q29	-0.401926104	0.027091707	DOWN
**ZNF345**	19q13.12	-0.402227695	0.048575057	DOWN
**ZNF619**	3p22.1	-0.402356042	0.000261258	DOWN
**HSD17B4**	5q23.1	-0.402824079	0.011751879	DOWN
**CASTOR2**	7q11.23	-0.403403658	0.017379222	DOWN

**RAPGEF2**	4q32.1	-0.406171643	9.50E-05	DOWN
**CNNM2**	10q24.32	-0.407139273	0.008912724	DOWN
**BRAF**	7q34	-0.407727092	0.01191254	DOWN
**MKS1**	17q22	-0.409163898	0.002243105	DOWN
**SYNE2**	14q23.2	-0.409757881	0.008516707	DOWN
**ZNF692**	1q44	-0.409788925	0.034487621	DOWN
**RALGDS**	9q34.13-q34.2	-0.410096604	0.014664435	DOWN
**VASN**	16p13.3	-0.41107695	0.037286618	DOWN
**NBPF9**	1q21.2	-0.41126636	0.009949674	DOWN
**COQ6**	14q24.3	-0.411636501	3.33E-05	DOWN
**RSRP1**	1p36.11	-0.412920415	0.024814617	DOWN
**ZFHX2**	14q11.2	-0.413013179	0.042848291	DOWN
**LRTOMT**	11q13.4	-0.414949846	0.002578773	DOWN
**PDXDC2P-NPIPB14P**		-0.415823133	0.022656136	DOWN
**ZFP28**	19q13.43	-0.415881824	0.006176629	DOWN
**CHD6**	20q12	-0.416238666	0.002782795	DOWN
**ARHGAP33**	19q13.12	-0.416881322	0.030463666	DOWN
**CDK5RAP3**	17q21.32	-0.418238415	0.010162383	DOWN
**CCDC130**	19p13.13	-0.418663568	0.007168098	DOWN
**TBC1D13**	9q34.11	-0.419106211	0.000675867	DOWN
**ZNF568**	19q13.12	-0.419356261	0.034042865	DOWN
**SMG1P6**	16p11.2	-0.420278874	0.019282143	DOWN
**EIF3L**	22q13.1	-0.420349527	0.027338884	DOWN
**TMEM106A**	17q21.31	-0.423768997	0.043589444	DOWN
**SNORD8**	14q11.2	-0.424141285	0.030799077	DOWN
**PRIMPOL**	4q35.1	-0.424399201	8.18E-06	DOWN
**PTPRM**	18p11.23	-0.424938766	0.038403688	DOWN
**PPFIBP1**	12p11.23-p11.22	-0.425042115	0.032469893	DOWN

LMLN	3q29	-0.425090923	0.014548804	DOWN
ACVR1B	12q13.13	-0.426295511	0.000408061	DOWN
ZNF439	19p13.2	-0.426622715	0.045135296	DOWN
FUT10	8p12	-0.426876085	0.001240595	DOWN
STING1	5q31.2	-0.428384972	0.041788916	DOWN
USP35	11q14.1	-0.42854633	0.010825993	DOWN
TPM2	9p13.3	-0.42875539	0.027288375	DOWN
BICRAL	6p21.1	-0.428806139	0.017721263	DOWN
SRGAP3	3p25.3	-0.429268176	0.011413021	DOWN
RILPL1	12q24.31	-0.429514332	0.011864114	DOWN
TTC23	15q26.3	-0.430002858	0.004358597	DOWN
ADD1	4p16.3	-0.430365701	2.02E-05	DOWN
NRP1	10p11.22	-0.430677007	0.028481214	DOWN
ZNF667	19q13.43	-0.430782599	0.040148024	DOWN
ZNF433	19p13.2	-0.431067421	0.005547924	DOWN
RAPGEF4	2q31.1	-0.431414531	0.017983893	DOWN
RNF217	6q22.31	-0.431714487	0.008927195	DOWN
TUBE1	6q21	-0.432921009	0.000541153	DOWN
CCDC88B	11q13.1	-0.433154388	0.049252809	DOWN
GLYCTK	3p21.2	-0.433154671	0.000866726	DOWN
BHLHE40	3p26.1	-0.434078874	0.04214928	DOWN
ZBTB48	1p36.31	-0.434098297	0.000127373	DOWN
DPYSL2	8p21.2	-0.434590808	0.049117784	DOWN
MIB2	1p36.33	-0.434938078	0.005606656	DOWN
ZNF844	19p13.2	-0.434983745	0.005916954	DOWN
CCDC62	12q24.31	-0.435099371	0.006756464	DOWN
DMTF1	7q21.12	-0.435322017	7.79E-05	DOWN
TRMO	9q22.33	-0.435831567	6.26E-06	DOWN

**FGGY**	1p32.1	-0.435849085	0.001628277	DOWN
**RAPGEF6**	5q31.1	-0.436586123	0.012030612	DOWN
**FAM193A**	4p16.3	-0.436703243	0.026786206	DOWN
**ZNF385A**	12q13.13	-0.436889881	0.023256524	DOWN
**SECISBP2**	9q22.2	-0.438202113	0.000898512	DOWN
**SMIM14**	4p14	-0.439048406	0.038377906	DOWN
**SPSB3**	16p13.3	-0.439772492	0.00375649	DOWN
**PLA2G4A**	1q31.1	-0.439836744	0.04512622	DOWN
**KCNJ13**	2q37.1	-0.440163939	4.85E-06	DOWN
**ZNF34**	8q24.3	-0.440200411	0.013989033	DOWN
**TSPAN4**	11p15.5	-0.441694437	0.001002006	DOWN
**ORAI3**	16p11.2	-0.442571457	0.038377906	DOWN
**DIPK1A**	1p22.1	-0.443021135	0.003055325	DOWN
**ZNF14**	19p13.11	-0.44340699	3.41E-06	DOWN
**TACC1**	8p11.22	-0.443557577	0.037587827	DOWN
**DUSP10**	1q41	-0.444360579	0.020757785	DOWN
**MCTP2**	15q26.2	-0.444584954	0.003700235	DOWN
**RBM43**	2q23.3	-0.444713673	0.012315403	DOWN
**MTMR3**	22q12.2	-0.444954876	0.022761397	DOWN
**RAB6C**	2q21.1	-0.44561332	0.027480545	DOWN
**ARHGAP32**	11q24.3	-0.445858442	0.002021515	DOWN
**GALT**	9p13.3	-0.445910475	0.001867596	DOWN
**A4GALT**	22q13.2	-0.44716708	0.018366597	DOWN
**STRADB**	2q33.1	-0.447386957	0.00026433	DOWN
**DOCK11**	Xq24	-0.447444898	0.013309346	DOWN
**ARMCX1**	Xq22.1	-0.447674679	0.000420471	DOWN
**SH2D3C**	9q34.11	-0.447823902	0.014919721	DOWN
**ZNF649**	19q13.41	-0.448318546	0.00257435	DOWN

RBM47	4p14	-0.448398186	0.012223766	DOWN
SLC37A2	11q24.2	-0.448863936	0.049252809	DOWN
GPSM1	9q34.3	-0.448967356	0.000851136	DOWN
ETV5	3q27.2	-0.449693001	0.014102737	DOWN
ZNF527	19q13.12	-0.449805923	0.028198411	DOWN
FAAH	16q23.1, 1p33	-0.450439973	0.019350886	DOWN
DDX5	17q23.3	-0.451618186	0.049787353	DOWN
CDC42EP4	17q25.1	-0.451633427	0.030623446	DOWN
UBN2	7q34	-0.45170491	0.000528148	DOWN
CCDC81	11q14.2	-0.452071187	0.03403508	DOWN
ALS2CL	3p21.31	-0.452808669	0.042097285	DOWN
SUN1	7p22.3	-0.453535499	0.000474767	DOWN
NPAS2	2q11.2	-0.454399647	0.014861283	DOWN
NBR1	17q21.31	-0.45463748	0.009228947	DOWN
CIC	19q13.2	-0.454799135	0.000390859	DOWN
TMEM205	19p13.2	-0.456359291	0.04639866	DOWN
GAS8-AS1	16q24.3	-0.457537445	0.023963098	DOWN
MVB12B	9q33.3	-0.458223002	0.012561314	DOWN
HEXD	17q25.3	-0.458461815	0.000349337	DOWN
LONP2	16q12.1	-0.458806385	1.08E-06	DOWN
ZNF449	Xq26.3	-0.459086826	0.00393617	DOWN
ARSD	Xp22.33	-0.459408179	0.01253619	DOWN
DENND2A	7q34	-0.460217266	0.033449385	DOWN
PGBD1	6p22.1	-0.460227785	0.021096738	DOWN
ARHGEF17	11q13.4	-0.460348354	0.037604149	DOWN
IER5	1q25.3	-0.460355655	0.015626672	DOWN
TRMT10B	9p13.2	-0.461681274	0.044371042	DOWN
NUTM2G	9q22.33	-0.46269271	0.005547924	DOWN

**BMS1P20**	22q11.22	-0.463950053	0.047877548	DOWN
**IQCA1**	2q37.2-q37.3	-0.464222046	0.048216515	DOWN
**TMEM143**	19q13.32	-0.464249164	0.003059312	DOWN
**ZNF182**	Xp11.23	-0.464728832	1.89E-05	DOWN
**KLHL21**	1p36.31	-0.465192379	0.016443504	DOWN
**DHRS1**	14q12	-0.465338667	0.023351652	DOWN
**RNF111**	15q22.1-q22.2	-0.465660728	0.022855213	DOWN
**PLEKHM1P1**	17q24.1	-0.465795104	0.001929276	DOWN
**CCNL2**	1p36.33	-0.466301763	4.65E-05	DOWN
**ZNF346**	5q35.2	-0.466315011	0.000710997	DOWN
**PCDHGA4**	5q31	-0.466406327	0.048613256	DOWN
**INO80B-WBP1**	2p13.1	-0.46697125	0.000225034	DOWN
**PDE4A**	19p13.2	-0.467573587	0.007858663	DOWN
**ZNF225**	19q13.31	-0.467655263	0.00294708	DOWN
**WDR6**	3p21.31	-0.467678356	6.35E-06	DOWN
**POMT2**	14q24.3	-0.469217756	0.048456966	DOWN
**TMUB2**	17q21.31	-0.469345118	0.015626672	DOWN
**ZNF841**	19q13.41	-0.469378893	0.03212167	DOWN
**SPTBN5**	15q21	-0.469501876	0.04279921	DOWN
**COMMD10**	5q23.1	-0.46955372	5.11E-05	DOWN
**ITGA8**	10p13	-0.469663754	0.030116217	DOWN
**CASP10**	2q33.1	-0.470659555	0.043066156	DOWN
**NDST1**	5q33.1	-0.471546909	0.00326417	DOWN
**ZBTB49**	4p16.3	-0.47259782	0.007406684	DOWN
**ZDHHC2**	8p22	-0.472835124	0.026531864	DOWN
**MAPK8IP2**	22q13.33	-0.473019168	0.036302149	DOWN
**NBL1**	1p36.13	-0.473172586	0.007233318	DOWN
**DOP1A**	6q14.1	-0.473582764	2.34E-06	DOWN

**PICK1**	22q13.1	-0.474067002	0.009228947	DOWN
**FAM120C**	Xp11.22	-0.474284965	0.019846684	DOWN
**LMF1**	16p13.3	-0.47479508	0.002819664	DOWN
**RBBP8**	18q11.2	-0.474862655	0.007755834	DOWN
**JRK**	8q24.3	-0.474918172	0.019389862	DOWN
**POC5**	5q13.3	-0.475188259	0.009095243	DOWN
**RPA4**	Xq21.33	-0.475661025	0.007489392	DOWN
**ACRBP**	12p13.31	-0.476450821	0.046913356	DOWN
**TUBGCP6**	22q13.33	-0.477486453	0.000118153	DOWN
**AGAP13P**	10q11.22	-0.477880275	0.020880002	DOWN
**DEPDC4**	12q23.1	-0.479642561	0.015338142	DOWN
**DCAKD**	17q21.31	-0.479760562	0.007954437	DOWN
**C2CD4D-AS1**	1q21.3	-0.480742305	0.041661939	DOWN
**EZH2**	7q36.1	-0.480821287	0.044845215	DOWN
**RCBTB2**	13q14.2	-0.481090076	7.45E-05	DOWN
**OTUD3**	1p36.13	-0.481440193	0.040265919	DOWN
**EPHB6**	7q34	-0.481490759	0.013216805	DOWN
**ZNF555**	19p13.3	-0.48165728	0.000134245	DOWN
**MDM4**	1q32.1	-0.482458165	0.004623245	DOWN
**ROR1**	15q22.2, 1p31.3	-0.482977739	0.034374656	DOWN
**MAP3K4**	6q26	-0.48381307	0.013917596	DOWN
**TESK2**	1p34.1	-0.484701451	0.027091707	DOWN
**D2HGDH**	2q37.3	-0.484925048	9.13E-05	DOWN
**DDX24**	14q32.12	-0.48514393	0.003812254	DOWN
**LRRC27**	10q26.3	-0.48559	0.041271405	DOWN
**BTBD7**	14q32.12	-0.485785074	0.031580818	DOWN
**HID1**	17q25.1	-0.486451115	0.04465884	DOWN
**QPCT**	2p22.2	-0.486588107	0.04713181	DOWN

**PINK1-AS**	1p36.12	-0.486710599	0.007406684	DOWN
**SEPSECS**	4p15.2	-0.487359571	1.01E-05	DOWN
**FOXO4**	Xq13.1	-0.487471394	0.001020553	DOWN
**KLF9**	9q21.12	-0.487794019	0.039129045	DOWN
**KANSL1**	17q21.31	-0.487924429	2.51E-05	DOWN
**ARHGEF6**	Xq26.3	-0.488819396	0.035269644	DOWN
**LIMA1**	12q13.12	-0.489146229	0.034293796	DOWN
**ZNF221**	19q13.31	-0.490095762	0.046207277	DOWN
**ECI2**	6p25.2	-0.491149836	0.005555389	DOWN
**PDCL3P4**	3q12.3	-0.491727305	0.030623446	DOWN
**GUSBP14**	5q13.2	-0.492814701	0.045345283	DOWN
**SYNC**	1p35.1	-0.492983345	0.007567975	DOWN
**STAT6**	12q13.3	-0.493206036	0.013473442	DOWN
**FAM13C**	10q21.1	-0.493303157	0.045170849	DOWN
**KREMEN1**	22q12.1	-0.493414863	0.032428529	DOWN
**RETREG1**	5p15.1	-0.493926764	0.034524242	DOWN
**CPEB2**	4p15.32	-0.494236951	8.65E-06	DOWN
**RRP8**	11p15.4	-0.494821925	0.040005392	DOWN
**GATM**	15q21.1	-0.495052344	0.004634431	DOWN
**PLCD1**	3p22.2	-0.49515066	0.000838957	DOWN
**ATL1**	14q22.1	-0.495221415	0.003320851	DOWN
**JHY**	11q24.1	-0.495988031	0.004118948	DOWN
**LOC100130950**		-0.496098863	0.034387267	DOWN
**RALGPS1**	9q33.3	-0.49709337	0.00201537	DOWN
**SEMA6C**	1q21.3	-0.497784862	0.003833243	DOWN
**ACAD8**	11q25	-0.498972542	0.003954091	DOWN
**TSPYL4**	6q22.1	-0.499321345	0.000308973	DOWN
**EGFR**	7p11.2	-0.499462551	0.019389862	DOWN

**ZNF699**	19p13.2	-0.499815701	0.010642586	DOWN
**NPIPB13**	16p11.2	-0.500330892	0.000713378	DOWN
**TSSK3**	1p35.1	-0.501170272	0.022455364	DOWN
**BBIP1**	10q25.2	-0.502628728	0.000168986	DOWN
**ANKRD33B**	5p15.2	-0.502859321	0.049844954	DOWN
**NUBPL**	14q12	-0.502944203	0.043165498	DOWN
**RERE**	1p36.23	-0.503083431	1.80E-08	DOWN
**LRP5**	11q13.2	-0.503814493	0.002187988	DOWN
**FAM120B**	6q27	-0.504655582	2.20E-05	DOWN
**AUTS2**	7q11.22	-0.50473392	0.047811948	DOWN
**ANOS1**	Xp22.31	-0.504862055	0.002064197	DOWN
**ALAD**	9q32	-0.50507212	8.64E-06	DOWN
**PRSS23**	11q14.2	-0.505146302	0.007592231	DOWN
**CD99L2**	Xq28	-0.505341256	0.010266246	DOWN
**FAM122A**	9q21.11	-0.505459297	0.003776625	DOWN
**METTL15**	11p14.1	-0.506402382	0.000474767	DOWN
**MZF1**	19q13.43	-0.506907656	0.000128383	DOWN
**ARMH1**	1p34.1	-0.507103662	0.006548056	DOWN
**TMEM53**	1p34.1	-0.507301919	0.023573848	DOWN
**PDE10A**	6q27	-0.508296877	0.005150281	DOWN
**NRIP1**	21q11.2-q21.1	-0.508745156	0.000342923	DOWN
**MYSM1**	1p32.1	-0.509489922	0.030739396	DOWN
**FZD3**	7q11.23, 8p21.1	-0.509703182	0.009231964	DOWN
**AHR**	7p21.1	-0.509800064	0.04504887	DOWN
**SHROOM3**	4q21.1	-0.510526603	0.034042865	DOWN
**USP40**	2q37.1	-0.511301335	0.027695364	DOWN
**LMO4**	1p22.3	-0.51131093	0.046997991	DOWN
**NBPF15**	1q21.1	-0.512605107	0.048216515	DOWN

**IQCC**	1p35.2	-0.512981989	0.037088918	DOWN
**CD200**	3q13.2	-0.513028626	0.004950302	DOWN
**ZNF846**	19p13.2	-0.513040648	0.005577531	DOWN
**STXBP4**	17q22	-0.513047975	8.84E-06	DOWN
**IL11RA**	9p13.3	-0.513223883	0.007478839	DOWN
**SNX1**	15q22.31	-0.514484478	0.004888562	DOWN
**KAZN**	1p36.21	-0.514860182	0.002390245	DOWN
**LOC100652768**		-0.515183641	0.036160813	DOWN
**SLC2A9**	4p16.1	-0.51526401	0.010974407	DOWN
**SPATA17**	1q41	-0.5153476	0.023167575	DOWN
**OAS3**	12q24.13	-0.515758645	0.029453509	DOWN
**FCHSD1**	5q31.3	-0.516331269	0.001966979	DOWN
**TTLL5**	14q24.3, 1p36.33	-0.516808546	0.025952756	DOWN
**KLC1**	14q32.33	-0.5181901	0.000104323	DOWN
**BBS1**	11q13.2	-0.519792226	5.22E-06	DOWN
**IFI16**	1q23.1	-0.520369317	0.036858434	DOWN
**CALHM2**	10q24.33	-0.520478305	0.000714062	DOWN
**KCNQ1**	11p15.5-p15.4	-0.521075737	0.007560973	DOWN
**C19orf44**	19p13.11	-0.521609626	0.000729651	DOWN
**SARM1**	17q11.2	-0.521674602	0.005313224	DOWN
**GEMIN8**	Xp22.2	-0.522119559	0.049851545	DOWN
**LFNG**	7p22.3	-0.522585811	0.034334416	DOWN
**ZNF407**	18q23	-0.523546236	0.002170617	DOWN
**TRIOBP**	22q13.1	-0.525673299	0.009214716	DOWN
**SEMA3C**	7q21.11	-0.526140416	0.012384028	DOWN
**STIMATE-MUSTN1**	3p21.1	-0.526856355	5.13E-06	DOWN
**LTBP3**	11q13.1, 14q24.3	-0.527257635	8.99E-07	DOWN
**TRAK2**	2q33.1	-0.528041952	0.012666266	DOWN

**ITFG2**	12p13.33	-0.528241496	0.035339516	DOWN
**GAS8**	16q24.3	-0.528446831	0.000932202	DOWN
**SRSF6**	20q13.11	-0.529067496	0.000581451	DOWN
**PIK3CG**	7q22.3	-0.529332687	0.04279921	DOWN
**EXOC6B**	2p13.2	-0.530086332	0.047369377	DOWN
**ANKRD37**	4q35.1	-0.530155393	0.041152744	DOWN
**SFSWAP**	12q24.33	-0.530545273	0.016179475	DOWN
**CARD10**	22q13.1	-0.530971612	0.036002101	DOWN
**SLIT1**	10q24.1, 5q34-q35.1	-0.532373919	0.009977667	DOWN
**PTCHD4**	6p12.3	-0.532499556	0.047288661	DOWN
**GPRC5B**	16p12.3	-0.532658457	0.011202709	DOWN
**PSTK**	10q26.13	-0.532745207	0.006353716	DOWN
**SLC40A1**	2q32.2	-0.532901471	0.030798123	DOWN
**GABBR1**	6p22.1	-0.53335823	0.000353899	DOWN
**ZNF133**	20p11.23	-0.533624092	0.00021096	DOWN
**USP27X**	Xp11.23	-0.534256462	0.039125424	DOWN
**BHLHE22**	8q12.3	-0.534890692	0.020559308	DOWN
**JADE3**	Xp11.3	-0.536343749	0.043822192	DOWN
**RRN3P1**	16p12.2	-0.536353295	0.015338142	DOWN
**ZNF20**	19p13.2	-0.536653603	0.007066332	DOWN
**AMIGO3**	3p21.31	-0.536856744	0.000391934	DOWN
**SSH3**	11q13.2	-0.536881117	0.000206452	DOWN
**SNX29P2**	16p11.2	-0.53712966	0.014798149	DOWN
**VWA8**	13q14.11	-0.537327469	0.001085274	DOWN
**ARSG**	17q24.2	-0.537762435	0.00158307	DOWN
**SMG6**	17p13.3	-0.538022109	3.11E-05	DOWN
**BDH2**	4q24	-0.538024916	1.86E-07	DOWN
**ANKRA2**	5q13.2	-0.538336025	5.96E-09	DOWN

**SDHAP2**	3q29	-0.540102413	0.037990294	DOWN
**RHEB**	10q11.22, 7q36.1	-0.541173653	0.04470483	DOWN
**EVA1C**	21q22.11	-0.541565602	0.000306325	DOWN
**FTO**	16q12.2	-0.54169493	0.018455918	DOWN
**MOB3B**	9p21.2	-0.542361536	0.00684911	DOWN
**SEC14L2**	22q12.2	-0.543045085	0.000325372	DOWN
**PLK2**	5q11.2	-0.543311668	0.031198876	DOWN
**CRYBG1**	6q21	-0.543345008	0.005308202	DOWN
**CRYM-AS1**	16p12.2	-0.543562715	0.015057817	DOWN
**TSC1**	9q34	-0.544160616	0.039510067	DOWN
**LNX1**	4q12	-0.544307268	0.007940452	DOWN
**ITGA1**	5q11.2	-0.544411866	0.003030606	DOWN
**MAP3K13**	3q27.2	-0.544438845	0.012451384	DOWN
**TCF7**	5q31.1	-0.545314996	0.006506561	DOWN
**PPIL6**	6q21	-0.545884827	0.049252809	DOWN
**ACSS1**	20p11.21	-0.546030855	0.011274762	DOWN
**LPCAT4**	15q14	-0.546201367	0.000258253	DOWN
**CENPH**	5q13.2	-0.546252843	0.04596238	DOWN
**CRTAP**	3p22.3	-0.546528868	0.023727549	DOWN
**PRKCE**	2p21	-0.546635879	2.55E-06	DOWN
**PCMT1**	6q25.1	-0.547554738	0.036260371	DOWN
**METTL25**	12q21.31	-0.547646286	1.77E-09	DOWN
**CLSTN2**	3q23	-0.549887994	0.049921567	DOWN
**CLIC2**	Xq28	-0.550127075	0.019632273	DOWN
**NECTIN3**	3q13.13	-0.55054513	0.001710252	DOWN
**ZEB1**	10p11.22	-0.550721324	0.016555259	DOWN
**PCDHB9**	5q31.3	-0.551456295	0.020291593	DOWN
**AK8**	9q34.13	-0.551718857	0.017396762	DOWN

**ITGA6**	2q31.1	-0.552087356	0.042767024	DOWN
**SLC16A7**	12q14.1	-0.552160407	0.041175272	DOWN
**SNRNP70**	19q13.33	-0.552340268	1.69E-07	DOWN
**KAT6B**	10q22.2	-0.552450893	0.001807874	DOWN
**LMF1-AS1**	16p13.3	-0.552707129	0.042878138	DOWN
**TGFB1I1**	16p11.2	-0.55309728	0.004851261	DOWN
**USP30**	12q24.11	-0.553898106	0.002081002	DOWN
**BMS1P1**	10q11.22	-0.554209913	0.002558892	DOWN
**CNTN4**	3p26.3-p26.2	-0.55458814	0.036106428	DOWN
**SLC25A25**	9q34.11	-0.554607009	7.27E-06	DOWN
**PDE3A**	12p12.2	-0.554727697	0.008860302	DOWN
**ATXN7**	3p14.1	-0.554851283	0.000737162	DOWN
**ASB1**	2q37.3	-0.55490131	0.000898512	DOWN
**GBP4**	1p22.2	-0.554950821	0.018301853	DOWN
**IFT43**	14q24.3	-0.554980806	0.003833243	DOWN
**LOC100288069**		-0.555340468	0.007371231	DOWN
**GRAMD1C**	3q13.31	-0.555345825	0.0325566	DOWN
**GSTM3**	1p13.3	-0.555440799	0.021790865	DOWN
**ABTB1**	3q21.3	-0.556068033	0.000127757	DOWN
**PTPRS**	19p13.3	-0.556252896	0.000347644	DOWN
**CEACAM1**	19q13.2	-0.55640212	0.013579414	DOWN
**KIF17**	1p36.12	-0.556527152	0.035916179	DOWN
**EDNRA**	4q31.22-q31.23	-0.556802573	0.00239606	DOWN
**TMEM135**	11q14.2	-0.556910756	1.21E-05	DOWN
**LRRD1**	7q21.2	-0.557150484	0.033589104	DOWN
**IFIT3**	10q23.31	-0.558487007	0.045670523	DOWN
**RGMB**	5q15	-0.559275676	8.01E-05	DOWN
**JAM2**	21q21.3	-0.559440754	0.028220621	DOWN

**SYTL4**	Xq22.1	-0.559596404	0.004289176	DOWN
**RIBC1**	Xp11.22	-0.560154178	0.039324941	DOWN
**FAM126A**	7p15.3	-0.560183631	0.007167203	DOWN
**TTN-AS1**	2q31.2	-0.56038933	0.000220642	DOWN
**ARPC4-TTLL3**	3p25.3	-0.560509825	0.024263794	DOWN
**TFCP2L1**	2q14.2	-0.560754102	0.028041402	DOWN
**ABCA9**	17q24.2	-0.56077104	0.020110462	DOWN
**CDKN2A**	9p21.3	-0.561191966	0.048507485	DOWN
**ANKRD42**	11q14.1	-0.56134551	2.44E-05	DOWN
**CRACR2A**	12p13.32	-0.561722225	0.008715531	DOWN
**RAVER2**	1p31.3	-0.561841704	0.02879303	DOWN
**ABL1**	9q34.12	-0.562233735	0.016376396	DOWN
**EPS8L2**	11p15.5	-0.562445251	0.034245312	DOWN
**CYP2C8**	10q23.33	-0.562736185	0.01934939	DOWN
**MAP3K20-AS1**	2q31.1	-0.562911928	0.019585016	DOWN
**SPC25**	2q31.1	-0.563048079	0.022115737	DOWN
**PLEKHM1**	17q21.31	-0.563108376	0.00020054	DOWN
**GJD3**	17q21.2	-0.564364259	0.012969094	DOWN
**SHC3**	9q22.1	-0.565469683	0.039125424	DOWN
**ZNF721**	4p16.3	-0.565637835	0.004404421	DOWN
**LOC653160**		-0.566092392	0.011571168	DOWN
**ITIH2**	10p14	-0.566200624	0.048084772	DOWN
**ITGB4**	17q25.1	-0.567351613	0.007877732	DOWN
**ATP1A1-AS1**	1p13.1	-0.567565316	0.009526156	DOWN
**KIF7**	15q26.1	-0.56870408	0.000980956	DOWN
**CEP290**	12q21.32	-0.569674663	1.78E-05	DOWN
**UBE2L6**	11q12.1	-0.569691227	0.038406632	DOWN
**FGFR1**	8p11.23	-0.569852886	0.02599155	DOWN

**PLPPR2**	19p13.2	-0.570169438	0.00294708	DOWN
**DAB2**	5p13.1	-0.570210412	0.000575993	DOWN
**DLEU2L**	1p31.3	-0.570365846	0.017816664	DOWN
**REP15**	12p11.22	-0.570422231	0.006451836	DOWN
**SCARNA17**	18q21.1	-0.570976079	0.008049383	DOWN
**CADM1**	11q23.3	-0.57117742	0.0239141	DOWN
**TRIM3**	11p15.4	-0.571619235	0.000777336	DOWN
**LOC100128288**		-0.572056632	0.01504047	DOWN
**PHOSPHO2**	2q31.1	-0.57309165	0.002871091	DOWN
**TRPM6**	9q21.13	-0.573210558	0.009260791	DOWN
**H3-2**	1q21.1	-0.573287417	0.043604587	DOWN
**ADAMTS1**	21q21.3	-0.573420774	0.028291008	DOWN
**PCDHGB6**	5q31	-0.573635305	0.002182417	DOWN
**IL18RAP**	2q12.1	-0.575004547	0.047816545	DOWN
**PILRB**	7q22.1	-0.575182385	0.045170849	DOWN
**PCDHGA11**	5q31.3	-0.576878428	0.008935059	DOWN
**NUDT16**	3q22.1	-0.578690153	0.035862267	DOWN
**APC2**	19p13.3, 9q34.3	-0.579083498	0.013469676	DOWN
**TVP23C-CDRT4**	17p12	-0.579306361	0.007745696	DOWN
**SLC39A6**	18q12.2	-0.579434553	1.31E-05	DOWN
**ZNF823**	19p13.2	-0.579788961	1.84E-07	DOWN
**EIF3E**	8q23.1	-0.5800584	0.030861494	DOWN
**ARID4A**	14q23.1	-0.580583021	0.008130672	DOWN
**CFAP206**	6q15	-0.580837082	0.00520804	DOWN
**EPHA4**	2q36.1	-0.581178483	0.009255093	DOWN
**ISYNA1**	19p13.11	-0.58246307	0.022131373	DOWN
**STEAP1**	7q21.13	-0.58254737	0.005130355	DOWN
**CHRM5**	15q14	-0.583034622	0.016940642	DOWN

**ECE1-AS1**	1p36.12	-0.583673389	0.027324431	DOWN
**UVSSA**	4p16.3	-0.584575173	3.09E-05	DOWN
**CRIM1**	2p22.2	-0.584819457	0.000776018	DOWN
**CROT**	7q21.12	-0.585313757	0.000191631	DOWN
**HACD3**	15q22.31	-0.585542646	0.049844954	DOWN
**RRP7BP**	22q13.2	-0.58620306	0.003533169	DOWN
**ZNF79**	9q33.3	-0.586708764	0.027705999	DOWN
**CDH13**	16q23.3	-0.588914545	0.018436593	DOWN
**SIX5**	19q13.32	-0.589181696	0.001510834	DOWN
**FHIT**	3p14.2	-0.589647956	0.046536232	DOWN
**TRIM52**	5q35.3	-0.589705338	8.12E-05	DOWN
**PDE4DIPP2**	1p11.2	-0.589917485	0.000281083	DOWN
**SULT1A1**	16p11.2	-0.589983577	0.037286618	DOWN
**THTPA**	14q11.2	-0.590107945	2.23E-08	DOWN
**MAMLD1**	Xq28	-0.590643258	0.00294708	DOWN
**BRD8**	5q31.2	-0.590643773	0.008365163	DOWN
**PLAG1**	8q12.1	-0.590689139	0.038222962	DOWN
**RRN3P3**	16p12.2	-0.590716867	0.024263794	DOWN
**CYC1**	8q24.3	-0.590788469	0.024307496	DOWN
**RABL2A**	2q14.1	-0.591152671	0.001105039	DOWN
**TATDN1**	8q24.13	-0.591473218	0.032735937	DOWN
**ZNF799**	19p13.2	-0.591649353	1.06E-05	DOWN
**NOP53**	19q13.33	-0.591808134	0.000438263	DOWN
**ZFHX4**	8q21.13	-0.591902493	0.028889244	DOWN
**GOLGA8R**	15q13.2	-0.592375799	0.043234111	DOWN
**GUSBP17**	5q13.2	-0.592450477	0.015346951	DOWN
**SNX7**	1p21.3	-0.592858469	0.021886343	DOWN
**PSMA3**	14q23.1	-0.593484091	0.04550631	DOWN

**AGFG2**	7q22.1	-0.593840864	0.008917943	DOWN
**ZNF219**	14q11	-0.593896568	0.000883116	DOWN
**ZFP36L2**	2p21	-0.59514361	7.52E-06	DOWN
**RAB3IL1**	11q12.2-q12.3	-0.596115175	0.024429558	DOWN
**NPDC1**	9q34.3	-0.59654919	0.004128346	DOWN
**TP73-AS1**	1p36.32	-0.59668067	0.000133301	DOWN
**CRYBG3**	3q11.2	-0.596695127	0.001696688	DOWN
**ZKSCAN7**	3p21.31	-0.596916066	0.007371231	DOWN
**UBXN11**	1p36.11	-0.596981675	0.005126041	DOWN
**ZNF324**	19q13.43	-0.597340109	0.00521845	DOWN
**MPDZ**	4q12, 9p23	-0.597413673	0.000365044	DOWN
**CCNL1**	3q21.3, 3q25.31	-0.597763546	4.88E-06	DOWN
**TRIB3**	20p13	-0.597802764	0.026986762	DOWN
**FAM110A**	20p13	-0.598219632	0.042805827	DOWN
**ZNF418**	19q13.43	-0.598226896	0.018592142	DOWN
**PCLO**	7q21.11	-0.598862048	0.013037948	DOWN
**MBD5**	2q23.2	-0.599179833	0.017174747	DOWN
**SPATA25**	20q13.12	-0.599244425	0.009515092	DOWN
**LRRN1**	3p26.2, 7q22.1	-0.599617796	0.032540013	DOWN
**RBM5**	3p21.31	-0.599771259	1.53E-09	DOWN
**PCDHGB3**	5q31	-0.600121139	0.013955892	DOWN
**WNT4**	1p36.12	-0.60017337	0.007466868	DOWN
**IFT46**	11q23.3	-0.600484692	1.89E-05	DOWN
**COL16A1**	1p35.2	-0.601436122	0.001497981	DOWN
**PCDHGB4**	5q31	-0.60161562	0.001451286	DOWN
**TTC14**	3q26.33	-0.601780403	6.12E-07	DOWN
**LAGE3**	Xq28	-0.602212811	0.036154146	DOWN
**ARHGEF9**	Xq11.1	-0.6033369	0.032961091	DOWN

**REV1**	2q11.2	-0.6037873	0.000500752	DOWN
**SRSF5**	14q24	-0.603985337	0.000494881	DOWN
**PSMA3-AS1**	14q23.1	-0.604039804	0.008913249	DOWN
**H2AX**	11q23.3	-0.605105036	0.018871032	DOWN
**POLR3G**	5q14.3	-0.605470902	0.006343447	DOWN
**LY6E**	8q24.3	-0.605774363	0.005316573	DOWN
**PCDHGA9**	5q31	-0.605985057	0.000417933	DOWN
**GGPS1**	1q42.3	-0.606930312	0.035070618	DOWN
**SHPRH**	6q24.3	-0.607133982	1.50E-06	DOWN
**STEAP3**	2q14.2	-0.607234535	0.015866684	DOWN
**ZCCHC4**	4p15.2	-0.607443951	0.00303172	DOWN
**ITGA2B**	17q21.31	-0.608218274	0.049252809	DOWN
**NR3C1**	5q31.3	-0.608886697	1.10E-07	DOWN
**MBOAT2**	2p25.1	-0.609454222	0.046997991	DOWN
**PSD**	10q24.32	-0.609557295	0.046724938	DOWN
**ARL4A**	7p21.3	-0.60988131	0.000777682	DOWN
**XBP1**	22q12.1, 5q22.2	-0.610079158	0.006262179	DOWN
**CFAP54**	12q23.1	-0.610284347	0.005576913	DOWN
**ADPRM**	17p13.1	-0.610396548	0.000315193	DOWN
**LOC220729**		-0.610435062	0.007419574	DOWN
**UST**	6q25.1	-0.610598588	0.049380531	DOWN
**INKA1**	3p21.31	-0.610896302	0.001368572	DOWN
**CORIN**	4p12	-0.61098511	0.028876999	DOWN
**SLC1A5**	19q13.32	-0.611215726	0.029771834	DOWN
**L3MBTL1**	20q13.12	-0.61181698	0.000655237	DOWN
**ZNF609**	15q22.1	-0.611880651	8.54E-07	DOWN
**ZC4H2**	Xq11.2	-0.611980772	0.036438716	DOWN
**SLC35E4**	22q12.2	-0.612531059	0.046090261	DOWN

**ATP1A1**	1p13.1	-0.613164719	0.045243951	DOWN
**CEP78**	9q21.2	-0.613303141	0.036472304	DOWN
**LAMA5**	20q13.33	-0.613585445	0.000136521	DOWN
**LINC01138**	1q21.2	-0.6141637	0.025430818	DOWN
**LOC102724814**		-0.614390665	9.97E-06	DOWN
**MAGED4**	Xp11.22	-0.61458293	0.000973998	DOWN
**IFITM1**	11p15.5	-0.615297715	0.005420245	DOWN
**AFF1**	4q21.3-q22.1	-0.615454604	1.88E-06	DOWN
**SLC13A4**	7q33	-0.6156851	0.019070741	DOWN
**ACKR1**	1q23.2	-0.615875932	0.045525857	DOWN
**TEAD2**	19q13.33	-0.616169453	0.027619493	DOWN
**PDGFRB**	5q32	-0.616260376	7.20E-05	DOWN
**ANXA2P2**	9p13.3	-0.616336989	0.03287444	DOWN
**APCDD1L**	20q13.32	-0.616400301	0.005527509	DOWN
**AKR1C1**	10p15.1	-0.6168437	0.031486131	DOWN
**POU5F1B**	8q24.21	-0.617394246	0.018967701	DOWN
**PIK3AP1**	10q24.1	-0.617670404	0.04428363	DOWN
**DLK2**	6p21.1	-0.617849561	0.005205945	DOWN
**C12orf4**	12p13.32	-0.618069675	0.029848282	DOWN
**RAB30**	11q14.1	-0.618705418	0.000614	DOWN
**MYH3**	17p13.1	-0.619427362	4.78E-05	DOWN
**RAPGEF3**	12q13.11	-0.61997033	0.022761397	DOWN
**TCEAL1**	Xq22.2	-0.620053356	0.032811647	DOWN
**HIC1**	17p13.3	-0.620558921	0.010642586	DOWN
**NT5DC2**	3p21.1	-0.621112283	0.034904734	DOWN
**ZNF175**	19q13.41	-0.621633558	0.000177027	DOWN
**TLN2**	15q22.2	-0.622695173	0.041586756	DOWN
**CARMIL3**	14q11.2	-0.623172287	0.022219168	DOWN

**CCL3**	17q12	-0.623496238	0.010974407	DOWN
**ABAT**	16p13.2	-0.623698469	0.030463666	DOWN
**THEM6**	8q24.3	-0.624011193	0.036082808	DOWN
**RAB38**	11q14.2	-0.624029021	0.002133392	DOWN
**PRPF40B**	12q13.12	-0.625593857	4.91E-08	DOWN
**VWA7**	6p21.33	-0.625605771	0.020963335	DOWN
**FBXL7**	5p15.1	-0.625741688	0.027361586	DOWN
**BATF**	14q24.3	-0.626208091	0.039018575	DOWN
**VDAC1**	5q31.1	-0.626243241	0.043611874	DOWN
**CCND2**	12p13.32	-0.626647529	3.79E-05	DOWN
**TC2N**	14q32.12	-0.626758847	0.004013061	DOWN
**GTF2IRD2B**	7q11.23	-0.627454475	0.000720274	DOWN
**ATG2B**	14q32.2	-0.62824431	1.53E-09	DOWN
**MIF**	19p13.3, 1q21.3, 22q11.23	-0.62920159	0.012074217	DOWN
**NDC1**	1p32.3	-0.629306846	0.017869181	DOWN
**LINC00265**	7p14.1	-0.629356873	0.000282672	DOWN
**H2BP2**	1q21.1	-0.629778047	0.042100108	DOWN
**FOXP1**	3p13	-0.630597674	9.24E-05	DOWN
**ARFGEF1**	8q13.2	-0.630728631	0.026290387	DOWN
**ADD3**	10q25.1-q25.2	-0.6310202	2.20E-05	DOWN
**HEMK1**	3p21.31	-0.631364169	0.004544995	DOWN
**PCDHB14**	5q31.3	-0.631513106	0.000324102	DOWN
**PGM2**	4p14	-0.632003703	0.019849628	DOWN
**XIST**	Xq13.2	-0.632197815	0.000328998	DOWN
**NSG1**	4p16.3	-0.632715649	0.026531864	DOWN
**TMEM50A**	1p36.11	-0.632770309	0.047293529	DOWN
**MSR1**	8p22	-0.633004075	0.01370272	DOWN

**QRICH2**	17q25.1	-0.633047836	0.006621378	DOWN
**FAM111B**	11q12.1	-0.63327379	0.031326168	DOWN
**ALDH1L2**	12q23.3	-0.633620285	0.034958758	DOWN
**TGFB1**	19q13.2	-0.633816332	0.039323473	DOWN
**EFHC1**	6p12.2	-0.633824362	0.00791365	DOWN
**FZD7**	2q33.1	-0.633914421	0.006499867	DOWN
**EME1**	17q21.33	-0.634014847	0.037414824	DOWN
**TUBA1A**	12q13.12	-0.634678652	0.026137668	DOWN
**WDR46**	6p21.32	-0.63494037	0.044601031	DOWN
**NHLRC1**	6p22.3	-0.635131386	0.049432119	DOWN
**DLG5**	10q22.3	-0.635548301	2.60E-05	DOWN
**RPL7**	8q21.11	-0.63574787	0.023887022	DOWN
**OSTC**	4q25	-0.636349382	0.04512432	DOWN
**SMARCD3**	7q36.1	-0.636739163	2.06E-05	DOWN
**CYRIB**	8q24.21	-0.636793675	0.009775153	DOWN
**C17orf107**	17p13.2	-0.637091814	0.000232844	DOWN
**RMDN2**	2p22.2	-0.637374188	0.016017939	DOWN
**TTC21B**	2q24.3	-0.638658928	1.65E-06	DOWN
**CDK19**	6q21	-0.638689958	0.019084142	DOWN
**FILIP1**	6q14.1	-0.638703093	0.000320121	DOWN
**ALDH2**	12q24.12	-0.639088172	0.033669958	DOWN
**MUC12**	7q22.1	-0.639129592	0.002473864	DOWN
**BUB1**	2q13	-0.639847232	0.026280729	DOWN
**MKI67**	10q26.2	-0.640453339	0.030487115	DOWN
**DDX47**	12p13.1	-0.640751602	0.042767024	DOWN
**VRK1**	14q32.2	-0.641235268	0.019459517	DOWN
**MRPL12**	17q25.3	-0.641355533	0.02840473	DOWN
**MIPOL1**	14q13.3-q21.1	-0.641417296	3.03E-05	DOWN

**H2BC18**	1q21.2	-0.641746871	0.004507058	DOWN
**MPHOSPH8**	13q12.11	-0.642104254	0.001102341	DOWN
**AMY2B**	1p21.1	-0.642260142	0.008820403	DOWN
**PEX12**	17q12	-0.642740233	0.019389862	DOWN
**ALG1L9P**	11q13.4	-0.642764962	0.018334639	DOWN
**KIAA1109**	4q27	-0.643269463	0.010914455	DOWN
**HSPA4L**	4q28.1	-0.644576686	0.012200397	DOWN
**GLRA1**	5q33.1	-0.644581308	0.038403688	DOWN
**CENPV**	17p11.2	-0.644714461	0.01179198	DOWN
**DUSP1**	1q23.3, 5q35.1	-0.644823357	0.02030283	DOWN
**RCCD1**	15q26.1	-0.645142815	0.030450534	DOWN
**SDR39U1**	14q12	-0.645858386	0.000785949	DOWN
**ZNF425**	7q36.1	-0.646764165	0.000999916	DOWN
**ANKRD20A5P**	18p11.21	-0.646911581	0.015338142	DOWN
**SLC25A29**	14q32.2	-0.647148496	0.002465082	DOWN
**FBXL5**	4p15.32	-0.648209868	5.07E-08	DOWN
**AHI1**	6q23.3	-0.648346161	5.12E-08	DOWN
**NPTXR**	22q13.1	-0.648650517	0.039900022	DOWN
**NRAS**	1p13.2	-0.649095129	0.032508917	DOWN
**LOC112268219**		-0.649236684	0.024645264	DOWN
**RABEP1**	17p13.2	-0.6492467	2.95E-07	DOWN
**RIC3**	11p15.4	-0.649775068	0.030733656	DOWN
**EDARADD**	1q42.3-q43	-0.649808393	0.044150603	DOWN
**GPR39**	2q21.2	-0.650089649	0.033266322	DOWN
**DNAI4**	1p31.3	-0.650394561	4.16E-05	DOWN
**PTDSS1**	8q22.1	-0.651401368	0.030912654	DOWN
**TCF4**	10q25.2-q25.3, 18q21.2	-0.651566262	5.00E-05	DOWN
**HSF1**	8q24.3	-0.651645038	0.023963098	DOWN

**CENPBD1**	16q24.3	-0.652181558	0.032092076	DOWN
**ZDHHC11B**	5p15.33	-0.652535594	0.017741326	DOWN
**ADA2**	17q12, 22q11.1	-0.653010044	0.01374718	DOWN
**ABCA10**	17q24.3	-0.653412732	0.015089527	DOWN
**ZNF286A**	17p12	-0.653716702	0.015818533	DOWN
**TGIF2**	20q11.23	-0.65377087	0.048250097	DOWN
**ZNF607**	19q13.1	-0.653874839	0.022018473	DOWN
**SNX33**	15q24.2	-0.654060939	1.46E-06	DOWN
**DCUN1D3**	16p12.3	-0.654978893	1.20E-05	DOWN
**SRP9**	1q42.12	-0.655219161	0.038222962	DOWN
**TLR3**	4q35.1	-0.656216619	0.00082593	DOWN
**VTA1**	6q24.1-q24.2	-0.65625557	0.037033395	DOWN
**UBAP2L**	1q21.3	-0.656277712	0.031879814	DOWN
**CHRNA5**	15q25.1	-0.656376899	0.04428363	DOWN
**MRPS16**	10q22.2	-0.656776998	0.02969724	DOWN
**MIR497HG**	17p13.1	-0.656846522	0.020614771	DOWN
**ZBTB47**	3p22.1	-0.657181204	0.003065284	DOWN
**EYA1**	8q13.3	-0.657314332	0.011576013	DOWN
**NAP1L5**	4q22.1	-0.657829806	0.000742766	DOWN
**ABLIM1**	10q25.3	-0.658024388	0.028461987	DOWN
**SNORD94**	2p11.2	-0.658250303	0.002967159	DOWN
**WDR5**	9q34.2	-0.658707157	0.018969046	DOWN
**HOOK1**	1p32.1	-0.658937779	0.045279344	DOWN
**SCML1**	Xp22.13	-0.659497347	0.000269415	DOWN
**KCNJ8**	12p12.1	-0.659763689	0.000961978	DOWN
**TOP1MT**	8q24.3	-0.660251348	0.02158799	DOWN
**DLG4**	17p11.2, 17p13.1	-0.660627146	5.76E-06	DOWN
**ARRDC4**	15q26.2	-0.660891661	1.03E-05	DOWN

EMB	5q11.1	-0.661094102	0.02148325	DOWN
MOK	14q32.31	-0.662180813	0.008492234	DOWN
C21orf62	21q22.11	-0.662267738	0.010974407	DOWN
HERC6	4q22.1	-0.663199053	0.008749843	DOWN
TCF7L2	10q25.2-q25.3	-0.663226833	0.032464203	DOWN
ASTN2	9q33.1	-0.663761201	0.015365278	DOWN
KNL1	15q15.1	-0.664052924	0.030904802	DOWN
KIAA1755	20q11.23	-0.664480079	0.002044037	DOWN
COX19	7p22.3	-0.665190243	0.044392341	DOWN
CASP9	1p36.21	-0.665861581	0.001390422	DOWN
LOC440311		-0.666096683	0.00033764	DOWN
PAIP2	5q31.2	-0.666452505	0.034572699	DOWN
ADAMTS3	4q13.3	-0.667290057	0.012969094	DOWN
TTC41P	12q23.3	-0.667890721	0.01691264	DOWN
TADA2B	4p16.1	-0.667992891	0.000207105	DOWN
RHOBTB2	8p21.3	-0.668061753	1.83E-07	DOWN
APBB1	11p15.4	-0.668438721	2.39E-05	DOWN
COLCA2	11q23.1	-0.668553412	0.03807736	DOWN
P4HTM	3p21.31	-0.668872827	5.75E-05	DOWN
PRSS12	4q26	-0.669071771	0.002792814	DOWN
STAT5B	17q21.2	-0.669537178	0.000283528	DOWN
AGPAT3	21q22.3	-0.669554123	0.043650154	DOWN
GASK1B	4q32.1	-0.669997279	0.003715264	DOWN
METTL2B	7q32.1	-0.670613105	0.045484742	DOWN
RHPN2	19q13.11	-0.671219002	0.008997989	DOWN
DLC1	12q24.31, 3p22.2, 8p22	-0.671337261	0.001016929	DOWN
DZIP1L	3q22.3	-0.671571664	0.001237697	DOWN
RAD51B	14q24.1	-0.671760639	0.000144132	DOWN

**PLEKHH3**	17q21.2	-0.671792356	1.03E-05	DOWN
**ZSCAN18**	19q13.43	-0.671987012	0.000437331	DOWN
**TAB2**	6q25.1	-0.672188032	0.033955328	DOWN
**ZNF471**	19q13.43	-0.672807452	4.92E-06	DOWN
**EXOSC5**	19q13.2	-0.672976744	0.019389862	DOWN
**TUBG2**	17q21.2	-0.672991474	0.000363997	DOWN
**PARP3**	3p21.2	-0.673125005	3.20E-06	DOWN
**NMI**	17p13.3, 2q23.3	-0.673456629	0.005205945	DOWN
**HSD11B1L**	19p13.3	-0.674482801	0.011251397	DOWN
**TFAP2A-AS2**	6p24.3	-0.674868319	0.028291008	DOWN
**CAB39L**	13q14.2	-0.674961344	0.001455438	DOWN
**TAS2R10**	12p13.2	-0.675058383	0.001455438	DOWN
**PPP1CA**	11q13.2	-0.675407714	0.037734498	DOWN
**DNAJB5**	9p13.3	-0.676036699	2.40E-05	DOWN
**ZNF703**	8p11.23	-0.676103368	0.000740614	DOWN
**TPMT**	6p22.3	-0.676191326	0.044551183	DOWN
**GTF3C5**	9q34.13	-0.676794611	0.020090199	DOWN
**BZW2**	7p21.1	-0.677747356	0.012219743	DOWN
**DPH6**	15q14	-0.678835672	0.028220621	DOWN
**DOCK8-AS1**	9p24.3	-0.679758534	0.002536944	DOWN
**PWAR5**	15q11.2	-0.680243506	0.000302818	DOWN
**ANKHD1-EIF4EBP3**	5q31.3	-0.68049044	1.35E-08	DOWN
**ZNF714**	19p12	-0.681332986	0.020558159	DOWN
**BTBD3**	20p12.2	-0.682558502	0.015470305	DOWN
**FLRT3**	20p12.1	-0.683028378	0.026375509	DOWN
**SEMA7A**	15q24.1	-0.683194105	0.017478592	DOWN
**STYK1**	12p13.2	-0.683517141	0.003463293	DOWN
**SLC13A3**	20q13.12	-0.683589657	0.004957653	DOWN

**ZNF540**	19q13.13	-0.684776776	0.010726643	DOWN
**SNORD116-24**	15q11.2	-0.684925142	0.005368333	DOWN
**LRRC40**	1p31.1	-0.685095244	0.028164186	DOWN
**LMNB2**	19p13.3	-0.685496798	0.01061473	DOWN
**TMEM234**	1p35.2	-0.68566898	0.000121507	DOWN
**ARID5B**	10q21.2	-0.68637894	2.75E-06	DOWN
**ASIC3**	7q36.1	-0.686404865	0.002286949	DOWN
**C2orf81**	2p13.1	-0.686591989	0.009809893	DOWN
**PPHLN1**	12q12	-0.686755339	0.02708459	DOWN
**HLA-J**	6p22.1	-0.686994929	0.000364874	DOWN
**STARD8**	Xq13.1	-0.687303624	0.000109783	DOWN
**CFAP58**	10q25.1	-0.687398404	0.001714511	DOWN
**UBL5**	19p13.2	-0.687511231	0.036719683	DOWN
**SMPDL3B**	1p35.3	-0.687511494	0.038606304	DOWN
**MROH7**	1p32.3	-0.688249052	0.036260371	DOWN
**PARM1**	4q13.3	-0.688453835	0.001126639	DOWN
**GHRLOS**	3p25.3	-0.688690931	0.004056884	DOWN
**ZNF623**	8q24.3	-0.688792299	0.012124897	DOWN
**RHOJ**	14q23.2	-0.688864432	0.005191587	DOWN
**AFMID**	17q25.3	-0.689915475	0.010471645	DOWN
**TMEM177**	2q14.2	-0.690102144	0.029922351	DOWN
**DLL1**	6q27	-0.690443041	0.000303066	DOWN
**FOXO6**	1p34.2	-0.690549211	0.040148024	DOWN
**IFIT5**	10q23.31	-0.691397313	0.019347872	DOWN
**BVES**	6q21	-0.69147891	0.015057817	DOWN
**UPF3B**	Xq24	-0.691569349	0.010825993	DOWN
**RB1CC1**	8q11.23	-0.691862917	0.014043892	DOWN
**GLI2**	2q14.2	-0.692521946	0.001867596	DOWN

**CKAP2**	13q14.3	-0.692843635	0.005066643	DOWN
**RBM20**	10q25.2	-0.693096785	0.025776268	DOWN
**ISM1**	20p12.1	-0.693196631	0.006225014	DOWN
**PTPRK**	6q22.33	-0.693413341	0.001618657	DOWN
**ERH**	14q24.1	-0.693426752	0.023167575	DOWN
**ABCA5**	17q24.3	-0.694091719	2.83E-06	DOWN
**RBIS**	8q21.2	-0.695702666	0.030891796	DOWN
**MRS2**	6p22.3	-0.695829572	0.037517502	DOWN
**CENPC**	12q21.33, 4q13.2	-0.696030638	0.001040008	DOWN
**FIGNL2**	12q13.13	-0.696782828	0.00482739	DOWN
**ATF7-NPFF**	12q13.13	-0.697037868	1.28E-06	DOWN
**FAM86B3P**	8p23.1	-0.697279048	0.006169764	DOWN
**PSMA6**	14q13.2	-0.697297316	0.019645583	DOWN
**LOC100507373**		-0.697734957	0.042587244	DOWN
**HMGXB4**	22q12.3	-0.698116775	0.016816588	DOWN
**PCDHGB2**	5q31	-0.698415882	0.000477809	DOWN
**GTPBP4**	10p15.3	-0.700026975	0.015133209	DOWN
**C5orf63**	5q23.2	-0.700614164	0.00548256	DOWN
**ZNF833P**	19p13.2	-0.700734601	0.000315193	DOWN
**TPPP**	5p15.33	-0.700744945	0.045523934	DOWN
**FMO5**	1q21.1	-0.701296251	0.001504581	DOWN
**EPHB3**	3q27.1	-0.70135827	0.000684981	DOWN
**NXF1**	11q12.3	-0.701487205	1.11E-05	DOWN
**UBQLNL**	11p15.4	-0.701913254	0.02389265	DOWN
**MLYCD**	16q23.3	-0.70219687	1.93E-05	DOWN
**KIF16B**	20p12.1	-0.702923717	0.027096124	DOWN
**SNX21**	20q13.12	-0.703607799	1.19E-05	DOWN
**TXN**	9q31.3	-0.703941446	0.001148633	DOWN

ZNF575	19q13.31	-0.704107952	0.007745392	DOWN
SLC16A2	Xq13.2	-0.704116784	3.00E-06	DOWN
OSGIN1	16q23.3	-0.704459211	0.002175985	DOWN
HLA-A	6p22.1	-0.704814608	0.006225014	DOWN
PAK3	13q34, Xq23	-0.704911485	0.000776018	DOWN
FRY	13q13.1	-0.70501666	0.000114663	DOWN
KLHL29	2p24.1	-0.705606754	0.010818144	DOWN
LIFR	5p13.1	-0.705839432	0.000460747	DOWN
ITIH3	3p21.1	-0.70657969	0.028273073	DOWN
TRMT9B	8p22	-0.707497488	0.03114018	DOWN
MFSD3	8q24.3	-0.707513786	0.015048187	DOWN
MSX1	4p16.2	-0.70859254	0.032938772	DOWN
RAP1GDS1	4q23	-0.708969465	0.041586756	DOWN
CWC15	11q21	-0.709378829	0.037033395	DOWN
SNORA22	7q11.21	-0.709452173	0.013989033	DOWN
PALM	19p13.3	-0.709979769	0.00805746	DOWN
VPS72	1q21.3	-0.71020383	0.012043922	DOWN
VTN	17q11.2	-0.712225475	0.016625934	DOWN
TK1	17q25.3	-0.712622835	0.012202471	DOWN
SEZ6	17q11.2	-0.712739021	0.003690933	DOWN
SREK1	5q12.3	-0.713165169	8.87E-06	DOWN
SPDYA	2p23.2	-0.713331038	0.029411201	DOWN
SIGLEC9	19q13.3-q13.4	-0.713438996	0.027091707	DOWN
TMEM108	3q22.1	-0.713832272	0.012890052	DOWN
DOK6	18q22.2	-0.713994957	0.000198354	DOWN
ABCB10	1q42.13	-0.714052502	0.010248434	DOWN
MYL5	4p16.3	-0.714564626	0.037033395	DOWN
GGT7	20q11.22	-0.714597265	8.18E-06	DOWN

**PON1**	7q21.3	-0.715362849	0.019950342	DOWN
**DSG3**	18q12.1	-0.715397715	0.011122251	DOWN
**ATP6V1E1**	22q11.21	-0.716032587	0.020480228	DOWN
**MEIS3P1**	17p12	-0.716187225	4.01E-05	DOWN
**GIPR**	19q13.32	-0.716194151	0.009228947	DOWN
**BBS5**	2q31.1	-0.716304103	1.33E-08	DOWN
**PRKX**	Xp22.33	-0.716375458	0.013973437	DOWN
**TIGD5**	8q24.3	-0.716430094	0.030861494	DOWN
**DDX17**	22q13.1	-0.716649779	6.35E-05	DOWN
**OSBPL3**	7p15.3	-0.716728255	0.010485811	DOWN
**C16orf86**	16q22.1	-0.717402202	0.003537324	DOWN
**UTRN**	6q24.2	-0.717844275	0.000641519	DOWN
**MTUS1**	8p22	-0.718241258	0.000153146	DOWN
**HMGA1**	6p21.31	-0.718838115	0.001672179	DOWN
**BCL2**	18q21.33	-0.71922661	0.00026736	DOWN
**UBAC2**	13q32.3	-0.720863783	0.013274834	DOWN
**STARD4-AS1**	5q22.1	-0.721208624	0.007167203	DOWN
**SMAP1**	6q13	-0.721426184	0.041234971	DOWN
**PDCD2**	6q27	-0.721758229	0.026521885	DOWN
**B4GALT5**	20q13.13	-0.721933536	0.028294527	DOWN
**GGT5**	22q11.23	-0.722211293	8.44E-06	DOWN
**KIAA0232**	4p16.1	-0.722864059	1.90E-07	DOWN
**SEPTIN7P2**	7p12.3	-0.723087438	0.000100118	DOWN
**EIF4E2**	2q37.1	-0.723137084	0.03445143	DOWN
**SLC5A6**	2p23.3	-0.723568306	0.008865434	DOWN
**PTPN13**	4q21.3	-0.723795939	1.72E-05	DOWN
**PSMA4**	15q25.1	-0.723888217	0.01553097	DOWN
**SYNE4**	19q13.12	-0.72419298	0.022326655	DOWN

**LOC554249**		-0.724296734	0.010543674	DOWN
**EMG1**	12p13.31	-0.724585563	0.013262796	DOWN
**MSRB3**	12q14.3	-0.725213094	0.000181858	DOWN
**KDF1**	1p36.11	-0.725219755	0.027081603	DOWN
**LHFPL6**	13q13.3-q14.11	-0.725473541	0.002551024	DOWN
**CHRD**	3q27.1	-0.725548127	0.01680818	DOWN
**AK2**	1p35.1	-0.725731976	0.0351388	DOWN
**ARRDC3**	5q14.3	-0.726331143	2.27E-07	DOWN
**BCL6**	3q27.3	-0.727157375	0.001463671	DOWN
**ESAM**	11q24.2	-0.727568396	0.011031552	DOWN
**USP39**	2p11.2	-0.727587336	0.035106542	DOWN
**PDK1**	16p13.3, 2q31.1	-0.727657382	0.003176725	DOWN
**FLAD1**	1q21.3	-0.727684321	0.016415086	DOWN
**UNC79**	14q32.12	-0.728348459	0.002182417	DOWN
**POLR2J**	7q22.1	-0.728504383	0.03186548	DOWN
**ACCS**	11p11.2	-0.72863815	2.10E-06	DOWN
**HDX**	Xq21.1	-0.729665906	2.16E-05	DOWN
**ALAS1**	3p21.2	-0.730415154	0.04477032	DOWN
**GTF2IRD1**	7q11.23	-0.730637593	0.007682651	DOWN
**SPATA33**	16q24.3	-0.731310247	0.006176629	DOWN
**EDA**	Xq13.1	-0.73135629	0.024298319	DOWN
**KCNQ4**	1p34.2	-0.731718379	0.009926613	DOWN
**DNAJB6**	7q36.3	-0.732346562	0.01225794	DOWN
**PRMT5**	14q11.2, reserved	-0.732484667	0.016240025	DOWN
**ZNF687**	1q21.3	-0.732717236	0.025591442	DOWN
**PAPOLB**	7p22.1	-0.733312196	0.021664426	DOWN
**PLEKHH2**	2p21	-0.73359381	0.000331379	DOWN
**ARF1**	1q42.13	-0.734573005	0.020480228	DOWN

**LOC100996419**		-0.735447976	0.000703318	DOWN
**UQCRH**	1p33	-0.735485238	0.015317895	DOWN
**PDCD6**	5p15.33	-0.736149769	0.020110462	DOWN
**IFIT1**	10q23.31	-0.73744696	0.000242049	DOWN
**MLF2**	12p13.31	-0.737811874	0.012420247	DOWN
**SDHB**	1p36.13	-0.738326578	0.024757714	DOWN
**KITLG**	12q21.32	-0.738498241	0.001033397	DOWN
**FGF18**	5q35.1	-0.739085509	0.034270003	DOWN
**NEK11**	3q22.1	-0.739172509	2.33E-07	DOWN
**SYTL2**	11q14.1	-0.739480237	0.001631536	DOWN
**CIP2A**	3q13.13	-0.740126132	0.004789862	DOWN
**KLHDC9**	1q23.3	-0.740204834	0.048941447	DOWN
**SPDEF**	6p21.31	-0.740267699	0.04922026	DOWN
**PAPLN**	14q24.2	-0.740547813	0.00111328	DOWN
**GPAA1**	8q24.3	-0.740690276	0.013418927	DOWN
**SEC31A**	4q21.22	-0.741048164	0.044206573	DOWN
**DNAJC17**	15q15.1	-0.741602573	0.006140065	DOWN
**RP1L1**	8p23.1	-0.74225616	0.011241876	DOWN
**RTF2**	20q13.31	-0.742595758	0.037661632	DOWN
**FKBP3**	14q21.2	-0.742889114	0.028164186	DOWN
**PSMA2**	7p14.1	-0.742892094	0.01227309	DOWN
**CDK15**	2q33.1	-0.743062817	0.009879055	DOWN
**BBS2**	16q13	-0.743389075	0.002699649	DOWN
**BRSK2**	11p15.5	-0.743422834	0.035103962	DOWN
**C11orf98**	11q12.3	-0.743604994	0.025148293	DOWN
**FOXK1**	7p22.1	-0.743854037	0.039598141	DOWN
**RABIF**	1q32.1	-0.744631718	0.041610108	DOWN
**CYCS**	7p15.3	-0.744784793	0.017289046	DOWN

GPR183	13q32.3	-0.744874485	0.005401769	DOWN
CFAP20DC	3p14.2	-0.74493848	0.004626366	DOWN
TBX2	17q23.2	-0.745267302	0.009214716	DOWN
CTDNEP1	17p13.1	-0.745582356	0.030719818	DOWN
PDGFRA	4q12	-0.745857949	0.000826382	DOWN
EFNA4	1q21.3	-0.745929816	0.000356203	DOWN
PRRC2A	6p21.33	-0.746253393	0.021410319	DOWN
CCRL2	3p21.31	-0.746571699	0.02995378	DOWN
U2AF1	21q22.3	-0.747156761	0.018204007	DOWN
ZNF763	19p13.2	-0.747803626	1.77E-06	DOWN
LRPPRC	2p21	-0.748087447	0.006614758	DOWN
ZNF784	19q13.42	-0.748529814	1.54E-05	DOWN
SF3B4	1q21.2	-0.749430424	0.020551978	DOWN
HNRNPK	9q21.32	-0.749675833	0.040315948	DOWN
SIRT6	19p13.3	-0.750778159	0.030891796	DOWN
KLHDC10	7q32.2	-0.751656805	0.045579104	DOWN
CHAF1A	19p13.3	-0.751866179	0.009179027	DOWN
COBLL1	2q24.3	-0.75251309	1.51E-08	DOWN
HAT1	2q31.1	-0.752732472	0.038098062	DOWN
KIF6	6p21.2	-0.75284886	0.015667843	DOWN
LRRC17	7q22.1	-0.753275051	0.003112036	DOWN
EIF4A3	17q25.3	-0.753897399	0.010050366	DOWN
BRK1	3p25.3	-0.754295884	0.005547924	DOWN
TTLL11	9q33.2	-0.754984355	0.002182417	DOWN
SH3D19	4q31.3	-0.755011311	0.000470761	DOWN
PTCH2	1p34.1	-0.755192088	0.002279489	DOWN
CATSPER2P1	15q15.3	-0.755540277	0.000119858	DOWN
SCAPER	15q24.3	-0.756132089	0.00023383	DOWN

RFTN2	2q33.1	-0.756288296	0.000598237	DOWN
CLEC4A	12p13.31	-0.756624258	0.004765136	DOWN
STK17A	7p13	-0.756653373	0.003598619	DOWN
ATP5PF	21q21.3	-0.756867556	0.008427257	DOWN
CAT	11p13	-0.757645625	4.24E-05	DOWN
CCDC17	1p34.1	-0.758870322	0.002261478	DOWN
OR51M1	11p15.4	-0.758878344	0.032956229	DOWN
RAB12	18p11.22	-0.759216697	0.007629579	DOWN
LRRC49	15q23	-0.759433263	0.000163841	DOWN
ASMTL-AS1	Xp22.33 and Yp11.32	-0.759623512	0.004843014	DOWN
KCNAB3	17p13.1	-0.759631014	0.004160011	DOWN
CRLF1	19p12	-0.759679084	0.045535097	DOWN
TTC21A	3p22.2	-0.759896462	4.99E-07	DOWN
NOXRED1	14q24.3	-0.760025414	0.006441722	DOWN
ECE1	1p36.12	-0.760644973	3.55E-06	DOWN
FLJ20021		-0.76128317	0.020335855	DOWN
CIRBP	19p13.3	-0.762857139	0.000401553	DOWN
SNORA47	5q13.3	-0.762926022	0.009328379	DOWN
MGST3	1q24.1	-0.76308612	0.004287865	DOWN
SYBU	8q23.2	-0.763563966	0.001320942	DOWN
LRFN1	19q13.2	-0.76363555	0.006854254	DOWN
GPD1L	3p22.3	-0.764796526	0.000136498	DOWN
TMEM186	16p13.2	-0.765284524	0.016843765	DOWN
ADIRF	10q23.2	-0.765330531	0.005262038	DOWN
FNDC5	1p35.1	-0.765338407	0.00874044	DOWN
RNF165	18q21.1	-0.765826976	0.003767255	DOWN
EGR3	8p21.3	-0.766254904	0.000999916	DOWN
IDUA	4p16.3	-0.766261289	2.91E-05	DOWN

**ZNF10**	12q24.33	-0.767298448	4.84E-06	DOWN
**PTPRB**	12q15	-0.767339737	0.000498281	DOWN
**MAPK9**	5q35.3	-0.767622884	0.01822781	DOWN
**SKP2**	5p13.2	-0.76777252	0.007824902	DOWN
**TCEAL7**	Xq22.2	-0.76871423	0.038710587	DOWN
**SLCO1C1**	12p12.2	-0.769328794	0.049839648	DOWN
**SLC22A17**	14q11.2	-0.769394861	0.001801697	DOWN
**PKD2**	19q13.32, 4q22.1	-0.769537665	0.000851136	DOWN
**PLCD3**	17q21.31	-0.769568838	1.46E-05	DOWN
**SLC46A1**	17q11.2	-0.770343056	1.13E-06	DOWN
**CD40LG**	Xq26.3	-0.770593838	0.003161941	DOWN
**STXBP2**	19p13.2	-0.770706275	0.011101869	DOWN
**SLC7A9**	19q13.11	-0.77178572	0.006614758	DOWN
**APOM**	6p21.33	-0.772000956	0.009381236	DOWN
**TSPAN11**	12p11.21	-0.772682813	2.90E-05	DOWN
**AMPD2**	1p13.3	-0.772953248	0.020106142	DOWN
**NUS1**	6q22.1	-0.773319504	0.008839656	DOWN
**EEPD1**	7p14.2	-0.773358527	0.0004353	DOWN
**EDEM1**	3p26.1	-0.774150575	0.016433856	DOWN
**MAST4**	5q12.3	-0.774208385	0.001483089	DOWN
**SLC22A23**	6p25.2	-0.774510186	5.63E-06	DOWN
**PRDM11**	11p11.2	-0.776110515	1.03E-05	DOWN
**RALGAPA2**	20p11.23	-0.776430084	6.03E-08	DOWN
**STOML2**	9p13.3	-0.776654857	0.003756333	DOWN
**ADRB2**	5q32	-0.777145378	0.005038358	DOWN
**UFC1**	1q23.3	-0.777203795	0.014840144	DOWN
**EXD3**	9q34.3	-0.777901914	0.000906654	DOWN
**TMEM102**	17p13.1	-0.778203231	0.00257573	DOWN

**GRIA4**	11q22.3	-0.778259337	0.044609441	DOWN
**FAM13B**	5q31.2	-0.779142871	4.19E-06	DOWN
**PINLYP**	19q13.31	-0.779539105	0.009136738	DOWN
**SEMA4A**	1q22	-0.779560544	0.004333702	DOWN
**GGT2**	22q11.21	-0.77969919	0.04083169	DOWN
**PLEKHG4B**	5p15.33	-0.779825213	0.006854254	DOWN
**PCDHGA5**	5q31	-0.779990304	0.000260469	DOWN
**GCAT**	22q13.1	-0.780610074	0.009338896	DOWN
**YTHDC2**	5q22.2	-0.780790779	5.05E-05	DOWN
**KIAA2013**	1p36.22	-0.781286712	0.004587225	DOWN
**PSRC1**	1p13.3	-0.781656321	0.002133392	DOWN
**TTC28**	22q12.1	-0.78175846	0.00041858	DOWN
**POC1A**	3p21.2	-0.78221631	0.002512978	DOWN
**C11orf24**	11q13.2	-0.782963474	0.018519069	DOWN
**ZNF706**	8q22.3	-0.783807952	0.002945796	DOWN
**DPYD**	1p21.3	-0.784384952	8.57E-06	DOWN
**TTYH3**	7p22.3	-0.784493473	0.005606656	DOWN
**ARF6**	14q21.3	-0.78472932	0.046816769	DOWN
**AUNIP**	1p36.11	-0.785049193	0.001791908	DOWN
**ADAM20**	14q24.2	-0.785562551	2.56E-06	DOWN
**FLJ45513**		-0.785952065	0.000140449	DOWN
**ZNF83**	19q13.41	-0.786127067	1.22E-05	DOWN
**RCN2**	15q24.3	-0.78639345	0.00303172	DOWN
**UNC13D**	17q25.3	-0.786550354	0.000614722	DOWN
**OR52D1**	11p15.4	-0.78664189	0.028220621	DOWN
**MRPL36**	5p15.33	-0.788002045	0.005496213	DOWN
**SLC22A5**	5q31.1	-0.788749596	1.98E-10	DOWN
**SETBP1**	18q12.3	-0.788764685	6.99E-05	DOWN

**MSTO1**	1q22	-0.789055794	0.019664794	DOWN
**PPT2-EGFL8**	6p21.32	-0.789481874	1.40E-07	DOWN
**OVCH2**	11p15.4	-0.789636428	0.031069539	DOWN
**ZIM2**	19q13.43	-0.790076416	0.026468827	DOWN
**APBB2**	4p14-p13	-0.790434932	0.002079391	DOWN
**MMD**	17q22	-0.790549687	0.011534826	DOWN
**CCNB1IP1**	14q11.2	-0.791260225	0.003429405	DOWN
**RNF113B**	13q32.2	-0.791350045	0.001079419	DOWN
**LTV1**	6q24.2	-0.791353742	0.018495525	DOWN
**MYO15B**	17q25.1	-0.791405591	1.13E-05	DOWN
**NPC1L1**	7p13	-0.791422466	0.04279921	DOWN
**MSI1**	12q24.31	-0.79176525	0.00551226	DOWN
**TSN**	2q14.3	-0.791841105	0.015908524	DOWN
**DENND2C**	1p13.2	-0.791894756	6.09E-05	DOWN
**FAM214A**	15q21.2-q21.3	-0.791986856	1.81E-09	DOWN
**DHRS12**	13q14.3	-0.792681377	1.90E-06	DOWN
**NOS1AP**	1q23.3	-0.792856943	0.001390609	DOWN
**PHF6**	Xq26.2	-0.79288429	0.006365634	DOWN
**AARD**	8q24.11	-0.793954425	0.023016214	DOWN
**ELAVL1**	19p13.2	-0.794077309	0.034036142	DOWN
**BFSP1**	20p12.1	-0.794495317	0.004241656	DOWN
**H1-5**	6p22.1	-0.794663224	0.002707549	DOWN
**PDE8B**	5q13.3	-0.795210259	0.002380153	DOWN
**LRRC46**	17q21.32	-0.795890857	0.009896336	DOWN
**DARS2**	1q25.1	-0.796454473	0.010958458	DOWN
**CBLN3**	14q12	-0.797240199	0.005311116	DOWN
**CPS1**	2q34, 6p21.33	-0.797319762	3.43E-05	DOWN
**PCDHB15**	5q31.3	-0.797959013	0.000250488	DOWN

MRPL55	1q42.13	-0.799258796	0.013027851	DOWN
CHD4	12p13.31	-0.799657606	0.032428529	DOWN
MAP2	12q22, 2q34	-0.799678497	0.002561165	DOWN
ZNF534	19q13.41	-0.801446185	0.038041411	DOWN
SSBP1	7q34	-0.801603226	0.006621378	DOWN
YPEL4	11q12.1	-0.80191331	0.008997989	DOWN
DKK2	4q25	-0.801923548	0.008312217	DOWN
PTPRF	1p34.2	-0.802005765	0.003584594	DOWN
KLHL3	5q31.2	-0.802465452	7.77E-05	DOWN
LYPD6B	2q23.2	-0.803779764	0.04639866	DOWN
PGAP4	9q31.1	-0.803797013	0.00412276	DOWN
C1orf159	1p36.33	-0.80382877	0.005990137	DOWN
PGS1	17q25.3	-0.804104832	0.024314363	DOWN
TEX30	13q33.1	-0.80417318	0.007757607	DOWN
SLC6A8	Xq28	-0.804230441	0.001063211	DOWN
SKOR1	15q23	-0.804258604	0.040975002	DOWN
TMEM45B	11q24.3	-0.804295465	0.013955892	DOWN
PHLDB1	11q23.3	-0.805679378	6.95E-05	DOWN
PSMD13	11p15.5	-0.806743783	0.012043922	DOWN
CALR	19p13.13	-0.807232667	0.001473646	DOWN
CFAP300	11q22.1	-0.807484596	0.008912724	DOWN
NDUFA9	12p13.3	-0.807982307	0.002496025	DOWN
SLC27A3	1q21.3	-0.808413026	7.54E-07	DOWN
DNASE1L3	3p14.3	-0.809125881	0.048507485	DOWN
GALNT6	12q13.13	-0.809155915	4.31E-05	DOWN
NIFK	2q14.3	-0.80922647	0.006478184	DOWN
FYCO1	3p21.31	-0.809481774	3.38E-05	DOWN
INPP1	2q32.2	-0.810570311	6.52E-07	DOWN

**KLHL12**	1q32.1	-0.811563727	0.006132882	DOWN
**NADK2**	5p13.2	-0.812324828	0.003202467	DOWN
**RAB10**	2p23.3	-0.812504412	0.000874306	DOWN
**ZNF300P1**	5q33.1	-0.812793912	0.012936071	DOWN
**CCDC181**	1q24.2	-0.813323722	6.52E-05	DOWN
**LDHD**	16q23.1	-0.813805527	0.007345792	DOWN
**MDH2**	7q11.23	-0.814237814	0.004167112	DOWN
**ARG1**	1p35.2, 6q23.2	-0.814360025	0.001621852	DOWN
**FAM3C**	7q31.31	-0.814535683	0.003537466	DOWN
**H2BC5**	6p22.2	-0.814598162	0.003932933	DOWN
**G2E3**	14q12	-0.816109627	0.017538062	DOWN
**HSF2BP**	21q22.3	-0.816790791	0.001302649	DOWN
**P2RY4**	Xq13.1	-0.818635126	0.006199058	DOWN
**UBE2E1**	3p24.2	-0.819294958	0.012106074	DOWN
**TSPYL2**	Xp11.22	-0.820583775	0.002051807	DOWN
**PSMB2**	1p34.3	-0.821656403	0.002138199	DOWN
**FAM177A1**	14q13.2	-0.822744575	0.002727712	DOWN
**SEH1L**	18p11.21	-0.823241294	0.007460377	DOWN
**TMEM225B**	7q22.1	-0.824804624	0.047811948	DOWN
**RRP12**	10q24.1	-0.826694444	0.007813965	DOWN
**GLT8D2**	12q23.3	-0.826776936	0.000248744	DOWN
**SPCS1**	3p21.1	-0.826848809	0.006723741	DOWN
**ARL15**	5q11.2	-0.827036695	2.10E-05	DOWN
**NME5**	5q31.2	-0.827393504	0.002779965	DOWN
**SLC16A9**	10q21.2	-0.827878854	0.00393109	DOWN
**DENR**	12q24.31	-0.827894952	0.004457602	DOWN
**PRX**	19q13.2, 1q25.1	-0.828814776	0.000204849	DOWN
**ATAD1**	10q23.31	-0.829612914	0.004979345	DOWN

ARL8A	1q32.1	-0.831767151	0.013309346	DOWN
EEF1AKNMT	1q24.3	-0.83208633	0.001573693	DOWN
ZNF280D	15q21.3	-0.832152283	1.63E-10	DOWN
TEKT3	17p12	-0.832190651	0.009617102	DOWN
ARHGAP6	Xp22.2	-0.833162944	0.000572779	DOWN
CUX2	12q24.11-q24.12	-0.833804747	0.043348143	DOWN
PAXIP1	7q36.2	-0.834958514	0.015089527	DOWN
H4C5	6p22.2	-0.835155203	0.005998474	DOWN
C4orf36	4q21.3	-0.835491003	0.000868447	DOWN
DMRTA1	9p21.3	-0.835519842	0.014752235	DOWN
NDUFA6	22q13.2	-0.835676449	0.003134489	DOWN
HSD17B3	9q22.32	-0.835809844	0.01504047	DOWN
LDHAL6A	11p15.1	-0.835935605	0.009096643	DOWN
GOLGA8T	15q13.2	-0.836089608	0.001229162	DOWN
CAPN11	6p21.1	-0.836140878	0.038663	DOWN
NFYC	1p34.2	-0.837017628	0.040109765	DOWN
PLIN5	19p13.3	-0.837410519	0.044670436	DOWN
BEGAIN	14q32.2	-0.837655148	0.02376276	DOWN
SNCAIP	5q23.2	-0.837835434	4.17E-05	DOWN
KLHDC1	14q21.3	-0.837841677	0.001495308	DOWN
LOXL2	8p21.3	-0.838144045	0.000157231	DOWN
UTP18	17q21.33	-0.839348463	0.023496285	DOWN
CAPRIN1	11p13	-0.839487996	0.003116624	DOWN
DERPC	16q22.1	-0.839661545	0.012674982	DOWN
THSD1	13q14.3	-0.840237912	4.74E-06	DOWN
ZFP91	11q12.1	-0.840507936	0.010833346	DOWN
CCDC90B	11q14.1	-0.840745697	0.003656618	DOWN
AGR2	7p21.1	-0.840821207	0.041788916	DOWN

**ADIPOQ-AS1**	3q27.3	-0.842322062	0.017843139	DOWN
**LRG1**	19p13.3	-0.842484957	0.045397979	DOWN
**TAF4B**	18q11.2, 9q33.2	-0.842536621	0.000696463	DOWN
**UNKL**	16p13.3	-0.842926168	1.08E-06	DOWN
**STPG2**	4q22.3-q23	-0.843165994	0.000135474	DOWN
**BEND3**	6q21	-0.843939819	0.00024007	DOWN
**FBXO36**	2q36.3	-0.844024136	0.002797529	DOWN
**GMCL1**	2p13.3	-0.844142311	0.006886734	DOWN
**UCHL5**	1q31.2	-0.844612421	0.002551024	DOWN
**POLR3C**	1q21.1	-0.844628168	0.012972598	DOWN
**ZNF260**	19q13.12	-0.845264558	0.00521845	DOWN
**OR51I2**	11p15.4	-0.84571872	0.008247692	DOWN
**SGK2**	20q13.12, 20q13.2, 8q13.1	-0.845890874	0.01098197	DOWN
**NOC2L**	1p36.33	-0.846819821	0.002700188	DOWN
**PRELID1**	5q35.3	-0.847346566	0.009918844	DOWN
**WSB2**	12q24.23	-0.847351835	0.016296428	DOWN
**ST20**	15q25.1	-0.848534092	0.000167872	DOWN
**C1orf198**	1q42.2	-0.848701653	0.002398016	DOWN
**SCRN2**	17q21.32	-0.848744142	9.39E-05	DOWN
**HCRTR1**	1p35.2	-0.849333259	0.005994348	DOWN
**MMS22L**	6q16.1	-0.849443571	0.001033397	DOWN
**TTLL9**	20q11	-0.850671902	0.021671447	DOWN
**LRIF1**	1p13.3	-0.851472508	0.006169764	DOWN
**NXPH3**	17q21.33	-0.852115276	0.000707289	DOWN
**GUCY1A2**	11q22.3	-0.852191499	4.29E-05	DOWN
**NEIL1**	15q24.2	-0.852730744	5.07E-08	DOWN
**GM2A**	5q33.1	-0.852972998	0.004165933	DOWN

KCNJ11	11p15.1	-0.853827113	0.006952129	DOWN
UQCRFS1	19q12	-0.853961987	0.009270523	DOWN
ACSBG2	19p13.3	-0.853976737	0.017359286	DOWN
ANKRD65	1p36.33	-0.85433413	0.030045163	DOWN
CLIP3	19q13.12	-0.855297283	7.26E-05	DOWN
CACNA1H	16p13.3	-0.856010892	3.89E-07	DOWN
ASNS	7q21.3	-0.856051251	0.001116876	DOWN
CD160	1q21.1	-0.856230936	0.000851136	DOWN
ITSN1	21q22.11	-0.856935909	1.46E-07	DOWN
DTNBP1	6p22.3	-0.857388461	0.011209385	DOWN
ACSM6	10q23.33	-0.858230172	0.015338142	DOWN
RNF2	1q25.3	-0.858613256	0.003533169	DOWN
ICA1L	2q33.2	-0.85885091	4.31E-05	DOWN
OSGIN2	8q21.3	-0.859360958	0.002862363	DOWN
ZNF730	19p12	-0.859944463	0.004013061	DOWN
PRR29	17q23.3	-0.860730467	0.003059312	DOWN
CDC42SE1	1q21.3	-0.861975592	0.002520542	DOWN
ST6GALNAC1	17q25.1	-0.862669331	0.010630039	DOWN
PTPRN2	7q36.3	-0.862879868	0.001754007	DOWN
FNDC3B	3q26.31	-0.863585186	0.009440727	DOWN
ITPR1	3p26.1	-0.863960175	2.87E-05	DOWN
PRDX4	Xp22.11	-0.863967282	9.37E-05	DOWN
CRB1	1q31.3	-0.864192287	0.006012276	DOWN
NISCH	3p21.1	-0.865010515	2.89E-10	DOWN
SYNPO	5q33.1	-0.865131976	3.47E-05	DOWN
CRTC2	1q21.3	-0.865256352	0.01770189	DOWN
SHANK3	22q13.33	-0.865987477	4.50E-05	DOWN
ZNF639	3q26.33	-0.866347579	0.006943524	DOWN

**ANKRD62P1-PARP4P3**	22q11.1	-0.869634018	0.012553598	DOWN
**PLA2R1**	2q23-q24	-0.871055928	0.000123759	DOWN
**TFAM**	10q21.1	-0.871493522	0.000841394	DOWN
**THAP5**	7q31.1	-0.871701225	0.002625634	DOWN
**PYCR3**	8q24.3	-0.872406451	0.000976876	DOWN
**MCUR1**	6p23	-0.872584646	0.001422118	DOWN
**FAM131B**	7q34	-0.873346261	0.004226103	DOWN
**PCDHA2**	5q31.3	-0.874417479	0.000874498	DOWN
**PSMA5**	1p13.3	-0.874567148	0.001156223	DOWN
**VOPP1**	7p11.2	-0.875652424	0.002792617	DOWN
**PLEKHO1**	1q21.2	-0.876718803	0.002182417	DOWN
**SIRT3**	11p15.5	-0.877733711	1.95E-08	DOWN
**TGM5**	15q15.2	-0.87894236	0.000940015	DOWN
**NECAB1**	8q21.3	-0.879130845	0.002703766	DOWN
**CMAS**	12p12.1	-0.879172085	0.00068807	DOWN
**SHMT2**	12q13.3	-0.879610344	0.003190409	DOWN
**LGALS12**	11q12.3	-0.880356722	0.035625248	DOWN
**GNB5**	15q21.2	-0.881239207	3.50E-05	DOWN
**RALGAPA1**	14q13.2	-0.881864122	1.14E-06	DOWN
**PDCD10**	3q26.1	-0.882529538	0.000998793	DOWN
**FAM43A**	3q29	-0.884606749	0.001079419	DOWN
**TGFBR2**	3p24.1	-0.884958601	1.16E-07	DOWN
**SLC10A6**	4q21.3	-0.886248953	0.001627336	DOWN
**SLC7A10**	19q13.11	-0.886676183	0.025376993	DOWN
**PES1**	22q12.2	-0.887236255	0.001066829	DOWN
**SF3B6**	2p23.3	-0.887294348	0.001621852	DOWN
**RERG**	12p12.3	-0.888357352	0.000177011	DOWN

**RIPK2**	8q21.3	-0.888600619	0.001845152	DOWN
**VEGFD**	Xp22.2	-0.889305831	0.020453906	DOWN
**VMO1**	17p13.2	-0.889570915	0.042618097	DOWN
**PRSS44P**	3p21.31	-0.889708121	0.017396236	DOWN
**LRRC4B**	19q13.33	-0.892356121	0.003577995	DOWN
**HROB**	17q21.31	-0.892384999	0.000874498	DOWN
**TMEM183B**	3q25.1	-0.892993113	0.000624486	DOWN
**SUSD5**	3p22.3	-0.894782449	0.001056003	DOWN
**HAVCR1**	5q33.3	-0.895215565	0.011474683	DOWN
**PSMC6**	14q22.1	-0.895289529	0.003300717	DOWN
**LYPLA2P2**	19p13.2	-0.896345817	0.004830936	DOWN
**FBL**	19q13.2	-0.896868009	0.000776018	DOWN
**SLC35F2**	11q22.3	-0.89737055	6.47E-05	DOWN
**SSR4**	Xq28	-0.89819195	0.000594092	DOWN
**GEN1**	2p24.2	-0.898373517	0.002189843	DOWN
**PRSS16**	6p22.1	-0.898756047	0.000477037	DOWN
**SHANK2-AS3**	11q13.4	-0.899595648	0.032716815	DOWN
**CSNK2B**	6p21.33	-0.900236918	0.001156223	DOWN
**KLK10**	19q13.41	-0.901071128	0.018492155	DOWN
**GRIN1**	5q35.2, 9q34.3	-0.901460624	0.016224906	DOWN
**BOLA2-SMG1P6**	16p11.2	-0.902577272	0.002056849	DOWN
**AMY2A**	1p21.1	-0.902687951	0.006835566	DOWN
**KSR2**	12q24.22-q24.23, Xp22.12	-0.904924467	0.013588331	DOWN
**SMYD2**	1q32.3	-0.908134055	0.000580465	DOWN
**ZNF705E**	11q13.4	-0.908931954	0.000813492	DOWN
**BARD1**	2q35	-0.908977294	0.000853008	DOWN
**MRPS14**	1q25.1	-0.910070666	0.00548256	DOWN

PMM2	16p13.2	-0.910226364	0.007884052	DOWN
EYS	6q12	-0.910284597	0.002133897	DOWN
HOXA9	7p15.2	-0.911068836	0.020715494	DOWN
NBEA	13q13.3	-0.91175891	1.23E-05	DOWN
CNDP2	18q22.3	-0.911791778	0.000942	DOWN
CEACAM8	19q13.2	-0.912035699	0.049921567	DOWN
OR1Q1	9q33.2	-0.913396149	0.000575993	DOWN
ADRA1A	20p13, 8p21.2	-0.914429846	0.0116761	DOWN
ALMS1P1	2p13.1	-0.915134137	0.000215741	DOWN
XPO1	2p15	-0.915519399	0.008138785	DOWN
GNB4	3q26.33	-0.915529889	0.000389357	DOWN
KRT87P	12q13.13	-0.916090886	0.018455918	DOWN
JMJD7-PLA2G4B	15q15.1	-0.916111627	1.02E-07	DOWN
RPL30	8q22.2	-0.916520436	4.94E-05	DOWN
TIE1	1p34.2	-0.917675783	1.84E-07	DOWN
OLFML1	11p15.4	-0.918000662	2.92E-08	DOWN
PHTF1	1p13.2	-0.918027919	0.000749906	DOWN
KPNA6	1p35.2	-0.918509082	0.002871091	DOWN
C1QTNF2	5q33.3	-0.919238527	0.017200432	DOWN
WFDC2	20q13.12	-0.919648781	0.035652558	DOWN
GSK3A	19q13.2	-0.92085291	0.002782795	DOWN
MIR205HG	1q32.2	-0.922730868	0.00734572	DOWN
EIF3G	19p13.2	-0.922887126	0.00196457	DOWN
HELLS	10q23.33	-0.92336848	6.80E-05	DOWN
EIF5A	17p13.1	-0.925354007	0.000581451	DOWN
NOA1	4q12	-0.925527454	0.008598485	DOWN
PAICS	4q12	-0.925844947	2.17E-06	DOWN
EPRS1	1q41	-0.926042518	0.000948482	DOWN

NPHP1	2q13	-0.92607989	2.54E-05	DOWN
NOL6	9p13.3	-0.927284847	0.01063363	DOWN
BOP1	8q24.3	-0.927458379	1.44E-05	DOWN
IGSF22	11p15.1	-0.92748607	6.15E-05	DOWN
MRTFB	16p13.12	-0.928123855	1.12E-10	DOWN
WDR49	3q26.1	-0.928691364	0.000698825	DOWN
PRB4	12p13.2	-0.928741126	0.04683708	DOWN
ZDHHC9	Xq26.1	-0.929467604	0.003017907	DOWN
WSCD1	17p13.2	-0.930216351	0.003152605	DOWN
USP53	4q26	-0.933422697	5.07E-08	DOWN
PSMD7	16q23.1	-0.93454761	0.001941822	DOWN
DNAJB11	3q27.3	-0.934909808	0.000794606	DOWN
UTP25	1q32.2	-0.935605898	0.000942697	DOWN
DTX3	12q13.3	-0.936024432	1.51E-10	DOWN
GABRB3	15q12	-0.937198163	0.020110462	DOWN
SLC7A8	14q11.2	-0.93753324	3.97E-08	DOWN
RPL7L1	6p21.1	-0.939684574	0.000492294	DOWN
FAM228B	2p23.3	-0.939702901	1.73E-06	DOWN
OR14A2	1q44	-0.940004388	0.028461987	DOWN
MSL3	Xp22.2	-0.940145874	0.003331474	DOWN
MRPL37	1p32.3	-0.941168886	0.000534173	DOWN
IPO4	14q12	-0.941828963	0.000962159	DOWN
ZNRD2	11q13.1	-0.942332053	0.001856872	DOWN
CTPS1	1p34.2	-0.94307033	0.00010142	DOWN
EIF4H	7q11.23	-0.943330598	0.003871572	DOWN
MTHFD2	2p13.1	-0.943404232	5.19E-05	DOWN
EGFLAM	5p13.2-p13.1	-0.943682812	4.41E-05	DOWN
AHCY	20q11.22	-0.944107062	0.00033764	DOWN

GPR27	3p13	-0.944138633	0.03297811	DOWN
SFT2D2	1q24.2	-0.944267453	0.000166791	DOWN
AWAT1	Xq13.1	-0.944429425	0.022764599	DOWN
CSTF3	11p13	-0.946254707	0.001184633	DOWN
AGBL4	1p33	-0.949007028	0.035175462	DOWN
NDUFS3	11p11.2	-0.949705721	0.004933741	DOWN
SH3GL1	19p13.3	-0.952708864	0.00248846	DOWN
IRAIN	15q26.3	-0.954221006	0.038973855	DOWN
EFCAB6	22q13.2-q13.31	-0.958614767	3.12E-05	DOWN
PSMC4	19q13.11-q13.13	-0.958833356	0.000939091	DOWN
MED4	13q14.2	-0.960849809	0.005277153	DOWN
MICU2	13q12.11	-0.961032458	0.001240595	DOWN
PPID	4q32.1	-0.961495662	0.001225259	DOWN
KIF13B	8p12	-0.961956546	1.44E-05	DOWN
MAGEE2	Xq13.3	-0.963135584	0.040927472	DOWN
SPAG8	9p13.3	-0.963631179	0.006870127	DOWN
SYCP3	12q23.2	-0.963701583	4.60E-05	DOWN
PSMG3-AS1	7p22.3	-0.964255619	3.47E-05	DOWN
MSTN	2q32.2	-0.964751056	0.014437078	DOWN
TRHDE	12q21.1	-0.965385127	0.007671204	DOWN
SLC14A2	18q12.3	-0.966119576	0.005746446	DOWN
PPP4R2	3p13	-0.966288904	0.002013512	DOWN
TAPT1-AS1	4p15.32	-0.966700545	0.001762082	DOWN
ODC1	14q13.3, 2p25.1	-0.967173359	4.54E-05	DOWN
CFAP70	10q22.2	-0.970020395	1.93E-06	DOWN
CDK2AP1	12q24.31	-0.970345286	0.000172882	DOWN
NOX5	15q23	-0.971610774	0.022758885	DOWN
DET1	15q26.1	-0.97257327	5.84E-09	DOWN

**ZNF441**	19p13.2	-0.975951325	1.89E-09	DOWN
**SSU72**	1p36.33	-0.978394126	0.001085274	DOWN
**PUF60**	8q24.3	-0.979916991	0.000259982	DOWN
**TPGS2**	18q12.2	-0.980667656	0.000165542	DOWN
**XXYLT1**	3q29	-0.98085266	0.00059101	DOWN
**CAPNS2**	16q12.2	-0.981660681	0.029453509	DOWN
**MYOC**	1q24.3	-0.982031784	0.034334416	DOWN
**MTMR6**	13q12.13	-0.983098335	0.003399481	DOWN
**KY**	3q22.2	-0.984564536	0.018762567	DOWN
**DAD1**	14q11.2	-0.985654289	0.000563864	DOWN
**TUBA1B**	12q13.12	-0.985880724	0.000167247	DOWN
**KCNT1**	9q34.3	-0.986952145	0.033615913	DOWN
**LINC01550**	14q32.2	-0.988953631	0.001322079	DOWN
**ANKRD20A8P**	2q11.1	-0.989375777	0.002132282	DOWN
**RBL1**	20q11.23	-0.990282516	8.06E-05	DOWN
**GLI3**	7p14.1	-0.990456084	3.92E-09	DOWN
**PFDN6**	6p21.32	-0.990791313	9.13E-05	DOWN
**PM20D1**	1q32.1	-0.990907466	0.028164186	DOWN
**EPB41L4A-DT**	5q22.2	-0.991550135	0.000445871	DOWN
**MYO19**	17q12	-0.994487992	0.002254818	DOWN
**BCAP31**	Xq28	-0.994536956	0.000166791	DOWN
**AQP5**	12q13.12	-0.995734928	0.048339181	DOWN
**UBQLN3**	11p15.4	-0.995898478	0.002764585	DOWN
**DCT**	13q32.1	-0.996044928	0.035583449	DOWN
**KRTCAP2**	1q22	-0.997055221	5.40E-05	DOWN
**APOO**	Xp22.11	-0.999200837	2.94E-05	DOWN
**MAGI2**	7q21.11	-1.001560937	3.26E-06	DOWN
**YKT6**	7p13	-1.003181482	0.000381095	DOWN

**NFKBIZ**	3q12.3	-1.003988184	7.06E-08	DOWN
**KCNA6**	12p13.32	-1.004150614	0.006382429	DOWN
**IGSF1**	Xq25	-1.006924395	0.011538381	DOWN
**MTUS2**	13q12.3	-1.00742065	0.0036615	DOWN
**CARS2**	13q34	-1.008304881	0.002911432	DOWN
**EMC6**	17p13.2	-1.00843358	9.75E-05	DOWN
**SLC25A39**	17q21.31	-1.008449166	0.00014357	DOWN
**LRRC59**	17q21.33	-1.008694467	0.00015492	DOWN
**SYNGR2**	17q25.3	-1.008772723	3.74E-05	DOWN
**TWF1**	12q12	-1.008873216	0.000284643	DOWN
**CDC123**	10p14-p13	-1.009287716	4.89E-05	DOWN
**BAIAP2L1**	7q21.3-q22.1	-1.009437759	1.14E-05	DOWN
**LRIT3**	4q25	-1.00992452	7.52E-05	DOWN
**COPA**	1q23.2	-1.010323281	0.000166791	DOWN
**ANXA9**	1q21.3	-1.0140524	1.97E-05	DOWN
**TRIM27**	6p22.1	-1.014132452	0.000749997	DOWN
**CPEB3**	10q23.32	-1.016243987	1.22E-07	DOWN
**PSMA7**	20q13.33	-1.016353655	7.18E-05	DOWN
**POLR1G**	19q13.32	-1.016389617	3.78E-05	DOWN
**PCBP1-AS1**	2p13.3	-1.018813781	3.89E-06	DOWN
**MIR17HG**	13q31.3	-1.01961241	0.035376388	DOWN
**VCP**	9p13.3	-1.019852222	0.000448272	DOWN
**CYP4F22**	19p13.12	-1.020053263	0.021966384	DOWN
**B3GALT5**	21q22.2	-1.02022009	0.011579243	DOWN
**CENPQ**	6p12.3	-1.02241416	2.76E-05	DOWN
**TTPA**	8q12.3	-1.023319324	0.041387133	DOWN
**NTF4**	19q13.33	-1.023802495	0.006106359	DOWN
**DNAI1**	9p13.3	-1.02419684	0.040922073	DOWN

**TEX10**	9q31.1	-1.025049238	0.002324056	DOWN
**BRIX1**	5p13.2	-1.031197188	1.44E-05	DOWN
**ADAMTS19**	5q23.3	-1.031207992	0.016018629	DOWN
**AP4B1-AS1**	1p13.2	-1.03220033	0.012405778	DOWN
**CCNC**	6q16.2	-1.034133308	1.01E-05	DOWN
**CABP4**	11q13.2	-1.034916679	2.96E-05	DOWN
**CNNM1**	10q24.2	-1.034936929	0.019294465	DOWN
**PDCD2L**	19q13.11	-1.035633315	2.21E-05	DOWN
**C1orf112**	1q24.2	-1.039997592	0.000745359	DOWN
**GPR17**	2q14.3	-1.041020816	9.25E-05	DOWN
**SLC17A7**	19q13.33	-1.043394772	0.017155666	DOWN
**SPCS2**	11q13.4	-1.0435894	0.000230566	DOWN
**OR13G1**	1q44	-1.043772556	0.019729943	DOWN
**HOXA7**	7p15.2	-1.043777552	0.013161742	DOWN
**VWA3A**	16p12.2	-1.044158613	0.042767024	DOWN
**DIRAS3**	1p31.3	-1.047995646	0.004089562	DOWN
**HAP1**	14q11.2, 17q21.2, reserved	-1.048175207	0.040899791	DOWN
**SCYL1**	11q13.1, 17q12	-1.049263761	0.000965602	DOWN
**NCKAP1**	2q32.1	-1.051476616	4.14E-05	DOWN
**C19orf18**	19q13.43	-1.052921647	2.41E-05	DOWN
**CDCA4**	14q32.33	-1.053578512	6.23E-06	DOWN
**STAMBP**	2p13.1	-1.053626552	9.28E-05	DOWN
**DRICH1**	22q11.23	-1.053766973	0.002133392	DOWN
**GPIHBP1**	8q24.3	-1.053777119	0.020329665	DOWN
**TNRC18P1**	4q31.21	-1.054033857	0.002484709	DOWN
**PURG**	8p12	-1.055076185	0.001464765	DOWN
**TOR1A**	9q34.11	-1.057066902	0.000714062	DOWN

**BDKRB2**	14q32.2	-1.057218358	1.83E-05	DOWN
**TASOR2**	10p15.1	-1.057604301	5.19E-05	DOWN
**C16orf91**	16p13.3	-1.05824257	9.28E-05	DOWN
**TP53**	17p13.1	-1.058809515	7.28E-07	DOWN
**UQCRHL**	1p36.21	-1.060540343	2.70E-06	DOWN
**LOC101926935**		-1.061364493	0.0023014	DOWN
**GPRASP2**	Xq22.1	-1.064768054	6.72E-12	DOWN
**CARM1**	19p13.2	-1.065084695	0.000156362	DOWN
**CNOT11**	2q11.2	-1.066336775	0.002095867	DOWN
**NIPA2**	15q11.2	-1.067549181	0.000431643	DOWN
**GUK1**	1q42.13	-1.067694313	0.000251344	DOWN
**SYNPO2L**	10q22.2	-1.068068578	0.002677457	DOWN
**GPX7**	1p32.3	-1.069917792	0.000192873	DOWN
**WARS2-AS1**	1p12	-1.069994248	0.001040008	DOWN
**TMEM144**	4q32.1	-1.072044956	5.03E-09	DOWN
**SS18L2**	3p22.1	-1.075472819	0.000216625	DOWN
**ISOC2**	19q13.42	-1.075852151	0.000261258	DOWN
**PCK1**	20q13.31	-1.077373338	0.002170617	DOWN
**NUDT10**	Xp11.22	-1.079082203	0.002550635	DOWN
**URAHP**	16q24.3	-1.080477896	0.015163773	DOWN
**MYOZ2**	4q26	-1.080566342	0.000332291	DOWN
**FAIM2**	12q13.12	-1.080728747	0.019071842	DOWN
**MCM6**	2q21.3	-1.081503653	5.00E-07	DOWN
**MMP24-AS1-EDEM2**		-1.081513749	0.003030606	DOWN
**TREH**	11q23.3	-1.082218385	0.000282672	DOWN
**F11R**	1q23.3	-1.08250878	1.68E-07	DOWN
**POLD2**	7p13	-1.087121712	1.49E-05	DOWN
**TRARG1**	17p13.3	-1.08824402	0.016484737	DOWN

SPTY2D1	11p15.1	-1.088935918	0.000571333	DOWN
SPATS2	12q13.12	-1.089657322	7.93E-05	DOWN
NUDCD1	8q23.1	-1.089735903	5.87E-06	DOWN
APH1A	1q21.2	-1.090721925	4.33E-05	DOWN
SNORC	2q37.1	-1.091272045	0.001497981	DOWN
RFWD3	16q23.1	-1.091312721	1.45E-05	DOWN
TIPRL	1q24.2	-1.091371142	9.93E-06	DOWN
TCTEX1D4	1p34.1	-1.092403804	0.002502223	DOWN
MGARP	4q31.1	-1.093594781	0.020335855	DOWN
KLK8	19q13.41	-1.09364862	0.021380022	DOWN
CCDC170	6q25.1	-1.095249139	2.87E-06	DOWN
ISLR2	15q24.1	-1.097186732	0.000781775	DOWN
ADIPOR1	1q32.1	-1.098251217	0.000144474	DOWN
ATP1A4	1q23.2	-1.101287784	0.033650917	DOWN
LOC112267934		-1.105331355	0.019662644	DOWN
TULP2	19q13.33	-1.105501567	0.021398358	DOWN
MUSTN1	3p21.1	-1.105987346	0.037803438	DOWN
FAM110C	2p25.3	-1.106007766	0.010774843	DOWN
HCN1	5p12	-1.106030087	0.034718567	DOWN
TAC4	17q21.33	-1.106351612	0.000155164	DOWN
HYPK	15q15.3	-1.108819381	0.047745273	DOWN
RIMBP2	12q24.33	-1.109419371	0.00239606	DOWN
USH2A	1q41	-1.112495066	2.86E-05	DOWN
SCUBE1	22q13.2	-1.113721914	0.0027438	DOWN
MYO1H	12q24.11	-1.113820188	0.005444945	DOWN
ACTL6A	3q26.33	-1.11607366	2.21E-07	DOWN
FOXP4	6p21.1	-1.11665558	2.07E-06	DOWN
FAM221B	9p13.3	-1.116812277	0.004221885	DOWN

TRPC6	11q22.1	-1.117760443	6.18E-12	DOWN
MACO1	1p36.11	-1.118926961	0.0004242	DOWN
CHML	1q43	-1.122429015	1.51E-07	DOWN
CLPSL1	6p21.31	-1.12492306	0.015365278	DOWN
LOC103908605		-1.127100436	0.040476344	DOWN
OR51J1	11p15.4	-1.127626621	0.000768238	DOWN
PSME4	2p16.2	-1.129576069	2.75E-06	DOWN
RNF115	1q21.1	-1.130188173	4.98E-05	DOWN
MUSK	9q31.3	-1.130566571	8.99E-07	DOWN
C2orf72	2q37.1	-1.133109985	0.010384866	DOWN
CACNB2	10p12	-1.133664096	1.47E-07	DOWN
SLC1A7	1p32.3	-1.133775632	0.012202697	DOWN
CRB2	9q33.3	-1.136432316	5.12E-05	DOWN
JTB	1q21.3	-1.137003308	2.42E-05	DOWN
PLA2G4F	15q15.1	-1.141896268	0.007962866	DOWN
GDF10	10q11.22	-1.143063188	0.006183878	DOWN
ANKFN1	17q22	-1.144291499	0.022893547	DOWN
TMEM209	7q32.2	-1.147086038	2.66E-06	DOWN
RPSAP58	19p12	-1.147311333	2.61E-06	DOWN
HOXA6	7p15.2	-1.147343751	0.000906552	DOWN
TIMELESS	12q13.3	-1.14761258	2.77E-06	DOWN
ZNF648	1q25.3	-1.148159428	0.000673105	DOWN
SNORA78	16p13.3	-1.152473434	0.04428363	DOWN
DCAF12	9p13.3	-1.153097725	1.44E-05	DOWN
OR51B6	11p15.4	-1.153801338	0.000348024	DOWN
DNAJC1	10p12.31	-1.15628728	0.000103586	DOWN
IRX4	5p15.33	-1.161374795	0.008326917	DOWN
C1QL1	17q21.31	-1.162738367	0.049199107	DOWN

**SNX4**	3q21.2	-1.163848277	3.43E-05	DOWN
**OXSR1**	3p22.2	-1.164537498	0.000346938	DOWN
**PCYT1B**	Xp22.11	-1.164958931	0.016160915	DOWN
**TFDP1**	13q34	-1.167657488	1.10E-08	DOWN
**EFCAB12**	3q21.3	-1.168121486	0.004620223	DOWN
**PRMT1**	19q13.33	-1.170037602	2.29E-06	DOWN
**RUNDC3B**	7q21.12	-1.172357464	2.42E-06	DOWN
**EDA2R**	Xq12	-1.174063628	1.13E-06	DOWN
**SEC1P**	19q13.33	-1.174815522	7.74E-05	DOWN
**BAK1**	6p21.31	-1.176866332	5.21E-05	DOWN
**RABGGTB**	1p31.1	-1.177258535	2.41E-05	DOWN
**TBC1D9**	4q31.21	-1.177753327	6.33E-15	DOWN
**NLN**	5q12.3	-1.181741897	1.36E-06	DOWN
**RRP1B**	21q22.3	-1.184013168	2.33E-06	DOWN
**REEP1**	2p11.2	-1.185126773	0.006816267	DOWN
**TSPAN8**	12q21.1	-1.190022358	0.019245199	DOWN
**RGS7**	1q43	-1.190871633	0.014957796	DOWN
**USP50**	15q21.1	-1.191106176	0.015243859	DOWN
**SLCO1A2**	12p12.1	-1.192590312	0.032780966	DOWN
**COLCA1**	11q23.1	-1.19609901	0.000519943	DOWN
**PITX2**	4q25	-1.198208459	0.004310267	DOWN
**OR5K2**	3q11.2	-1.199810744	0.007460377	DOWN
**OPN1SW**	7q32.1	-1.201219036	0.000179847	DOWN
**NPHP3-AS1**	3q22.1	-1.206025332	0.012106074	DOWN
**TMSB10**	2p11.2	-1.208275368	4.59E-07	DOWN
**NPAS4**	11q13.2	-1.209258756	0.013041589	DOWN
**POLR1B**	2q14.1	-1.2118973	1.03E-05	DOWN
**PGP**	16p13.3, 19p13.11	-1.214849118	4.97E-05	DOWN

**DDO**	6q21	-1.21523789	5.30E-05	DOWN
**GGT3P**	22q11.21	-1.215556827	0.013955892	DOWN
**GNAI3**	1p13.3	-1.217900843	1.41E-06	DOWN
**CHRNB2**	1q21.3	-1.219317648	0.000500752	DOWN
**FOXE1**	9q22.33	-1.219862418	0.027749019	DOWN
**SORCS1**	10q25.1	-1.220661557	0.005300549	DOWN
**SRSF9**	12q24.31	-1.223456399	9.01E-06	DOWN
**GLRX3**	10q26.3	-1.230650184	9.87E-06	DOWN
**DAZAP1**	19p13.3	-1.230651244	4.54E-05	DOWN
**MTOR-AS1**	1p36.22	-1.23145286	0.035047298	DOWN
**SOX4**	6p22.3	-1.23520089	1.56E-09	DOWN
**USP17L4**	8p23.1	-1.235645901	0.025081854	DOWN
**KHDRBS2**	6q11.1	-1.236412319	0.028164186	DOWN
**RAMP2-AS1**	17q21.2	-1.2365123	0.019849628	DOWN
**IFNE**	9p21.3	-1.238738153	0.016376396	DOWN
**MEOX1**	17q21.31	-1.240345076	0.00059101	DOWN
**CALCR**	7q21.3	-1.243386897	0.005499739	DOWN
**LOC389199**		-1.243401503	0.032861956	DOWN
**LSR**	19q13.12	-1.24514748	2.57E-08	DOWN
**KRT222**	17q21.2	-1.249825943	0.002142408	DOWN
**SLC8A3**	14q24.2	-1.252473867	0.001249215	DOWN
**IZUMO4**	19p13.3	-1.253201687	0.00056711	DOWN
**NTN5**	19q13.33	-1.264486103	6.73E-05	DOWN
**RALY**	20q11.22	-1.264645977	1.33E-06	DOWN
**RIOK1**	6p24.3	-1.269772574	7.13E-08	DOWN
**VIPR2**	7q36.3	-1.271528634	0.00074775	DOWN
**PRB1**	12p13.2, 20q11.23	-1.273455554	0.026115226	DOWN
**VSIG10L2**	11q24.2	-1.276787097	0.009110988	DOWN

**OR51Q1**	11p15.4	-1.280077636	8.99E-05	DOWN
**SERPINI2**	3q26.1	-1.281367298	0.028041402	DOWN
**PRICKLE2-AS3**	3p14.1	-1.282945631	0.000132705	DOWN
**TCEAL2**	Xq22.1	-1.289420442	0.022724482	DOWN
**PKNOX2**	11q24.2	-1.297566226	0.000416413	DOWN
**MYO18B**	22q12.1	-1.297977467	0.002190193	DOWN
**MMP21**	10q26.2, 1p36.33	-1.301007198	5.32E-05	DOWN
**CADM2**	3p12.1	-1.303680975	0.008296032	DOWN
**GRIA1**	5q33.2	-1.307042986	0.004167112	DOWN
**OR5P3**	11p15.4	-1.308979049	0.003224746	DOWN
**C8orf34**	8q13.2	-1.312266874	0.000494881	DOWN
**AS3MT**	10q24.32	-1.312853585	0.003380055	DOWN
**SYPL2**	1p13.3	-1.314624052	0.026622196	DOWN
**WRNIP1**	6p25.2	-1.317136094	9.40E-09	DOWN
**ME2**	18q21.2, 22q13.31, 5q31.3	-1.317369079	5.70E-09	DOWN
**RFPL1**	22q12.2	-1.31791438	0.027524692	DOWN
**SIGLEC15**	18q21.1	-1.319818909	0.011028025	DOWN
**H1-9P**	17q21.33	-1.322241111	0.048216515	DOWN
**MYL3**	3p21.31	-1.324740062	0.017741326	DOWN
**PCP2**	19p13.2	-1.325991305	0.010301742	DOWN
**FAM83E**	19q13.33	-1.334471625	0.000196555	DOWN
**HPD**	12q24.31	-1.336841645	0.019319232	DOWN
**CHGA**	14q32.12	-1.339240357	0.007460377	DOWN
**LIMS3-LOC440895**		-1.34264517	0.009639152	DOWN
**NPY4R**	10q11.22	-1.344292456	0.029848282	DOWN
**PUS7**	7q22.3	-1.350791861	1.76E-10	DOWN
**RAB11B-AS1**	19p13.2	-1.351204823	0.027054365	DOWN

**LINC01587**	4p16.2	-1.353754531	0.048247107	DOWN
**KLHL14**	18q12.1	-1.353797843	0.001856872	DOWN
**MAILR**	8q22.3	-1.356987821	0.027459455	DOWN
**HERC2P4**	16p11.2	-1.360219631	0.006768701	DOWN
**TTC36**	11q23.3	-1.364098423	0.009096643	DOWN
**TFF3**	21q22.3	-1.365058638	0.003173947	DOWN
**PRRT4**	7q32.1	-1.370804114	0.005420666	DOWN
**SDR16C5**	8q12.1	-1.371047401	0.007489392	DOWN
**RXFP4**	1q22	-1.377775697	0.002357163	DOWN
**KLK11**	19q13.41	-1.379210051	0.004321374	DOWN
**TMPRSS6**	22q12.3	-1.390381956	9.55E-05	DOWN
**IRS4**	Xq22.3	-1.397187756	0.010650749	DOWN
**LINC00242**	6q27	-1.401709806	0.03635547	DOWN
**ZNF729**	19p12	-1.403205522	0.015498815	DOWN
**PSMG3**	7p22.3	-1.407004121	4.43E-09	DOWN
**MYZAP**	15q21.3	-1.409111161	0.005382814	DOWN
**NANOG**	12p13.31	-1.409410776	0.017734594	DOWN
**KCNH3**	12q13.12	-1.410092417	5.72E-06	DOWN
**AK7**	14q32.2	-1.411472788	1.25E-06	DOWN
**CLDN19**	1p34.2	-1.414166847	0.034470042	DOWN
**FABP5P3**	7q36.1	-1.415852771	0.035269644	DOWN
**ZACN**	17q25.1	-1.417351406	0.028554371	DOWN
**H2BC4**	6p22.2	-1.418096753	2.66E-15	DOWN
**CIDEA**	18p11.21	-1.421474229	0.000485647	DOWN
**PCP4L1**	1q23.3	-1.423688902	0.00126583	DOWN
**FOXK2**	17q25.3	-1.424019533	4.54E-11	DOWN
**CPLX1**	4p16.3	-1.42998233	0.000868447	DOWN
**NUP93**	16q13	-1.434907024	2.49E-09	DOWN

PLCXD3	5p13.1	-1.439270928	2.02E-05	DOWN
OPN3	1q43	-1.44523231	4.35E-12	DOWN
KCNA7	19q13.33	-1.44751787	0.006433438	DOWN
ALKAL2	2p25.3	-1.456477259	0.001316803	DOWN
SLC2A2	3q26.2	-1.456808098	0.033647572	DOWN
SLC7A14	3q26.2	-1.457790351	0.002642131	DOWN
TTC39A-AS1	1p32.3	-1.461713426	0.015295248	DOWN
TRHDE-AS1	12q21.1	-1.463593567	0.003011497	DOWN
TRIM58	1q44	-1.468429829	3.43E-05	DOWN
ADGB	6q24.3	-1.47398342	0.027775742	DOWN
CITED1	Xq13.1	-1.476238611	0.000657933	DOWN
DUSP15	20q11.21	-1.478168982	0.002190193	DOWN
CELP	9q34.13	-1.478920186	0.026010345	DOWN
WIF1	12q14.3	-1.480658589	0.000867452	DOWN
RPL34-DT	4q25	-1.485100959	0.003381732	DOWN
CYP4F11	19p13.12	-1.485820854	0.014474597	DOWN
ALDH3A1	17p11.2	-1.485868735	0.016710268	DOWN
PPP2R2C	4p16.1	-1.492934403	1.40E-05	DOWN
CLCNKB	1p36.13	-1.495484035	0.011410322	DOWN
LOC649352		-1.49552938	0.016816588	DOWN
ASTN1	1q25.2	-1.497436285	4.58E-05	DOWN
CGB7	19q13.33	-1.499188564	0.024969238	DOWN
FAM71A	1q32.3	-1.504003792	0.019662644	DOWN
BTNL8	5q35.3	-1.504344288	0.00026433	DOWN
NDP	Xp11.3	-1.506429524	0.004487561	DOWN
VIP	19p13.11, 6q25.2	-1.510915617	0.00874044	DOWN
UNC13C	15q21.3	-1.512511408	0.000655237	DOWN
PTPRR	12q15	-1.516195647	0.000488955	DOWN

**DPP6**	7q36.2	-1.519953289	0.002534368	DOWN
**ESPNL**	2q37.3	-1.523221063	0.000453537	DOWN
**IL1RAPL2**	Xq22.3	-1.525659069	0.00712661	DOWN
**SLC51B**	15q22.31	-1.53019258	0.000582185	DOWN
**ALG1L**	3q21.2	-1.535940628	0.004118948	DOWN
**PRSS45P**	3p21.31	-1.536656002	0.008995955	DOWN
**GLP1R**	6p21.2	-1.542003936	0.006199058	DOWN
**RAB3C**	5q11.2	-1.542033663	7.40E-06	DOWN
**LINC01545**	Xp11.3	-1.544754495	0.014484456	DOWN
**ABCC8**	11p15.1	-1.547625439	0.000408011	DOWN
**LINC00342**	2q11.1	-1.556940489	0.000390317	DOWN
**RSPO1**	1p34.3	-1.569414013	0.000109165	DOWN
**SYT14**	1q32.2	-1.576050757	3.76E-05	DOWN
**CABCOCO1**	10q21.2	-1.582228372	3.39E-05	DOWN
**CD300LG**	17q21.31	-1.588475001	0.000357215	DOWN
**OR4N4C**	15q11.2	-1.590462593	0.046580385	DOWN
**SPHKAP**	2q36.3	-1.591411231	3.50E-05	DOWN
**ABCG4**	11q23.3	-1.592724195	0.020230994	DOWN
**PCDH10**	4q28.3	-1.603189084	0.004998324	DOWN
**PAK6**	15q15.1	-1.604280179	4.54E-05	DOWN
**DACT2**	6q27	-1.605565693	0.004333702	DOWN
**NOTO**	2p13.2	-1.607010327	0.037990294	DOWN
**PRSS21**	16p13.3	-1.60800075	0.00082593	DOWN
**OR13J1**	9p13.3	-1.608374702	0.02873907	DOWN
**HDHD5-AS1**	22q11.1	-1.61210928	0.018291524	DOWN
**IFITM5**	11p15.5	-1.61602583	0.028164186	DOWN
**DDX25**	11q24.2	-1.618772672	0.027324431	DOWN
**KLHL33**	14q11.2	-1.62301028	4.74E-05	DOWN

**PTCHD3**	10p12.1	-1.624010275	0.034245312	DOWN
**ARHGAP27P2**	17q24.2	-1.625292331	0.03449473	DOWN
**TMEM272**	13q14.3	-1.626824589	0.018068429	DOWN
**OR7C1**	19p13.1	-1.628619695	0.015153372	DOWN
**LRRN4CL**	11q12.3	-1.631391593	0.000109397	DOWN
**DYNLRB2**	16q23.2	-1.632143559	0.008399642	DOWN
**CCER2**	19q13.2	-1.63296016	0.009287363	DOWN
**LOC389705**		-1.635307511	0.023167575	DOWN
**FAM155B**	Xq13.1	-1.636131984	4.31E-05	DOWN
**HBZ**	16p13.3	-1.647762385	0.031448887	DOWN
**NRM**	6p21.33	-1.65201974	1.28E-09	DOWN
**TPSG1**	16p13.3	-1.656817926	0.0004242	DOWN
**CERS3**	15q26.3	-1.657941664	1.21E-05	DOWN
**TSPEAR**	21q22.3	-1.658012365	0.010974407	DOWN
**SYCE1**	10q26.3	-1.658999052	0.00858584	DOWN
**TEX13B**	Xq22.3	-1.659950585	0.048456966	DOWN
**LINC00862**	1q32.1	-1.660734582	0.008322854	DOWN
**CA4**	17q23.1	-1.672457365	0.000352503	DOWN
**PSG1**	19q13.2, 19q13.31	-1.674568585	0.00059101	DOWN
**MLNR**	13q14.2	-1.675077342	0.024199035	DOWN
**SNORD114-3**	14q32.31	-1.675092574	0.043290662	DOWN
**F7**	13q34	-1.678677773	0.003932933	DOWN
**LINC02145**	5p15.31	-1.678721094	0.049673318	DOWN
**GREM2**	1q43	-1.681957837	0.014220066	DOWN
**ALOX12B**	17p13.1	-1.683623133	0.001135222	DOWN
**PCDH15**	10q21.1	-1.69486413	0.00375649	DOWN
**KNDC1**	10q26.3	-1.695830901	0.000609253	DOWN
**PLCZ1**	12p12.3	-1.697068143	0.003585554	DOWN

**REELD1**	4q31.22	-1.701408586	0.032699207	DOWN
**ADH7**	4q23	-1.705962062	0.049097411	DOWN
**KRT12**	17q21.2	-1.708667645	0.004008812	DOWN
**CYS1**	2p25.1	-1.708951837	0.005618726	DOWN
**LINC02875**	17q23.2	-1.709578918	0.003881357	DOWN
**CASP12**	11q22.3	-1.712594502	0.0004242	DOWN
**GLYAT**	11q12.1	-1.714242088	0.002879992	DOWN
**PRR15**	7p14.3	-1.716336937	0.016981728	DOWN
**PSG8**	19q13.2	-1.716435414	0.000333541	DOWN
**KCNJ16**	17q24.3	-1.718126943	0.00115007	DOWN
**LRRC24**	8q24.3	-1.725349719	8.71E-05	DOWN
**GRK7**	3q23	-1.729425759	0.007719219	DOWN
**OR6B1**	7q35	-1.731293223	0.006320482	DOWN
**ZSCAN1**	19q13.43	-1.73312336	0.000599896	DOWN
**OR10H1**	19p13.12	-1.734398642	0.026131667	DOWN
**CFAP52**	17p13.1	-1.73482549	0.048575057	DOWN
**CCL27**	9p13.3	-1.735561019	0.048018126	DOWN
**SPDYE8**	7q11.23	-1.739602872	0.049463832	DOWN
**CCDC178**	18q12.1	-1.740766713	0.000713536	DOWN
**PACRG**	6q26	-1.744968344	0.001218045	DOWN
**DUXB**	16q23.1	-1.747955662	0.034921993	DOWN
**SUMO1P3**	1q23.2	-1.749354447	5.64E-14	DOWN
**ASPG**	14q32.33	-1.749988866	0.000166791	DOWN
**CSMD3**	8q23.3	-1.751004505	4.46E-06	DOWN
**ANGPT4**	20p13	-1.752397238	0.009000949	DOWN
**PPEF2**	4q21.1	-1.753010558	0.024841346	DOWN
**LOC105371485**		-1.753971579	0.018519069	DOWN
**ANP32A-IT1**	15q23	-1.762624836	0.031879814	DOWN

**RLBP1**	15q26.1	-1.763632989	0.001631536	DOWN
**CLEC4G**	19p13.2	-1.765081149	0.00132843	DOWN
**CBLN4**	20q13.2	-1.769775008	0.008525896	DOWN
**ALOXE3**	17p13.1	-1.777474951	0.00303172	DOWN
**CPB1**	3q24	-1.785184556	0.001100223	DOWN
**LOC101927583**		-1.785771677	0.000932202	DOWN
**NPFFR2**	4q13.3	-1.786528311	0.032475833	DOWN
**SNX15**	11q13.1	-1.789889763	0.000794606	DOWN
**P2RX6**	22q11.21	-1.791502836	0.00541584	DOWN
**CCDC151**	19p13.2	-1.794745567	0.001139651	DOWN
**LOC145783**		-1.79671116	2.74E-06	DOWN
**IL17D**	13q12.11	-1.801796452	0.001042629	DOWN
**LOC101927045**		-1.802125044	0.020635871	DOWN
**RNASE13**	14q11.1	-1.80934258	0.001510834	DOWN
**LOC100509620**		-1.810062127	0.035482911	DOWN
**ADAMTS9-AS1**	3p14.1	-1.811001736	0.005712587	DOWN
**RBM24**	6p22.3	-1.811116063	0.001553904	DOWN
**HOXD11**	2q31.1	-1.814461501	0.011031552	DOWN
**TPSD1**	16p13.3	-1.815365026	0.004189807	DOWN
**PIR-FIGF**		-1.816816806	0.008427257	DOWN
**C20orf203**	20q11.21	-1.817079046	0.000131356	DOWN
**OTOP3**	17q25.1	-1.817473168	0.016577005	DOWN
**LOC284898**		-1.82293161	0.015264722	DOWN
**LRRC26**	9q34.3	-1.825284532	0.047393152	DOWN
**LOC100506281**		-1.827868283	0.016484737	DOWN
**PRPH**	12q13.12	-1.832200017	0.001079419	DOWN
**LOC105379143**		-1.836823294	0.023573848	DOWN
**OR2L8**	1q44	-1.837839025	0.000818748	DOWN

**OR52N5**	11p15.4	-1.848870594	0.007639366	DOWN
**GDF7**	2p24.1	-1.850931864	0.044999259	DOWN
**EPHA5**	4q13.1-q13.2	-1.85466152	0.00477149	DOWN
**DPY19L2P4**	7q21.13	-1.859365159	0.002982445	DOWN
**C4BPB**	1q32.1	-1.865078628	1.09E-05	DOWN
**GPR142**	17q25.1	-1.866358105	0.001592992	DOWN
**LOC283028**		-1.867047358	0.020476089	DOWN
**TBILA**	3q13.2	-1.868223225	0.036351133	DOWN
**XAGE1B**	Xp11.22	-1.874974975	0.04249273	DOWN
**C20orf204**	20q13.33	-1.877070559	0.00861207	DOWN
**C12orf40**	12q12	-1.879729217	0.032645778	DOWN
**PRSS43P**	3p21.31	-1.884815206	0.046997991	DOWN
**LUARIS**	7p13	-1.885610437	0.043092394	DOWN
**HAND2-AS1**	4q34.1	-1.886197975	0.002672582	DOWN
**LOC101928882**		-1.891254444	1.07E-07	DOWN
**SULT1C3**	2q12.3	-1.891483125	0.024498168	DOWN
**FAM180B**	11p11.2	-1.893031105	0.020122642	DOWN
**RAB44**	6p21.2	-1.893215633	0.011038626	DOWN
**PRAMEF34P**	1p36.21	-1.893526992	0.016202173	DOWN
**PRAMEF36P**	1p36.21	-1.893526992	0.016202173	DOWN
**OR7A5**	19p13.1	-1.894053063	0.01424763	DOWN
**KIAA1210**	Xq24	-1.896844211	1.66E-05	DOWN
**SMYD1**	2p11.2	-1.900219078	0.002145408	DOWN
**SERPINA10**	14q32.13	-1.913362107	0.002135941	DOWN
**ROPN1L**	5p15.2	-1.914325209	0.003845449	DOWN
**CYP4Z2P**	1p33	-1.914896713	1.98E-06	DOWN
**CPNE4**	3q22.1	-1.918541163	0.036692326	DOWN
**FAM182A**	20p11.1	-1.921733929	0.048148751	DOWN

**OR11H7**	14q11.2	-1.922452944	0.001116876	DOWN
**ZMAT4**	8p11.21	-1.922956163	0.012595579	DOWN
**C6orf99**	6q25.3	-1.928798895	5.07E-05	DOWN
**ADAD2**	16q24.1	-1.9322983	0.028294527	DOWN
**CYP4F3**	19p13.12	-1.933277664	0.043822192	DOWN
**LOC100240734**		-1.937455019	0.002602813	DOWN
**TP53AIP1**	11q24.3	-1.938434634	0.000114718	DOWN
**TMEM59L**	19p13.11	-1.940567324	0.000152413	DOWN
**LRRTM4**	2p12	-1.942148057	0.039129045	DOWN
**LOC100507551**		-1.94293555	0.023512667	DOWN
**RTL3**	Xq21.1	-1.947050112	0.023167575	DOWN
**TEX49**	12q13.12	-1.961598922	0.023272834	DOWN
**FABP9**	8q21.13	-1.962454195	0.04063949	DOWN
**EZR-AS1**	6q25.3	-1.962894939	0.040064792	DOWN
**SLCO1B3-SLCO1B7**	12p12.2	-1.970131047	0.045243951	DOWN
**ACSM2A**	16p12.3	-1.976154057	0.009333288	DOWN
**HS6ST3**	13q32.1	-1.976959858	0.027811491	DOWN
**TMEM198B**	12q13.2	-1.977757993	0.000832004	DOWN
**PRR25**	16p13.3	-1.977977888	0.038470694	DOWN
**GLP2R**	17p13.1	-1.979083853	0.019109366	DOWN
**ARC**	16q22.1, 8q24.3	-1.979355878	0.003399481	DOWN
**LOC101928093**		-1.984158813	0.019229334	DOWN
**RIMS4**	20q13.12	-1.98713089	0.034042865	DOWN
**SMTNL2**	17p13.2	-1.989319086	0.001240555	DOWN
**MKRN7P**	20q13.12	-1.990666613	0.004254894	DOWN
**PCDHB1**	5q31.3	-1.991013866	0.033833214	DOWN
**PPDPFL**	8q11.21	-1.991402447	0.034983107	DOWN
**OR2G2**	1q44	-1.991426484	0.030891796	DOWN

SLC6A13	12p13.33	-1.994671477	0.034148969	DOWN
DCX	Xq23	-1.995213595	0.000304203	DOWN
LOC101927752		-1.99583476	0.008133995	DOWN
WWC2-AS2	4q35.1	-1.996141245	0.006017078	DOWN
TMPRSS11E	4q13.2	-2.001064385	0.032440459	DOWN
GYPB	4q31.21	-2.00152632	0.011991215	DOWN
FRMPD2	10q11.22	-2.005880256	1.13E-05	DOWN
SEC14L6	22q12.2	-2.007894455	0.036709535	DOWN
LOC100128079		-2.012263304	0.012034071	DOWN
SYTL5	Xp11.4	-2.012545239	5.35E-06	DOWN
NR2E3	15q23	-2.012831458	4.68E-07	DOWN
HSD17B3-AS1	9q22.32	-2.013249718	0.004289176	DOWN
KLK12	19q13.41	-2.020043847	0.013586261	DOWN
ADRA1D	20p13	-2.025629729	0.000130497	DOWN
FGF20	8p22	-2.032035223	0.030798123	DOWN
BEST4	1p34.1	-2.032832157	0.000276969	DOWN
IFNA13	9p21.3	-2.041286173	0.042152376	DOWN
SP5	2q31.1	-2.042843866	0.000835452	DOWN
IL17B	5q32	-2.04327832	0.017802899	DOWN
C14orf178	14q24.3	-2.046481988	0.043668111	DOWN
OR2L5	1q44	-2.050080032	0.000969885	DOWN
LOC101929165		-2.055790596	0.048062718	DOWN
IGFBP1	7p12.3	-2.062725631	0.044582895	DOWN
LOC100130872		-2.063499043	0.036470509	DOWN
FAR2P2	2q21.1	-2.066829678	0.031811913	DOWN
SLC7A4	22q11.21	-2.067285021	0.000164009	DOWN
C9orf147	9q32	-2.070609101	0.018435297	DOWN
LOC100996643		-2.076593167	0.000315128	DOWN

**IFNA8**	9p21.3	-2.077883358	0.009543318	DOWN
**NXF2**	Xq22.1	-2.078591649	0.031378033	DOWN
**PSG3**	19q13.2	-2.080611374	2.00E-06	DOWN
**OPTC**	1q32.1	-2.084604594	0.049921567	DOWN
**RSPH6A**	19q13.3	-2.086174024	2.05E-06	DOWN
**LOC101929577**		-2.089696338	0.007925248	DOWN
**PSMD6-AS2**	3p14.1	-2.091794024	0.004094002	DOWN
**ABCC6P2**	16p13.11	-2.09698613	0.044912958	DOWN
**CACNG8**	19q13.42	-2.099754374	0.042618097	DOWN
**MMP27**	11q22.2	-2.101737476	1.98E-06	DOWN
**INSM2**	14q13.2	-2.114325469	0.030891796	DOWN
**PPBP**	4q13.3	-2.115275378	0.004807508	DOWN
**ANXA13**	8q24.13	-2.116071849	0.048365905	DOWN
**LCN10**	9q34.3	-2.120193254	4.84E-06	DOWN
**FRG1FP**	22p11.2	-2.120209991	0.000904608	DOWN
**MOBP**	3p22.1	-2.120692054	0.033191796	DOWN
**MAS1L**	6p22.1	-2.124393728	6.87E-06	DOWN
**SCGB3A1**	5q35.3	-2.125798148	0.000327326	DOWN
**CXADRP3**	18p11.21	-2.126314471	0.000164262	DOWN
**DSCAM**	21q22.2	-2.128858479	6.64E-06	DOWN
**PF4V1**	4q13.3	-2.128916989	0.025520174	DOWN
**LOC283299**		-2.131940621	0.005822848	DOWN
**NLRP10**	11p15.4	-2.13198288	0.009908243	DOWN
**PHGR1**	15q15.1	-2.134792508	0.015314639	DOWN
**C2CD4B**	15q22.2	-2.135147104	0.000653081	DOWN
**LINC01152**	17q24.3	-2.13738802	0.003367243	DOWN
**YIPF7**	4p12	-2.137840723	0.043326476	DOWN
**PHYHIP**	8p21.3	-2.13952414	1.47E-07	DOWN

KCNE1B	21p11.2	-2.139614843	0.044551183	DOWN
TP53TG3C	16p11.2	-2.145319376	0.017905624	DOWN
NLRP2B	Xp11.21	-2.148986911	0.032187599	DOWN
SLC6A10P	16p11.2	-2.150181689	8.91E-06	DOWN
LURAP1	1p34.1	-2.150227772	0.033214417	DOWN
FAM183A	1p34.2	-2.151963336	0.014032847	DOWN
PRDM7	16q24.3	-2.152872697	0.005662475	DOWN
LOC105370980		-2.153303352	0.042233743	DOWN
RHOXF1	Xq24	-2.156228903	0.009896336	DOWN
FAM41C	1p36.33	-2.156243306	0.017868951	DOWN
TPRG1-AS2	3q28	-2.156474319	0.040151164	DOWN
BEND2	Xp22.13	-2.15996936	0.032239094	DOWN
NR0B2	1p36.11	-2.16320151	0.049921567	DOWN
FAM90A7P	8p23.1	-2.165717996	2.96E-06	DOWN
EGFEM1P	3q26.2	-2.168795211	0.000281083	DOWN
SNTG1	8q11.21	-2.170579336	0.007062305	DOWN
SHOX	Xp22.33 and Yp11.32	-2.171669285	0.01116121	DOWN
RHOXF1-AS1	Xq24	-2.173041016	0.012200397	DOWN
SNORD116-27	15q11.2	-2.173671645	0.00555526	DOWN
LINC01252	12p13.2	-2.173860778	0.004747137	DOWN
ITPK1-AS1	14q32.12	-2.175975859	0.047523971	DOWN
KRT17P2	17p11.2	-2.180763279	0.000802308	DOWN
NANOS3	19p13.12	-2.185820287	0.007168098	DOWN
LINC00476	9q22.32	-2.186652029	0.043136044	DOWN
CALHM1	10q24.33	-2.187762307	0.000617268	DOWN
PROKR1	2p13.3	-2.189361328	0.010301742	DOWN
SCTR	2q14.2	-2.190541483	0.043337597	DOWN
NHLRC4	16p13.3	-2.195091108	0.000123568	DOWN

**CYP4F30P**	2q21.1	-2.195812322	0.041234971	DOWN
**DPF1**	19q13.2	-2.196224963	3.81E-05	DOWN
**AGR3**	7p21.1	-2.197374869	3.94E-07	DOWN
**LOC286437**		-2.200607073	0.049839648	DOWN
**FAM153A**	5q35.3	-2.202079014	0.006225014	DOWN
**NMUR2**	5q33.1	-2.202326055	0.04428363	DOWN
**LINC01119**	2p21	-2.208079536	0.045372636	DOWN
**CELF2-AS2**	10p14	-2.209087837	0.004283433	DOWN
**CFAP65**	2q35	-2.215630397	0.007625835	DOWN
**ITIH4-AS1**	3p21.1	-2.216318959	0.00326417	DOWN
**DLX6**	7q21.3	-2.220513613	0.022381907	DOWN
**SLC22A25**	11q12.3	-2.222089304	0.031536003	DOWN
**CBLN1**	16q12.1	-2.222660185	0.01564708	DOWN
**CLEC3A**	16q23.1	-2.223712976	0.020757785	DOWN
**TNP1**	2q35	-2.224167191	0.041234971	DOWN
**DDN-AS1**	12q13.12	-2.224838977	0.033897661	DOWN
**LOC101929583**		-2.227184365	0.014484456	DOWN
**SPEM1**	17p13.1	-2.229778413	0.028510084	DOWN
**IL13**	5q31.1	-2.229891795	0.024253235	DOWN
**GPR101**	Xq26.3	-2.231091375	0.04946423	DOWN
**FAM153B**	5q35.2	-2.232634009	0.012200397	DOWN
**HSD3B1**	1p12	-2.233497343	0.000165879	DOWN
**BTNL3**	5q35.3	-2.23683145	0.001801697	DOWN
**UNC45B**	17q12	-2.237098695	0.039789563	DOWN
**PSG6**	19q13.31	-2.239582694	0.001867596	DOWN
**DANT2**	Xq23	-2.242274988	0.049332117	DOWN
**PGA4**	11q12.2	-2.244256893	0.005601251	DOWN
**SYT9**	11p15.4	-2.247102969	1.19E-09	DOWN

**SUPT20HL2**	Xp22.11	-2.247132417	0.020687709	DOWN
**SCGB1C2**	17p13.3	-2.247583453	0.018815808	DOWN
**LINC02691**	14q32.33	-2.248782255	0.018967701	DOWN
**OR2W3**	1q44	-2.249685996	0.000166791	DOWN
**NKAIN3**	8q12.3	-2.249856393	0.039700609	DOWN
**ELFN2**	22q13.1	-2.25027626	1.62E-05	DOWN
**MAB21L3**	1p13.1	-2.250760713	0.001123695	DOWN
**LOC105376917**		-2.25216276	0.004118948	DOWN
**KCNE1**	21q22.12	-2.252303256	0.009093988	DOWN
**MARCHF11**	5p15.1	-2.253546853	0.015089527	DOWN
**PCSK2**	20p12.1	-2.254578415	0.04331147	DOWN
**REN**	17p13.1, 1q32.1	-2.256506264	0.002740682	DOWN
**LOC105379807**		-2.258457626	0.028658741	DOWN
**PENK**	8q12.1	-2.258928459	1.33E-06	DOWN
**MMEL1**	1p36.32	-2.259797184	0.000130643	DOWN
**WFDC6**	20q13.12	-2.260529614	0.01680818	DOWN
**CALML6**	1p36.33	-2.260853647	0.000777682	DOWN
**MAP1LC3C**	1q43	-2.263441634	0.000351386	DOWN
**RTP3**	3p21.31	-2.264998243	0.047745273	DOWN
**LINC01948**	5q11.2	-2.267984355	0.002377416	DOWN
**EEF1AKMT4-ECE2**	3q27.1	-2.269039751	0.023101572	DOWN
**TRIM50**	7q11.23	-2.270578005	7.90E-05	DOWN
**CGB2**	19q13.33	-2.277248021	0.009228947	DOWN
**RNF183**	9q32	-2.283335462	0.013269469	DOWN
**C5orf49**	5p15.31	-2.283676196	0.018264227	DOWN
**LINC01963**	2q35	-2.284825373	0.039243733	DOWN
**MSMB**	10q11.22	-2.285786084	0.009767462	DOWN

**LHFPL4**	3p25.3, 7q22.2-q22.3, 7q31	-2.29575168	0.000513282	DOWN
**MUC7**	4q13.3	-2.296404224	0.012660964	DOWN
**HAPLN4**	19p13.11	-2.298484314	0.00087997	DOWN
**LOC339260**		-2.304017851	0.018455918	DOWN
**SLCO1B7**	12p12.2	-2.309948289	0.033391538	DOWN
**LOC100506869**		-2.312403346	0.03171888	DOWN
**SYNE1-AS1**	6q25.2	-2.312663442	0.03114018	DOWN
**ZAR1**	4p11	-2.312989727	0.040309731	DOWN
**LINC00266-3**	6p25.3	-2.3142055	0.031332558	DOWN
**LOC100506639**		-2.318583977	0.035937784	DOWN
**SUGT1P1**	9p13.3	-2.319722111	8.12E-05	DOWN
**PSG4**	19q13.31	-2.319759138	2.41E-06	DOWN
**OTC**	Xp11.4	-2.323998599	0.006960457	DOWN
**LARGE-AS1**	22q12.3	-2.324696611	0.001791908	DOWN
**DDX11L10**	16p13.3	-2.324740768	0.000247525	DOWN
**TCTE1**	6p21.1	-2.32583924	0.000225456	DOWN
**MORN5**	9q33.2	-2.326074921	0.017417116	DOWN
**SYNPR**	3p14.2	-2.328445647	0.015295248	DOWN
**PPP3R2**	9q31.1	-2.332779115	0.010058022	DOWN
**PNLIPRP3**	10q25.3	-2.335012308	0.043736152	DOWN
**LOC112267881**		-2.336652855	0.020851072	DOWN
**VCX**	Xp22.31	-2.339590051	0.000108273	DOWN
**SCGB1D2**	11q12.3	-2.340717893	1.09E-07	DOWN
**RNF225**	19q13.43	-2.3409111	0.004851261	DOWN
**KTN1-AS1**	14q22.3	-2.34091483	0.007102607	DOWN
**TRDN**	6q22.31	-2.345401097	1.36E-06	DOWN
**SPRNP1**	10q26.3	-2.345849359	0.009211179	DOWN

**LRP4-AS1**	11p11.2	-2.350121433	1.32E-06	DOWN
**LINC01750**	1p13.2	-2.354670899	0.008890856	DOWN
**PSG7**	19q13.31	-2.357728039	0.000114493	DOWN
**HAO2**	1p12	-2.357975308	0.000135232	DOWN
**HOTAIRM1**	7p15.2	-2.363119705	0.00673854	DOWN
**VCX3B**	Xp22.31	-2.363516506	0.000276769	DOWN
**FAM217A**	6p25.2	-2.36895728	0.003833886	DOWN
**CRISP2**	6p12.3	-2.370652354	0.026491713	DOWN
**ASPDH**	19q13.33	-2.371455124	0.01074624	DOWN
**ANKRD2**	10q24.2	-2.372453574	0.008419913	DOWN
**DMRTC1**	Xq13.1	-2.373009787	0.000195226	DOWN
**SOWAHA**	5q31.1	-2.375073245	0.002051217	DOWN
**LUZP2**	11p14.3	-2.377805277	1.43E-08	DOWN
**TMEM35A**	Xq22.1	-2.377866868	0.02591687	DOWN
**LINC00923**	15q26.2	-2.377943805	0.006618094	DOWN
**ACP4**	19q13.33	-2.381100826	0.000123169	DOWN
**KCNK3**	2p23.3	-2.382884492	1.89E-05	DOWN
**MIR499A**	20q11.22	-2.385289966	0.03287444	DOWN
**USP17L20**	4p16.1	-2.394286458	0.007788241	DOWN
**OR10A6**	11p15.4	-2.398271691	1.45E-05	DOWN
**SHISA3**	4p13	-2.40034693	0.003577995	DOWN
**CA7**	16q22.1	-2.409850585	0.027612169	DOWN
**FAM170A**	5q23.1	-2.419165094	0.016162371	DOWN
**INSC**	11p15.2	-2.419892493	0.003127233	DOWN
**ANKRD18B**	9p13.3	-2.421585675	0.001026231	DOWN
**DSG1-AS1**	18q12.1	-2.424266422	0.03348928	DOWN
**LOC100130172**		-2.42508577	0.000393062	DOWN
**LOC101929762**		-2.425655734	0.029857713	DOWN

**HBE1**	11p15.4	-2.425695476	0.012558216	DOWN
**HMGCS2**	1p12	-2.426230313	3.00E-08	DOWN
**LOC730668**		-2.428064882	0.000924452	DOWN
**FBXO40**	3q13.33	-2.428589691	0.02745541	DOWN
**TCERG1L**	10q26.3	-2.433679717	0.005309617	DOWN
**FGG**	4q32.1	-2.436322833	0.031112493	DOWN
**OPN4**	10q23.2	-2.438243363	0.028566541	DOWN
**GNG12-AS1**	1p31.3	-2.438719813	0.002312246	DOWN
**CPA1**	7q32.2	-2.441805481	0.00303172	DOWN
**CRISP3**	6p12.3	-2.44280266	0.012219743	DOWN
**VN1R2**	19q13.42	-2.445878148	0.012134981	DOWN
**FSCB**	14q21.2	-2.454532467	0.020937938	DOWN
**CCDC177**	14q24.1	-2.457471077	0.015429273	DOWN
**LOC401463**		-2.457492777	0.016330027	DOWN
**MEF2C-AS2**	5q14.3	-2.457787351	1.97E-05	DOWN
**MT1JP**	16q13	-2.45795366	0.002092496	DOWN
**LIX1L-AS1**	1q21.1	-2.461689526	0.019950342	DOWN
**OR13H1**	Xq26.2	-2.466566741	0.022498551	DOWN
**OR5K3**	3q11.2	-2.467909399	0.01915108	DOWN
**DUSP26**	2q37.3, 8p12	-2.470213427	0.025231277	DOWN
**G6PC2**	2q31.1	-2.473823904	0.026364086	DOWN
**BEST3**	12q15	-2.474515841	0.017709578	DOWN
**EFCAB1**	8q11.21	-2.476801481	4.79E-07	DOWN
**DHRS2**	14q11.2	-2.478692749	7.81E-06	DOWN
**SPAG6**	10p12.2	-2.479620569	1.02E-07	DOWN
**LPA**	6q25.3-q26	-2.487752214	0.000435911	DOWN
**WDR72**	15q21.3	-2.490138849	8.80E-06	DOWN
**GAL3ST1**	22q12.2	-2.490689073	6.15E-07	DOWN

**PLD5**	1q43	-2.490844854	2.77E-10	DOWN
**NXF5**	Xq22.1	-2.493708626	0.012972598	DOWN
**TARID**	6q23.2	-2.497320064	0.009996661	DOWN
**NRSN1**	6p22.3	-2.499371486	0.010344541	DOWN
**DBX2**	12q12	-2.5008194	2.85E-07	DOWN
**XKR4**	8q12.1	-2.501800359	0.00036999	DOWN
**SPINK9**	5q32	-2.502008716	0.020656325	DOWN
**SLC13A2**	17q11.2	-2.505562508	2.27E-06	DOWN
**KANSL1L-AS1**	2q34	-2.506385172	0.022714627	DOWN
**LINC00639**	14q21.1	-2.507603482	0.009211179	DOWN
**SLC25A48**	5q31.1	-2.508828187	0.00921093	DOWN
**LOC100130075**		-2.509360364	0.01934939	DOWN
**LIPF**	10q23.31	-2.513184726	0.024422649	DOWN
**DNMBP-AS1**	10q24.2	-2.519179797	0.022343356	DOWN
**PAGE2B**	Xp11.21	-2.519576274	0.023101572	DOWN
**SCN2B**	11q23.3	-2.522407623	0.000133301	DOWN
**LOC650226**		-2.525391166	0.001349125	DOWN
**GCK**	11q13.1, 7p13	-2.526269338	0.005499739	DOWN
**MAG**	19q13.1, 19q13.32	-2.527393227	0.021701236	DOWN
**FAM181B**	11q14.1	-2.527882584	0.003537466	DOWN
**FAM25C**	10q11.22	-2.528609378	0.011022112	DOWN
**SMR3B**	4q13.3	-2.531787714	0.009660674	DOWN
**CELA2B**	1p36.21	-2.533887908	0.000625512	DOWN
**CNTN4-AS1**	3p26.2	-2.534059421	0.012853945	DOWN
**DEFB108B**	11q13.4	-2.535908118	0.007234872	DOWN
**DLGAP2**	8p23.3	-2.537173512	4.76E-05	DOWN
**FIGLA**	2p13.3	-2.537763311	3.08E-05	DOWN
**USP17L11**	4p16.1	-2.539975762	0.02156636	DOWN

**HEPACAM2**	7q21.2	-2.542579149	3.22E-06	DOWN
**FAM133A**	Xq21.32	-2.548043577	0.000101826	DOWN
**OR7D2**	19p13.2	-2.549956516	0.020122262	DOWN
**TSBP1-AS1**	6p21.32	-2.553174492	0.000166791	DOWN
**SLITRK3**	3q26.1	-2.55699839	0.003190409	DOWN
**OR8B3**	11q24.2	-2.558412653	0.019437336	DOWN
**HBG2**	11p15.4	-2.559525849	0.000995006	DOWN
**ISL1**	5q11.1	-2.559904729	0.016202173	DOWN
**TMEM225**	11q24.1	-2.560085937	0.007674295	DOWN
**MORC1**	3q13.13	-2.562156629	0.01880533	DOWN
**OR8D4**	11q24.1	-2.562647563	0.007745696	DOWN
**B3GALT1**	2q24.3	-2.563265122	1.96E-08	DOWN
**CERNA1**	15q21.2	-2.569011679	9.61E-06	DOWN
**ZP2**	16p12.3-p12.2	-2.57183212	0.012813226	DOWN
**ZPBP**	7p12.2	-2.574245213	0.020754157	DOWN
**TFDP3**	Xq26.2	-2.576223592	1.22E-05	DOWN
**CYP2C9**	10q23.33	-2.577852373	0.002660452	DOWN
**OR4M2**	15q11.2	-2.588320001	0.018563203	DOWN
**CYP2G1P**	19q13.2	-2.593557586	0.004694012	DOWN
**GNRH2**	20p13	-2.594504354	0.00412276	DOWN
**VENTXP7**	3p24.3	-2.594637094	0.001054667	DOWN
**PF4**	4q13.3	-2.595376788	0.021120158	DOWN
**LOC100130373**		-2.59685835	0.005330857	DOWN
**NPTX1**	17q25.3	-2.596905969	8.23E-05	DOWN
**GJB1**	Xq13.1	-2.597066786	0.009260139	DOWN
**SDR9C7**	12q13.3	-2.599613886	0.015057817	DOWN
**AMHR2**	12q13.13	-2.60050787	0.017816664	DOWN
**CLEC4GP1**	19p13.2	-2.605577152	0.010958458	DOWN

OR2T4	1q44	-2.607946589	0.011224551	DOWN
ARPP21	3p22.3	-2.610376067	0.017691627	DOWN
SMIM34A	21q22.12	-2.613139697	0.006723741	DOWN
CFAP99	4p16.3	-2.617491418	0.005574378	DOWN
LMX1A	1q23.3	-2.620729038	0.004452303	DOWN
FCN2	9q34.3	-2.624726554	0.000344308	DOWN
MYHAS	17p13.1	-2.625512389	0.000100541	DOWN
SNORD114-1	14q32.31	-2.629073529	0.012969094	DOWN
DPEP3	16q22.1	-2.635681401	0.000777682	DOWN
CTRB1	16q23.1	-2.639233539	0.000898512	DOWN
MYOZ3	5q33.1	-2.640742634	0.002345542	DOWN
LOC100289230		-2.643294806	0.009559214	DOWN
C14orf180	14q32.33	-2.6434006	0.003749717	DOWN
LOC105370792		-2.644411538	0.017035716	DOWN
BOLA3-AS1	2p13.1	-2.647345382	0.004236089	DOWN
UGT2B4	4q13.3	-2.647750389	0.002795602	DOWN
LOC105377924		-2.650569922	0.00239606	DOWN
CRP	1q23.2, 20q13.2	-2.65366659	0.007629579	DOWN
KRT37	17q21.2	-2.654487029	0.002792814	DOWN
PPFIA2-AS1	12q21.31	-2.655016439	0.002871091	DOWN
DPY19L1P2	7p14.3	-2.656515327	0.016013252	DOWN
VWC2	7p12.2	-2.658463807	0.014202991	DOWN
SCGN	6p22.2	-2.662849039	0.014096373	DOWN
BEX1	Xq22.1	-2.664564401	0.002972169	DOWN
LOC101929227		-2.665582431	0.011847607	DOWN
MAGEA8	Xq28	-2.668102954	3.79E-07	DOWN
GOLGA8EP	15q11.2	-2.672661083	0.016415086	DOWN
SNORD114-14	14q32.31	-2.673632157	0.014561301	DOWN

**MCCD1**	6p21.33	-2.675439281	0.013118368	DOWN
**PNMA5**	Xq28	-2.676770184	0.002189462	DOWN
**EDN3**	20q13.32	-2.68117214	3.04E-08	DOWN
**LINC02256**	15q13.3	-2.682653924	0.004935329	DOWN
**KCNA4**	11p14.1	-2.684172378	0.009187347	DOWN
**AGXT**	2q37.3	-2.684416814	0.000361759	DOWN
**MRTFA-AS1**	22q13.1	-2.684685998	0.007719219	DOWN
**LOC105376526**		-2.685993434	0.012200397	DOWN
**GLRA4**	Xq22.2	-2.687341239	0.002864719	DOWN
**VCX3A**	Xp22.31	-2.695093685	0.007697798	DOWN
**GLB1L3**	11q25	-2.695860018	7.26E-05	DOWN
**WNT8A**	5q31.2	-2.695911562	0.003429027	DOWN
**DACH2**	Xq21.2	-2.696661825	0.005543616	DOWN
**SLC17A3**	6p22.2	-2.698120943	0.013579414	DOWN
**CEACAM20**	19q13.31	-2.69982592	0.012193519	DOWN
**SHISA9**	16p13.12	-2.700181419	1.42E-09	DOWN
**FRMPD4**	Xp22.2	-2.700878791	2.71E-06	DOWN
**ABCC13**	21q11.2	-2.703363187	0.006746122	DOWN
**UGT2B11**	4q13.2, 4q13.3	-2.711417648	0.003447596	DOWN
**SPATA31D5P**	9q21.32	-2.713435029	0.002133897	DOWN
**STMND1**	6p22.3	-2.717897922	1.50E-06	DOWN
**CCDC70**	13q14.3	-2.71831915	0.008839656	DOWN
**CROCC2**	2q37.3	-2.727844639	0.000410434	DOWN
**PAK5**	15q15.1, 20p12.2	-2.728194626	9.95E-10	DOWN
**FOXN1**	17q11.2	-2.730675274	0.009211179	DOWN
**ZNF33BP1**	10p11.21	-2.730997802	0.010308178	DOWN
**PRR29-AS1**	17q23.3	-2.734413677	0.003767255	DOWN
**FAM66B**	8p23.1	-2.735991917	0.001951338	DOWN

LOC107986192		-2.736094546	0.002393782	DOWN
PGLYRP2	19p13.12	-2.737483163	0.001793975	DOWN
FAM187B	19q13.12	-2.737934764	0.009228947	DOWN
NEUROD2	17q12	-2.73922045	0.005645368	DOWN
SPINK13	5q32	-2.74572501	0.005679385	DOWN
NR5A1	9q33.3	-2.746154859	0.00624152	DOWN
SERPINA9	14q32.13	-2.74673249	0.006607557	DOWN
IFNA1	9p21.3	-2.752642883	0.015721075	DOWN
LOC401021		-2.756029742	0.001150042	DOWN
SLC15A5	12p12.3	-2.762085756	2.23E-08	DOWN
LOC388436		-2.766566141	0.009328379	DOWN
LOC79999		-2.766566141	0.009328379	DOWN
PGK2	6p12.3	-2.769556085	0.010238468	DOWN
ACOT12	5q14.1	-2.770049176	0.008191465	DOWN
LOC283665		-2.772409428	0.0024349	DOWN
OR4N3P	15q11.2	-2.774636241	0.006870127	DOWN
OR11H6	14q11.2	-2.77896796	0.009706511	DOWN
H1-8	3q22.1	-2.780908684	0.004876334	DOWN
LINC02410	12q23.1	-2.781643516	0.000221364	DOWN
LGI1	10q23.33	-2.782083722	0.001540633	DOWN
GABRA2	4p12	-2.782871092	1.84E-07	DOWN
COPG2IT1	7q32.2	-2.783694259	0.000256727	DOWN
LOC101926915		-2.784853119	0.000511796	DOWN
C3orf84	3p21.31	-2.786238119	0.000776018	DOWN
GYPA	4q31.21	-2.786380698	0.003059312	DOWN
SMIM9	Xq28	-2.786549307	0.000334523	DOWN
MTMR9LP	1p35.2	-2.792772378	0.006169764	DOWN
KHDC3L	6q13	-2.794638109	0.003209172	DOWN

**FRMD1**	6q27	-2.799785743	6.36E-06	DOWN
**OR6M1**	11q24.1	-2.800360541	0.000819828	DOWN
**C1orf185**	1p32.3	-2.808491464	0.008839656	DOWN
**GAS2L2**	17q12	-2.808711692	5.09E-06	DOWN
**CFAP46**	10q26.3	-2.809955231	1.37E-12	DOWN
**CALN1**	7q11.22	-2.835986352	0.003291862	DOWN
**ZNF536**	19q12	-2.842352262	0.002819664	DOWN
**SLC22A11**	11q13.1	-2.850301324	0.005574378	DOWN
**TMC3-AS1**	15q25.1	-2.853275171	6.81E-05	DOWN
**TEKT1**	17p13.1	-2.862256428	0.000614722	DOWN
**GLUD1P2**	10q11.22	-2.862556174	0.00282753	DOWN
**GALR1**	18q23	-2.867437209	0.007590257	DOWN
**SLC22A10**	11q12.3	-2.867614161	0.003890118	DOWN
**HS3ST4**	16p12.1	-2.870159522	5.64E-05	DOWN
**KIRREL1-IT1**	1q23.1	-2.871062349	0.001845152	DOWN
**LINC02206**	15q22.32	-2.874242991	0.001710252	DOWN
**TP53TG3D**	16p11.2	-2.876072545	0.001240587	DOWN
**KCNIP4**	4p15.31-p15.2	-2.876831394	2.56E-06	DOWN
**IGDCC3**	15q22.31	-2.878742399	0.000166791	DOWN
**LOC102723701**		-2.880262936	0.006756464	DOWN
**DPRX**	19q13.42	-2.884124305	0.001405493	DOWN
**LY6G6C**	6p21.33	-2.884239617	0.002133897	DOWN
**HRH3**	20q13.33	-2.887092521	0.001451286	DOWN
**RBFOX1**	16p13.3	-2.887573014	0.001580243	DOWN
**PTPRVP**	1q32.1	-2.888488292	0.003951017	DOWN
**ARMCX5-GPRASP2**	Xq22.1	-2.888962767	0.006254603	DOWN
**FAM43B**	1p36.12	-2.891549371	1.98E-05	DOWN
**SLC36A3**	5q33.1	-2.893709154	4.79E-07	DOWN

**RARA-AS1**	17q21.2	-2.898830976	2.69E-06	DOWN
**LRRC10B**	11q12.2	-2.910456737	1.51E-07	DOWN
**FAM181A**	14q32.12	-2.910649979	0.000384003	DOWN
**LOC105369201**		-2.913876624	0.006073924	DOWN
**SMIM10L2A**	Xq26.3	-2.914988451	0.004588624	DOWN
**IGF2-AS**	11p15.5	-2.917466568	0.000167872	DOWN
**ZBTB11-AS1**	3q12.3	-2.927091912	0.004186129	DOWN
**ATP13A4-AS1**	3q29	-2.927432214	1.38E-07	DOWN
**SPACA4**	19q13.33	-2.927923929	6.15E-05	DOWN
**PLPPR1**	9q31.1	-2.936601951	9.22E-09	DOWN
**SMIM24**	19p13.3	-2.944545793	0.00410293	DOWN
**LRCOL1**	12q24.33	-2.945612636	3.19E-05	DOWN
**CNGA3**	2q11.2	-2.957997776	2.23E-06	DOWN
**OR6F1**	1q44	-2.959747298	1.34E-08	DOWN
**MTRNR2L4**	16p13.3	-2.964515106	0.001012951	DOWN
**RAD51-AS1**	15q15.1	-2.964671498	0.004130337	DOWN
**LINC01772**	1p36.13	-2.966835938	0.00393617	DOWN
**TAMALIN-AS1**	12q13.13	-2.972482437	0.000303852	DOWN
**TBPL2**	14q22.3	-2.975077687	0.000127373	DOWN
**PGM5P4**	2q13	-2.977282324	0.001116876	DOWN
**SNORA36A**	Xq28	-2.977970723	0.006548056	DOWN
**OR10V1**	11q12.1	-2.980876388	0.00265518	DOWN
**SCGB2A1**	11q12.3	-2.981089637	8.99E-07	DOWN
**LOC105371267**		-2.987049981	0.001048206	DOWN
**KRTAP10-9**	21q22.3	-2.988764121	0.003231381	DOWN
**LINC02412**	12q22	-2.989249011	0.002792814	DOWN
**ELANE**	19p13.3	-2.994856761	3.81E-09	DOWN
**RCVRN**	17p13.1	-2.995398966	1.69E-05	DOWN

**LINC01921**	2q35	-2.999933503	0.003812254	DOWN
**NR2F1-AS1**	5q15	-3.000101372	0.003845449	DOWN
**OR14C36**	1q44	-3.010637725	6.69E-05	DOWN
**LOC100128006**		-3.013561303	0.000225456	DOWN
**TCF23**	2p23.3	-3.014735797	0.00393617	DOWN
**GPM6A**	4q34.2	-3.017225196	4.92E-08	DOWN
**LOC654780**		-3.020671179	0.004276511	DOWN
**MRGPRX3**	11p15.1	-3.022981751	0.001399997	DOWN
**KRTAP10-11**	21q22.3	-3.025033258	0.001446112	DOWN
**MT1A**	16q13	-3.030098464	0.000657933	DOWN
**TPPP2**	14q11.2	-3.032321166	0.004046041	DOWN
**CASP16P**	16p13.3	-3.034092732	0.000777682	DOWN
**SERTM1**	13q13.3	-3.034240471	0.00327077	DOWN
**LOC105379443**		-3.037038939	6.25E-11	DOWN
**DPP10**	2q14.1	-3.037447434	0.002672582	DOWN
**CDHR4**	3p21.31	-3.046214871	4.71E-05	DOWN
**LOC101928195**		-3.059805775	0.000100118	DOWN
**TMEM221**	19p13.11	-3.063585886	0.002636674	DOWN
**ADCY8**	8q24.22	-3.067599398	5.88E-06	DOWN
**SNORD116-29**	15q11.2	-3.076165334	0.00303172	DOWN
**TMEM211**	22q11.23	-3.081945813	0.002919661	DOWN
**LRRC37A11P**	17q12	-3.082387582	0.000445074	DOWN
**PSG5**	19q13.31	-3.083357361	2.04E-08	DOWN
**LOC100129603**		-3.08643296	0.002919683	DOWN
**CDH9**	5p14.1	-3.088131954	0.001879496	DOWN
**TCEAL5**	Xq22.1	-3.088605861	2.67E-13	DOWN
**LOC284344**		-3.095085033	0.001573799	DOWN
**USP26**	Xq26.2	-3.09683478	0.00059101	DOWN

**C22orf31**	22q12.1	-3.10579471	4.09E-08	DOWN
**SLC25A48-AS1**	5q31.1	-3.110555989	0.001302649	DOWN
**ZC2HC1B**	6q24.2	-3.110842715	3.46E-05	DOWN
**NPAP1**	15q11.2	-3.111190503	0.002919661	DOWN
**SLC25A41**	19p13.3	-3.116837622	0.001845012	DOWN
**FAM198B-AS1**	4q32.1	-3.134670499	9.28E-05	DOWN
**SERPINA6**	14q32.13	-3.137031631	0.001231919	DOWN
**DAAM2-AS1**	6p21.2	-3.140192099	6.37E-08	DOWN
**NMBR**	6q24.1	-3.149810579	0.000172882	DOWN
**OR1C1**	1q44	-3.152357584	1.61E-07	DOWN
**POC1B-AS1**	12q21.33	-3.15358058	1.84E-05	DOWN
**GLRA3**	4q34.1	-3.154119881	9.22E-09	DOWN
**KCNH5**	14q23.2	-3.169823644	5.88E-06	DOWN
**NR0B1**	Xp21.2	-3.170273843	0.002529959	DOWN
**OR10A3**	11p15.4	-3.183284711	9.17E-05	DOWN
**LINC02054**	3q27.1	-3.189242539	0.002133392	DOWN
**LINC01239**	9p21.3	-3.190711221	0.002051836	DOWN
**TBX22**	Xq21.1	-3.191493441	1.45E-05	DOWN
**CCDC184**	12q13.11	-3.192609727	0.000109397	DOWN
**PGC**	6p21.1	-3.198982178	0.000776018	DOWN
**DNM1P50**	15q13.2	-3.20138351	8.98E-07	DOWN
**HOXA-AS3**	7p15.2	-3.209884513	0.000462287	DOWN
**TRIM63**	1p36.11	-3.210051946	3.95E-05	DOWN
**UGT2B15**	4q13.2	-3.214833567	0.000268157	DOWN
**OR2L3**	1q44	-3.217379212	5.53E-05	DOWN
**TOMM20L**	14q23.1	-3.223789343	9.40E-09	DOWN
**KRTAP10-10**	21q22.3	-3.223893409	0.001829611	DOWN
**PMP2**	8q21.13	-3.226830811	0.001941822	DOWN

**TM4SF4**	3q25.1	-3.231139455	2.47E-08	DOWN
**KBTBD13**	15q22.31	-3.241414352	0.001468887	DOWN
**C1QTNF9**	13q12.12	-3.244716093	2.64E-09	DOWN
**PLA2G3**	22q12.2	-3.245837899	0.00059602	DOWN
**FRMPD2B**	10q11.22	-3.248906202	0.001655678	DOWN
**OR2AJ1**	1q44	-3.251034004	0.000950025	DOWN
**LINC01342**	1p36.33	-3.25269172	0.000786798	DOWN
**PMCHL2**	5q13.2	-3.259259367	0.000270718	DOWN
**ADAM30**	1p12	-3.261412253	0.001532068	DOWN
**FBXW12**	3p21.31	-3.263506585	5.75E-05	DOWN
**LOC221946**		-3.270902721	0.000485568	DOWN
**WFIKKN2**	17q21.33	-3.295281827	0.000980956	DOWN
**ARHGAP36**	Xq26.1	-3.298950735	0.001563092	DOWN
**KRT24**	17q21.2	-3.302064382	0.00107412	DOWN
**TMEM26-AS1**	10q21.2	-3.310807734	0.000939091	DOWN
**CCKAR**	4p15.2	-3.314466221	0.001206459	DOWN
**AZU1**	19p13.3	-3.334139662	0.000929999	DOWN
**NT5C1A**	1p34.2	-3.335688007	0.000745359	DOWN
**TRNP1**	14q11.2, 1p36.11	-3.339381049	0.000101157	DOWN
**CDH22**	20q13.12	-3.339847465	5.96E-09	DOWN
**LRRC3B**	3p24.1	-3.342771257	5.03E-06	DOWN
**GPR62**	3p21.2	-3.342877633	8.37E-06	DOWN
**CER1**	3p22-p21.2, 9p22.3	-3.348564584	4.54E-05	DOWN
**TAT-AS1**	16q22.2	-3.358181456	5.17E-09	DOWN
**CES1P1**	16q12.2	-3.359215687	0.000847137	DOWN
**PEBP4**	8p21.3	-3.371774036	9.33E-10	DOWN
**CHRNA2**	8p21.2	-3.373672006	0.000918367	DOWN
**FAM25A**	10q23.2	-3.376914213	0.000868447	DOWN

FGB	4q31.3	-3.377374359	0.001105702	DOWN
RXRG	1q23.3	-3.380966065	6.73E-12	DOWN
PSG10P	19q13.2	-3.389648762	1.30E-09	DOWN
MRGPRX2	11p15.1	-3.395034143	0.000768956	DOWN
OR2L13	1q44, reserved	-3.402135721	0.000464405	DOWN
CALML3	10p15.1	-3.404036629	6.02E-11	DOWN
ANKRD33	12q13.13	-3.410471399	0.000745359	DOWN
PABPN1L	16q24.3	-3.413612628	0.00018602	DOWN
CATIP	2q35	-3.415442849	0.000830713	DOWN
ASB11	Xp22.2	-3.416941221	0.000166791	DOWN
EGOT	3p26.1	-3.430853913	0.00011397	DOWN
SERPINA11	14q32.13	-3.432486424	3.39E-05	DOWN
OR2L2	1q44	-3.436901176	6.83E-11	DOWN
HOGA1	10q24.2	-3.444112573	5.80E-10	DOWN
OR2T33	1q44	-3.451491024	4.13E-07	DOWN
OR2M1P	1q44	-3.459580071	0.000672862	DOWN
TRIM54	2p23.3	-3.462360489	0.00049273	DOWN
GRID2	4q22.1-q22.2	-3.465328092	6.99E-05	DOWN
LINC01193	15q11.2	-3.465872516	0.000743116	DOWN
ZNF804B	7q21.13	-3.473495519	0.000562522	DOWN
LINC00933	15q25.2	-3.47782428	9.01E-05	DOWN
CAPZA3	12p12.3	-3.481724453	2.22E-10	DOWN
DRD1	5q35.2	-3.500699741	6.30E-08	DOWN
OR2M4	1q44	-3.504553756	0.000363997	DOWN
NOS1	10q26.11, 12q24.22	-3.514106451	6.56E-09	DOWN
LRRC53	1p31.1	-3.514418852	2.28E-06	DOWN
EMX2OS	10q26.11	-3.519128926	0.000266176	DOWN
SLC5A7	2q12.3	-3.521336904	4.95E-05	DOWN

**PIRT**	17p12	-3.529070863	0.000200043	DOWN
**ADD3-AS1**	10q25.1	-3.529651186	6.36E-06	DOWN
**CSF3**	17q21.1	-3.535094294	0.000126763	DOWN
**C2CD4C**	19p13.3	-3.550715919	5.53E-12	DOWN
**BMP3**	4q21.21	-3.555353525	1.12E-06	DOWN
**KCNC2**	12q21.1	-3.566552017	1.37E-12	DOWN
**OR10S1**	11q24.1	-3.568639927	5.65E-05	DOWN
**MRPL23-AS1**	11p15.5	-3.571690127	0.000215741	DOWN
**CEBPB-AS1**	20q13.13	-3.574015442	0.000403305	DOWN
**GALNTL6**	4q34.1	-3.575516838	1.61E-07	DOWN
**C2CD4A**	15q22.2	-3.580494322	9.37E-11	DOWN
**LMO7DN**	13q22.2	-3.587197542	1.52E-05	DOWN
**TEX13A**	Xq22.3	-3.588318507	1.65E-06	DOWN
**OR2M3**	1q44	-3.594349319	1.89E-05	DOWN
**GFAP**	17q21.31	-3.600928004	7.53E-08	DOWN
**TFF1**	21q22.3	-3.601001856	7.86E-09	DOWN
**SPATA4**	4q34.2	-3.60272577	1.08E-06	DOWN
**LINC02817**	1q41	-3.609588328	6.11E-05	DOWN
**PRDM16-DT**	1p36.32	-3.620137647	1.81E-05	DOWN
**GABRB1**	4p12	-3.621461579	5.40E-05	DOWN
**FAM138B**	2q14.1	-3.634366638	4.90E-05	DOWN
**CCL16**	17q12	-3.635259575	1.34E-05	DOWN
**GADL1**	3p24.1-p23	-3.64771702	0.00018602	DOWN
**FRRS1L**	9q31.3	-3.66913659	1.39E-08	DOWN
**TEX26**	13q12.3	-3.673509017	0.000107746	DOWN
**ADGRD1-AS1**	12q24.33	-3.678120734	0.000117571	DOWN
**ABCC12**	16q12.1	-3.71815335	2.42E-05	DOWN
**TEX47**	7q21.13	-3.72052708	4.63E-05	DOWN

**SPOCK3**	4q32.3	-3.726641288	0.000149258	DOWN
**DLK1**	14q32.2	-3.735670288	8.42E-11	DOWN
**LINC01124**	2q31.1	-3.757465099	2.88E-05	DOWN
**OR2M5**	1q44	-3.763472253	5.64E-05	DOWN
**POTEC**	18p11.21	-3.764185304	2.35E-05	DOWN
**MROH2B**	5p13.1	-3.777651773	6.87E-05	DOWN
**KRTAP10-12**	21q22.3	-3.787011834	8.37E-06	DOWN
**EIF3IP1**	7q31.1	-3.788613127	3.76E-05	DOWN
**CHST8**	19q13.11	-3.799555315	1.38E-13	DOWN
**FSIP2-AS1**	2q32.1	-3.820101039	1.89E-05	DOWN
**LINC01537**	11q13.4	-3.825950659	4.97E-08	DOWN
**ACTL7A**	9q31.3	-3.826953023	5.23E-14	DOWN
**NOP14-AS1**	4p16.3	-3.842836779	3.85E-05	DOWN
**GRIFIN**	7p22.3	-3.846612701	6.65E-05	DOWN
**CDH10**	5p14.2-p14.1	-3.856257119	1.16E-05	DOWN
**OR5K1**	3q11.2	-3.874354816	4.14E-05	DOWN
**KRT32**	17q21.2	-3.885005601	7.35E-05	DOWN
**LOC400682**		-3.894630286	2.85E-05	DOWN
**TMEFF2**	2q32.3	-3.906180717	2.74E-16	DOWN
**CYP4A11**	1p33	-3.926938354	6.47E-16	DOWN
**KIAA0087**	7p15.2	-3.929042078	1.89E-05	DOWN
**LCN6**	9q34.3	-3.935417842	9.27E-06	DOWN
**GPR88**	1p21.2	-3.938816822	5.96E-07	DOWN
**OR6X1**	11q24.1	-3.939878232	3.12E-05	DOWN
**NXNL2**	9q22.1	-3.94117341	4.73E-06	DOWN
**C1orf167**	1p36.22	-3.944253406	8.14E-06	DOWN
**LOC110091777**		-3.944331735	8.63E-12	DOWN
**OR14A16**	1q44	-3.956650771	6.73E-12	DOWN

**KRTAP10-8**	21q22.3	-3.959069196	2.21E-05	DOWN
**NPY2R**	4q32.1	-3.969282527	1.10E-12	DOWN
**NXPH1**	7p21.3	-3.979182618	8.45E-06	DOWN
**DEFB132**	20p13	-3.989210433	7.38E-06	DOWN
**GUSBP5**	4q31.21	-4.0165297	2.62E-07	DOWN
**FLJ43315**		-4.023453436	4.77E-15	DOWN
**NXNL1**	19p13.11	-4.025428145	1.88E-05	DOWN
**BPIFB1**	20q11.21	-4.026265402	4.35E-12	DOWN
**SLC22A12**	11q13.1	-4.06232352	1.45E-05	DOWN
**TCEAL6**	Xq22.1	-4.066906112	1.05E-05	DOWN
**TCF21**	6q23.2	-4.069372676	1.62E-05	DOWN
**RHAG**	6p12.3	-4.073569526	2.64E-11	DOWN
**OR2M2**	1q44	-4.082466171	1.66E-05	DOWN
**DEFB124**	20q11.21	-4.092626971	4.52E-06	DOWN
**C10orf62**	10q24.2	-4.101895079	5.16E-06	DOWN
**CLEC4M**	19p13.2	-4.102342736	1.40E-05	DOWN
**NEUROG2**	4q25	-4.104112807	7.52E-06	DOWN
**GCSAML**	1q44	-4.116888033	9.35E-13	DOWN
**PDZD3**	11q23.3	-4.121221997	4.94E-06	DOWN
**CYP4F8**	19p13.12	-4.121843531	1.05E-05	DOWN
**LOC283731**		-4.144903163	7.85E-06	DOWN
**GNAT3**	7q21.11	-4.149437993	7.31E-06	DOWN
**ASIC2**	17q11.2-q12	-4.153297458	9.59E-08	DOWN
**CLVS2**	6q22.31	-4.173190601	8.37E-06	DOWN
**HYMAI**	6q24.2	-4.17697228	3.28E-06	DOWN
**FOXI2**	10q26.2	-4.181802557	3.36E-06	DOWN
**RAB6C-AS1**	2q21.1	-4.199804762	2.96E-06	DOWN
**SLC7A13**	8q21.3	-4.204515503	8.12E-12	DOWN

**AQP7P3**	9q21.11	-4.204800146	1.33E-06	DOWN
**CARMN**	5q32	-4.216150752	5.05E-08	DOWN
**CAV3**	3p25.3	-4.225810621	4.07E-06	DOWN
**SLC7A3**	Xq13.1	-4.23926693	7.77E-11	DOWN
**PSG11**	19q13.31	-4.240771318	6.17E-09	DOWN
**ZSCAN4**	19q13.43	-4.267304975	1.01E-09	DOWN
**SLC26A3**	7q22.3-q31.1	-4.273540625	2.90E-15	DOWN
**KCNJ3**	2q24.1	-4.274834608	1.52E-10	DOWN
**AZGP1P1**	7q22.1	-4.293389745	4.25E-08	DOWN
**KLHDC7A**	1p36.13	-4.297724751	2.39E-18	DOWN
**STMN4**	8p21.2	-4.331186319	6.20E-07	DOWN
**OR2M7**	1q44	-4.336382473	1.49E-06	DOWN
**RNF186**	1p36.13	-4.339932369	9.77E-07	DOWN
**KLK3**	19q13.33, 4q35.2	-4.359082757	1.41E-09	DOWN
**RFX6**	6q22.1	-4.380148959	6.82E-15	DOWN
**OPRPN**	4q13.3	-4.398907677	2.41E-17	DOWN
**GATA3-AS1**	10p14	-4.420420765	1.32E-06	DOWN
**PLK5**	19p13.3	-4.440787061	2.09E-09	DOWN
**SPINK8**	3p21.31	-4.441270508	1.89E-09	DOWN
**PSG2**	19q13.31	-4.454816225	4.05E-07	DOWN
**SLIT3-AS2**	5q34	-4.457265706	1.65E-10	DOWN
**LINC00993**	10p11.21	-4.462937556	2.69E-18	DOWN
**GABRA4**	4p12	-4.486723664	3.00E-07	DOWN
**CST9**	20p11.21	-4.533897486	6.27E-13	DOWN
**NLRP5**	19q13.43	-4.568881219	3.34E-07	DOWN
**ERICH4**	19q13.2	-4.588746725	7.51E-11	DOWN
**LOC102723493**		-4.600439659	9.13E-08	DOWN
**MAGEB17**	Xp22.2	-4.634448164	1.22E-07	DOWN

**C10orf82**	10q25.3	-4.723274277	3.44E-13	DOWN
**SBK3**	19q13.42	-4.741023428	1.95E-08	DOWN
**IQCJ**	3q25.32	-4.809271479	1.10E-08	DOWN
**NCBP2L**	Xq22.3	-4.845942536	3.12E-09	DOWN
**TMEM252**	9q21.11	-4.857583522	8.29E-09	DOWN
**SSTR1**	14q13	-4.889563825	1.17E-12	DOWN
**BPIFB2**	20q11.21	-4.892557795	1.07E-08	DOWN
**ADAMTS9-AS2**	3p14.1	-4.905449882	1.54E-09	DOWN
**SPPL2C**	17q21.31	-4.938601122	7.86E-09	DOWN
**OR2T12**	1q44	-4.981849553	1.46E-11	DOWN
**HHATL**	3p22.1	-5.020847595	6.73E-12	DOWN
**TAC1**	7q21.3	-5.025821574	8.18E-16	DOWN
**ABCC6P1**	16p12.3	-5.045859701	1.94E-13	DOWN
**AFP**	4q13.3	-5.127906017	1.15E-18	DOWN
**UMOD**	16p12.3	-5.134492877	7.86E-10	DOWN
**PHF21B**	22q13.31	-5.193176255	4.51E-20	DOWN
**SGCZ**	8p22	-5.322768309	2.03E-11	DOWN
**MYL7**	7p13	-5.330207241	7.40E-12	DOWN
**GP2**	16p12.3	-5.386486192	1.06E-18	DOWN
**NLRP13**	19q13.43	-5.668423016	5.64E-14	DOWN
**C8orf86**	8p11.22	-5.778236459	7.51E-23	DOWN
**SRARP**	1p36.13	-5.814062662	1.06E-15	DOWN
**ABCA9-AS1**	17q24.2	-5.836231136	4.22E-15	DOWN
**DCDC2C**	2p25.3	-5.883388007	1.13E-14	DOWN
**FAM47A**	Xp21.1	-5.952584786	2.85E-22	DOWN
**CT62**	15q23	-5.954226906	2.08E-22	DOWN
**EPS8L3**	1p13.3	-6.221728489	3.11E-20	DOWN
**POTED**	21q11.2	-6.264016486	1.87E-17	DOWN

**NLRP8**	19q13.43	-6.275386551	1.06E-18	DOWN
**LINC01087**	2q21.1	-6.28837116	4.51E-20	DOWN
**Symbol**	Chromosome	logFC	adj.P.Val	TNBC regulated
**IBSP**	4q22.2	-3.238841806	5.22E-03	DOWN

Supplementary Table 3: Ingenuity Pathway Analysis of transcripts differentially expressed in TNBCtype-excluded versus TNBCtype-included tumors

Ingenuity Canonical Pathways	-log(p-value)	Ratio	Molecules
GABAergic Receptor Signaling Pathway (Enhanced)	3	0.0719	ADCY8,CACNA1H,CACNB2,GABRA2,GABRA4,GABRB1,GNAI3,GNB5,ITPR1,KCNJ3
Circadian Rhythm Signaling	2.6	0.0522	ADCY8,BTRC,CACNA1H,CACNB2,GNAI3,GNB5,GUCY1A2,ITPR1,KLK3,NOS1,NR3C1,PLCD3,PRKCE,TRPC6
GABA Receptor Signaling	2.6	0.0682	ADCY8,CACNA1H,CACNB2,GABRA2,GABRA4,GABRB1,GNAI3,GNB5,ITPR1
Cyclins and Cell Cycle Regulation	2.58	0.0824	BTRC,CCND2,FBXL5,PPP2R2C,RBL1,TFDP1,TP53
Dopamine-DARPP32 Feedback in cAMP Signaling	2.56	0.0591	ADCY8,CACNB2,DRD1,GNAI3,GUCY1A2,ITPR1,KCNJ3,NOS1,PLCD3,PPP2R2C,PRKCE
Cell Cycle: G1/S Checkpoint Regulation	2.43	0.0882	BTRC,CCND2,FBXL5,RBL1,TFDP1,TP53
nNOS Signaling in Skeletal Muscle Cells	2.41	0.104	CACNA1H,CACNB2,ITPR1,NOS1,SNTB2
Corticotropin Releasing Hormone Signaling	2.19	0.0592	ADCY8,CACNA1H,CACNB2,GLI3,GNAI3,GUCY1A2,ITPR1,NOS1,PRKCE
Phototransduction Pathway	2.19	0.0926	CNGA3,GNB5,GUCY1A2,OPN3,RCVRN
Gαs Signaling	2.18	0.0635	ADCY8,ADD1,ADD3,CNGA3,DRD1,GNAI3,GNB5,RAPGEF2
G Beta Gamma Signaling	2.12	0.062	CACNA1H,CACNB2,CAV3,GNAI3,GNB5,ITPR1,KCNJ3,PRKCE
CXCR4 Signaling	1.92	0.0536	ADCY8,GNAI3,GNB5,ITPR1,MYL7,PAK5,PAK6,PRKCE,RHOBTB2
Synaptic Long Term Depression	1.91	0.0505	CACNA1H,CACNB2,GNAI3,GRID2,GUCY1A2,ITPR1,NOS1,PLCD3,PPP2R2C,PRKCE
Oxytocin in Brain Signaling Pathway	1.9	0.0503	CACNA1H,CACNB2,GNAI3,GNB5,ITPR1,KCNJ13,NLRP13,NLRP5,NLRP8,PRKCE
Glutaminergic Receptor Signaling Pathway (Enhanced)	1.89	0.0431	ADCY8,CACNA1H,CACNB2,GABRA2,GABRA4,GABRB1,GNAI3,ITPR1,KCNK3,NR3C1,PLCD3,PLD5,PRKCE,SHANK3
Neurovascular Coupling Signaling Pathway	1.86	0.0474	BDKRB2,CACNA1H,CACNB2,CYP4A11,GABRA2,GABRA4,GABRB1,GUCY1A2,ITPR1,KCNJ3,NOS1

**GPCR-Mediated Nutrient Sensing in Enteroendocrine Cells**	1.82	0.0593	ADCY8,CACNA1H,CACNB2,GNAI3,ITPR1,PLCD3,PRKCE
**α-tocopherol Degradation**	1.81	0.222	CYP4A11,CYP4F8
**Endocannabinoid Neuronal Synapse Pathway**	1.77	0.0537	ADCY8,CACNA1H,CACNB2,GNAI3,GNB5,ITPR1,KCNJ3,PLCD3
**Non-Small Cell Lung Cancer Signaling**	1.76	0.0638	CCND2,ITPR1,RBL1,RXRG,TFDP1,TP53
**Small Cell Lung Cancer Signaling**	1.72	0.0625	CCND2,NOS1,RBL1,RXRG,TFDP1,TP53
**Apelin Cardiomyocyte Signaling Pathway**	1.66	0.0606	CAT,GNAI3,ITPR1,MYL7,PLCD3,PRKCE
**Ketogenesis**	1.64	0.182	BDH2,HMGCS2
**GPCR-Mediated Integration of Enteroendocrine Signaling Exemplified by an L Cell**	1.61	0.0667	ADCY8,GNAI3,ITPR1,NPY2R,PLCD3
**GNRH Signaling**	1.6	0.0471	ADCY8,CACNA1H,CACNB2,GNAI3,GNB5,ITPR1,PAK5,PAK6,PRKCE
**Sperm Motility**	1.57	0.0428	CACNA1H,CNGA3,GNAI3,GNB5,GUCY1A2,ITPR1,MUSK,PDGFRB,PLCD3,PRKCE,TIE1
**γ-glutamyl Cycle**	1.56	0.167	GGT5,GGT7
**Acetylcholine Receptor Signaling Pathway**	1.56	0.0464	ADCY8,APH1A,CACNA1H,CACNB2,GNAI3,ITPR1,PLCD3,PRKCE,SLC5A7
**Endothelin-1 Signaling**	1.56	0.0464	ADCY8,ECE1,GNAI3,GUCY1A2,ITPR1,NOS1,PLCD3,PLD5,PRKCE
**CCR3 Signaling in Eosinophils**	1.55	0.0522	GNAI3,GNB5,ITPR1,JMJD7-PLA2G4B,PAK5,PAK6,PRKCE
**SNARE Signaling Pathway**	1.52	0.0515	ADCY8,CACNB2,MYH3,MYL7,STXBP4,SYT14,SYT9
**BEX2 Signaling Pathway**	1.47	0.061	BAK1,KLK3,PPP2R2C,TCF4,TSC2
**Gustation Pathway**	1.46	0.0446	ADCY8,ASIC2,CACNA1H,CACNB2,GABRA2,GABRA4,GABRB1,GNAT3,ITPR1
**Leukotriene Biosynthesis**	1.44	0.143	GGT5,GGT7
**Orexin Signaling Pathway**	1.43	0.042	ADCY8,CACNA1H,CACNB2,GNB5,ITPR1,JMJD7-PLA2G4B,KCNJ3,PLCD3,PRKCE,TRPC6

**Inhibition of Angiogenesis by TSP1**	1.42	0.0882	GUCY1A2,TGFBR2,TP53
**Protein Kinase A Signaling**	1.42	0.0365	ADCY8,ADD1,ADD3,CNGA3,GLI3,GNAI3,GNB5,ITPR1,MYL7,PGP,PLCD3,PRKCE,PTPN13,TCF4,TGFBR2
**Molecular Mechanisms of Cancer**	1.39	0.0356	ADCY8,APH1A,BAK1,BMP3,CCND2,GNAI3,GNB5,PAK5,PAK6,PRKCE,RBL1,RHOBTB2,TCF4,TFDP1,TGFBR2,TP53
**Oxytocin in Spinal Neurons Signaling Pathway**	1.39	0.0857	GNAI3,GUCY1A2,NOS1
**Superpathway of Citrulline Metabolism**	1.38	0.133	CPS1,NOS1
**4-hydroxyproline Degradation I**	1.36	0.5	HOGA1
**Putrescine Biosynthesis III**	1.36	0.5	ODC1
**Opioid Signaling Pathway**	1.34	0.0393	ADCY8,CACNA1H,CACNB2,GNAI3,GNB5,ITPR1,KCNJ3,NOS1,PENK,PRKCE,TCF4
**Dilated Cardiomyopathy Signaling Pathway**	1.33	0.0467	ADCY8,CACNA1H,CACNB2,ITPR1,MYH3,MYL7,PRKCE
**Cellular Effects of Sildenafil (Viagra)**	1.33	0.0467	ADCY8,CACNB2,GUCY1A2,ITPR1,MYH3,MYL7,PLCD3
**RHOGDI Signaling**	1.27	0.0409	CDH10,CDH22,GNAI3,GNB5,MYH3,MYL7,PAK5,PAK6,RHOBTB2
**Serotonin Receptor Signaling**	1.26	0.0341	ADCY8,CACNA1H,CACNB2,CAV3,GNAI3,GNB5,KCNJ3,PAK5,PAK6,PLCD3,PLD5,PRKCE,RBL1,RHOBTB2,SHANK3,TRPC6
**Glioma Signaling**	1.24	0.048	CCND2,PDGFRB,PRKCE,RBL1,TFDP1,TP53
**Role of NFAT in Cardiac Hypertrophy**	1.23	0.0402	ADCY8,CACNA1H,CACNB2,GNAI3,GNB5,ITPR1,PLCD3,PRKCE,TGFBR2
**Epithelial Adherens Junction Signaling**	1.23	0.0443	MAGI2,MYL7,PAK5,PAK6,PPP2R2C,TCF4,TGFBR2
**Uracil Degradation II (Reductive)**	1.19	0.333	DPYD
**Inosine-5'-phosphate Biosynthesis II**	1.19	0.333	PAICS
**Thymine Degradation**	1.19	0.333	DPYD
**Estrogen Receptor Signaling**	1.17	0.0342	ADCY8,CACNB2,CCNC,DLG4,GNAI3,GNB5,MMP21,MMP27,MYL7,NR3C1,PLCD3,PRKCE,TP53,TRIM63

**Basal Cell Carcinoma Signaling**	1.14	0.0556	BMP3,GLI3,TCF4,TP53
**CREB Signaling in Neurons**	1.11	0.0313	ADCY8,BDKRB2,CACNA1H,CACNB2,DRD1,GNAI3,GNB5,GPR17,GPR62,GPR88,GRID2,ITPR1,MAS1L,NPY2R,PDGFRB,PLCD3,PRKCE,SSTR1,TGFBR2
**Iron homeostasis signaling pathway**	1.09	0.0438	BMP3,BTRC,FBXL5,PDGFRB,SLC46A1,TMPRSS6
**Glioblastoma Multiforme Signaling**	1.08	0.0409	CCND2,ITPR1,PDGFRB,PLCD3,RHOBTB2,TP53,TSC2
**White Adipose Tissue Browning Pathway**	1.08	0.0435	ADCY8,CACNA1H,CACNB2,GUCY1A2,ITPR1,RXRG
**nNOS Signaling in Neurons**	1.08	0.0638	DLG4,NOS1,PRKCE
**Synaptogenesis Signaling Pathway**	1.06	0.0349	ADCY8,CACNB2,CDH10,CDH22,DLG4,ITPR1,ITSN1,PRKCE,STXBP4,SYT14,SYT9
**α-Adrenergic Signaling**	1.04	0.0459	ADCY8,GNAI3,GNB5,ITPR1,PRKCE
**Cell Cycle: G2/M DNA Damage Checkpoint Regulation**	1.02	0.06	BTRC,FBXL5,TP53
**Role of BRCA1 in DNA Damage Response**	1.01	0.05	DPF1,RBL1,SMARCD3,TP53
**Tetrahydrofolate Salvage from 5,10-methenyltetrahydrofolate**	0.98	0.2	MTHFD2
**Tetrapyrrole Biosynthesis II**	0.98	0.2	ALAD
**Citrulline-Nitric Oxide Cycle**	0.98	0.2	NOS1
**Lactose Degradation III**	0.98	0.2	GLB1L3
**Myelination Signaling Pathway**	0.974	0.0336	APH1A,BMP3,GLI3,GPR17,KLK3,MTHFD2,PDGFRB,RAPGEF2,RXRG,TCF4,TSC2
**Estrogen-mediated S-phase Entry**	0.958	0.0769	RBL1,TFDP1
**Amyotrophic Lateral Sclerosis Signaling**	0.953	0.0431	CACNB2,CAT,GRID2,NOS1,TP53
**Bladder Cancer Signaling**	0.953	0.0431	MMP21,MMP27,RBL1,TFDP1,TP53

**PAK Signaling**	0.942	0.0427	DSCAM,MYL7,PAK5,PAK6,PDGFRB
**BAG2 Signaling Pathway**	0.935	0.0471	PSMA7,PSME4,SIRT3,TP53
**Apelin Liver Signaling Pathway**	0.931	0.0741	EDN3,PDGFRB
**HIPPO signaling**	0.922	0.0465	BTRC,DLG4,DLG5,PPP2R2C
**Factors Promoting Cardiogenesis in Vertebrates**	0.921	0.0392	BMP3,CER1,PLCD3,PRKCE,TCF4,TGFBR2
**Thrombin Signaling**	0.913	0.0356	ADCY8,GNAI3,GNB5,ITPR1,MYL7,PLCD3,PRKCE,RHOBTB2
**Sphingosine-1-phosphate Signaling**	0.908	0.0417	ADCY8,GNAI3,PDGFRB,PLCD3,RHOBTB2
**Nitric Oxide Signaling in the Cardiovascular System**	0.908	0.0417	BDKRB2,CACNB2,GUCY1A2,ITPR1,PRKCE
**Urea Cycle**	0.905	0.167	CPS1
**Selenocysteine Biosynthesis II (Archaea and Eukaryotes)**	0.905	0.167	SEPSECS
**Renin-Angiotensin Signaling**	0.897	0.0413	ADCY8,ITPR1,PAK5,PAK6,PRKCE
**eNOS Signaling**	0.892	0.0385	ADCY8,BDKRB2,CNGA3,GUCY1A2,ITPR1,PRKCE
**Signaling by Rho Family GTPases**	0.875	0.0337	CDH10,CDH22,GFAP,GNAI3,GNB5,MYL7,PAK5,PAK6,RHOBTB2
**Aryl Hydrocarbon Receptor Signaling**	0.864	0.0377	CCND2,RBL1,RXRG,TFDP1,TFF1,TP53
**Polyamine Regulation in Colon Cancer**	0.858	0.0508	ODC1,TCF4,TP53
**Colorectal Cancer Metastasis Signaling**	0.847	0.0332	ADCY8,GNAI3,GNB5,MMP21,MMP27,RHOBTB2,TCF4,TGFBR2,TP53
**Pancreatic Adenocarcinoma Signaling**	0.844	0.0397	PLD5,RBL1,TFDP1,TGFBR2,TP53
**GADD45 Signaling**	0.842	0.05	CCND2,TGFBR2,TP53
**SPINK1 Pancreatic Cancer Pathway**	0.842	0.05	ELANE,KLK3,TGFBR2
**Gap Junction Signaling**	0.838	0.0354	ADCY8,DRD1,GNAI3,GUCY1A2,ITPR1,PLCD3,PRKCE

**Semaphorin Signaling in Neurons**	0.827	0.0492	PAK5,PAK6,RHOBTB2
**Adrenergic Receptor Signaling Pathway (Enhanced)**	0.823	0.035	ADCY8,CACNA1H,CACNB2,GNAI3,ITPR1,PLCD3,PRKCE
**Chronic Myeloid Leukemia Signaling**	0.801	0.0324	CCND2,GLI3,PLCD3,PTPN13,RBL1,TCF4,TFDP1,TGFBR2,TP53
**Histidine Degradation III**	0.79	0.125	MTHFD2
**Superoxide Radicals Degradation**	0.79	0.125	CAT
**AMPK Signaling**	0.788	0.0331	AK7,DPF1,GNAI3,GNB5,MLYCD,PPP2R2C,SMARCD3,TSC2
**Androgen Signaling**	0.778	0.0355	CACNA1H,CACNB2,GNAI3,GNB5,ITPR1,PRKCE
**P2Y Purigenic Receptor Signaling Pathway**	0.776	0.0376	ADCY8,GNAI3,GNB5,PLCD3,PRKCE
**Oxytocin Signaling Pathway**	0.776	0.0319	CACNB2,GNAI3,GNB5,GUCY1A2,ITPR1,JMJD7-PLA2G4B,MYH3,MYL7,PRKCE
**DNA Methylation and Transcriptional Repression Signaling**	0.776	0.0408	RBL1,TCF21,TCF4,TP53
**Ribonucleotide Reductase Signaling Pathway**	0.769	0.0353	BTRC,DPF1,FBXL5,PARP3,SMARCD3,TP53
**Gαq Signaling**	0.769	0.0353	GNAI3,GNB5,ITPR1,PLD5,PRKCE,RHOBTB2
**Glutamate Receptor Signaling**	0.756	0.0455	DLG4,GNB5,GRID2
**Role of PKR in Interferon Induction and Antiviral Response**	0.749	0.0368	NLRP13,NLRP5,NLRP8,PDGFRB,TP53
**IL-8 Signaling**	0.748	0.0333	CCND2,GNAI3,GNB5,MYL7,PLD5,PRKCE,RHOBTB2
**Agranulocyte Adhesion and Diapedesis**	0.748	0.0333	CCL16,CCL7,GNAI3,MMP21,MMP27,MYH3,MYL7
**Neuropathic Pain Signaling in Dorsal Horn Neurons**	0.743	0.0396	ITPR1,PLCD3,PRKCE,TAC1
**Heme Biosynthesis II**	0.743	0.111	ALAD
**Folate Transformations I**	0.743	0.111	MTHFD2
**WNT/β-catenin Signaling**	0.738	0.0345	BTRC,PPP2R2C,SOX4,TCF4,TGFBR2,TP53

**MIF-mediated Glucocorticoid Regulation**	0.731	0.0556	JMJD7-PLA2G4B,NR3C1
**Agrin Interactions at Neuromuscular Junction**	0.717	0.0435	MUSK,PAK5,PAK6
**Sirtuin Signaling Pathway**	0.716	0.0308	ATG2B,CPS1,HMGCS2,MLYCD,POLR1B,SIRT3,TOMM20L,TP53,TP53BP1
**Stearate Biosynthesis I (Animals)**	0.704	0.0429	BDH2,CYP4A11,SLC27A3
**Ketolysis**	0.702	0.1	BDH2
**Cell Cycle Regulation by BTG Family Proteins**	0.695	0.0526	PPP2R2C,PRMT1
**Notch Signaling**	0.695	0.0526	APH1A,DTX3
**WNK Renal Signaling Pathway**	0.693	0.0377	ASIC2,KLHL3,PRKCE,UBR1
**Tight Junction Signaling**	0.693	0.0333	F11R,MAGI2,MYH3,MYL7,PPP2R2C,TGFBR2
**Calcium Signaling**	0.681	0.0318	CACNA1H,CACNB2,ITPR1,MYH3,MYL7,TRDN,TRPC6
**Melatonin Signaling**	0.68	0.0417	GNAI3,PLCD3,PRKCE
**Ephrin B Signaling**	0.68	0.0417	GNAI3,GNB5,ITSN1
**Netrin Signaling**	0.68	0.0417	CACNA1H,CACNB2,ITPR1
**Inhibition of Matrix Metalloproteases**	0.678	0.0513	MMP21,MMP27
**Cardiac Hypertrophy Signaling**	0.669	0.0307	ADCY8,CACNB2,GNAI3,GNB5,MYL7,PLCD3,RHOBTB2,TGFBR2
**Purine Nucleotides De Novo Biosynthesis II**	0.665	0.0909	PAICS
**Pyrimidine Ribonucleotides Interconversion**	0.662	0.05	AK7,PGP
**Endocannabinoid Cancer Inhibition Pathway**	0.657	0.034	ADCY8,CCND2,GNAI3,NOS1,TCF4
**Axonal Guidance Signaling**	0.638	0.0275	ADAM20,BMP3,GLI3,GNAI3,GNB5,ITSN1,MMP21,MMP27,MYL7,NTN5,PAK5,PAK6,PLCD3,PRKCE
**Angiopoietin Signaling**	0.635	0.0395	PAK5,PAK6,TIE1

**Semaphorin Neuronal Repulsive Signaling Pathway**	0.634	0.0333	GUCY1A2,MYL7,PAK5,PAK6,TP53
**Acetone Degradation I (to Methylglyoxal)**	0.631	0.0476	CYP4A11,CYP4F8
**Antiproliferative Role of Somatostatin Receptor 2**	0.624	0.039	GNB5,GUCY1A2,NOS1
**Prostate Cancer Signaling**	0.62	0.0351	KLK3,RBL1,TFDP1,TP53
**Pyrimidine Ribonucleotides De Novo Biosynthesis**	0.616	0.0465	AK7,PGP
**VDR/RXR Activation**	0.613	0.0385	CCNC,PRKCE,RXRG
**Type II Diabetes Mellitus Signaling**	0.612	0.0327	CACNA1H,CACNB2,ITPR1,PRKCE,SLC27A3
**Regulation of Actin-based Motility by Rho**	0.612	0.0348	MYL7,PAK5,PAK6,RHOBTB2
**S100 Family Signaling Pathway**	0.605	0.0259	BDKRB2,CACNA1H,CACNB2,DRD1,GPR17,GPR62,GPR88,ITPR1,JMJD7-PLA2G4B,KLK3,MAS1L,MMP21,MMP27,NPY2R,NR3C1,PLCD3,PRKCE,SSTR1,TCF4,TP53
**Thyroid Cancer Signaling**	0.603	0.038	KLK3,TCF4,TP53
**Assembly of RNA Polymerase I Complex**	0.602	0.0769	POLR1B
**MIF Regulation of Innate Immunity**	0.601	0.0455	JMJD7-PLA2G4B,TP53
**BER (Base Excision Repair) Pathway**	0.601	0.0455	NEIL1,TP53
**Hepatic Fibrosis / Hepatic Stellate Cell Activation**	0.598	0.0309	ECE1,LHX2,MYH3,MYL7,PDGFRB,TGFBR2
**Dopamine Receptor Signaling**	0.593	0.0375	ADCY8,DRD1,PPP2R2C
**Mevalonate Pathway I**	0.574	0.0714	HMGCS2
**Phenylalanine Degradation IV (Mammalian, via Side Chain)**	0.574	0.0714	SLC27A3

**Pulmonary Healing Signaling Pathway**	0.567	0.0302	DLK1,ELANE,MMP21,MMP27,PRKCE,TCF4
**Neuroprotective Role of THOP1 in Alzheimer's Disease**	0.563	0.0331	ECE1,KLK3,PRSS41,TAC1
**Ephrin Receptor Signaling**	0.55	0.0297	GNAI3,GNB5,ITSN1,PAK5,PAK6,PTPN13
**Fatty Acid Activation**	0.549	0.0667	SLC27A3
**Choline Biosynthesis III**	0.549	0.0667	PLD5
**Apelin Muscle Signaling Pathway**	0.548	0.0417	GNAI3,GNB5
**Huntington's Disease Signaling**	0.546	0.0282	DLG4,GNB5,ITPR1,PENK,PRKCE,PSMA7,PSME4,TP53
**Regulation of the Epithelial Mesenchymal Transition in Development Pathway**	0.527	0.0345	APH1A,GLI3,TCF4
**D-myo-inositol (1,4,5)-trisphosphate Degradation**	0.525	0.0625	INPP1
**Parkinson's Signaling**	0.525	0.0625	SNCAIP
**FAT10 Cancer Signaling Pathway**	0.524	0.04	TGFBR2,TP53
**14-3-3-mediated Signaling**	0.519	0.0315	GFAP,PLCD3,PRKCE,TSC2
**TNFR1 Signaling**	0.512	0.0392	PAK5,PAK6
**UVC-Induced MAPK Signaling**	0.512	0.0392	PRKCE,TP53
**Amyloid Processing**	0.512	0.0392	APH1A,PRKCE
**Dermatan Sulfate Degradation (Metazoa)**	0.504	0.0588	IDUA
**NER (Nucleotide Excision Repair, Enhanced Pathway)**	0.502	0.0333	POLD1,POLD2,UVSSA
**UVB-Induced MAPK Signaling**	0.501	0.0385	PRKCE,TP53
**Apelin Adipocyte Signaling Pathway**	0.494	0.033	ADCY8,CAT,GNAI3

**fMLP Signaling in Neutrophils**	0.491	0.0305	GNAI3,GNB5,ITPR1,PRKCE
**Aldosterone Signaling in Epithelial Cells**	0.489	0.0291	ASIC2,DNAJB5,ITPR1,PLCD3,PRKCE
**Synaptic Long Term Potentiation**	0.485	0.0303	ADCY8,ITPR1,PLCD3,PRKCE
**Mismatch Repair in Eukaryotes**	0.483	0.0556	POLD1
**Superpathway of Geranylgeranyldiphosphate Biosynthesis I (via Mevalonate)**	0.483	0.0556	HMGCS2
**Senescence Pathway**	0.478	0.0268	CACNB2,CAT,CCND2,DMTF1,PPP2R2C,RBL1,TGFBR2,TP53
**Pyroptosis Signaling Pathway**	0.478	0.0323	NLRP13,NLRP5,NLRP8
**ERBB Signaling**	0.478	0.0323	PAK5,PAK6,PRKCE
**RAR Activation**	0.469	0.0256	ADCY8,CNGA3,DLK1,DPF1,F11R,NEUROG2,PRMT1,RHOBTB2,RXRG,SMARCD3,TGFBR2
**γ-linolenate Biosynthesis II (Animals)**	0.464	0.0526	SLC27A3
**Mitochondrial L-carnitine Shuttle Pathway**	0.464	0.0526	SLC27A3
**Cell Cycle Control of Chromosomal Replication**	0.459	0.0357	MCM6,POLD1
**IL-1 Signaling**	0.455	0.0312	ADCY8,GNAI3,GNB5
**FAT10 Signaling Pathway**	0.449	0.0351	PSMA7,PSME4
**Salvage Pathways of Pyrimidine Ribonucleotides**	0.447	0.0309	AK7,PAK5,PRKCE
**Cardiac β-adrenergic Signaling**	0.445	0.0278	ADCY8,CACNB2,GNAI3,GNB5,PPP2R2C
**UVA-Induced MAPK Signaling**	0.44	0.0306	PARP3,PLCD3,TP53
**Role of CHK Proteins in Cell Cycle Checkpoint Control**	0.44	0.0345	PPP2R2C,TP53

Gαi Signaling	0.435	0.0286	ADCY8,GNAI3,GNB5,GPR17
Apelin Endothelial Signaling Pathway	0.429	0.0284	ADCY8,GNAI3,GNB5,PRKCE
ATM Signaling	0.426	0.03	PPP2R2C,TP53,TP53BP1
Hereditary Breast Cancer Signaling	0.424	0.0282	CCND2,DPF1,SMARCD3,TP53
Retinoic acid Mediated Apoptosis Signaling	0.421	0.0333	PARP3,RXRG
Superpathway of D-myo-inositol (1,4,5)-trisphosphate Metabolism	0.414	0.0455	INPP1
Neuroinflammation Signaling Pathway	0.407	0.0252	APH1A,GABRA2,GABRA4,GABRB1,JMJD7-PLA2G4B,KCNJ3,KLK3,TGFBR2
Sumoylation Pathway	0.406	0.0291	NR3C1,RHOBTB2,TP53
Insulin Secretion Signaling Pathway	0.402	0.0255	ADCY8,ASIC2,CACNA1H,CACNB2,ITPR1,PLCD3,PRKCE
Granulocyte Adhesion and Diapedesis	0.4	0.0265	CCL16,CCL7,GNAI3,MMP21,MMP27
Apoptosis Signaling	0.4	0.0288	BAK1,PRKCE,TP53
Pyrimidine Deoxyribonucleotides De Novo Biosynthesis I	0.399	0.0435	AK7
Methionine Degradation I (to Homocysteine)	0.399	0.0435	PRMT1
Leukocyte Extravasation Signaling	0.382	0.0259	F11R,GNAI3,MMP21,MMP27,PRKCE
PXR/RXR Activation	0.379	0.0308	HMGCS2,NR3C1
Induction of Apoptosis by HIV1	0.379	0.0308	BAK1,TP53
Pyridoxal 5'-phosphate Salvage Pathway	0.379	0.0308	PAK5,PRKCE
NAD Signaling Pathway	0.375	0.0265	HMGCS2,PARP3,SIRT3,TP53
Bupropion Degradation	0.372	0.04	CYP4F8
Gluconeogenesis I	0.372	0.04	ME2
Ethanol Degradation IV	0.372	0.04	CAT

**Cysteine Biosynthesis III (mammalia)**	0.372	0.04	PRMT1
**Breast Cancer Regulation by Stathmin1**	0.37	0.0236	ANKHD1/ANKHD1-EIF4EBP3,BDKRB2,CCND2,DRD1,GNB5,GPR17,GPR62,GPR88,MAS1L,NPY2R,PPP2R2C,PRKCE,SSTR1,TP53
**CLEAR Signaling Pathway**	0.364	0.0246	ITPR1,PDGFRB,PPP2R2C,PRKCE,TGFBR2,TP53,TSC2
**Mitotic Roles of Polo-Like Kinase**	0.364	0.0299	PLK5,PPP2R2C
**D-myo-inositol (1,4,5)-Trisphosphate Biosynthesis**	0.359	0.0385	PLCD3
**ERBB4 Signaling**	0.356	0.0294	APH1A,PRKCE
**Sertoli Cell-Sertoli Cell Junction Signaling**	0.356	0.0247	CDH10,CDH22,F11R,PAK5,PAK6,TGFBR2
**Relaxin Signaling**	0.355	0.0258	ADCY8,GNAI3,GNB5,GUCY1A2
**Necroptosis Signaling Pathway**	0.355	0.0258	JMJD7-PLA2G4B,RBL1,TOMM20L,TP53
**Adrenomedullin signaling pathway**	0.355	0.0251	ADCY8,GUCY1A2,ITPR1,PLCD3,RXRG
**Actin Cytoskeleton Signaling**	0.352	0.0246	MYH3,MYL7,NCKAP1,PAK5,PAK6,TMSB10/TMSB4X
**Hepatic Fibrosis Signaling Pathway**	0.35	0.0236	CACNB2,GLI3,GNAI3,MYL7,PDGFRB,PRKCE,RHOBTB2,SIRT3,TCF4,TGFBR2
**Phospholipases**	0.349	0.029	PLCD3,PLD5
**Human Embryonic Stem Cell Pluripotency**	0.347	0.0249	BMP3,KLK3,PDGFRB,PRKCE,TGFBR2
**ILK Signaling**	0.347	0.0249	MYH3,MYL7,PPP2R2C,RHOBTB2,TMSB10/TMSB4X
**Ovarian Cancer Signaling**	0.341	0.0253	RBL1,TCF4,TFDP1,TP53
**CDP-diacylglycerol Biosynthesis I**	0.335	0.0357	TAMM41
**CDK5 Signaling**	0.335	0.0261	ADCY8,DRD1,PPP2R2C
**IL-13 Signaling Pathway**	0.33	0.0259	DEFB124,DEFB132,TP53
**G-Protein Coupled Receptor Signaling**	0.326	0.0228	ADCY8,BDKRB2,CNGA3,DRD1,GNAI3,GNB5,GPR17,GPR62,GPR88,MAS1L,MYL7,NPY2R,PAK5,PAK6,PRKCE,SSTR1
**Neuregulin Signaling**	0.325	0.0256	DLG4,PRKCE,TMEFF2

**Superpathway of Cholesterol Biosynthesis**	0.324	0.0345	HMGCS2
**Airway Pathology in Chronic Obstructive Pulmonary Disease**	0.32	0.0254	ELANE,LCN10,LCN6
**HIF1α Signaling**	0.319	0.024	MMP21,MMP27,P4HTM,PRKCE,TP53
**Inhibition of ARE-Mediated mRNA Degradation Pathway**	0.319	0.0245	PPP2R2C,PSMA7,PSME4,ZFP36L2
**Mitochondrial Dysfunction**	0.315	0.0232	APH1A,CACNA1H,CACNB2,ITPR1,NOS1,SIRT3,TP53,UQCRHL
**Cholecystokinin/Gastrin-mediated Signaling**	0.315	0.0252	ITPR1,PRKCE,RHOBTB2
**Phosphatidylglycerol Biosynthesis II (Non-plastidic)**	0.314	0.0333	TAMM41
**Role of p14/p19ARF in Tumor Suppression**	0.314	0.0333	TP53
**p38 MAPK Signaling**	0.31	0.025	JMJD7-PLA2G4B,TGFBR2,TP53
**Methylglyoxal Degradation III**	0.304	0.0323	CYP4A11
**Sonic Hedgehog Signaling**	0.304	0.0323	GLI3
**Leptin Signaling in Obesity**	0.303	0.0263	ADCY8,PLCD3
**mTOR Signaling**	0.297	0.0234	PLD5,PPP2R2C,PRKCE,RHOBTB2,TSC2
**TREM1 Signaling**	0.297	0.026	CCL7,NLRP5
**IL-15 Production**	0.295	0.0244	MUSK,PDGFRB,TIE1
**ERK/MAPK Signaling**	0.294	0.0233	JMJD7-PLA2G4B,PAK5,PAK6,PPP2R2C,PRKCE
**Germ Cell-Sertoli Cell Junction Signaling**	0.291	0.0235	PAK5,PAK6,RHOBTB2,TGFBR2
**Autophagy**	0.287	0.023	ATG2B,GNAI3,PPP2R2C,TP53,TSC2
**Airway Inflammation in Asthma**	0.285	0.0303	ELANE
**Maturity Onset Diabetes of Young (MODY) Signaling**	0.285	0.0253	APOO,CACNB2

**Renal Cell Carcinoma Signaling**	0.285	0.0253	PAK5,PAK6
**Endocannabinoid Developing Neuron Pathway**	0.277	0.0236	ADCY8,GNAI3,GNB5
**Fatty Acid β-oxidation I**	0.277	0.0294	SLC27A3
**Chemokine Signaling**	0.274	0.0247	CCL7,GNAI3
**Coagulation System**	0.268	0.0286	BDKRB2
**Ethanol Degradation II**	0.268	0.0286	DHRS2
**Erythropoietin Signaling Pathway**	0.268	0.0227	CCND2,ITPR1,PRKCE,TP53
**Interferon Signaling**	0.26	0.0278	BAK1
**VEGF Family Ligand-Receptor Interactions**	0.258	0.0238	JMJD7-PLA2G4B,PRKCE
**PEDF Signaling**	0.258	0.0238	TCF4,TP53
**HER-2 Signaling in Breast Cancer**	0.255	0.022	NUP93,PRKCE,RBL1,TP53,TSC2
**Noradrenaline and Adrenaline Degradation**	0.252	0.027	DHRS2
**Complement System**	0.252	0.027	C4BPB
**Gα12/13 Signaling**	0.252	0.0226	CDH10,CDH22,MYL7
**Superpathway of Methionine Degradation**	0.245	0.0263	PRMT1
**IL-17A Signaling in Fibroblasts**	0.244	0.023	CCL7,NFKBIZ
**PDGF Signaling**	0.244	0.023	CAV3,PDGFRB
**DHCR24 Signaling Pathway**	0.237	0.0219	PRKCE,RXRG,TP53
**RAC Signaling**	0.237	0.0219	NCKAP1,PAK5,PAK6
**Regulation of Cellular Mechanics by Calpain Protease**	0.23	0.0222	CCND2,CNGA3
**Mechanisms of Viral Exit from Host Cells**	0.224	0.0244	PRKCE

**Intrinsic Prothrombin Activation Pathway**	0.218	0.0238	KLK3
**Pulmonary Fibrosis Idiopathic Signaling Pathway**	0	0.0184	MMP21,MMP27,PDGFRB,TCF4,TGFBR2,TP53
**Wound Healing Signaling Pathway**	0	0.00794	TAC1,TGFBR2
**ID1 Signaling Pathway**	0	0.00995	TGFBR2,TP53
**CDX Gastrointestinal Cancer Signaling Pathway**	0	0.0099	BMP3,TCF4
**Immunogenic Cell Death Signaling Pathway**	0	0.0111	BAK1
**MicroRNA Biogenesis Signaling Pathway**	0	0.016	DDX17,NUP93,TP53
**Multiple Sclerosis Signaling Pathway**	0	0.0045	PARP3
**Pathogen Induced Cytokine Storm Signaling Pathway**	0	0.00809	CCL16,CCL7,NLRP5
**Role of Chondrocytes in Rheumatoid Arthritis Signaling Pathway**	0	0.0142	MMP21,MMP27
**Role of Osteoblasts in Rheumatoid Arthritis Signaling Pathway**	0	0.0164	BMP3,MMP21,MMP27,TCF4
**Role of Osteoclasts in Rheumatoid Arthritis Signaling Pathway**	0	0.013	ADAM20,MMP21,MMP27,RHOBTB2
**Glucocorticoid Receptor Signaling**	0	0.012	DPF1,JMJD7-PLA2G4B,KRT32,NR3C1,RXRG,SMARCD3,TGFBR2
**Natural Killer Cell Signaling**	0	0.0101	PAK5,PAK6
**Macrophage Alternative Activation Signaling Pathway**	0	0.0106	NR3C1,RXRG
**Neutrophil Extracellular Trap Signaling Pathway**	0	0.0125	ELANE,ITPR1,PLCD3,PRKCE,TOMM20L
**Fc Epsilon RI Signaling**	0	0.0169	JMJD7-PLA2G4B,PRKCE

**Chaperone Mediated Autophagy Signaling Pathway**	0	0.00945	GFAP,MMP21,MMP27,PSMA7,SIRT3,TP53
**IL-33 Signaling Pathway**	0	0.00541	ELANE
**Activin Inhibin Signaling Pathway**	0	0.0138	CCND2,TCF4,TRIM63
**Cachexia Signaling Pathway**	0	0.019	ADCY8,NR3C1,PRKCE,PSMA7,PSME4,TGFBR2,TRIM63
**ISGylation Signaling Pathway**	0	0.00926	TP53
**Microautophagy Signaling Pathway**	0	0.0187	PSMA7,PSME4,TP53
**Sertoli Cell Germ Cell Junction Signaling Pathway (Enhanced)**	0	0.0169	F11R,GUCY1A2,PPP2R2C,TGFBR2
**NRF2-mediated Oxidative Stress Response**	0	0.0169	CAT,CYP4A11,DNAJB5,PRKCE
**PPARα/RXRα Activation**	0	0.0155	ADCY8,PLCD3,TGFBR2
**LPS/IL-1 Mediated Inhibition of RXR Function**	0	0.0195	CAT,CYP4A11,HMGCS2,HS3ST4,SLC27A3
**p53 Signaling**	0	0.0204	CCND2,TP53
**Acute Phase Response Signaling**	0	0.0162	C4BPB,NR3C1,TCF4
**Hepatic Cholestasis**	0	0.0105	ADCY8,PRKCE
**LXR/RXR Activation**	0	0.0163	CCL7,RXRG
**Ceramide Signaling**	0	0.011	PPP2R2C
**TR/RXR Activation**	0	0.0156	RXRG,SLC16A2
**Caveolar-mediated Endocytosis Signaling**	0	0.0133	ITSN1
**Fcγ Receptor-mediated Phagocytosis in Macrophages and Monocytes**	0	0.0213	PLD5,PRKCE
**IL-12 Signaling and Production in Macrophages**	0	0.00426	PRKCE

**Role of Pattern Recognition Receptors in Recognition of Bacteria and Viruses**	0	0.0128	CLEC6A,PRKCE
**Role of NFAT in Regulation of the Immune Response**	0	0.0029	GNAI3,GNB5,ITPR1
**FcγRIIB Signaling in B Lymphocytes**	0	0.00563	CACNA1H,CACNB2,ITPR1
**LPS-stimulated MAPK Signaling**	0	0.0118	PRKCE
**NF-κB Activation by Viruses**	0	0.0128	PRKCE
**CCR5 Signaling in Macrophages**	0	0.012	CACNA1H,CACNB2,GNAI3,GNB5,ITPR1,PRKCE
**Calcium-induced T Lymphocyte Apoptosis**	0	0.00435	ITPR1,PRKCE
**IL-3 Signaling**	0	0.0127	PRKCE
**IL-17 Signaling**	0	0.0107	DEFB124,DEFB132
**Thrombopoietin Signaling**	0	0.0159	PRKCE
**CTLA4 Signaling in Cytotoxic T Lymphocytes**	0	0.00329	PLD5,PPP2R2C
**T Helper Cell Differentiation**	0	0.00212	TGFBR2
**CD28 Signaling in T Helper Cells**	0	0.00193	ITPR1
**Virus Entry via Endocytic Pathways**	0	0.0169	ITSN1,PRKCE
**Dendritic Cell Maturation**	0	0.00168	PLCD3
**Reelin Signaling in Neurons**	0	0.00725	APBB1
**ICOS-ICOSL Signaling in T Helper Cells**	0	0.00197	ITPR1
**HGF Signaling**	0	0.00758	PRKCE
**HMGB1 Signaling**	0	0.00599	RHOBTB2

**Melanocyte Development and Pigmentation Signaling**	0	0.0102	ADCY8
**Role of OCT4 in Mammalian Embryonic Stem Cell Pluripotency**	0	0.0217	TP53
**Role of NANOG in Mammalian Embryonic Stem Cell Pluripotency**	0	0.0161	BMP3,TP53
**Growth Hormone Signaling**	0	0.0141	PRKCE
**Prolactin Signaling**	0	0.0211	NR3C1,PRKCE
**Melanoma Signaling**	0	0.02	TP53
**Endometrial Cancer Signaling**	0	0.0167	TP53
**Acute Myeloid Leukemia Signaling**	0	0.011	TCF4
**Production of Nitric Oxide and Reactive Oxygen Species in Macrophages**	0	0.0209	CAT,PPP2R2C,PRKCE,RHOBTB2
**p70S6K Signaling**	0	0.00688	GNAI3,PLCD3,PPP2R2C,PRKCE
**MYC Mediated Apoptosis Signaling**	0	0.02	TP53
**G Protein Signaling Mediated by Tubby**	0	0.00855	GNAI3,GNB5,NPY2R,TTC21B
**Systemic Lupus Erythematosus Signaling**	0	0.00281	BDKRB2,PRPF40B,SNRNP70
**CDC42 Signaling**	0	0.00174	MYL7
**FAK Signaling**	0	0.0134	BDKRB2,DRD1,GPR17,GPR62,GPR88,MAS1L,NPY2R,PAK5,PAK6,PDGFRB,SSTR1,TCF4,TGFBR2,TP53
**EIF2 Signaling**	0	0.00435	RPL30
**RHOA Signaling**	0	0.0161	MYL7,RAPGEF2
**Role of Osteoblasts, Osteoclasts and Chondrocytes in Rheumatoid Arthritis**	0	0.00877	BMP3,TCF4

**Phospholipase C Signaling**	0	0.00803	ADCY8,GNB5,ITPR1,JMJD7-PLA2G4B,MYL7,PLCD3,PLD5,PRKCE,RHOBTB2
**Role of Macrophages, Fibroblasts and Endothelial Cells in Rheumatoid Arthritis**	0	0.00904	PLCD3,PRKCE,TCF4
**Regulation of eIF4 and p70S6K Signaling**	0	0.00543	PPP2R2C
**Glioma Invasiveness Signaling**	0	0.0137	RHOBTB2
**Regulation of IL-2 Expression in Activated and Anergic T Lymphocytes**	0	0.00216	TGFBR2
**Role of WNT/GSK-3β Signaling in the Pathogenesis of Influenza**	0	0.0128	TCF4
**NUR77 Signaling in T Lymphocytes**	0	0.00195	PRKCE
**PKCθ Signaling in T Lymphocytes**	0	0.00538	CACNA1H,CACNB2,ITPR1
**Role of PI3K/AKT Signaling in the Pathogenesis of Influenza**	0	0.0152	GNAI3
**Antiproliferative Role of TOB in T Cell Signaling**	0	0.00235	TGFBR2
**MSP-RON Signaling Pathway**	0	0.0172	KLK3
**PI3K Signaling in B Lymphocytes**	0	0.00338	ITPR1,PLCD3
**Role of Tissue Factor in Cancer**	0	0.0145	RBL1,TGFBR2,TP53
**Spliceosomal Cycle**	0	0.0189	PRPF40B
**Role of IL-17A in Arthritis**	0	0.0175	CCL7
**Role of IL-17F in Allergic Inflammatory Airway Diseases**	0	0.0213	CCL7

**Role of JAK2 in Hormone-like Cytokine Signaling**	0	0.0161	CCND2
**Actin Nucleation by ARP-WASP Complex**	0	0.0108	RHOBTB2
**NGF Signaling**	0	0.00833	TP53
**Paxillin Signaling**	0	0.0187	PAK5,PAK6
**Telomerase Signaling**	0	0.0185	PPP2R2C,TP53
**Mouse Embryonic Stem Cell Pluripotency**	0	0.0192	TCF4,TP53
**Transcriptional Regulatory Network in Embryonic Stem Cells**	0	0.0122	TGFBR2,TP53
**GDNF Family Ligand-Receptor Interactions**	0	0.0132	ITPR1
**Heparan Sulfate Biosynthesis**	0	0.0111	HS3ST4
**Nicotine Degradation III**	0	0.0169	CYP4F8
**Heparan Sulfate Biosynthesis (Late Stages)**	0	0.012	HS3ST4
**D-myo-inositol-5-phosphate Metabolism**	0	0.0204	PLCD3,PPP2R2C,PTPN13,THTPA
**Melatonin Degradation I**	0	0.0159	CYP4F8
**Estrogen Biosynthesis**	0	0.0217	CYP4F8
**Chondroitin Sulfate Biosynthesis**	0	0.0164	HS3ST4
**Dermatan Sulfate Biosynthesis**	0	0.0159	HS3ST4
**Dermatan Sulfate Biosynthesis (Late Stages)**	0	0.02	HS3ST4
**D-myo-inositol (1,4,5,6)-Tetrakisphosphate Biosynthesis**	0	0.0167	PPP2R2C,PTPN13,THTPA
**Nicotine Degradation II**	0	0.0149	CYP4F8
**Superpathway of Inositol Phosphate Compounds**	0	0.0171	PLCD3,PPP2R2C,PTPN13,THTPA

**Serotonin Degradation**	0	0.0139	DHRS2
**D-myo-inositol (3,4,5,6)-tetrakisphosphate Biosynthesis**	0	0.0167	PPP2R2C,PTPN13,THTPA
**3-phosphoinositide Degradation**	0	0.0157	PPP2R2C,PTPN13,THTPA
**3-phosphoinositide Biosynthesis**	0	0.0146	PPP2R2C,PTPN13,THTPA
**Chondroitin Sulfate Biosynthesis (Late Stages)**	0	0.0189	HS3ST4
**Superpathway of Melatonin Degradation**	0	0.0147	CYP4F8
**Antioxidant Action of Vitamin C**	0	0.0175	PLCD3,PLD5
**DNA damage-induced 14-3-3σ Signaling**	0	0.0217	TP53
**Regulation of the Epithelial-Mesenchymal Transition Pathway**	0	0.0205	APH1A,PDGFRB,TCF4,TGFBR2
**TEC Kinase Signaling**	0	0.0104	GNAI3,GNB5,PAK5,PAK6,PRKCE,RHOBTB2
**STAT3 Pathway**	0	0.0148	PDGFRB,TGFBR2
**Oxidative Phosphorylation**	0	0.00893	UQCRHL
**Adipogenesis pathway**	0	0.0144	DLK1,TP53
**Unfolded protein response**	0	0.0111	DNAJB5
**WNT/Ca+ pathway**	0	0.0152	PLCD3
**EGF Signaling**	0	0.0179	ITPR1
**SAPK/JNK Signaling**	0	0.00398	GNB5,TP53
**PI3K/AKT Signaling**	0	0.015	PPP2R2C,TP53,TSC2
**PTEN Signaling**	0	0.0199	MAGI2,PDGFRB,TGFBR2
**Protein Ubiquitination Pathway**	0	0.0183	BTRC,DNAJB5,PSMA7,UBR1,USP53
**FGF Signaling**	0	0.0116	ITPR1

**Xenobiotic Metabolism Signaling**	0	0.017	ANKRA2,CAT,HS3ST4,PPP2R2C,PRKCE
**IL-4 Signaling**	0	0.00347	GNAI3,NR3C1
**Insulin Receptor Signaling**	0	0.0214	ASIC2,STXBP4,TSC2
**Neurotrophin/TRK Signaling**	0	0.0128	KLK3
**Integrin Signaling**	0	0.0189	MYL7,PAK5,PAK6,RHOBTB2
**Death Receptor Signaling**	0	0.0104	PARP3
**PPAR Signaling**	0	0.00935	PDGFRB
**cAMP-mediated signaling**	0	0.0212	ADCY8,CNGA3,DRD1,GNAI3,GPR17
**TGF-β Signaling**	0	0.0104	TGFBR2
**NF-κB Signaling**	0	0.00525	BTRC,PDGFRB,TGFBR2
**Hypoxia Signaling in the Cardiovascular System**	0	0.0132	TP53
**T Cell Receptor Signaling**	0	0.00162	TCF4
**BMP signaling pathway**	0	0.011	BMP3
**Phagosome Formation**	0	0.0216	BDKRB2,DRD1,GPR17,GPR62,GPR88,ITPR1,MAS1L,MYH3,MYL7,NPY2R,PAK5,PAK6,PLD5,PRKCE,SSTR1
**Phagosome Maturation**	0	0.0061	NOS1
**Macropinocytosis Signaling**	0	0.0132	PRKCE
**Cancer Drug Resistance by Drug Efflux**	0	0.0172	TP53
**Th1 and Th2 Activation Pathway**	0	0.0116	APH1A,TGFBR2
**Th1 Pathway**	0	0.0082	APH1A
**Th2 Pathway**	0	0.0146	APH1A,TGFBR2
**Osteoarthritis Pathway**	0	0.0127	GLI3,TCF4,TGFBR2
**GP6 Signaling Pathway**	0	0.0157	ITPR1,PRKCE
**IL-7 Signaling Pathway**	0	0.0128	BAK1
**Th17 Activation Pathway**	0	0.00413	DEFB124,DEFB132
**Cardiac Hypertrophy Signaling (Enhanced)**	0	0.0166	ADCY8,CACNB2,GNAI3,GNB5,ITPR1,PLCD3,PRKCE,TGFBR2,TSC2

**PFKFB4 Signaling Pathway**	0	0.0208	TP53
**T Cell Exhaustion Signaling Pathway**	0	0.00529	FOXP1,PPP2R2C,TGFBR2
**Systemic Lupus Erythematosus in T Cell Signaling Pathway**	0	0.00778	GNAI3,ITPR1,NOS1,PPP2R2C,RHOBTB2
**Systemic Lupus Erythematosus in B Cell Signaling Pathway**	0	0.00276	CCND2,PRKCE
**HOTAIR Regulatory Pathway**	0	0.0184	MMP21,MMP27,TCF4
**Xenobiotic Metabolism General Signaling Pathway**	0	0.00699	PRKCE
**Xenobiotic Metabolism AHR Signaling Pathway**	0	0.0115	ANKRA2
**Xenobiotic Metabolism CAR Signaling Pathway**	0	0.0155	HS3ST4,PPP2R2C,PRKCE
**Xenobiotic Metabolism PXR Signaling Pathway**	0	0.0154	CAT,HS3ST4,PRKCE
**Regulation of the Epithelial Mesenchymal Transition by Growth Factors Pathway**	0	0.0104	PDGFRB,TGFBR2
**Kinetochore Metaphase Signaling Pathway**	0	0.00909	CENPQ
**Coronavirus Pathogenesis Pathway**	0	0.0196	RBL1,TFDP1,TGFBR2,TP53
**Tumor Microenvironment Pathway**	0	0.0112	MMP21,MMP27
**MSP-RON Signaling in Cancer Cells Pathway**	0	0.0143	KLK3,TCF4
**MSP-RON Signaling in Macrophages Pathway**	0	0.0168	KLK3,NFKBIZ
**Ferroptosis Signaling Pathway**	0	0.0153	RBL1,TP53

Supplementary Table 4: Gene network analysis of transcripts differentially expressed in TNBCtype-excluded versus TNBCtype-included tumors

ID	Molecules in Network	Score	Focus Molecules	Top Diseases and Functions
1	ANKHD1/ANKHD1-EIF4EBP3,BRIX1,CCNL2,CENPQ,CPS1,D2HGDH,DAZAP1,DBX2,DHRS2,FIGLA, FOXK2,GAS2L2,GRID2,IFT46,IFT88,Immunoglobulin,Inflammasome, KANSL1,NEIL1,NLRP13,NLRP5,NUDCD1,PAICS,RBM5,RERE,RHAG,SIRT3,SMC6,SMG6,TASOR2, TP53BP1,UQCRHL,YTHDC2,ZFP36L2,ZNF609	60	33	[Endocrine System Disorders, Organismal Injury and Abnormalities, Reproductive System Disease]
2	AGR3,Alp,ANKRD42,apyrase,ARL15,AURK,BMP,BMP3,CDH10,CDH22,CER1, CSMD3,DEFB124,DENND2C,DTX3,EDA2R,MIPOL1,NFkB (complex),NRM, NXNL1,PGP,PTPN13,RAB3C,RABEP1,SETBP1,Smad,Sod,STXBP4,TCF21, TMEM252,TMPRSS6,TMSB10/TMSB4X,TRIM52,TTC12,UMOD	46	28	[Developmental Disorder, Hereditary Disorder, Organismal Injury and Abnormalities]
3	ACTL6A,BOP1,CCNC,COBLL1,DDX17,DPF1,FOXP1,GTPase,H2BC4,Histone h2a, Histone h3,Histone h4,Insulin,LHX2,NISCH,NUP93,NXF1,NXPH1,PLPPR1,POLR1B, POLR1G,PRMT1,RALGAPA1,RALGAPA2,RALY,RFX6,RIOK1,RNA polymerase II,Rnr, RRP1B,SMARCD3,SNRNP70,TFF1,ZNF106,ZNF83	46	28	[Cell Morphology, Gene Expression, RNA Post-Transcriptional Modification]
4	20s proteasome,AHI1,ARL6:GTP:BBSome:ciliary cargo,Basal body:transition zone proteins,BBS1,BBS5,BBSome,CEP290,Dlg,DLG5,DNAJB5,FAM120B,IDUA,KCNIP4, KIF13B,LCN6,LZTFL1 oligomer:BBSome,NALP,NLRP8,NPHP1,NPTX1,P38 MAPK,Proteasome,PSMA7,PSME4,PUS7,RAB3IP:BBSome,RPSA2,SEPSECS,SPAG6, SREK1,SRSF9,TBC1D9,TTC8,WDR72	39	25	[Developmental Disorder, Hereditary Disorder, Organismal Injury and Abnormalities]
5	Adaptor protein 2,ADCY,ADCY8,APOO,ARMCX5-GPRASP2/GPRASP2,ASIC2,ATG2B, BDKRB2,CYP,CYP4A11,DRD1,G protein alpha,Girk,GNRH,Gpcr,GPR62,GPR88,Growth hormone,HELLS,IBSP,Ifn,MAGEA8,MAS1L,MUSK,NPY2R,RCBTB2,SCRN2,SLC25A25, SLC27A3,SLC46A1,SNX21,SSTR1,TMEM209,USP53,Vegf	39	25	[Cell Signaling, Nucleic Acid Metabolism, Ophthalmic Disease]
6	Akt,ARRDC3,ARRDC4,BAIAP2L1,C2CD4A,CLIP3,creatine kinase,DLGAP2,EMC6, FRMD1,GGT5,HDX,ITSN1,Kallikrein,KIAA0232,MAGI2, NRXNs:NLGN dimer:PSD-95:GKAP1-4:SHANK1,2,3,NRXNs:NLGN dimer: PSD-95:GKAP1-4:SHANK1,2,3:2xHOMER1,2,3:GRM1,5,NRXNs:NLGN dimer: PSD-95:GKAP1-4:SHANK1,2,3:DBNL,Pak,PDGF (family), PDGF-DD,PHLDB1,PHYHIP,PSG5,Rab11,RAPGEF2,SHANK3,SLC22A5,SLC7A3, SYCP3,TIE1,TRPC6,TTC21B,UBIQUITIN LIGASE	37	24	[Nephrosis, Organismal Injury and Abnormalities, Renal and Urological Disease]
7	AFF1,Alpha tubulin,APH1A,BETA TUBULIN,BPIFB1,DHRS12,DNA damage response, DNA-directed DNA polymerase, dopamine receptor,Dynein,ERK1/2,FOXP4,GALNT6,LDL-cholesterol,mediator,MLH3,OPN3,P4HTM,PARP3,POLD1,POLD2,PRIMPOL, PSG11,PSGs:Proteoglycan,TGFB1,RFWD3,RPA,RSPH6A,SCGB2A1,SPHKAP, TC-NER incision complex: 5'-incised damaged DNA:trimmed nascent mRNA:(PCNA:POLD,POLE), (MonoUb:K164-PCNA:POLK):RPA:RFC, TC-NER post-incision complex:SSB-dsDNA:trimmed nascent mRNA: (PCNA:POLD,POLE), (MonoUb:K164-PCNA:POLK):RPA:RFC,TIMELESS,TMEFF2,UVSSA,WRNIP1	32	22	[DNA Replication, Recombination, and Repair, Metabolic Disease, Organismal Injury and Abnormalities]
8	ADD1,ADD3,Adducin,C4BPB,CCNL1,CCT/TRiC:substrate,COMMD10, COQ6,CRL E3 ubiquitin ligase:CAND1,CRL1 E3 ubiquitin ligase	32	22	[Cellular Assembly and Organization, Cellular

	complex:MyrG-DCUN1D3,CRL1 E3 ubiquitin ligase:COMMDs:CCDC22,DCAF12,<br><br>DCUN1D3,DDO,DMTF1,FBXL5,<br><br>FBXW12,KLHL3,LATS,LONP2,ME2,MLYCD,NEDD8-AcM-UBE2M:CRL1 E3 ubiquitin<br><br>ligase:COMMDs:CCDC22:DCUN1Ds,NEDD8-CRL1 E3 ubiquitin<br><br>ligase:COMMDs:CCDC22:DCUN1Ds,Nuclear factor 1,PEBP4,PEX5S,L:Cargo protein,PEX5S,L:Cargo:PEX13:PEX14:PEX2:PEX10:PEX12,PRDM11,<br><br>RCVRN,SLC35F2,SLC5A7,SWI-SNF,TP53,<br><br>TP53 Tetramer:E2F4:(TFDP1,TFDP2):(RBL1,RBL2):CDC25C Gene			Compromise, Increased Levels of Hematocrit]
9	AFP,APBB1,ARID5B,BPIFB2,CERS3,CLEC4M,CLEC6A,DRD1/5,ELANE,F11R,FAM20C:FAM20C substrates,FCRL4,Iga,IL23,KCTD5,LRP,Mir122a,b,MRTFB,Mucin,NEUROG2,NHERF4,NR-MED1 Coactivator,NR-NCOR CoRepressor Complex,Nr1h,NR2E3,OPRPN,PENK,PI3K (complex),Rab5,Retinoic acid-RAR-RXR,RGMB,RXRG,SLC26A3,STAMBP,TAMM41	32	22	[Endocrine System Development and Function, Nervous System Development and Function, Organ Morphology]
10	BDH2,C10orf62,CDCA4,CHML,Collagen type IV,DPYD,ECE1,GABA-A receptor,Gabr A,GABRA2,GLRX3,GUCY1A2,Hsp27,KRTAP10-8,Mlc,MYO15B,NBEA,p70 S6k,PAK5,Pdgf (complex),PDGF BB,PDGFRB,phosphatase,Pkc(s),PP2A,PPP2R2C,RABGGTB,ROCK,SLC22A23,SNTB2,SNX4,Stat5 dimer,SYTL5,TEX13A,THTPA	32	22	[Nucleic Acid Metabolism, Post-Translational Modification, Small Molecule Biochemistry]
11	EBF1:LDB1:LHX2:Intergenic olfactory enhancer:Olfactory Receptor gene,LHX2,OR10A3,OR10A6,OR10G4,OR10S1,OR13C3,OR13G1,OR14A16,OR14C36,OR1C1,OR1D4,OR2L2,OR2L3,OR2M2,OR2M3,OR2M5,OR2M7,OR2T1,OR2T12,OR2T33,OR4C3,OR4N2,OR51E1,OR51E2,OR51Q1,OR5A2,OR5K1,OR5M11,OR6C75,OR6F1,OR6M1,OR6X1,OR8B8,ZC2HC1B	28	20	[Cancer, Cell-To-Cell Signaling and Interaction, Nervous System Development and Function]
12	ADP:P2RY12:G-protein Gi (inactive),C1QTNF9,CACNA1H,CACNB2,calpain,CAV3,CNGA3,ERK,ESTG:ESRs:STRN:heterotrimeric G(i) protein,FRRS1L,G protein beta gamma core,GABA receptor,GABR heteropentamers:GABA,GABR heteropentamers:GABA:NPTN,GABRA4,GABRB1,GNAI3,GNAT3,GNB5,GRIA,HEPACAM2,Heterotrimeric G-protein Gi (inactive),INPP1,ITPR,KCNJ3,L-type Calcium Channel,N-type Calcium Channel,Pkg,PLEKHH3,Ryr,SHISA9,STMN4,TAC1,TRDN,voltage-gated calcium channel	26	19	[Cardiac Arrythmia, Cardiovascular Disease, Organismal Injury and Abnormalities]
13	CAT,CHST8,Collagen Alpha1,collagen type i (family),DLK1,Focal adhesion kinase,GAL3ST1,GGT7,GPM6A,ICA1L,IFN Beta,Ige,IgG,Igm,IL12 (complex),IL12 (family),JINK1/2,Ldh (complex),LDL,LSR,PAK6,PHF21B,PI3K (family),PI3K p85,Rac,RHOBTB2,SLC16A2,SOX4,SRC (family),TCF4,Tgf beta,TGFBR2,THSD1,TRIM58,TRMO	24	18	[Cancer, Ophthalmic Disease, Organismal Injury and Abnormalities]
14	AHR,Airway Pathology in Chronic Obstructive Pulmonary Disease,AK7,BCAS2,C10orf82,CD37,CFAP70,CIT,DNAI4,FAM228B,GOT,GP2,HARS1,HOGA1,IDUA,IL13,LARP7,LCN,LCN10,LCN6,LCN9,LRRC3B,LRRC53,LRRK2,MATK,MROH2B,OBSL1,OLFML1,P4HB,PEBP4,PHF2,PLCXD3,RAF1,RIOX1,WDR73	23	17	[Cellular Function and Maintenance, Cellular Movement, Gastrointestinal Disease]
15	Actin,Ag-substrate:E3:E2:Ub,ASB15,ASB18,BTRC,CBLL2,DET1,E3 co-factor,E3:Ub:substrate,EGFLAM,F Actin,FBXW12,GFAP,Ikb,MHC Class II (complex),Myosin2,PDCD2L,PFDN6,PLC gamma,Proinsulin,Ras homolog,RNF115,RPL30,SLC39A6,SNX33,TRIM50,tubulin,UBE2G1,UBE3B,UBE3D,Ubiquitin,UBR1,UNKL,WDR6,WWOX	21	16	[Connective Tissue Disorders, Developmental Disorder, Post-Translational Modification]
16	26s Proteasome,ALAD,ANKRA2,BAK1,Calcineurin protein(s),Calmodulin,CAMK2,cAMP-dependent protein kinase,CAPZA3,Ck2,Creb,cytochrome C,DLG4,ELFN2,FRMPD4,Hsp70,Hsp90 (family),IGSF22,ITPR1,KCNC2,KCNH3,KLK3,MYL7,Nfat (family),Ngf,nicotinic acetylcholine receptor,NMDA Receptor,NOS1,Octamer of Voltage gated K channels,PARP,Pka,Pka catalytic subunit,PRKAA,RAS,SYT9	19	15	[Cell Death and Survival, Cell-To-Cell Signaling and Interaction, Cellular Assembly and Organization]
17	AMPK,B4GALT1,BCR (complex),caspase,CCND2,CG,CPEB2,Cyclin B,E3:K48-iquitinated substrate,EFCAB6,FAM13B,FSH,Gsk3,hemoglobin,Interferon alpha,KCNK3,Lh,LTBP3,MAP2K1/2,Mek,MTHFD2,MTORC1,NR3C1,ODC1,p85 (pik3r),PRKCE,PTK,Smad2/3,Sos,SPATS2,STAT5a/b,TCR,TRIM63,TSC2,ZNF823	19	15	[Digestive System Development and Function, Nucleic Acid Metabolism, Organismal Development]
18	ACCS,ACTL8,ADAMTS9-AS2,B3GALT1,CCDC170,CLXN,CYP3A43,CYP4A11,CYP4F8,ECM1,EPS8L3,FAP,H2BC7,HNF4A,KCNK5,KLK14,mir-365,MSMB,MYC,MYCT1,Par,Prss34,PRSS41,PRSS57,RORC,SCGB1D2,Serine Protease,Serpin,SLC13A2,SPINK5,TCF21,TM4SF4,TMPRSS11A,TPSD1,UQCC4	19	15	[Cancer, Hematological Disease, Immunological Disease]
19	ANXA9,Cbp/p300,Cdc2,Cdk (family),CPEB3,Cyclin A,Cyclin D,Cyclin E,DSCAM,E2f,EDN3,estrogen receptor,Hdac,HISTONE,histone deacetylase,Histone H1,HMG CoA synthase,HMGCS2,Jnk,MCM6,NF-Y,NTN5,Rb,RBL1,SLC7A8,SNCAIP,SUMO1P3,TCF,TFDP1,TFDP3,thymidine kinase,TIP60,TOB2,transcription factor,YAP/TAZ	17	14	[Cell Cycle, Embryonic Development, Renal and Urological System Development and Function]
20	14-3-3,ADAM20,Alpha catenin,Ap1,CALML3,CCL16,CCL7,CD3,chemokine,Collagen type I (complex),Collagen(s),cytokine,G protein,G protein alpha i,GLI3,Hedgehog,Hif1,IL1,Mapk,Metalloprotease,Mmp,MMP21,MMP27,NFKBIZ,NLN,Notch,PLC,PLCD3,PLD,PLD5,PRDX4,SPACA4,SYNPO,Tnf (family),trypsin	17	14	[Cellular Movement, Hematological System Development and Function, Immune Cell Trafficking]
21	AR,CNDP2,CUX2,DNAH14,FRMPD2,GALNT18,GALNTL6,HNRNPL,KCNH5,KCNJ3,KCNQ5,LUZP2,Mitotic Roles of Polo-Like	17	14	[Developmental Disorder, Organismal Injury and

	Kinase,MTARC1,MYOM1,NXNL2,OR51E2,PDE9A,Plk,PLK5,PRC1,SERPINB6,SH3YL1,SHPRH,SLC15A5,SPPL2C,STARD10,STAT5B,TBX22,TMEM144,TMEM176B,TMEM98,TTC14,TTC21A,ZNF362			Abnormalities, Reproductive System Disease]
22	ABCA5,ANKRD13C,APP,C19orf18,C1orf105,CD44,CDC123,CRB2,CST9,ECE2,FBXO7,Fnbp1l,HINT3,IKBKE,ITGA4,KIAA0513,LIMS2,LRRC10B,METTL25,NEK11,NFAT (complex),PHTF2,PPT2-EGFL8,PRPF40B,PSMG3,RAD51C,SEMA5A,SLC41A3,SPATA2L,SPATA4,SRARP,STIMATE-MUSTN1,STING1,TRIM25,UBE2G1	17	14	[Cancer, Cell-To-Cell Signaling and Interaction, Immunological Disease]
23	AFP,ASTN1,BIRC3,BRD4,C2CD4C,CAV1,Caveolin,CYLD:CLIP3:TNF:TNFR1:TRADD:K63pUb-RIP1:TRAF2:BIRC2/3,DNM1L,FAM90A1,FAM90A26 (includes others),Filamin,GCSAML,GPR107,JMJD7-PLA2G4B,JTB,LAMP1,LINC00518,LRIT3,mir-499,MSRB2,Necroptosis Signaling Pathway,P-TEFb,PSG2,RAB23,RFPL4A/RFPL4AL1,RFPL4B,TENM3,TMEM135,TNFRSF10B,TP53COR1,TRAF2,ZNF280D,ZNF763,ZNF784	17	14	[Cell Cycle, Cell Death and Survival, Organismal Injury and Abnormalities]
24	ATXN3,CDHR4,CREB1,DCDC2C,HMMR,KRAB-ZNF / KAP,LOC100288966/POTED,POTEC,PRKD2,PSG3,PSG4,TCEAL5,TCEAL6,TOMM20L,TRIM28,ZFP28,ZKSCAN5,ZNF10,ZNF138,ZNF14,ZNF221,ZNF266,ZNF439,ZNF441,ZNF471,ZNF500,ZNF559,ZNF625,ZNF649,ZNF679,ZNF697,ZNF74,ZNF786,ZNF79,ZNF799	17	14	[Cellular Compromise, Cellular Function and Maintenance, Cellular Movement]
25	ACTL7A,BCL6B,C1orf167,C1orf174,C22orf31,CCDC181,CFAP46,CLVS2,CYB5R2,DDX39A,DENND2A,ERICH4,FAM43B,FOXI2,HDAC1,HK3,IQSEC3,KANK3,LRRC27,MEOX2,MYL7,NAA16,NKX3-2,NT5DC4,PAFAH1B2,PCYT2,PLA2G15,PRKN,SOX8,SPEF2,SUOX,SYT14,TEX47,ZFAND4,ZNF23	15	13	[Developmental Disorder, Embryonic Development, Organismal Development]

Supplementary Table 5: Differentially expressed transcripts in tumors classified vs. unclassified in TNBCtype analysis

Symbol	Chromosome	logFC	adj.P.Val	Classified regulated
THBS4-AS1	5q14.1	3.983482	0.012564	UP
EDRF1-AS1	10q26.2	3.862959	0.023975	UP
SH3GLB2	9q34.11	1.277673	0.031906	UP
WDR27	6q27	1.196377	0.008078	UP
CTDSP1	2q35	1.184243	0.002146	UP
HYAL2	3p21.31	1.047103	0.004913	UP
TERF2IP	16q23.1	0.951434	0.027452	UP
TMEM260	14q22.3	0.748762	0.027452	UP
KLHL22	22q11.21	0.636441	0.035153	UP
LMBR1L	12q13.12	0.581036	0.042462	UP
DCLRE1B	1p13.2	-0.62591	0.042284	DOWN
MCM10	10p13	-0.72499	0.031906	DOWN
CD3E	11q23.3	-0.82552	0.022873	DOWN
CDH13	16q23.3	-0.86565	0.043519	DOWN
GBP5	1p22.2	-0.87792	0.046168	DOWN
SNORD118	17p13.1	-0.95521	0.009675	DOWN
RAPGEFL1	17q21.1	-0.99871	0.017821	DOWN
PROM2	2q11.1	-1.00219	0.010338	DOWN
BTBD1	15q25.2	-1.06764	0.000991	DOWN
GM2A	5q33.1	-1.09871	0.027228	DOWN
MST1	20q13.12, 3p21.31	-1.10157	0.000259	DOWN
SH2D1A	Xq25	-1.11244	0.020653	DOWN
MICB	6p21.33	-1.11249	0.005479	DOWN
AOC2	17q21.31	-1.12623	0.007113	DOWN
OR2A9P	7q35	-1.13783	0.004913	DOWN

**SYT8**	11p15.5	-1.17187	0.031906	DOWN
**AGAP3**	7q36.1	-1.20253	0.023089	DOWN
**C9orf47**	9q22.1	-1.22422	0.01804	DOWN
**NDUFC2-KCTD14**	11q14.1	-1.26242	0.006508	DOWN
**MYCN**	2p24.3	-1.28182	0.006508	DOWN
**ZNF653**	19p13.2	-1.3392	0.004913	DOWN
**WDR64**	1q43	-1.35425	0.049439	DOWN
**DHDDS-AS1**	1p36.11	-1.3571	0.005173	DOWN
**PRICKLE3**	Xp11.23	-1.36551	0.00064	DOWN
**GABRB3**	15q12	-1.38752	0.042284	DOWN
**CLDN24**	4q35.1	-1.40722	0.004913	DOWN
**NXPE2**	11q23.2	-1.42937	0.023975	DOWN
**ZNF826P**	19p12	-1.49676	0.012747	DOWN
**MUCL3**	6p21.33	-1.50055	0.023089	DOWN
**NKAPL**	6p22.1	-1.51032	0.014317	DOWN
**PWARSN**	15q11.2	-1.59632	0.001261	DOWN
**AS3MT**	10q24.32	-1.59665	0.030124	DOWN
**NCCRP1**	19q13.2	-1.60156	0.004913	DOWN
**BORCS8**	19p13.11	-1.62295	0.030961	DOWN
**MB**	22q12.3	-1.65913	0.000382	DOWN
**EID2B**	19q13.2	-1.66359	0.000706	DOWN
**KRT83**	12q13.13	-1.81791	0.018946	DOWN
**MPIG6B**	6p21.33	-1.82487	0.001551	DOWN
**TSHB**	1p13.2	-1.88899	0.027452	DOWN
**SLC6A11**	3p25.3	-1.94998	1.07E-05	DOWN
**CADM2**	3p12.1	-1.97695	0.000827	DOWN
**NPY4R**	10q11.22	-1.98796	0.028333	DOWN
**STPG3-AS1**	9q34.3	-2.0157	1.50E-05	DOWN

**LOC101928008**	NA	-2.07284	0.048052	DOWN
**FAM3D**	3p14.2	-2.08137	0.009675	DOWN
**CT83**	Xq23	-2.12596	0.049017	DOWN
**RPS16P5**	6p12.1	-2.23943	1.07E-05	DOWN
**BRINP2**	1q25.2	-2.24922	0.005596	DOWN
**CCDC60**	12q24.23	-2.33981	0.031906	DOWN
**CNTN5**	11q22.1	-2.34305	0.042284	DOWN
**NDP**	Xp11.3	-2.36971	0.001048	DOWN
**RBM24**	6p22.3	-2.47502	0.001261	DOWN
**CYYR1-AS1**	21q21.3	-2.54577	0.020653	DOWN
**LOC100128079**	NA	-2.77907	0.004735	DOWN
**RPE65**	1p31.3	-2.94248	0.001551	DOWN
**FIRRE**	Xq26.2	-2.96134	0.004913	DOWN
**MIR492**	12q22	-3.03802	1.36E-06	DOWN
**KRT75**	12q13.13	-3.27331	2.31E-06	DOWN
**C4orf45**	4q32.1	-3.4828	0.004735	DOWN
**ASIC2**	17q11.2-q12	-3.91654	0.031449	DOWN
**SYCE3**	22q13.33	-3.91773	0.012852	DOWN

Supplementary Table 6: Ingenuity Pathway Analysis of transcripts differentially in TNBCtype unclassified tumors versus classified tumors

Ingenuity Canonical Pathways	-log(p)	Ratio	Molecules
Chondroitin Sulfate Degradation (Metazoa)	3.1	0.125	GM2A,HYAL2
Dermatan Sulfate Degradation (Metazoa)	3.05	0.118	GM2A,HYAL2
Phenylethylamine Degradation I	1.98	0.25	AOC2
Arsenate Detoxification I (Glutaredoxin)	1.88	0.2	AS3MT
Telomere Extension by Telomerase	1.41	0.0667	TERF2IP
GABA Receptor Signaling	1.33	0.0152	GABRB3,SLC6A11
GABAergic Receptor Signaling Pathway (Enhanced)	1.29	0.0144	GABRB3,SLC6A11
The Visual Cycle	1.21	0.0417	RPE65
Role of JAK1, JAK2 and TYK2 in Interferon Signaling	1.18	0.0385	TSHB
Natural Killer Cell Signaling	1.02	0.0101	MICB,SH2D1A
Gustation Pathway	1.01	0.0099	ASIC2,GABRB3
Mechanisms of Viral Exit from Host Cells	0.991	0.0244	SH3GLB2
Sertoli Cell-Sertoli Cell Junction Signaling	0.875	0.00823	CDH13,CLDN24
MSP-RON Signaling Pathway	0.85	0.0172	MST1
Triacylglycerol Degradation	0.85	0.0172	RPE65
Primary Immunodeficiency Signaling	0.836	0.0167	CD3E
PCP (Planar Cell Polarity) Pathway	0.836	0.0167	NDP
Glucocorticoid Receptor Signaling	0.708	0.00515	CD3E,KRT75,KRT83
Heparan Sulfate Biosynthesis (Late Stages)	0.708	0.012	RPE65
Synaptogenesis Signaling Pathway	0.699	0.00635	CDH13,SYT8
Neuroinflammation Signaling Pathway	0.695	0.00631	GABRB3,SLC6A11
HIPPO signaling	0.694	0.0116	MST1
Heparan Sulfate Biosynthesis	0.676	0.0111	RPE65
Ceramide Signaling	0.672	0.011	S1PR3
Crosstalk between Dendritic Cells and Natural Killer Cells	0.672	0.011	MICB
Pyroptosis Signaling Pathway	0.664	0.0108	GBP5
WNK Renal Signaling Pathway	0.614	0.00943	ASIC2

**Telomerase Signaling**	0.607	0.00926	TERF2IP
**MSP-RON Signaling in Macrophages Pathway**	0.571	0.0084	MST1
**Sphingosine-1-phosphate Signaling**	0.567	0.00833	S1PR3
**Th1 Pathway**	0.561	0.0082	CD3E
**TR/RXR Activation**	0.544	0.00781	TSHB
**Gα12/13 Signaling**	0.53	0.00752	CDH13
**SNARE Signaling Pathway**	0.522	0.00735	SYT8
**Th2 Pathway**	0.519	0.0073	CD3E
**Gαi Signaling**	0.511	0.00714	S1PR3
**Insulin Receptor Signaling**	0.511	0.00714	ASIC2
**MSP-RON Signaling in Cancer Cells Pathway**	0.511	0.00714	MST1
**Epithelial Adherens Junction Signaling**	0.468	0.00633	MST1
**Aldosterone Signaling in Epithelial Cells**	0.439	0.00581	ASIC2
**Th1 and Th2 Activation Pathway**	0.439	0.00581	CD3E
**Tight Junction Signaling**	0.423	0.00556	CLDN24
**Granulocyte Adhesion and Diapedesis**	0.406	0.00529	CLDN24
**Leukocyte Extravasation Signaling**	0.399	0.00518	CLDN24
**Clathrin-mediated Endocytosis Signaling**	0.375	0.00481	SH3GLB2
**Agranulocyte Adhesion and Diapedesis**	0.372	0.00476	CLDN24
**ERK/MAPK Signaling**	0.364	0.00465	MYCN
**RHOGDI Signaling**	0.356	0.00455	CDH13
**EIF2 Signaling**	0.342	0.00435	MYCN
**Neurovascular Coupling Signaling Pathway**	0.339	0.00431	GABRB3
**IL-12 Signaling and Production in Macrophages**	0.335	0.00426	MST1
**Sertoli Cell Germ Cell Junction Signaling Pathway (Enhanced)**	0.334	0.00424	CLDN24
**cAMP-mediated signaling**	0.334	0.00424	S1PR3
**Osteoarthritis Pathway**	0.334	0.00424	S1PR3
**Breast Cancer Regulation by Stathmin1**	0.334	0.00337	NPY4R/NPY4R2,S1PR3
**CREB Signaling in Neurons**	0.323	0.00329	NPY4R/NPY4R2,S1PR3

**Wound Healing Signaling Pathway**	0.314	0.00397	MST1
**Chaperone Mediated Autophagy Signaling Pathway**	0.302	0.00315	CD3E,MST1
**Systemic Lupus Erythematosus in T Cell Signaling Pathway**	0.296	0.00311	CD3E,S1PR3
**Signaling by Rho Family GTPases**	0.296	0.00375	CDH13
**Insulin Secretion Signaling Pathway**	0.287	0.00364	ASIC2
**FAK Signaling**	0.286	0.00288	CD3E,NPY4R/NPY4R2, S1PR3
**Sirtuin Signaling Pathway**	0.27	0.00342	MYCN
**Phagosome Formation**	0.262	0.00288	NPY4R/NPY4R2,S1PR3
**G-Protein Coupled Receptor Signaling**	0.257	0.00284	NPY4R/NPY4R2,S1PR3
**Glutaminergic Receptor Signaling Pathway (Enhanced)**	0.239	0.00308	GABRB3
**S100 Family Signaling Pathway**	0	0.00259	NPY4R/NPY4R2,S1PR3
**NFKBIE Signaling Pathway**	0	0.00223	CD3E
**RAR Activation**	0	0.00233	RPE65
**Role of NFAT in Regulation of the Immune Response**	0	0.000965	CD3E
**CCR5 Signaling in Macrophages**	0	0.002	CD3E
**Calcium-induced T Lymphocyte Apoptosis**	0	0.00217	CD3E
**Cytotoxic T Lymphocyte-mediated Apoptosis of Target Cells**	0	0.00235	CD3E
**CTLA4 Signaling in Cytotoxic T Lymphocytes**	0	0.00164	CD3E
**T Helper Cell Differentiation**	0	0.00212	CD3E
**CD28 Signaling in T Helper Cells**	0	0.00193	CD3E
**Dendritic Cell Maturation**	0	0.00168	CD3E
**ICOS-ICOSL Signaling in T Helper Cells**	0	0.00197	CD3E
**Lipid Antigen Presentation by CD1**	0	0.00241	CD3E
**Type I Diabetes Mellitus Signaling**	0	0.00196	CD3E
**Allograft Rejection Signaling**	0	0.00205	CD3E
**Autoimmune Thyroid Disease Signaling**	0	0.00218	CD3E
**Graft-versus-Host Disease Signaling**	0	0.00224	CD3E
**G Protein Signaling Mediated by Tubby**	0	0.00214	CD3E
**Communication between Innate and Adaptive Immune Cells**	0	0.00107	CD3E

**Systemic Lupus Erythematosus Signaling**	0	0.000937	CD3E
**CDC42 Signaling**	0	0.00174	CD3E
**Phospholipase C Signaling**	0	0.000892	CD3E
**Altered T Cell and B Cell Signaling in Rheumatoid Arthritis**	0	0.00106	CD3E
**Regulation of IL-2 Expression in Activated and Anergic T Lymphocytes**	0	0.00216	CD3E
**NUR77 Signaling in T Lymphocytes**	0	0.00195	CD3E
**PKCθ Signaling in T Lymphocytes**	0	0.00179	CD3E
**Antiproliferative Role of TOB in T Cell Signaling**	0	0.00235	CD3E
**OX40 Signaling Pathway**	0	0.00209	CD3E
**Hematopoiesis from Pluripotent Stem Cells**	0	0.00224	CD3E
**TEC Kinase Signaling**	0	0.00173	CD3E
**SAPK/JNK Signaling**	0	0.00199	CD3E
**IL-4 Signaling**	0	0.00173	CD3E
**NF-κB Signaling**	0	0.00175	CD3E
**T Cell Receptor Signaling**	0	0.00162	CD3E
**Th17 Activation Pathway**	0	0.00207	CD3E
**T Cell Exhaustion Signaling Pathway**	0	0.00176	CD3E

Supplementary Table 7: Gene network analysis of transcripts differentially expressed in TNBCtype unclassified versus classified tumors

ID	Molecules in Network	Score	Focus Molecules	Top Diseases and Functions
1	Akt,BTBD1,CD3E,CDH13,CG,CNTN5,DCLRE1B,ERK,ERK1/2,FAM3D,G protein alpha i,GBP5,GM2A,Histone h3,HYAL2,Immunoglobulin,Insulin,LMBR1L,MCM10,MICB,MST1,MYCN,NFkB (complex),NKAPL,NPY4R/NPY4R2,PWARSN,RAS,RBM24,RNA polymerase II,S1PR3,SH2D1A,SH3GLB2,SNORD118,SYT8,TERF2IP	61	24	[Developmental Disorder, Hereditary Disorder, Metabolic Disease]
2	Agranulocyte Adhesion and Diapedesis,AMTN,AOC2,ASIC2,ASIC3,BORCS8,BRINP2,CLDN24,CXCL16,DDX39A,ENaC,EPO,FIRRE,GABRA5,GABRB3,GPR18,IL11RA,IL1B,IL4R,KLHL22,LRAT,MYH10,NCCRP1,NRTN,PRSS8,PTPN1,QSOX1,RPE65,SLC6A11,SOX2,TMEM260,TNF,WDR27,ZNF503-AS1,ZNF653	31	14	[Cell Death and Survival, Cellular Compromise, Neurological Disease]
3	AHR,APP,AR,AS3MT,BCL6,C4orf45,CBFA2T2,CCDC60,CCR7,EDN2,EID2B,EID3,FAM32A,HBZ,Keratin,KRT3,KRT79,KRT83,MAGEF1,Mt1,MUCL3,NDP,NEUROD2,NR3C1,NXPE2,RAPGEFL1,SLC38A5,SMCHD1,SYCE2,SYCE3,TCF15,Tgf beta,TSHB,TSPAN12,WDR64	25	12	[Cancer, Organ Morphology, Organismal Injury and Abnormalities]
4	AGAP3,CADM2,CDC42SE1,CPNE7,CTDSP1,EGLN3,EPM2AIP1,G6B:PTPN6,PTPN11,GASK1B,HEXIM1,Hsf3,KIF9,KRT20,KRT24,KRT25,KRT26,KRT75,KRT79,LRAT,MB,mir-492,miR-492 (miRNAs w/seed GGACCUG),MPIG6B,MYO16,NDUFC2-KCTD14,PDZD4,PRICKLE3,PROM2,PTPN6,RAC1,STAT5A,TP53,TRIM71,TRIML2,ZRANB1	20	10	[Hematological Disease, Hematological System Development and Function, Organismal Injury and Abnormalities]

Supplementary Table 8: Differentially expressed transcripts by stage (1 vs. 2-4)

Symbol	Chromosome	logFC	adj.P.Val	Stage2-4 regulated
LOC101928525		2.49588	0.021429	UP
AQP1	7p14.3	0.806527	0.020073	UP
MRPL48	11q13.4	0.787001	0.021967	UP
ORC1	13q14.11, 1p32.3	0.75968	0.023403	UP
RECK	9p13.3	0.69914	0.038222	UP
GMPS	3q25.31	0.671895	0.03232	UP
EEF1E1	6p24.3	0.584998	0.021967	UP
HSPA1L	6p21.33	0.578013	0.031255	UP
TMPO	12q23.1	0.564157	0.038222	UP
C1orf131	1q42.2	0.559565	0.038222	UP
TARS1	5p13.3	0.483044	0.047793	UP
SAMD1	19p13.12	0.46228	0.01174	UP
MEA1	6p21.1	0.450845	0.024392	UP
MBOAT7	19q13.42	0.442596	0.046108	UP
NUP37	12q23.2	0.441611	0.037907	UP
NADK	1p36.33	0.436902	0.039812	UP
UXT	Xp11.23	0.420503	0.038222	UP
TCFL5	20q13.33	0.396335	0.048428	UP
IPO9	1q32.1	0.364512	0.03227	UP
B3GALNT1	3q26.1	0.362449	0.029984	UP
MRPL44	2q36.1	0.304409	0.038222	UP
FNBP4	11p11.2	-0.23617	0.036604	DOWN
L3HYPDH	14q23.1	-0.28685	0.038222	DOWN
USP4	3p21.31	-0.28921	0.038222	DOWN
APBB3	5q31.3	-0.30548	0.031428	DOWN
ST6GALNAC6	9q34.11	-0.32849	0.045179	DOWN

**ZNF433**	19p13.2	-0.35856	0.039812	DOWN
**RBM5**	3p21.31	-0.37143	0.01174	DOWN
**ZFP36L2**	2p21	-0.37758	0.048814	DOWN
**BDH2**	4q24	-0.39604	0.002812	DOWN
**LPCAT4**	15q14	-0.40032	0.046909	DOWN
**TNFRSF14**	1p36.32	-0.40534	0.038222	DOWN
**CCNL1**	3q21.3, 3q25.31	-0.40688	0.036358	DOWN
**THTPA**	14q11.2	-0.41599	0.001951	DOWN
**UVSSA**	4p16.3	-0.43426	0.022536	DOWN
**PDXDC2P-NPIPB14P**		-0.45233	0.031333	DOWN
**NISCH**	3p21.1	-0.46847	0.033451	DOWN
**ZNF449**	Xq26.3	-0.47595	0.017051	DOWN
**AHI1**	6q23.3	-0.48382	0.002409	DOWN
**ZNF846**	19p13.2	-0.49832	0.021967	DOWN
**PGAP1**	2q33.1	-0.51827	0.041939	DOWN
**IL11RA**	9p13.3	-0.52148	0.021967	DOWN
**PDE4DIPP2**	1p11.2	-0.53135	0.015365	DOWN
**ACCS**	11p11.2	-0.53291	0.021967	DOWN
**RHOU**	1q42.13	-0.55598	0.024392	DOWN
**JAM3**	11q25	-0.55866	0.021967	DOWN
**TBC1D9**	4q31.21	-0.56551	0.039058	DOWN
**DPYD**	1p21.3	-0.57263	0.021967	DOWN
**SCAPER**	15q24.3	-0.57556	0.03227	DOWN
**ZNF441**	19p13.2	-0.57581	0.028967	DOWN
**CDH26**	20q13.33	-0.59024	0.023639	DOWN
**ZNF749**	19q13.43	-0.59382	0.04801	DOWN
**RRN3P3**	16p12.2	-0.6007	0.038222	DOWN
**LOC100652768**		-0.60661	0.028695	DOWN

**ZNF880**	19q13.41	-0.60869	0.024659	DOWN
**TDP1**	14q32.11	-0.6389	0.038222	DOWN
**PDS5A**	4p14	-0.64195	0.03227	DOWN
**RPL26**	17p13.1	-0.65146	0.023639	DOWN
**SYNCRIP**	6q14.3	-0.65937	0.03227	DOWN
**UBE2E3**	2q31.3	-0.66777	0.021967	DOWN
**SHANK3**	22q13.33	-0.66825	0.031049	DOWN
**ACP1**	16p12.2, 2p25.3	-0.67269	0.038222	DOWN
**TMEM144**	4q32.1	-0.67699	0.021967	DOWN
**KCNAB3**	17p13.1	-0.67749	0.045179	DOWN
**PECR**	2q35	-0.6779	0.042749	DOWN
**EPHA4**	2q36.1	-0.67842	0.002812	DOWN
**ZNF525**	19q13.42	-0.68283	0.022554	DOWN
**LOC728392**		-0.68698	0.028967	DOWN
**SCAMP1**	5q14.1	-0.7012	0.038222	DOWN
**UBE2N**	12q22	-0.71161	0.038222	DOWN
**CCNB3**	Xp11.22	-0.71933	0.029984	DOWN
**EIF4EBP2**	10q22.1	-0.73113	0.040863	DOWN
**LRRC40**	1p31.1	-0.73707	0.029984	DOWN
**CACNA1F**	Xp11.23	-0.73882	0.042749	DOWN
**EBNA1BP2**	1p34.2	-0.74152	0.032531	DOWN
**H2BP2**	1q21.1	-0.75217	0.046603	DOWN
**STOML2**	9p13.3	-0.75225	0.030289	DOWN
**GEMIN6**	2p22.1	-0.76879	0.03177	DOWN
**NAA20**	20p11.23	-0.77524	0.010573	DOWN
**FLRT2**	14q31.3	-0.77761	0.021967	DOWN
**RPP21**	6p22.1	-0.7861	0.049416	DOWN
**TSN**	2q14.3	-0.79055	0.046603	DOWN

**DDX54**	12q24.13	-0.80128	0.033203	DOWN
**TGFBRAP1**	2q12.1-q12.2	-0.80188	0.022432	DOWN
**BRI3BP**	12q24.31	-0.81115	0.021967	DOWN
**PGAP4**	9q31.1	-0.81424	0.038222	DOWN
**NTRK1**	1q23.1	-0.81811	0.035889	DOWN
**SKP2**	5p13.2	-0.82192	0.015539	DOWN
**C1orf109**	1p34.3	-0.8249	0.041	DOWN
**TOR2A**	9q34.11	-0.83434	0.049649	DOWN
**UBA1**	Xp11.3	-0.84667	0.038222	DOWN
**EDA2R**	Xq12	-0.88817	0.023639	DOWN
**HCAR3**	12q24.31	-0.90099	0.035751	DOWN
**SFR1**	10q25.1	-0.90381	0.023639	DOWN
**COPA**	1q23.2	-0.9056	0.01174	DOWN
**SMIM13**	6p24.2	-0.91949	0.022912	DOWN
**MRPL37**	1p32.3	-0.95569	0.001951	DOWN
**SLC10A1**	14q24.1	-1.00211	0.041939	DOWN
**KIF5B**	10p11.22	-1.03549	0.015365	DOWN
**MT1M**	16q13	-1.26045	0.023639	DOWN
**FAM107A**	3p14.3-p14.2	-1.26211	0.038901	DOWN
**PLAAT5**	11q12.3	-1.29409	0.036326	DOWN
**HEPACAM**	11q24.2	-1.44193	0.01759	DOWN
**AGAP14P**	10q11.22	-1.46853	0.038222	DOWN
**RXFP4**	1q22	-1.58449	0.023639	DOWN
**SGCG**	13q12.12	-1.62691	0.042042	DOWN
**CA4**	17q23.1	-1.64612	0.038222	DOWN
**SYT9**	11p15.4	-1.71494	0.03227	DOWN
**CIDEA**	18p11.21	-1.77393	0.021967	DOWN
**NR2E3**	15q23	-1.79875	0.021967	DOWN

**CLCNKB**	1p36.13	-1.81163	0.038222	DOWN
**LOC283335**		-1.85752	0.032531	DOWN
**GABRA2**	4p12	-2.00561	0.035751	DOWN
**FAM71A**	1q32.3	-2.07376	0.015539	DOWN
**ADCY8**	8q24.22	-2.07812	0.046941	DOWN
**C22orf31**	22q12.1	-2.08832	0.038222	DOWN
**DBX2**	12q12	-2.10116	0.01759	DOWN
**RARA-AS1**	17q21.2	-2.11244	0.037595	DOWN
**ELANE**	19p13.3	-2.11465	0.03227	DOWN
**LOC100509620**		-2.11958	0.030074	DOWN
**FRMD1**	6q27	-2.12463	0.033451	DOWN
**SFRP5**	10q24.2	-2.13585	0.038222	DOWN
**LMX1A**	1q23.3	-2.1537	0.040594	DOWN
**TRDN**	6q22.31	-2.15996	0.01174	DOWN
**VWA5B1**	1p36.12	-2.19242	0.043329	DOWN
**CCL16**	17q12	-2.24862	0.038222	DOWN
**OR2B11**	1q44	-2.25536	0.03227	DOWN
**NPY2R**	4q32.1	-2.26668	0.03227	DOWN
**MS4A6E**	11q12.2	-2.2698	0.035751	DOWN
**CYP2C9**	10q23.33	-2.28347	0.03232	DOWN
**TMEFF2**	2q32.3	-2.34129	0.021967	DOWN
**COMMD4P1**	15q24.3	-2.34651	0.025051	DOWN
**CPA1**	7q32.2	-2.35045	0.021967	DOWN
**PSG11**	19q13.31	-2.40759	0.024392	DOWN
**APOL5**	22q12.3	-2.42176	0.021967	DOWN
**FBXO40**	3q13.33	-2.48974	0.015365	DOWN
**GLYAT**	11q12.1	-2.5117	0.001708	DOWN
**LINC01537**	11q13.4	-2.72672	0.009464	DOWN

**TMEM252**	9q21.11	-2.74847	0.002812	DOWN
**C14orf180**	14q32.33	-2.94152	0.001951	DOWN
**FAM217A**	6p25.2	-3.07724	0.000502	DOWN
**DEFB132**	20p13	-3.08826	0.001708	DOWN

Supplementary Table 9: Differentially expressed transcripts in tumors with early mortality (<2yr) versus late mortality (>2yr)

Symbol	Chromosome	logFC	adj.P.Val	> 2 years regulated
TMEM64	8q21.3	0.791673	0.046107	UP
POLR2B	4q12	0.64283	0.046748	UP
PIGS	17q11.2	0.571039	0.046107	UP
ZBP1	20q13.31	-0.79659	0.033456	DOWN
DAP	5p15.2	-0.79768	0.046911	DOWN
RITA1	12q24.13	-0.86335	0.043863	DOWN
GDAP1	8q21.11	-0.8905	0.035514	DOWN
SMAP1	6q13	-0.89819	0.046911	DOWN
PCDH19	Xq22.1	-0.92403	0.033456	DOWN
RNASEH2B	13q14.3	-0.92662	0.035514	DOWN
DYNLT3	Xp11.4	-0.9409	0.02052	DOWN
UBXN6	19p13	-0.97719	0.046748	DOWN
CD55	1q32.2	-0.98559	0.007946	DOWN
DHRS7	14q23.1	-0.99604	0.016232	DOWN
BORCS6	17p13.1	-1.00596	0.046107	DOWN
LCA5L	21q22.2	-1.01182	0.006317	DOWN
RHEX	1q32.1	-1.04766	0.046748	DOWN
UTP18	17q21.33	-1.11094	0.008547	DOWN
APPBP2	17q23.2	-1.12068	0.008547	DOWN
EPN1	19q13.42	-1.15331	0.008547	DOWN
SPRN	10q26.3	-1.22712	0.046911	DOWN

Supplementary Table 10: Differentially expressed transcripts by race

Symbol	Chromosome	logFC	adj.P.Val	Black regulated
TBC1D3C	17q12	3.764746	1.11E-06	UP
USP17L8	8p23.1	3.563138	1.92E-05	UP
SPINK2	4q12	3.036087	0.0008513	UP
FOLH1B	11q14.3	2.892109	0.0001152	UP
SPATA4	4q34.2	2.46282	0.0423109	UP
SYT5	19q13.42	2.398002	0.0455578	UP
GABRG3	15q12	2.350468	0.0381375	UP
UGT2B17	4q13.2	2.348177	0.0381375	UP
RNA5-8SN4	22 unplaced	2.324989	0.0402453	UP
HDHD5-AS1	22q11.1	2.241077	0.0372659	UP
SMIM1	1p36.32	2.171206	0.0419877	UP
WIF1	12q14.3	2.149975	0.0381375	UP
NCMAP	1p36.11	1.939958	0.0402453	UP
FAM107B	10p13	-0.7048	0.0055723	DOWN
NFYA	6p21.1	-0.70616	0.0321718	DOWN
LOC729737		-0.72284	0.0321718	DOWN
GALNT3	2q24.3	-0.76338	0.0372659	DOWN
SMG1P5	16p11.2	-0.79546	0.0002536	DOWN
GLT8D1	3p21.1	-0.80123	0.0352562	DOWN
TMEM184C	4q31.23	-0.81175	0.0402453	DOWN
GSTT2B	22q11.23	-0.85129	0.0241766	DOWN
SPATC1L	21q22.3	-1.44945	0.0027598	DOWN
USP17L1	8p23.1	-2.63954	0.0018539	DOWN
USP17L4	8p23.1	-2.70559	0.0001152	DOWN
FSHR	2p16.3	-3.13645	0.000477	DOWN
LOC284581		-4.18177	2.86E-08	DOWN

Supplementary Table 11: Ingenuity Pathway Analysis of transcripts differentially expressed by race

Ingenuity Canonical Pathways	-log(p)	Ratio	Molecules
Xenobiotic Metabolism General Signaling Pathway	2.21	0.014	GSTT2/GSTT2B,UGT2B17
Xenobiotic Metabolism CAR Signaling Pathway	1.95	0.0103	GSTT2/GSTT2B,UGT2B17
Xenobiotic Metabolism PXR Signaling Pathway	1.95	0.0103	GSTT2/GSTT2B,UGT2B17
Glutathione Redox Reactions I	1.64	0.0357	GSTT2/GSTT2B
Xenobiotic Metabolism Signaling	1.61	0.0068	GSTT2/GSTT2B,UGT2B17
Glutathione-mediated Detoxification	1.52	0.027	GSTT2/GSTT2B
Thyroid Hormone Metabolism II (via Conjugation and/or Degradation)	1.47	0.0244	UGT2B17
Nicotine Degradation III	1.32	0.0169	UGT2B17
GADD45 Signaling	1.31	0.0167	NFYA
Melatonin Degradation I	1.29	0.0159	UGT2B17
Nicotine Degradation II	1.27	0.0149	UGT2B17
Superpathway of Melatonin Degradation	1.26	0.0147	UGT2B17
Serotonin Degradation	1.23	0.0139	UGT2B17
Xenobiotic Metabolism AHR Signaling Pathway	1.16	0.0115	GSTT2/GSTT2B
Apelin Adipocyte Signaling Pathway	1.14	0.011	GSTT2/GSTT2B
Neuroprotective Role of THOP1 in Alzheimer's Disease	1.02	0.00826	NFYA
Gαs Signaling	1	0.00794	FSHR
GABA Receptor Signaling	0.982	0.00758	GABRG3
SNARE Signaling Pathway	0.969	0.00735	SYT5
GABAergic Receptor Signaling Pathway (Enhanced)	0.96	0.00719	GABRG3
Ovarian Cancer Signaling	0.908	0.00633	FSHR

**Aryl Hydrocarbon Receptor Signaling**	0.905	0.00629	GSTT2/GSTT2B
**HOTAIR Regulatory Pathway**	0.895	0.00613	WIF1
**Ribonucleotide Reductase Signaling Pathway**	0.878	0.00588	NFYA
**WNT/β-catenin Signaling**	0.869	0.00575	WIF1
**IL-33 Signaling Pathway**	0.844	0.00541	USP17L2 (includes others)
**Human Embryonic Stem Cell Pluripotency**	0.811	0.00498	NFYA
**Gustation Pathway**	0.809	0.00495	GABRG3
**Role of Osteoblasts, Osteoclasts and Chondrocytes in Rheumatoid Arthritis**	0.76	0.00439	WIF1
**Neurovascular Coupling Signaling Pathway**	0.754	0.00431	GABRG3
**cAMP-mediated signaling**	0.747	0.00424	FSHR
**NRF2-mediated Oxidative Stress Response**	0.745	0.00422	GSTT2/GSTT2B
**Role of Osteoblasts in Rheumatoid Arthritis Signaling Pathway**	0.734	0.0041	WIF1
**LPS/IL-1 Mediated Inhibition of RXR Function**	0.713	0.00389	GSTT2/GSTT2B
**Synaptogenesis Signaling Pathway**	0.635	0.00317	SYT5
**Neuroinflammation Signaling Pathway**	0.632	0.00315	GABRG3
**Glutaminergic Receptor Signaling Pathway (Enhanced)**	0.623	0.00308	GABRG3
**Role of Macrophages, Fibroblasts and Endothelial Cells in Rheumatoid Arthritis**	0.615	0.00301	WIF1
**Mitochondrial Dysfunction**	0.6	0.0029	GSTT2/GSTT2B
**Breast Cancer Regulation by Stathmin1**	0.405	0.00168	FSHR
**CREB Signaling in Neurons**	0.397	0.00165	FSHR
**Phagosome Formation**	0.353	0.00144	FSHR
**G-Protein Coupled Receptor Signaling**	0.349	0.00142	FSHR
**S100 Family Signaling Pathway**	0.32	0.0013	FSHR
**FAK Signaling**	0.231	0.000961	FSHR

Supplementary Table 12: gene network analysis of transcripts differentially expressed by race

ID	Molecules in Network	Score	Focus Molecules	Top Diseases and Functions
1	ANKS6,APP,APPL2,ATP6V1D,BCL2L13,CGB3 (includes others), CLIC3,GABRA4,GABRG3,GCHFR,GLT8D1,GTF3C6, ICAM3,LAMP1,LGMN, PCDHGC5,PPP2R5C,PRTFDC1,PVR,Rab5,RBMY1A1 (includes others),SIAH1,SMIM1,SOD1,SPATA4,SPATC1L,SPINK2, Synaptotagmin, SYT5,TBC1D3 (includes others),TMEM106B,TMEM184C,TRAPPC2L,YWHAE,ZDHHC15	23	9	[Cell Death and Survival, Cell Morphology, Nervous System Development and Function]
2	ADK,APP,BMP15,CD55,CGB3 (includes others), CRADD,EGR1,ESPN,FAM107B,FSHR,GALE,GALNT3, GAMT,GCHFR,GSTT2/GSTT2B,HSD3B1,IL4,L GMN,LHB,MMP2,MT-RNR2,MYC,NFYA,PKNOX1,PLA1A, RIGI,SNX14,Sod,SRC,TUG1,UGT1A1,UGT2B17,USP17: RCE1, CDC25A, DDX58, IFIH1,USP17L2 (includes others),WIF1	20	8	[Lipid Metabolism, Small Molecule Biochemistry, Vitamin and Mineral Metabolism]
3	FOLH1B,HNRNPL	3	1	[Cell-mediated Immune Response, Cellular Development, Cellular Function and Maintenance]
4	SMG1P5-203,SNCA	3	1	[Cell Death and Survival, Cell Morphology, Cell-To-Cell Signaling and Interaction]
5	EGR2,NCMAP,ZNF106	3	1	[Cellular Development, Cellular Growth and Proliferation, Nervous System Development and Function]

Supplementary Table 13: Ancestry Analysis

sample_id	number_SNPs	_missing	missing_percent	C_W_African	N_African	E_Asian	C_Asian	Medterranean	W_N_European	PacificIslander	Native_American	Self-reported Race
TNBC0415	161	22	0.137	0.002	0.004	0.002	0.004	0.014	0.971	0.002	0.002	Black
TNBC0414	161	18	0.112	0.004	0.008	0.005	0.011	0.045	0.916	0.007	0.006	Black
TNBC0394	161	15	0.093	0.482	0.028	0.004	0.038	0.041	0.397	0.006	0.003	Black
TNBC0072	161	10	0.062	0.6	0.029	0.003	0.016	0.047	0.296	0.005	0.004	Black
TNBC0253	161	31	0.193	0.511	0.069	0.007	0.035	0.046	0.286	0.008	0.038	Black
TNBC0712	161	71	0.441	0.023	0.028	0.353	0.032	0.067	0.223	0.005	0.271	Black
TNBC0897	161	13	0.081	0.403	0.071	0.01	0.022	0.113	0.221	0.131	0.031	Black
TNBC0850	161	34	0.211	0.358	0.049	0.084	0.02	0.12	0.221	0.008	0.139	Black
TNBC0325	161	25	0.155	0.67	0.015	0.022	0.034	0.017	0.217	0.005	0.02	Black
TNBC0638	161	24	0.149	0.748	0.009	0.003	0.005	0.012	0.21	0.003	0.011	Black
TNBC0705	161	5	0.031	0.63	0.031	0.016	0.025	0.055	0.197	0.014	0.031	Black
TNBC0230	161	41	0.255	0.426	0.149	0.013	0.063	0.125	0.184	0.008	0.031	Black
TNBC0760	161	15	0.093	0.742	0.009	0.007	0.031	0.013	0.183	0.004	0.011	Black
TNBC0363	161	50	0.311	0.601	0.023	0.08	0.053	0.039	0.172	0.006	0.027	Black
TNBC0161	161	13	0.081	0.361	0.038	0.034	0.099	0.243	0.17	0.022	0.034	Black
TNBC0343	161	55	0.342	0.615	0.033	0.087	0.038	0.014	0.158	0.005	0.049	Black
TNBC1037	161	19	0.118	0.553	0.128	0.006	0.018	0.113	0.156	0.011	0.016	Black
TNBC0876	161	10	0.062	0.575	0.047	0.014	0.027	0.179	0.141	0.005	0.013	Black
TNBC1028	161	5	0.031	0.694	0.06	0.006	0.056	0.034	0.126	0.005	0.02	Black
TNBC0682	161	31	0.193	0.801	0.018	0.004	0.021	0.024	0.115	0.005	0.013	Black
TNBC0356	161	13	0.081	0.665	0.115	0.007	0.073	0.019	0.11	0.005	0.006	Black
TNBC0364	161	24	0.149	0.657	0.044	0.003	0.021	0.162	0.105	0.005	0.004	Black
TNBC0614	161	27	0.168	0.643	0.099	0.005	0.041	0.099	0.101	0.007	0.005	Black
TNBC0150	161	20	0.124	0.715	0.026	0.019	0.084	0.016	0.098	0.006	0.036	Black
TNBC0732	161	34	0.211	0.76	0.027	0.006	0.031	0.075	0.091	0.003	0.007	Black

TNBC0968	161	7	0.043	0.821	0.021	0.005	0.04	0.014	0.085	0.008	0.005	Black
TNBC1083	161	16	0.099	0.765	0.016	0.008	0.012	0.07	0.079	0.038	0.012	Black
TNBC1027	161	10	0.062	0.804	0.013	0.005	0.016	0.072	0.077	0.007	0.007	Black
TNBC0936	161	16	0.099	0.874	0.007	0.006	0.013	0.005	0.074	0.017	0.005	Black
TNBC1038	161	22	0.137	0.704	0.087	0.01	0.034	0.029	0.071	0.012	0.052	Black
TNBC0572	161	17	0.106	0.845	0.007	0.011	0.007	0.029	0.07	0.011	0.021	Black
TNBC0821	161	25	0.155	0.884	0.024	0.003	0.012	0.006	0.067	0.002	0.002	Black
TNBC0794	161	8	0.05	0.815	0.013	0.023	0.02	0.025	0.067	0.029	0.008	Black
TNBC0096	161	10	0.062	0.741	0.019	0.009	0.058	0.013	0.064	0.04	0.056	Black
TNBC0606	161	15	0.093	0.877	0.021	0.005	0.009	0.013	0.058	0.004	0.013	Black
TNBC0264	161	51	0.317	0.719	0.073	0.007	0.02	0.057	0.055	0.009	0.061	Black
TNBC0173	161	47	0.292	0.714	0.059	0.007	0.045	0.108	0.052	0.009	0.007	Black
TNBC1047	161	14	0.087	0.809	0.017	0.004	0.014	0.071	0.051	0.026	0.008	Black
TNBC0949	161	7	0.043	0.715	0.019	0.006	0.081	0.1	0.051	0.01	0.018	Black
TNBC0599	161	14	0.087	0.726	0.029	0.007	0.019	0.145	0.05	0.013	0.01	Black
TNBC0147	161	57	0.354	0.611	0.035	0.015	0.047	0.219	0.049	0.006	0.018	Black
TNBC1013	161	3	0.019	0.819	0.012	0.005	0.016	0.064	0.048	0.015	0.021	Black
TNBC1049	161	5	0.031	0.843	0.025	0.007	0.021	0.039	0.047	0.005	0.012	Black
TNBC0258	161	12	0.075	0.792	0.011	0.022	0.071	0.041	0.047	0.01	0.007	Black
TNBC0408	161	67	0.416	0.503	0.218	0.049	0.028	0.12	0.047	0.027	0.007	Black
TNBC0667	161	8	0.05	0.772	0.047	0.021	0.014	0.036	0.046	0.059	0.005	Black
TNBC0647	161	10	0.062	0.837	0.03	0.002	0.009	0.038	0.045	0.006	0.032	Black
TNBC1020	161	3	0.019	0.802	0.021	0.005	0.01	0.099	0.045	0.012	0.006	Black
TNBC0229	161	40	0.248	0.609	0.043	0.011	0.068	0.132	0.045	0.042	0.051	Black
TNBC0884	161	6	0.037	0.847	0.015	0.004	0.035	0.011	0.044	0.019	0.025	Black
TNBC0940	161	19	0.118	0.875	0.044	0.003	0.01	0.009	0.043	0.005	0.011	Black
TNBC0706	161	14	0.087	0.731	0.059	0.01	0.092	0.031	0.041	0.005	0.032	Black

TNBC1021	161	10	0.062	0.833	0.031	0.006	0.049	0.016	0.039	0.007	0.02	Black
TNBC0516	161	23	0.143	0.781	0.022	0.012	0.046	0.008	0.038	0.009	0.085	Black
TNBC0974	161	3	0.019	0.672	0.062	0.009	0.049	0.091	0.038	0.049	0.03	Black
TNBC0207	161	30	0.186	0.793	0.029	0.008	0.034	0.06	0.037	0.008	0.032	Black
TNBC0906	161	5	0.031	0.717	0.045	0.006	0.005	0.148	0.036	0.016	0.026	Black
TNBC0611	161	4	0.025	0.785	0.025	0.004	0.026	0.091	0.034	0.032	0.003	Black
TNBC0644	161	35	0.217	0.58	0.114	0.005	0.013	0.242	0.033	0.008	0.005	Black
TNBC0984	161	14	0.087	0.717	0.058	0.013	0.035	0.082	0.032	0.01	0.054	Black
TNBC0093	161	39	0.242	0.798	0.036	0.008	0.023	0.067	0.031	0.008	0.028	Black
TNBC0510	161	23	0.143	0.755	0.049	0.009	0.028	0.105	0.03	0.011	0.012	Black
TNBC0181	161	22	0.137	0.62	0.137	0.007	0.024	0.059	0.03	0.113	0.011	Black
TNBC1066	161	7	0.043	0.725	0.018	0.085	0.034	0.088	0.029	0.013	0.006	Black
TNBC0074	161	10	0.062	0.816	0.027	0.009	0.019	0.076	0.028	0.007	0.019	Black
TNBC0541	161	12	0.075	0.848	0.013	0.011	0.014	0.023	0.027	0.003	0.061	Black
TNBC0710	161	10	0.062	0.845	0.013	0.008	0.013	0.043	0.027	0.003	0.048	Black
TNBC0427	161	38	0.236	0.642	0.031	0.082	0.067	0.136	0.027	0.007	0.009	Black
TNBC0969	161	3	0.019	0.858	0.033	0.003	0.008	0.067	0.026	0.002	0.003	Black
TNBC0841	161	11	0.068	0.833	0.022	0.017	0.016	0.029	0.026	0.031	0.026	Black
TNBC0376	161	4	0.025	0.845	0.017	0.021	0.017	0.034	0.025	0.007	0.033	Black
TNBC0977	161	16	0.099	0.887	0.011	0.011	0.034	0.013	0.023	0.006	0.014	Black
TNBC0184	161	19	0.118	0.91	0.011	0.003	0.007	0.038	0.022	0.005	0.005	Black
TNBC0177	161	17	0.106	0.769	0.06	0.005	0.021	0.097	0.022	0.004	0.022	Black
TNBC0186	161	39	0.242	0.836	0.045	0.009	0.045	0.028	0.021	0.007	0.011	Black
TNBC1008	161	19	0.118	0.831	0.031	0.008	0.026	0.069	0.02	0.004	0.012	Black
TNBC0626	161	27	0.168	0.833	0.043	0.009	0.03	0.057	0.019	0.004	0.005	Black
TNBC0098	161	38	0.236	0.813	0.023	0.04	0.018	0.066	0.019	0.008	0.014	Black
TNBC0437	161	11	0.068	0.772	0.044	0.004	0.019	0.122	0.019	0.01	0.009	Black

TNBC0871	161	17	0.106	0.714	0.111	0.014	0.036	0.08	0.019	0.017	0.009	Black
TNBC0651	161	23	0.143	0.372	0.197	0.013	0.073	0.29	0.019	0.009	0.027	Black
TNBC0687	161	6	0.037	0.886	0.013	0.004	0.007	0.058	0.017	0.01	0.006	Black
TNBC0739	161	29	0.18	0.834	0.038	0.007	0.011	0.033	0.017	0.019	0.04	Black
TNBC0942	161	25	0.155	0.829	0.048	0.006	0.04	0.033	0.017	0.009	0.017	Black
TNBC0922	161	10	0.062	0.79	0.021	0.005	0.015	0.138	0.017	0.008	0.007	Black
TNBC0188	161	3	0.019	0.895	0.025	0.003	0.008	0.042	0.016	0.007	0.005	Black
TNBC1112	161	4	0.025	0.742	0.063	0.009	0.038	0.05	0.016	0.021	0.061	Black
TNBC0700	161	24	0.149	0.856	0.038	0.004	0.007	0.061	0.015	0.006	0.012	Black
TNBC0583	161	23	0.143	0.828	0.057	0.033	0.014	0.031	0.014	0.006	0.016	Black
TNBC0189	161	12	0.075	0.852	0.024	0.009	0.022	0.04	0.013	0.018	0.021	Black
TNBC0823	161	3	0.019	0.769	0.056	0.006	0.047	0.084	0.013	0.002	0.023	Black
TNBC0721	161	110	0.683	0.477	0.013	0.416	0.016	0.014	0.013	0.005	0.047	Black
TNBC0255	161	21	0.13	0.946	0.006	0.003	0.003	0.016	0.012	0.003	0.012	Black
TNBC0369	161	14	0.087	0.898	0.007	0.007	0.011	0.003	0.012	0.006	0.055	Black
TNBC0981	161	17	0.106	0.876	0.026	0.004	0.01	0.044	0.012	0.007	0.019	Black
TNBC0930	161	10	0.062	0.804	0.026	0.009	0.023	0.051	0.012	0.009	0.066	Black
TNBC0619	161	10	0.062	0.787	0.053	0.007	0.071	0.055	0.012	0.006	0.008	Black
TNBC0839	161	21	0.13	0.646	0.039	0.004	0.011	0.283	0.012	0.003	0.003	Black
TNBC0925	161	15	0.093	0.816	0.054	0.01	0.019	0.055	0.011	0.005	0.03	Black
TNBC0899	161	6	0.037	0.909	0.01	0.005	0.017	0.017	0.01	0.019	0.013	Black
TNBC1101	161	7	0.043	0.888	0.016	0.012	0.015	0.016	0.009	0.012	0.032	Black
TNBC1084	161	14	0.087	0.882	0.011	0.022	0.036	0.008	0.009	0.006	0.026	Black
TNBC0935	161	9	0.056	0.659	0.2	0.007	0.031	0.055	0.009	0.032	0.007	Black
TNBC0990	161	6	0.037	0.905	0.013	0.008	0.009	0.022	0.008	0.005	0.031	Black
TNBC0435	161	21	0.13	0.876	0.018	0.007	0.021	0.009	0.008	0.006	0.054	Black
TNBC1087	161	5	0.031	0.941	0.027	0.004	0.006	0.01	0.007	0.004	0.002	Black

TNBC0097	161	7	0.043	0.91	0.018	0.004	0.013	0.017	0.007	0.014	0.016	Black
TNBC0482	161	4	0.025	0.896	0.008	0.02	0.014	0.013	0.007	0.011	0.031	Black
TNBC0975	161	25	0.155	0.865	0.012	0.011	0.008	0.019	0.007	0.005	0.073	Black
TNBC0460	161	2	0.012	0.833	0.011	0.008	0.007	0.015	0.007	0.012	0.108	Black
TNBC0303	161	7	0.043	0.814	0.013	0.022	0.025	0.027	0.007	0.014	0.078	Black
TNBC0917	161	8	0.05	0.929	0.006	0.015	0.01	0.007	0.006	0.021	0.006	Black
TNBC0347	161	10	0.062	0.944	0.011	0.004	0.011	0.007	0.005	0.013	0.004	Black
TNBC0403	161	8	0.05	0.915	0.007	0.018	0.019	0.005	0.004	0.023	0.009	Black
TNBC0218	161	26	0.161	0.888	0.008	0.012	0.013	0.009	0.004	0.008	0.057	Black
TNBC1115	161	5	0.031	0.959	0.006	0.003	0.004	0.007	0.003	0.003	0.016	Black
TNBC0160	161	18	0.112	0.9	0.006	0.015	0.011	0.006	0.003	0.004	0.054	Black
TNBC0301	161	27	0.168	0.89	0.005	0.013	0.013	0.004	0.003	0.005	0.067	Black
TNBC0346	161	23	0.143	0.002	0.006	0.002	0.006	0.02	0.96	0.002	0.002	White
TNBC0929	161	14	0.087	0.003	0.006	0.004	0.008	0.012	0.959	0.004	0.004	White
TNBC0438	161	8	0.05	0.004	0.011	0.002	0.006	0.015	0.958	0.003	0.002	White
TNBC0501	161	24	0.149	0.003	0.012	0.004	0.01	0.015	0.952	0.003	0.002	White
TNBC0256	161	10	0.062	0.002	0.008	0.005	0.009	0.037	0.935	0.003	0.002	White
TNBC0657	161	21	0.13	0.006	0.022	0.002	0.006	0.033	0.928	0.002	0.002	White
TNBC0868	161	49	0.304	0.004	0.011	0.03	0.012	0.009	0.928	0.002	0.004	White
TNBC0182	161	16	0.099	0.009	0.031	0.002	0.009	0.016	0.927	0.004	0.004	White
TNBC0436	161	7	0.043	0.008	0.029	0.003	0.011	0.017	0.927	0.002	0.003	White
TNBC0221	161	24	0.149	0.014	0.03	0.006	0.008	0.017	0.919	0.002	0.005	White
TNBC0709	161	26	0.161	0.002	0.007	0.007	0.007	0.044	0.917	0.003	0.013	White
TNBC1040	161	13	0.081	0.01	0.022	0.003	0.011	0.035	0.915	0.002	0.002	White
TNBC1074	161	5	0.031	0.005	0.019	0.004	0.01	0.04	0.915	0.005	0.003	White
TNBC0793	161	12	0.075	0.014	0.028	0.004	0.009	0.029	0.913	0.002	0.003	White
TNBC1082	161	7	0.043	0.007	0.027	0.003	0.015	0.024	0.911	0.008	0.003	White

TNBC0175	161	16	0.099	0.004	0.011	0.005	0.026	0.034	0.91	0.008	0.004	White
TNBC0252	161	22	0.137	0.004	0.018	0.003	0.008	0.053	0.91	0.003	0.002	White
TNBC0502	161	9	0.056	0.012	0.033	0.003	0.006	0.032	0.908	0.004	0.004	White
TNBC0420	161	21	0.13	0.011	0.022	0.003	0.008	0.02	0.908	0.026	0.002	White
TNBC0895	161	7	0.043	0.004	0.013	0.006	0.029	0.032	0.908	0.004	0.004	White
TNBC0561	161	10	0.062	0.008	0.034	0.003	0.008	0.038	0.907	0.002	0.002	White
TNBC1056	161	14	0.087	0.022	0.029	0.003	0.011	0.026	0.904	0.003	0.002	White
TNBC0849	161	7	0.043	0.013	0.041	0.002	0.008	0.028	0.903	0.002	0.002	White
TNBC0764	161	24	0.149	0.013	0.046	0.002	0.006	0.029	0.898	0.002	0.003	White
TNBC0654	161	15	0.093	0.008	0.04	0.003	0.013	0.035	0.894	0.004	0.002	White
TNBC0653	161	13	0.081	0.029	0.033	0.005	0.007	0.014	0.89	0.016	0.007	White
TNBC0265	161	6	0.037	0.012	0.063	0.002	0.006	0.021	0.889	0.004	0.003	White
TNBC0972	161	12	0.075	0.01	0.056	0.003	0.005	0.032	0.889	0.002	0.002	White
TNBC0592	161	27	0.168	0.002	0.009	0.004	0.015	0.068	0.883	0.004	0.013	White
TNBC0979	161	6	0.037	0.002	0.02	0.006	0.024	0.056	0.882	0.005	0.005	White
TNBC1089	161	13	0.081	0.023	0.048	0.003	0.005	0.034	0.879	0.006	0.002	White
TNBC0810	161	22	0.137	0.006	0.045	0.006	0.011	0.041	0.876	0.01	0.004	White
TNBC0348	161	8	0.05	0.025	0.046	0.003	0.012	0.034	0.873	0.004	0.003	White
TNBC0062	161	8	0.05	0.007	0.051	0.003	0.006	0.054	0.873	0.003	0.003	White
TNBC0320	161	27	0.168	0.012	0.056	0.003	0.015	0.035	0.872	0.005	0.003	White
TNBC0328	161	11	0.068	0.038	0.058	0.003	0.005	0.02	0.871	0.003	0.003	White
TNBC0331	161	26	0.161	0.003	0.014	0.062	0.016	0.019	0.87	0.009	0.007	White
TNBC1077	161	22	0.137	0.002	0.014	0.003	0.012	0.091	0.87	0.006	0.002	White
TNBC0720	161	13	0.081	0.008	0.04	0.011	0.019	0.039	0.869	0.002	0.012	White
TNBC0180	161	32	0.199	0.014	0.08	0.004	0.01	0.022	0.866	0.003	0.002	White
TNBC0497	161	11	0.068	0.008	0.054	0.002	0.007	0.062	0.862	0.003	0.002	White
TNBC0670	161	18	0.112	0.04	0.031	0.008	0.015	0.025	0.859	0.008	0.013	White

TNBC0973	161	22	0.137	0.009	0.058	0.002	0.011	0.055	0.857	0.006	0.002	White
TNBC0665	161	15	0.093	0.029	0.032	0.004	0.011	0.056	0.854	0.009	0.004	White
TNBC0082	161	7	0.043	0.021	0.039	0.041	0.011	0.033	0.848	0.004	0.003	White
TNBC0714	161	9	0.056	0.034	0.071	0.003	0.011	0.029	0.846	0.003	0.003	White
TNBC0390	161	25	0.155	0.025	0.052	0.008	0.013	0.037	0.846	0.015	0.004	White
TNBC0867	161	21	0.13	0.009	0.037	0.002	0.005	0.097	0.845	0.002	0.001	White
TNBC0126	161	18	0.112	0.003	0.017	0.002	0.018	0.108	0.845	0.004	0.004	White
TNBC0259	161	7	0.043	0.008	0.016	0.014	0.053	0.039	0.844	0.005	0.022	White
TNBC0430	161	12	0.075	0.03	0.069	0.003	0.014	0.033	0.843	0.005	0.002	White
TNBC0755	161	10	0.062	0.004	0.013	0.009	0.042	0.06	0.842	0.021	0.008	White
TNBC0474	161	8	0.05	0.008	0.102	0.012	0.01	0.014	0.84	0.002	0.012	White
TNBC0419	161	30	0.186	0.069	0.024	0.014	0.015	0.021	0.839	0.009	0.009	White
TNBC0800	161	7	0.043	0.021	0.08	0.005	0.015	0.017	0.837	0.003	0.021	White
TNBC0902	161	13	0.081	0.011	0.042	0.048	0.013	0.038	0.833	0.004	0.01	White
TNBC1007	161	11	0.068	0.008	0.063	0.003	0.008	0.079	0.831	0.003	0.004	White
TNBC0580	161	7	0.043	0.012	0.073	0.003	0.008	0.068	0.83	0.003	0.003	White
TNBC0499	161	11	0.068	0.012	0.057	0.001	0.004	0.101	0.822	0.002	0.001	White
TNBC0536	161	8	0.05	0.011	0.058	0.001	0.004	0.101	0.821	0.002	0.001	White
TNBC0933	161	12	0.075	0.017	0.078	0.008	0.034	0.035	0.819	0.005	0.005	White
TNBC0094	161	13	0.081	0.009	0.034	0.003	0.005	0.115	0.819	0.01	0.003	White
TNBC0956	161	9	0.056	0.017	0.084	0.004	0.012	0.06	0.818	0.004	0.002	White
TNBC0240	161	34	0.211	0.015	0.049	0.002	0.007	0.105	0.818	0.002	0.002	White
TNBC0852	161	9	0.056	0.012	0.106	0.002	0.007	0.051	0.818	0.002	0.002	White
TNBC0918	161	7	0.043	0.008	0.077	0.009	0.031	0.051	0.813	0.005	0.006	White
TNBC0735	161	18	0.112	0.017	0.086	0.003	0.006	0.076	0.807	0.003	0.002	White
TNBC0326	161	14	0.087	0.004	0.023	0.007	0.027	0.129	0.801	0.006	0.004	White
TNBC0458	161	17	0.106	0.012	0.123	0.003	0.006	0.053	0.8	0.002	0.002	White

TNBC0 238	161	4	0.025	0.012	0.152	0.003	0.008	0.032	0.786	0.004	0.004	White
TNBC0 216	161	15	0.093	0.005	0.052	0.004	0.021	0.125	0.78	0.01	0.003	White
TNBC0 529	161	13	0.081	0.002	0.005	0.003	0.013	0.199	0.772	0.003	0.002	White
TNBC0 496	161	21	0.13	0.008	0.132	0.008	0.018	0.054	0.768	0.009	0.004	White
TNBC0 508	161	23	0.143	0.021	0.109	0.042	0.011	0.047	0.765	0.003	0.003	White
TNBC0 208	161	26	0.161	0.005	0.023	0.005	0.024	0.157	0.765	0.017	0.004	White
TNBC0 863	161	11	0.068	0.007	0.018	0.005	0.048	0.147	0.764	0.007	0.004	White
TNBC0 404	161	5	0.031	0.018	0.144	0.007	0.021	0.05	0.735	0.006	0.018	White
TNBC0 534	161	31	0.193	0.01	0.125	0.002	0.009	0.126	0.725	0.002	0.001	White
TNBC0 412	161	12	0.075	0.007	0.042	0.012	0.015	0.178	0.724	0.011	0.012	White
TNBC0 759	161	14	0.087	0.024	0.197	0.003	0.007	0.041	0.721	0.004	0.002	White
TNBC0 060	161	15	0.093	0.005	0.099	0.007	0.018	0.154	0.711	0.003	0.003	White
TNBC0 010	161	23	0.143	0.017	0.07	0.002	0.009	0.196	0.7	0.004	0.002	White
TNBC0 151	161	43	0.267	0.035	0.175	0.026	0.013	0.054	0.691	0.004	0.003	White
TNBC0 134	161	32	0.199	0.005	0.026	0.039	0.013	0.226	0.685	0.003	0.004	White
TNBC0 187	161	21	0.13	0.009	0.083	0.005	0.06	0.123	0.684	0.013	0.022	White
TNBC0 870	161	6	0.037	0.074	0.2	0.003	0.009	0.026	0.683	0.003	0.002	White
TNBC0 083	161	18	0.112	0.004	0.029	0.002	0.009	0.268	0.682	0.004	0.002	White
TNBC0 649	161	7	0.043	0.006	0.018	0.005	0.012	0.264	0.678	0.013	0.004	White
TNBC0 761	161	26	0.161	0.009	0.046	0.004	0.01	0.263	0.658	0.004	0.007	White
TNBC0 646	161	27	0.168	0.017	0.06	0.101	0.02	0.145	0.64	0.012	0.004	White
TNBC0 059	161	24	0.149	0.015	0.181	0.005	0.025	0.122	0.64	0.007	0.005	White
TNBC0 171	161	39	0.242	0.009	0.096	0.008	0.015	0.215	0.632	0.007	0.017	White
TNBC0 888	161	28	0.174	0.01	0.165	0.003	0.029	0.17	0.608	0.012	0.003	White
TNBC0 319	161	17	0.106	0.01	0.139	0.003	0.015	0.246	0.581	0.003	0.003	White
TNBC1 060	161	14	0.087	0.008	0.033	0.019	0.022	0.352	0.533	0.025	0.009	White
TNBC0 413	161	8	0.05	0.081	0.122	0.02	0.039	0.178	0.526	0.014	0.02	White

TNBC0485	161	9	0.056	0.118	0.117	0.018	0.019	0.206	0.492	0.009	0.02	White
TNBC0664	161	46	0.286	0.007	0.009	0.195	0.016	0.029	0.487	0.018	0.24	White
TNBC0944	161	24	0.149	0.01	0.111	0.003	0.023	0.373	0.468	0.009	0.003	White
TNBC0163	161	69	0.429	0.008	0.022	0.006	0.013	0.476	0.462	0.008	0.004	White
TNBC0874	161	5	0.031	0.033	0.453	0.003	0.02	0.143	0.342	0.005	0.002	White
TNBC0669	161	8	0.05	0.003	0.054	0.503	0.028	0.061	0.336	0.008	0.006	White
TNBC0366	161	45	0.28	0.009	0.097	0.075	0.027	0.486	0.295	0.006	0.005	White
TNBC0239	161	33	0.205	0.012	0.295	0.011	0.085	0.271	0.293	0.026	0.008	White
TNBC0209	161	12	0.075	0.026	0.081	0.007	0.018	0.638	0.207	0.01	0.012	White
TNBC0844	161	20	0.124	0.137	0.035	0.004	0.015	0.706	0.097	0.005	0.002	White
TNBC0317	161	9	0.056	0.037	0.555	0.004	0.036	0.306	0.05	0.009	0.003	White
TNBC0441	161	48	0.298	0.008	0.068	0.006	0.039	0.449	0.047	0.007	0.378	White
TNBC0610	161	7	0.043	0.825	0.022	0.008	0.05	0.017	0.033	0.009	0.036	White
TNBC0480	161	5	0.031	0.678	0.051	0.006	0.039	0.14	0.033	0.037	0.016	White
TNBC1079	161	11	0.068	0.007	0.316	0.015	0.012	0.036	0.021	0.015	0.578	White

Supplementary Table 14: CIBERSORTx results in TNBC-type classified, unclassified and excluded tumors

	TNBC Type									
	All	BL1	BL2	IM	LAR	M	MSL	Unclassified	Excluded	p-value
B cells naive	0.0310	0.0328	0.0226	0.0382	0.0552	0.0087	0.0239	0.0141	0.0406	0.0009
B cells memory	0.0000	0.0000	0.0002	0.0000	0.0079	0.0042	0.0000	0.0208	0.0090	0.0196
Plasma cells	0.0000	0.0000	0.0000	0.0017	0.0000	0.0000	0.0014	0.0000	0.0000	0.0078
T cells CD8	0.0175	0.0120	0.0036	0.0207	0.0305	0.0000	0.0091	0.0172	0.0093	0.0689
T cells CD4 naive	0.0000	0.0000	0.0000	0.0000	0.0000	0.0000	0.0000	0.0000	0.0000	0.7083
T cells CD4 memory resting	0.2597	0.2498	0.2643	0.2511	0.3170	0.3218	0.2521	0.1785	0.2613	0.0001
T cells CD4 memory activated	0.0238	0.0267	0.0337	0.0004	0.0461	0.0277	0.0000	0.0178	0.0177	<.0001
T cells follicular helper	0.0346	0.0592	0.0366	0.0084	0.0464	0.0219	0.0401	0.0074	0.0536	<.0001
T cells regulatory (Tregs)	0.0000	0.0000	0.0000	0.0000	0.0000	0.0000	0.0000	0.0000	0.0000	0.0423
T cells gamma delta	0.0000	0.0000	0.0000	0.0000	0.0000	0.0000	0.0000	0.0000	0.0000	0.1311
NK cells resting	0.0490	0.0411	0.0597	0.0472	0.0544	0.0651	0.0533	0.0443	0.0413	0.2667
NK cells activated	0.0000	0.0000	0.0000	0.0000	0.0000	0.0000	0.0000	0.0000	0.0000	0.0004
Monocytes	0.0320	0.0195	0.0182	0.0664	0.0247	0.0419	0.0378	0.0467	0.0249	<.0001
Macrophages M0	0.1388	0.2088	0.2325	0.0334	0.0967	0.1234	0.2330	0.1475	0.1370	<.0001
Macrophages M1	0.0238	0.0541	0.0300	0.0040	0.0668	0.0200	0.0131	0.0096	0.0569	<.0001
Macrophages M2	0.1565	0.1489	0.1775	0.2032	0.1026	0.1794	0.1700	0.2216	0.1401	<.0001
Dendritic cells resting	0.0000	0.0000	0.0000	0.0000	0.0000	0.0000	0.0000	0.0000	0.0000	0.0514
Dendritic cells activated	0.0000	0.0028	0.0263	0.0000	0.0081	0.0000	0.0000	0.0000	0.0000	0.0017
Mast cells resting	0.0379	0.0187	0.0310	0.1306	0.0166	0.0453	0.0406	0.0575	0.0236	<.0001
Mast cells activated	0.0000	0.0000	0.0000	0.0000	0.0000	0.0000	0.0000	0.0000	0.0000	0.0530
Eosinophils	0.0040	0.0034	0.0015	0.0199	0.0000	0.0246	0.0090	0.0032	0.0154	0.0036
Neutrophils	0.0000	0.0000	0.0000	0.0000	0.0000	0.0000	0.0000	0.0000	0.0000	0.6401

Supplementary Table 15: *x*Cell results in TNBCtype classified, unclassified and excluded tumors

	TNBC Type									
	All	Basal-like 1	Basal-like 2	Immuno-modulatory	Luminal Androgen Receptor	Mesenchymal	Mesenchymal Stem-like	TNBC Type Unclassified	TNBC Type Excluded	p-value
B cells naive	0.0310	0.0328	0.0226	0.0382	0.0552	0.0087	0.0239	0.0141	0.0406	0.0009
B cells memory	0.0000	0.0000	0.0002	0.0000	0.0079	0.0042	0.0000	0.0208	0.0090	0.0196
Plasma cells	0.0000	0.0000	0.0000	0.0017	0.0000	0.0000	0.0014	0.0000	0.0000	0.0078
T cells CD8	0.0175	0.0120	0.0036	0.0207	0.0305	0.0000	0.0091	0.0172	0.0093	0.0689
T cells CD4 naive	0.0000	0.0000	0.0000	0.0000	0.0000	0.0000	0.0000	0.0000	0.0000	0.7083
T cells CD4 memory resting	0.2597	0.2498	0.2643	0.2511	0.3170	0.3218	0.2521	0.1785	0.2613	0.0001
T cells CD4 memory activated	0.0238	0.0267	0.0337	0.0004	0.0461	0.0277	0.0000	0.0178	0.0177	<.0001
T cells follicular helper	0.0346	0.0592	0.0366	0.0084	0.0464	0.0219	0.0401	0.0074	0.0536	<.0001
T cells regulatory (Tregs)	0.0000	0.0000	0.0000	0.0000	0.0000	0.0000	0.0000	0.0000	0.0000	0.0423
T cells gamma delta	0.0000	0.0000	0.0000	0.0000	0.0000	0.0000	0.0000	0.0000	0.0000	0.1311
NK cells resting	0.0490	0.0411	0.0597	0.0472	0.0544	0.0651	0.0533	0.0443	0.0413	0.2667
NK cells activated	0.0000	0.0000	0.0000	0.0000	0.0000	0.0000	0.0000	0.0000	0.0000	0.0004
Monocytes	0.0320	0.0195	0.0182	0.0664	0.0247	0.0419	0.0378	0.0467	0.0249	<.0001
Macrophages M0	0.1388	0.2088	0.2325	0.0334	0.0967	0.1234	0.2330	0.1475	0.1370	<.0001
Macrophages M1	0.0238	0.0541	0.0300	0.0040	0.0668	0.0200	0.0131	0.0096	0.0569	<.0001
Macrophages M2	0.1565	0.1489	0.1775	0.2032	0.1026	0.1794	0.1700	0.2216	0.1401	<.0001
Dendritic cells resting	0.0000	0.0000	0.0000	0.0000	0.0000	0.0000	0.0000	0.0000	0.0000	0.0514
Dendritic cells activated	0.0000	0.0028	0.0263	0.0000	0.0081	0.0000	0.0000	0.0000	0.0000	0.0017
Mast cells resting	0.0379	0.0187	0.0310	0.1306	0.0166	0.0453	0.0406	0.0575	0.0236	<.0001
Mast cells activated	0.0000	0.0000	0.0000	0.0000	0.0000	0.0000	0.0000	0.0000	0.0000	0.0530
Eosinophils	0.0040	0.0034	0.0015	0.0199	0.0000	0.0246	0.0090	0.0032	0.0154	0.0036
Neutrophils	0.0000	0.0000	0.0000	0.0000	0.0000	0.0000	0.0000	0.0000	0.0000	0.6401

Supplementary Table 16. Results from multivariable Cox proportional hazards models of overall survival in triple negative breast cancer.

	HR (95% CI)	p-value	HR (95% CI)	p-value	HR (95% CI)	p-value	HR (95% CI)	p-value
**Stage at diagnosis (reference Stage I)**								
**Stage II**	1.9 (1.1,3.2)	0.0213	1.9 (1.1,3.2)	0.0225	3.1 (1.5,6.1)	0.0015	1.8 (1.1,3.1)	0.0309
**Stage III**	5.5 (2.7,11.1)	<.0001	4.7 (2.4,9.3)	<.0001	7.0 (3.0,16.4)	<.0001	5.2 (2.6,10.5)	<.0001
**Stage IV**	53.2 (19.6,144.3)	<.0001	45.9 (16.6,126.9)	<.0001	116.0 (33.5,401.9)	<.0001	62.2 (22.4,172.8)	<.0001
**Race (reference White)**								
**Black**	0.7 (0.4,1.1)	0.0944						
**Obesity Class (reference Lean)**								
**Obese I**			0.8 (0.5,1.4)	0.3998				
**Obese II**			0.4 (0.2,0.9)	0.0218				
**Obese III**			0.5 (0.2,1.0)	0.0445				
**TNBC Type (reference Immunomodulatory)**								
**Basal-like 1**					2.4 (0.9,6.9)	0.0894		
**Basal-like 2**					6.3 (2.3,17.4)	0.0004		
**Luminal Androgen Receptor**					3.2 (0.9,11.4)	0.0681		
**Mesenchymal**					5.2 (2.1,13.0)	0.0005		
**Mesenchymal Stem-like**					3.9 (1.4,10.5)	0.0084		
**Unclassified**					3.0 (1.1,8.0)	0.0279		
**B Cells Naive, 5% increase**							0.7 (0.6,1.0)	0.0221
**B Cells Memory, 5% increase**							0.9 (0.6,1.2)	0.3758

Abbreviations: HR= hazard ratio, CI= confidence interval, TNBC= triple negative breast cancer.

Supplementary Table 17: Expanded demographics, clinico-pathological and molecular characteristics of TNBC tumors

	All		Lean		Obese I		Obese II		Obese III		
	%	n	%	n	%	n	%	n	%	n	p-
All	100.0	253	100.0	77	100.0	80	100.0	51	100.0	45	
Age, y											0.0642
mean, std	62.2	12.8	63.8	14.5	63.6	12.7	58.4	11.3	61.0	10.5	
Age											0.5309
Less than 50	17.4	44	18.2	14	15.0	12	23.5	12	13.3	6	
50 or older	82.6	209	81.8	63	85.0	68	76.5	39	86.7	39	
Race											0.0046
White	50.6	128	35.1	27	51.3	41	60.8	31	64.4	29	
Black	49.4	125	64.9	50	48.8	39	39.2	20	35.6	16	
AJCC Stage											0.5790
I	41.1	104	40.3	31	45.0	36	43.1	22	33.3	15	
II	47.0	119	41.6	32	48.8	39	45.1	23	55.6	25	
III	9.1	23	13.0	10	5.0	4	9.8	5	8.9	4	
IV	2.8	7	5.2	4	1.3	1	2.0	1	2.2	1	
Late Stage											0.6295
No	41.1	104	40.3	31	45.0	36	43.1	22	33.3	15	
Yes	58.9	149	59.7	46	55.0	44	56.9	29	66.7	30	
Grade											0.2707
1	2.4	6	2.6	2	2.5	2	3.9	2	.	.	
2	11.5	29	7.8	6	13.8	11	9.8	5	15.6	7	
3	82.2	208	81.8	63	82.5	66	86.3	44	77.8	35	
Unknown	4.0	10	7.8	6	1.3	1	.	.	6.7	3	
2-year Survival											0.7240
No	13.4	34	16.9	13	12.5	10	9.8	5	13.3	6	
Yes	84.2	213	80.5	62	85.0	68	90.2	46	82.2	37	
Censored	2.4	6	2.6	2	2.5	2	.	.	4.4	2	
TNBC Type											0.2807
Basal-like 1	14.6	37	13.0	10	10.0	8	11.8	6	28.9	13	
Basal-like 2	7.1	18	10.4	8	2.5	2	5.9	3	11.1	5	
Immuno-modulatory	19.4	49	14.3	11	25.0	20	23.5	12	13.3	6	
Luminal Androgen	5.5	14	6.5	5	7.5	6	2.0	1	4.4	2	
Mesenchymal	13.8	35	13.0	10	12.5	10	13.7	7	17.8	8	
Mesenchymal Stem-like	16.6	42	16.9	13	20.0	16	17.7	9	8.9	4	
Unclassified	7.9	20	9.1	7	7.5	6	11.8	6	2.2	1	
Excluded	15.0	38	16.9	13	15.0	12	13.7	7	13.3	6	
Area Deprivation Index											0.0817
Median (IQR), n	76	251*	75	77	65	78*	72	51	81	45	

Abbreviations: BMI = body mass index, AJCC = American Joint Commission on Cancer, TNBC = Triple negative breast cancer, IQR = Inter-quartile range.

*Area deprivation index could not be computed for 2 patients.

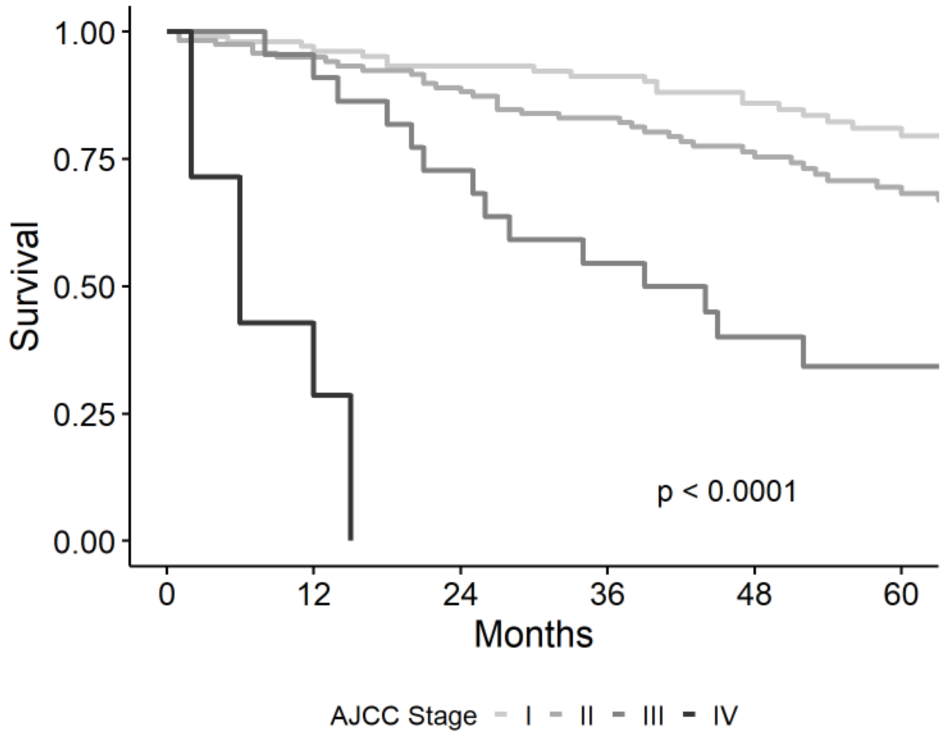

Supplementary Figure 1: Kaplan-Meyer curves showing that in our sample, stage at diagnosis (A) was strongly associated with survival.

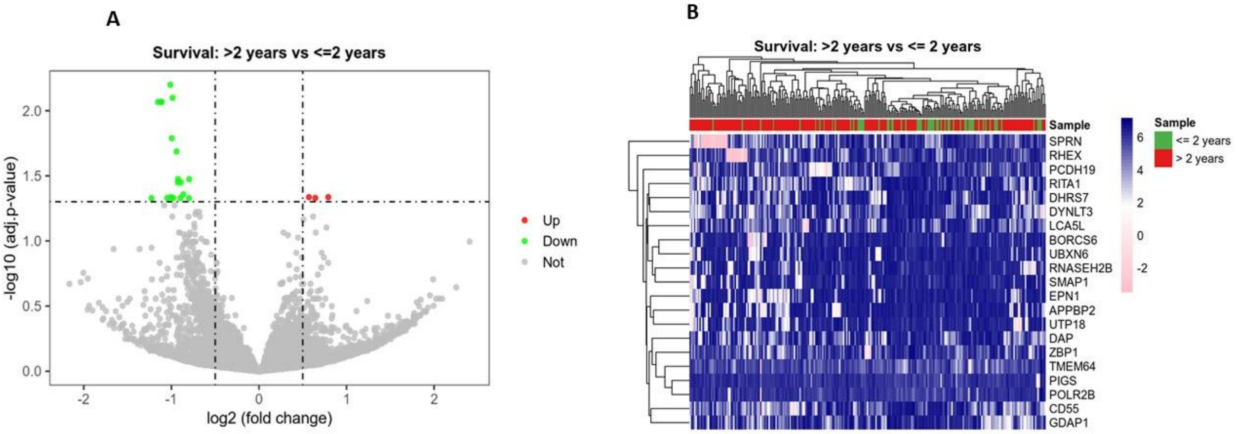

Supplementary Figure 2: A) Volcano plot and B) Heatmap showing transcripts significantly different between tumors fatal withing 2 years and tumors with survival > 2 years.

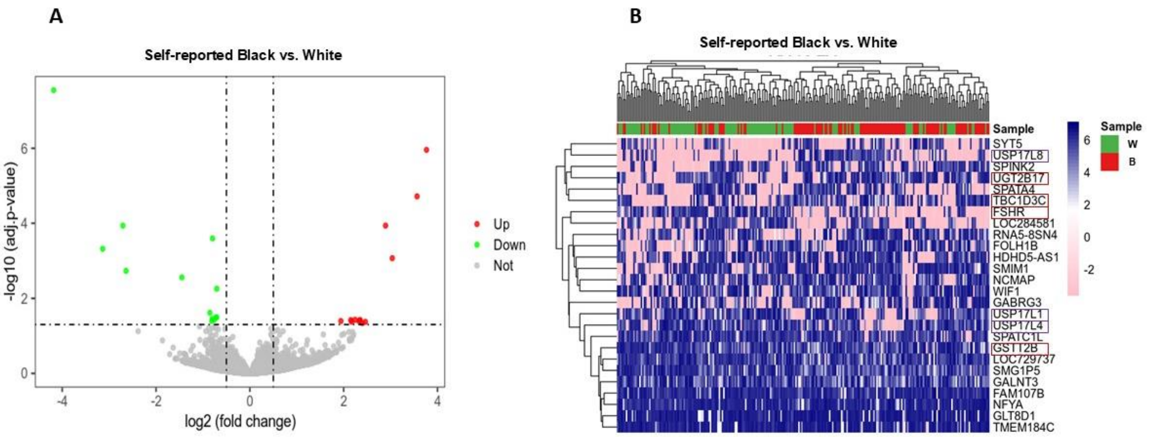

Supplementary Figure 3: A) Volcano plot and B) Heatmap showing transcripts significantly different between tumors from self-reported Black and White women.

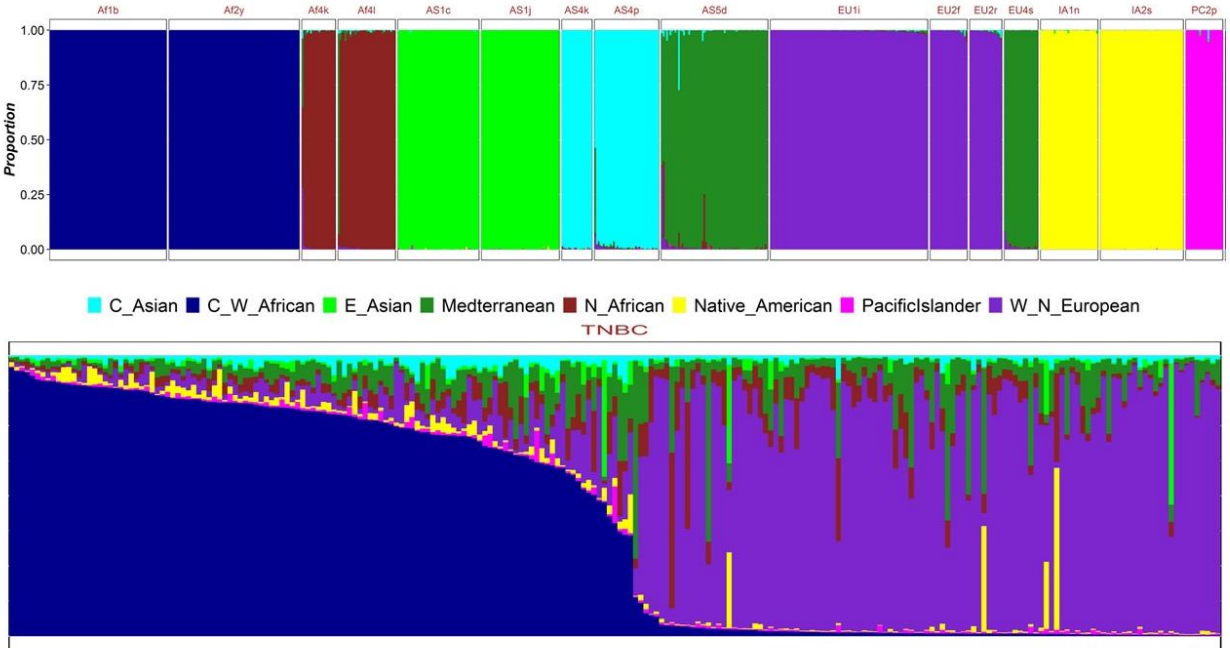

Supplementary Figure 4: Geographic ancestry distribution of our sample, based on supervised analysis using 8 pre-defined reference groups: Central Asian, Central and West African, East Asian, Mediterranean, North African, Native American, Pacific Islander and Western and Northern European. Top: reference groups. Bottom: Ancestry distribution in our sample.

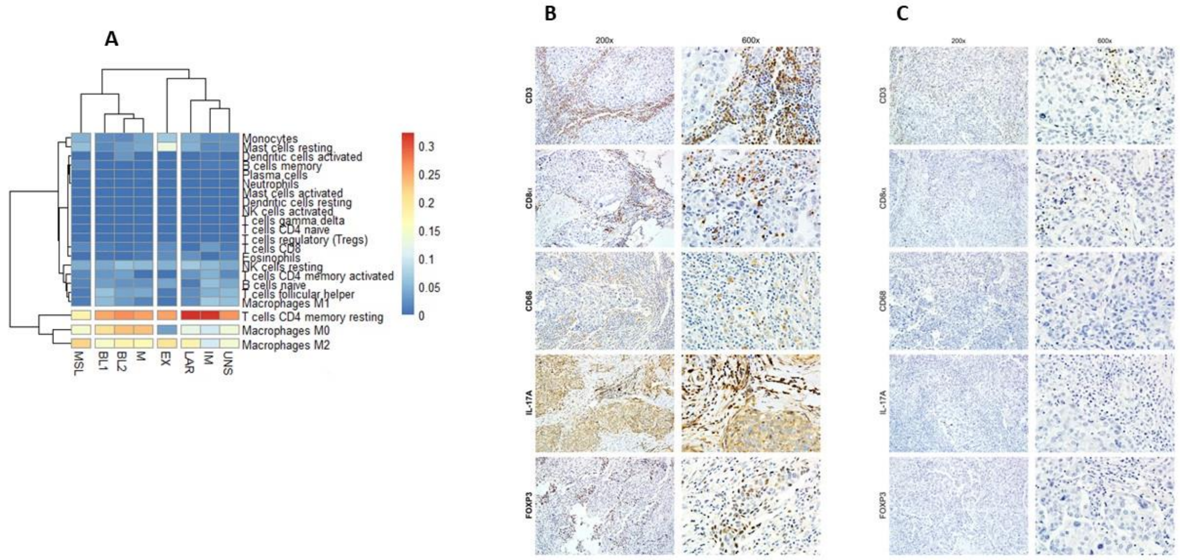

Supplementary Figure 5: A) Immune microenvironment composition as estimated by CIBERSORTx in tumors of different TNBC-type subtypes, including Excluded (EX) and Unclassified (UNS) tumors. B) Immunohistochemical validation of immune microenvironment populations, including pan-T-cells (CD3), CD8 T-cells (CD8α), macrophages (CD68), IL-17A-producing cells (potentially several populations, including CD8 Tc17 T-cells, mast cells, myeloid cells and others) and T-reg T-cells (FOXP3).
